# A Study of 3-Substituted 7-Methoxy-2,3,4,5-tetrahydro-1*H*-benzo[*d*]azepin-1-ols Leading to Candidate PET Radioligands for Imaging Brain GluN2B: Design, Synthesis, and Structure–Activity Relationships

**DOI:** 10.3390/molecules31091541

**Published:** 2026-05-06

**Authors:** Lisheng Cai, Leah Noelle Millard, Sean Wallace Costner, Alyssa Wang, Yonglan Liu, Victor William Pike

**Affiliations:** 1 PET Radiopharmaceutical Sciences Section, Molecular Imaging Branch, National Institute of Mental Health, National Institutes of Health, Bethesda, MD 20892, USA; leah.millard@nih.gov (L.N.M.); seancostner1@gmail.com (S.W.C.); ajw150@georgetown.edu (A.W.); pikev@mail.nih.gov (V.W.P.); 2 Bioinformatics and Computational Biosciences Branch, National Institute of Allergy and Infectious Diseases, National Institutes of Health, Bethesda, MD 20892, USA; yonglan.liu2@nih.gov

**Keywords:** GluN2B, NMDA receptor, structure–activity relationship, radioligand, PET

## Abstract

*N*-Methyl-D-aspartate (NMDA) receptors are ligand- and voltage-gated ion channels essential for synaptic plasticity, learning, and memory. The GluN2B subunit, highly expressed in the forebrain and spinal cord, is implicated in multiple neurological and psychiatric disorders, making it an attractive target for positron emission tomography (PET) imaging. However, the development of selective GluN2B PET radioligands remains challenging. Here, we describe the design, synthesis, and evaluation of eighteen 3-alkylaryl derivatives of 7-methoxy-2,3,4,5-tetrahydro-1*H*-benzo[*d*]azepin-1-ol, including enantiomerically resolved compounds, as candidate PET radioligands. Structure–activity relationship studies show that binding affinity is largely insensitive to electronic and steric variation at the terminal aryl group but strongly dependent on alkyl linker length, with a four-carbon chain providing optimal affinity. Binding affinity does not correlate with calculated lipophilicity, suggesting hydrophobicity is not the primary determinant of receptor interaction. Absolute configuration was established using vibrational circular dichroism and infrared spectroscopy, and docking studies provided insight into enantiomer-specific binding modes. Two ligands, **L3** and **L6**, and their enantiomers exhibited high GluN2B affinity, favorable physicochemical properties, and suitability for carbon-11 labeling. Separate PET imaging studies confirmed strong and specific brain binding of the radiolabeled compounds. These findings establish this scaffold as a promising platform for GluN2B PET ligand development.

## 1. Introduction

*N*-Methyl-D-aspartate (NMDA) receptors are ligand- and voltage-gated ion channels that mediate synaptic Ca^2+^ and Na^+^ influx and K^+^ efflux. They play central roles in synaptic plasticity, learning, and memory [1]. NMDA receptors are widely expressed throughout the central nervous system and are implicated in the pathophysiology of numerous neurological and psychiatric disorders, making them important therapeutic targets [2,3,4].

Functional NMDA receptors are hetero-tetrameric complexes assembled from GluN1, GluN2 (A–D), and GluN3 (A or B) subunits. The diversity of subunit composition generates receptor subtypes with distinct structural, physiological, and pharmacological properties. NMDA receptors possess multiple ligand-binding domains, including sites for L-glutamate, glycine/D-serine, polyamines, Mg^2+^, Zn^2+^, and channel blockers such as phencyclidine [1]. Clinically used NMDA-targeting drugs include memantine, which is approved for the treatment of Alzheimer’s disease [5,6].

Among NMDA receptor subunits, GluN2B has attracted particular interest. GluN2B-enriched receptors are predominantly expressed in the forebrain and dorsal horn of the spinal cord and are implicated in schizophrenia, stroke, neurodegeneration, and neuropathic pain [7,8,9]. Selective targeting of GluN2B, rather than non-selective inhibition of NMDA receptors, offers the possibility of therapeutic efficacy while minimizing adverse effects, such as hallucinations, sedation, and cognitive impairment.

Positron emission tomography (PET) enables non-invasive quantification of specific molecular targets in the living brain using radiolabeled ligands. PET imaging, therefore, has substantial potential to elucidate disease mechanisms in neurological and psychiatric disorders [10,11,12,13] and to support drug development through target engagement studies [14,15]. Any radioligand for brain PET imaging must incorporate a short-lived positron emitter such as carbon-11 (*t*_1/2_ = 20.4 min) or fluorine-18 (*t*_1/2_ = 109.8 min) and satisfy stringent requirements regarding affinity, selectivity, lipophilicity, metabolic stability, and brain penetration [16,17,18]. PET radioligands for robustly quantifying brain GluN2B would serve as valuable tools for translational research and for drug development [19].

**Chart 1:**
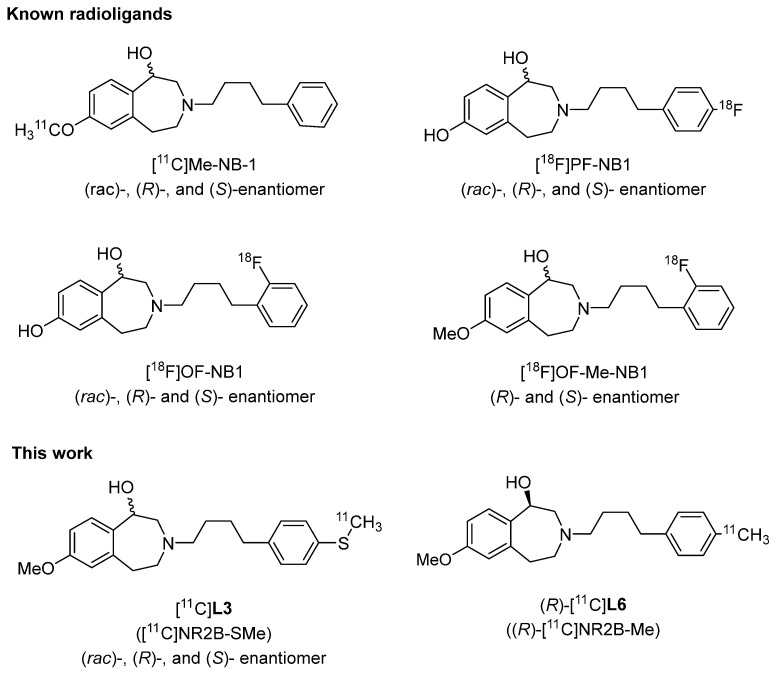
Radioligands evaluated for PET imaging of brain GluN2B in animals and humans.

Despite decades of effort, the development of successful PET radioligands for NMDA receptors has proven challenging [19,20,21]. To date, only radioligands based on a 3-(4-phenylbutyl)-2,3,4,5-tetrahydro-1*H*-benzo[*d*]azepine-1,7-diol framework have demonstrated clearly displaceable GluN2B signals in vivo [22], with only [^11^C](*R*)-Me-NB-1 (Chart 1) evaluated in humans [23]. [^11^C](*R*)-Me-NB-1 performed well but has unexpectedly high uptake in cerebellar gray matter. The development of alternative GluN2B radioligands could be useful in providing tools for understanding [^11^C](*R*)-Me-NB-1 uptake in primate cerebellum.

**Chart 2:**
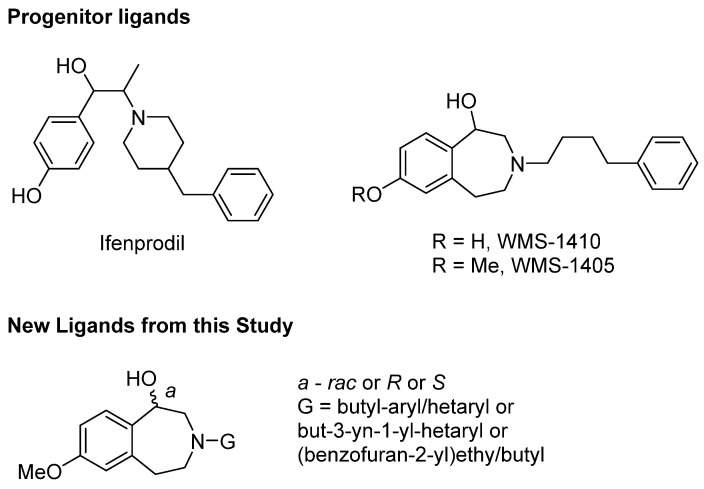
Progenitor GluN2B ligands and new ligands described in this study.

Here, we report our systematic investigation of structural variations at the 3-position of 7-methoxy-2,3,4,5-tetrahydro-1*H*-benzo[*d*]azepin-1-ol aimed at optimizing GluN2B ligands for PET imaging (Chart 2). Through synthesis and pharmacological evaluation of these derivatives, we sought to expand structure–activity knowledge and identify ligands with properties favorable for translation into GluN2B PET radioligands. This study produced the enantiomers of [^11^C]NR2B-SMe ([^11^C]**L3**) [24] and [^11^C]NR2B-Me ([^11^C]**L6**) [25,26] (Chart 1), which exhibited nanomolar affinity for GluN2B, favorable lipophilicity, and suitability for carbon-11 labeling. In our separately published PET studies, the [^11^C]**L3** enantiomers demonstrated strong specific binding in rat brain [24], and [^11^C](*R*)-**L6** showed high binding potential in monkeys [26].

## 2. Results and Discussion

### 2.1. Ligand Design 

X-ray crystallography [27] has shown that the prototypical GluN2B ligand, ifenprodil (Chart 2), binds at the interface of GluN1 and GluN2B subunits in the NMDA receptor [28] by forming interactions critical for affinity and subtype selectivity. Key interactions include hydrogen bonding between the phenolic hydroxyl group and Glu236, and between the protonated amine and Gln210 of GluN2B. Additional aromatic and hydrophobic interactions involving residues from both GluN1 and GluN2B further stabilize binding (see Supporting Information for docking of (1S,2R)-ifenprodil to GluN2B/GluN1).

A chemical scaffold underlying many recent GluN2B ligands derives from WMS-1410, a conformationally constrained analog of ifenprodil (Chart 2). WMS-1410 preserves key pharmacophoric elements while enhancing GluN2B selectivity [29]. Docking studies have indicated that WMS-1410 adopts a similar GluN2B binding pose to ifenprodil [30]. Importantly, the 2,3,4,5-tetrahydro-1*H*-benzo[*d*]azepine-1,7-diol scaffold of WMS-1410 confers high affinity and selectivity for GluN2B over other NMDA receptor subtypes and over a broad panel of off-target proteins [31,32].

The phenolic hydroxy group in WMS-1410 undergoes glucuronidation in vivo, limiting bioavailability [33]. Replacement of the 7-hydroxy group with a methoxy group, as in WMS-1405 (Chart 2), blocks glucuronidation and introduces a convenient site for ^11^C-methylation. (*R*)-[^11^C]Me-NB-1, a radiolabeled derivative of WMS-1405, demonstrated high GluN2B affinity and specific binding in vivo [23,34]. Thus, the 7-methoxy substitution of WMS-1405 represents a viable platform for GluN2B PET radioligand development.

Both ifenprodil and WMS-1410 exhibit pronounced stereoselectivity: (1*R*,2*R*)-ifenprodil displays approximately 2.5-fold higher affinity (*K*_i_ = 5.8 nM) than its (1*S*,2*S*) antipode [35], whereas the *R*-enantiomer of WMS-1410 (*K*_i_ = 30 nM) is substantially more potent than the *S*-enantiomer (*K*_i_ = 740 nM) [33]. These findings underscore the importance of stereochemical orientation for productive binding within the GluN2B allosteric site.

*N*-Substituted 7-hydroxy and 7-methoxy derivatives of 2,3,4,5-tetrahydro-1*H*-benzo[*d*]azepin-1-ol and their enantiomers are among the most potent and selective GluN2B ligands. These scaffolds have served as the basis for several candidate PET radioligands, not only [^11^C](*R*)-Me-NB-1 [23,34], but also [^18^F](*S*)-oF-NB1 [26], and [^18^F]PF-NB1 [36] (Chart 1). Here, we built on this scaffold in a search for improved PET radioligands.

This study is the first systematic evaluation of alkyl tether length and terminal aryl substitution at the 3-position in this scaffold. We focused on systematic modification of the alkyl-tethered terminal aryl group. Specifically, we examined: (i) the effect of para-substitution on the terminal phenyl ring, (ii) variation in alkyl linker length to probe spatial tolerance within the binding pocket, and (iii) introduction of unsaturation within the linker to modulate conformational flexibility and electronic properties (Chart 2). Through these targeted structural variations, we sought to refine structure–activity relationships and identify ligands optimized for high-affinity binding, favorable physicochemical properties, and suitability for PET radiolabeling.

### 2.2. Ligand Syntheses 

**Scheme 1:**
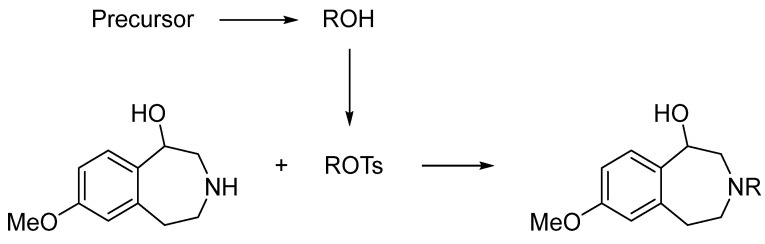
Strategy for ligand synthesis.

The overall strategy for ligand synthesis was to couple a requisite alkyl tosylate with 7-methoxy-2,3,4,5-tetrahydro-1*H*-benzo[*d*]azepin-1-ol (Scheme 1). Tosylates were prepared from the corresponding alcohols that were either purchased directly or obtained by various methods from commercially available starting materials, as described in the Supporting Information.

**Scheme 2:**
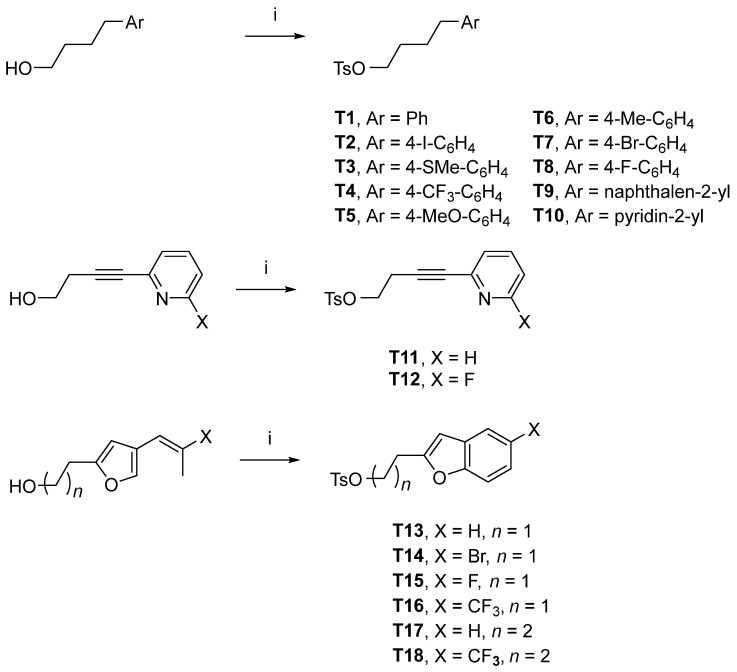
Syntheses of tosylates **T1**–**T18**. Reagents, conditions, and yields: (i) TsCl, Et_3_N, DCM, RT, overnight–24 h (**T1**, 77%; **T2**, 61%; **T3**, 47%; **T4**, 74%; **T5**, 21%; **T6**, 24%; and **T7**, 58% after 5 d of stirring; **T8**, 52%; **T9**, 22%; **T10**, 21%; **T11**, 22%; **T12**, 13%; **T13**, 45%; **T14**, 54%; **T15**, 72%; **T16**, 39%; **T17**, 76%; and **T18**, 56%).

Treatment of the synthesized alcohols with tosyl chloride in the presence of base gave the tosylates, **T1**–**T18,** in yields ranging from 13 to 77% (Scheme 2). Some reactions did not go to completion even with excess reagent. A long reaction time risked reduced yield from the substitution of the tosylate group from the product by the chloride anion generated from the tosyl chloride. Normally, overnight reactions gave the best compromise between yield and purity.

**Scheme 3:**
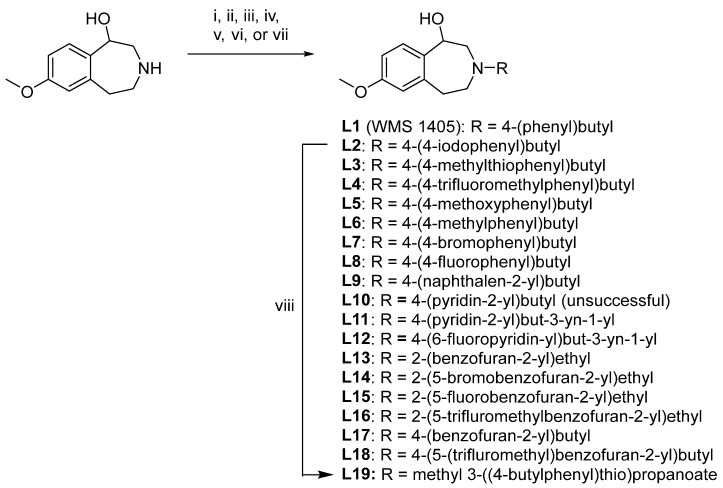
Syntheses of racemic ligands **L1**–**L9** and **L11**–**L19**. Reagents, conditions and yields: (i) ROTs (**T1**), K_2_CO_3_, MeCN, 6 d at reflux. (**L1**, 72%). (ii) ROTs (**T2**), K_2_CO_3_, MeCN, 5 d at 75 °C. (**L2**, 73%). (iii) ROTs (**T3** or **T4**), K_2_CO_3_, MeCN, 2 d at 75 °C. (**L3**, 75%; **L4**, 71%). (iv) ROTs (**T5**, **T6**, or **T8**), K_2_CO_3_, MeCN, 2 d at 90 °C. (**L5**, 87%; **L6**, 37%; **L8**, 79%). (v) ROTs (**T7**, **T9**, or **T11**), K_2_CO_3_, MeCN, 5 d at 90 °C. (**L7**, 62%; **L9**, 74%; **L11**, 77%). (vi) ROTs (**T12**, **T14**, **T15**, **T16**, **T17**, or **T18**) MW (135 °C, 10 min, 60 W, 250 psi). (**L12**, 12%; **L14**, 51%; **L15**, 59%; **L16**, 64%; **L17**, 71%; **L18**, 68%). (vii) ROTs (**T13**), Na_2_HPO_4_, MeCN, 22 d at 90 °C. (**L13**, 71%). (viii) **L2**, HSCH_2_CH_2_CO_2_Me, Me_2_NSnMe_3_, Pd(PPh_3_)_4_, DMSO, MW (110 °C, 50 W, 250 psi) (**L19**, 86%).

For the syntheses of the prospective GluN2B ligands, treatment of the prepared tosylates with 7-methoxy-2,3,4,5-tetrahydro-1*H*-benzo[*d*]azepin-1-ol was generally successful (Scheme 3), except in two cases, namely **L10** and **L12**, which likely resulted in competing cyclization reactions.

**Scheme 4:**
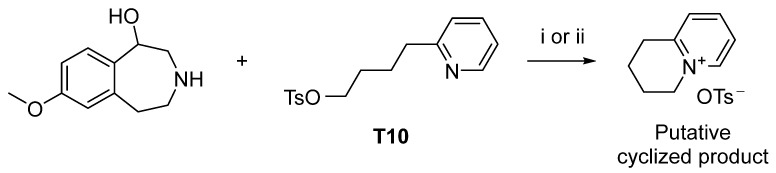
Attempted synthesis of **L10**. Reagents, conditions and yields: (i) Na_2_HPO_4_, DMSO, MW (80 °C, 10 min, 30 W, 250 psi, or 120 °C, 10 min, 50 W, 250 psi, or 150 °C, 10 min, 60 W, 250 psi). (No **L10**; putative cyclized product only). (ii) Na_2_HPO_4_, MeCN, 90 °C for 22 d. (No **L10**; putative cyclized product only).

When **T10** (R = 4-(pyridin-2-yl)butyl) was used in the synthesis, only an unexpected byproduct was observed (Scheme 4). LC-MS indicated that this was a cyclic product from an intramolecular attack on the tosylate group by the nucleophilic pyridinyl nitrogen. Changing the concentration of **T10**, including the use of pure **T10** as a solvent, did not generate any of the desired ligand (**L10**). No further attempt was made to synthesize **L10**.

When 7-methoxy-2,3,4,5-tetrahydro-1*H*-benzo[*d*]azepin-1-ol was treated with **T12** in the presence of disodium hydrogen phosphate as base, **L12** was produced in low yield. An appreciable by-product was also obtained. This was probably a cyclic product from intramolecular substitution of the 6-fluoro group by the remote hydroxy group (probably as its phenoxide) in **L12**. The use of other bases, such as sodium carbonate or bicarbonate, did not improve the yield of **L12**. These outcomes further underscore the possible susceptibility of pyridinyl-containing tosylates to intramolecular cyclization, as likely seen in the attempted synthesis of **L10**.

### 2.3. Precursors for ^11^C-Labeling 

**Scheme 5:**
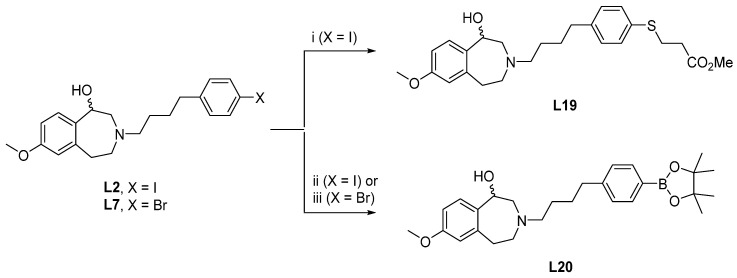
Syntheses of precursors **L19** and **L20**. Reagents, conditions, and yields: (i) HSCH_2_CH_2_CO_2_Me, Me_2_NSnMe_3_, Pd(PPh_3_)_4_, DMSO, MW (110 °C, 10 min, 50 W, 250 psi). (86% from **L2**). (ii) B_2_Pin_2_, Pd(DPPF)Cl_2_^●^CH_2_Cl_2_, DMSO, MW (80 °C, 60 min, 60 W, 250 psi). (37% from **L2**). (iii) B_2_Pin_2_, Pd(DPPF)Cl_2_^●^CH_2_Cl_2_, DMSO, MW (110 °C, 30 min, 50 W, 250 psi). (54% from **L7**).

The methyl arylthiopropanoate esters **L19** and its enantiomers were synthesized from **L2** and its enantiomers, respectively, and purified by preparative chiral HPLC (see Experimental and Supporting Information) (Scheme 5).

**L20** was synthesized by the substitution of either the iodo or bromo substituent in **L2** or **L7**, respectively, with Bpin using Pd(DPPF)Cl_2_^●^CH_2_Cl_2_ as a catalyst under microwave heating conditions (Scheme 5). The enantiomers of **L20** were obtained by chiral resolution (see Experimental and Supporting Information).

### 2.4. Absolute Configuration Correlation 

**Scheme 6:**
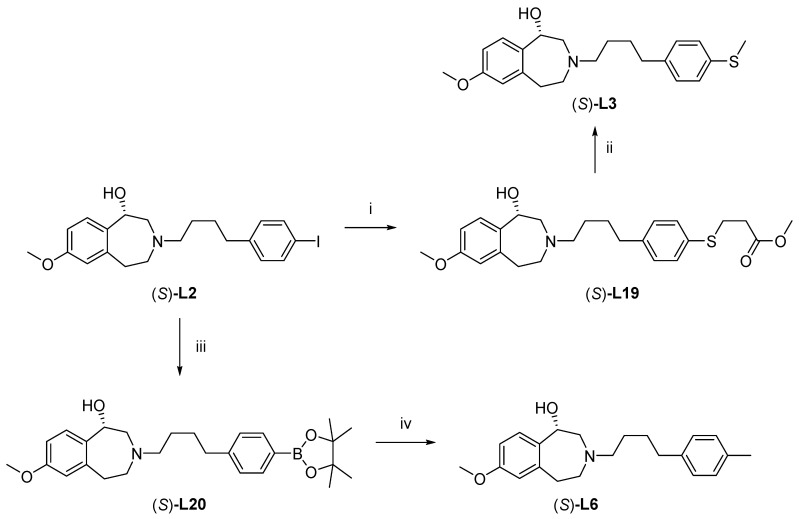
Determination of the absolute configurations of **L3**, **L6**, **L19**, and **L20** enantiomers by relation to the absolute configuration of (*S*)-**L3**, determined by VCD/IR and stereo-retentive reactions. Reagents, conditions, and yields: (i) Me_3_SnSCH_2_CH_2_CO_2_Me, Pd(DPPF)Cl_2,_ Et_3_N, MeCN, MW (90 °C, 20 min, 120 W, 250 psi), 84%; (ii) MeI, TBAOH, DCM, rt, 20 min, 75%; (iii) B_2_Pin_2_, KOAc, Pd(DPPF)Cl_2_, MeCN, MW (80 °C. 20 min, 150 W, 250 psi), 31%; and (iv) MeI, CsF, Pd(PPh_3_)_4_, MeOH/MeCN, MW (90 °C. 20 min, 80 W, 250 psi). 84%.

We used vibrational circular dichroism/infrared (VCD/IR) plus quantum calculations to determine the absolute configuration of the enantiomers of ligand **L3** (see Supporting Information) [24]. Four homochiral ligands (enantiomers of **L2**, **L6**, **L19**, and **L20**) were related to (*S*)-**L3** through stereo-retentive chemical reactions, thereby allowing unequivocal assignment of their absolute configurations (Scheme 6). In each case, we observed that the early eluting enantiomers of these ligands on chiral HPLC analysis with the (*S*,*S*)-Whelk column had the same absolute configuration, which we determined to be *S*. We also observed that each enantiomer of **L3** has opposite specific optical rotation sign in ethanol and chloroform (see Section 3.3.4), which had to be considered in the VCD/IR analysis.

The chemical transformations shown in Scheme 6 did not cause any appreciable racemization of the products formed from homochiral starting materials.

### 2.5. Ligand Structure Versus Affinity for GluN2B 

A total of 18 racemic ligands and a few enantiomers were variously evaluated for binding to rat brain GluN2B through in vitro binding assays with [^3^H]ifenprodil as reference radioligand, including the manual and automatic assays at the Psychoactive Drug Screening Program (PDSP) and an in-house binding assay with rat brain homogenate (Table 1). The manual PDSP assay gave three to tenfold lower *K*_i_ values than the automated assay, but a similar ranking of ligand affinities. The in-house assay gave lower *K*_i_ values than the PDSP assay. Nonetheless, the results are very useful for ranking and comparing binding affinities for the whole set of ligands.

We systematically varied substituents on the terminal aryl group and the length and composition of the alkyl linker to define structure–activity relationships within this series. Among ligands bearing a para-substituted phenylbutyl group (**L2**–**L8**), binding affinities varied only modestly (20–44 nM) across a range of substituents (SMe, OMe, H, Me, Br, F), indicating that the GluN2B binding pocket is largely insensitive to electronic and steric variation at the aryl terminus. Even bulky substituents, such as naphthalenyl (**L11**), were well tolerated. In contrast, introduction of a terminal hydrogen bond acceptor (e.g., pyridinyl groups in **L11** and **L12**) markedly reduced affinity, consistent with a predominantly hydrophobic interaction in this region of the binding pocket.

In contrast to the limited influence of aryl substitution, linker length had a pronounced effect on binding affinity. Comparisons across series (e.g., **L13** vs. **L17** and **L16** vs. **L18**) indicate that a flexible four-carbon tether provides optimal positioning of the terminal aryl group within the binding site. Shorter or more constrained linkers resulted in reduced affinity, highlighting the importance of spatial alignment over electronic effects in governing ligand–receptor interactions.

Notably, counter to typical CNS ligand expectations, no meaningful correlation was found between binding affinity and calculated lipophilicity (clog*D*) (see Supporting Information, Figure S120). This indicates that global hydrophobicity is not a dominant determinant of binding. This finding further supports the view that specific geometric and local interactions, rather than bulk physicochemical properties, govern productive engagement with the GluN2B binding pocket.

Together, these results identify linker length as the primary determinant of affinity in this series, with the binding pocket exhibiting substantial tolerance to variation in terminal aryl substitution.

### 2.6. Ligand Binding Poses and Interactions with the Target GluN2B Complex 

**Figure 1:**
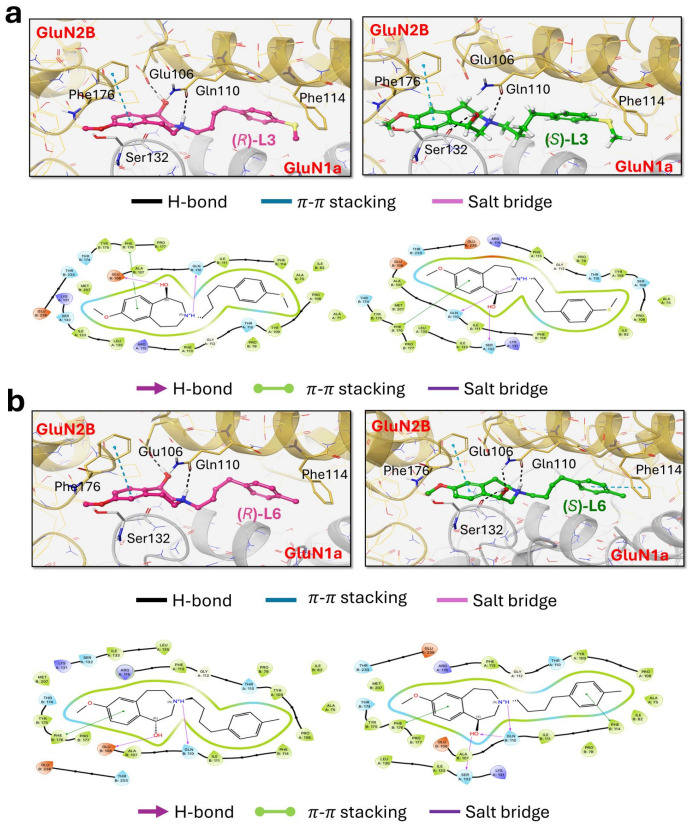
Binding poses and interactions of (**a**) (*R*)- and (*S*)-**L3** and (**b**) (*R*)- and (*S*)-**L6** with the GluN1a/GluN2B complex. In the 3D interaction representations, GluN1a and GluN2B are shown in light gray and yellow, respectively, and residues involved in key interactions are highlighted as sticks. In the 2D interaction diagrams, negatively charged, positively charged, hydrophobic, and hydrophilic residues are colored dark orange, blue, green, and cyan, respectively.

Molecular docking was performed to predict the binding poses and the interactions of **L3** and **L6** with the GluN1a/GluN2B complex. The protonated amine in both the *R*- and *S*-configurations of **L3** (Figure 1a) and **L6** (Figure 1b) forms a hydrogen bond with Gln110 of GluN2B, consistent with the interaction observed in ifenprodil binding (Supporting Information Figure S118). The hydroxyl group adopts distinct orientations depending on stereochemistry. In the *S*-configuration, the hydroxyl group is oriented toward GluN1a and forms hydrogen bonds with the backbone of Ser132 in GluN1a and with Gln110 of GluN2B, in agreement with the X-ray structure of the ifenprodil-bound complex [27]. In contrast, in the *R*-configuration, the hydroxyl group flips in the opposite direction and forms a hydrogen bond with Glu106 of GluN2B. Another conserved interaction is the π–π stacking between the benzene ring of the methoxyphenyl group and Phe176 of GluN2B within the binding pocket. In ifenprodil, the phenolic hydroxyl group forms a hydrogen bond with Glu236 of GluN2B. However, in **L3** and **L6**, this group is replaced by a methoxy substituent, resulting in the loss of this interaction. The variable regions of **L3** and **L6** participate in conserved hydrophobic interactions with Ala75, Pro106, and Tyr109 of GluN1a, as well as Ile82, Ile111, Phe114, and Pro78 of GluN2B.

**L19** contains a long terminal *p*-S-CH_2_CH_2_CO_2_Me group that might extend toward Phe114 of GluN2B, potentially leading to steric clashes. However, **L19** still exhibits high binding affinity (Table 1). This suggests that **L19** may adopt a distinct binding mode or interaction pattern within the pocket. To account for receptor flexibility during ligand binding, Induced Fit Docking (IFD) was applied. As expected, the resulting binding poses of **L19** were diverse, with the long terminal *p*-S-CH_2_CH_2_CO_2_Me group displaying significant conformational flexibility (Supplementary Figures S121 and S122). In the binding, in addition to hydrogen bond interactions involving the protonated amine and hydroxyl group of **L19** with Gln110 of GluN2B (and) Ser132 of GluN1a (observed for (*S*)-**L19**), the central benzene ring adopts a rotated orientation that enables π–π stacking with Tyr109 of GluN1a (Supplementary Figures S121b and S122b). Furthermore, the protonated amine is likely to form a salt bridge with Glu106 of GluN2B. The terminal carbonyl oxygen might be able to position in a region favorable for hydrogen bonding with Gln110 (Supplementary Figures S121c and S122c).

Overall, these results indicate that **L3** and **L6** largely preserve the key interaction network observed for ifenprodil, whereas **L19** adopts a more flexible binding mode that accommodates its extended substituent without compromising binding affinity.

### 2.7. PET Radioligand Selection and Synthesis 

From a total of eighteen new ligands prepared in this study, we selected two ligands and their enantiomers for labeling with carbon-11 and evaluation as GluN2B PET radioligands in animals, namely **L3** (NR2B-SMe) [24] and **L6** (NR2B-Me) [25]. These two ligands were selected based on a combination of their high affinities for GluN2B, acceptable lipophilicities (moderate clog*D* values), acceptable CNS PET MPO scores, and amenability to labeling with carbon-11 at a methyl group (Table 1).

**Scheme 7:**
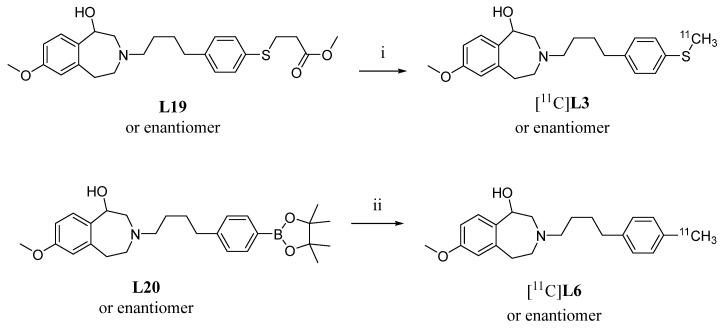
Radiosyntheses of [^11^C]**L3** and [^11^C]**L6** and their enantiomers. Reagents, conditions, and yields: (i) [^11^C]CH_3_I, 1 M TBAOH in MeOH; DMF, RT, 5 min.; 15−20% for a product formulated for i.v. injection from cyclotron-produced [^11^C]CO_2_ [24]. (ii) [^11^C]CH_3_I, CsF in MeOH, Pd_2_(dba)_3_, P(2.4-dmp)_3_, 80 °C, 5 min; 20−30% for product formulated for i.v. injection from [^11^C]CO_2_ [25].

We have previously reported the labeling of **L3** (NR2B-SMe) and its enantiomers in the *S*-methyl position with carbon-11 for their evaluation as GluN2B radioligands in rat brain, as summarized in Scheme 7 [24]. Labeling was achieved readily by treating the methyl thiopropanoate precursor (**L19** or an enantiomer) with no-carrier-added [^11^C]methyl iodide, produced from cyclotron-produced [^11^C]carbon dioxide, as fully described in the Supporting Information of [24]. No racemization occurred during the radiosyntheses from homochiral precursors, and, therefore, there was no need to use chiral chromatography for radioligand purification.

[^11^C]**L6** ([^11^C]NR2BMe) and its enantiomers have been labeled by palladium-mediated treatment of the boronic ester precursor **L20** or enantiomer with [^11^C]methyl iodide (Scheme 7), as fully described in the Supporting Information of [25]. Again, no racemization occurred during the radiosyntheses from homochiral precursors.

### 2.8. PET Radioligand Performance 

Both enantiomers of [^11^C]**L3** performed well as radioligands for PET imaging in rats [24]. [^11^C](*S*)-**L3** demonstrated high initial brain uptake, specific binding (up to 90% blockable), absence of radiometabolites in the brain, and favorable characteristics for further evaluation in higher species. [^11^C](*R*)-**L3** showed similar behavior, albeit with slightly faster washout characteristics.

[^11^C]**L6** ([^11^C]NR2BMe) and its enantiomers also entered the rat brain avidly to give receptor-specific signals that were uncontaminated by radiometabolites. Unexpectedly high binding was seen in the cerebellum with these radioligands, as for all other radioligands reported for PET imaging of GluN2B. This binding was determined not to be off-site binding to σ_1_ receptors [25], consistent with in vitro measurements of the low binding affinities of **L6** and several related ligands from this study for σ_1_ and σ_2_ receptors (Supporting Information Table S2). Notably, [^11^C](*R*)-**L6** demonstrated good uptake and high specific binding in the rhesus monkey brain. [^11^C](*R*)-**L6** exhibited favorable regional non-displaceable binding potential (specific to non-specific binding ratio; *BP*_ND_) values of 2 to 3 in a published head-to-head comparison with other radioligands, including [^18^F]OF-Me-NB1 and [^18^F]OF-NB1 [26]. Notably, all three radioligands showed similar brain distribution of radioactivity, including displaceable high uptake in the cerebellum. The source of this unexpected binding requires deeper investigation.

## 3. Materials and Methods

### 3.1. Starting Materials

7-Methoxy-2,3,4,5-tetrahydro-1*H*-benzo[*d*]azepin-1-ol was obtained from Ambeed Inc. (Arlington Heights, IL, USA). 4-Phenyl-1-butanol, 4-(4-methoxyphenyl)-1-butanol, 4-tolylbutan-1-ol, 4-(4-bromophenyl)butan-1-ol, 4-(4-fluorophenyl)butan-1-ol, 4-(*p*-iodophenyl)butyric acid, 3-butyn-1-ol, 5-hexyn-1-ol, 2-iodophenol, 4-iodobenzotrifluoride, diborane, triethylamine, *bis*(triphenylphosphine)palladium(II) dichloride, (Pd(DPPF)Cl_2_), 1,1′-bis(diphenylphosphino)ferrocene]dichloropalladium(II)·CH_2_Cl_2_, (Pd(DPPF)Cl_2_^.^CH_2_Cl_2_), and 4-(4-nitrophenyl)-1-butanol, were obtained from Aldrich (St Louis, MO, USA). 2-Naphthalenebutanol was obtained from AstaTech (Bristol, PA, USA). (Pyridin-2-yl)but-3-yn-1-ol, 2-bromo-6-fluoropyridine, 4-bromo-2-iodophenol, 4-fluoro-2-iodophenol, and 2-iodo-4(trifluoromethyl)-phenol were obtained from Combi-Blocks (San Diego, CA, USA). The syntheses of other alcohols are described in Supplementary Information.

### 3.2. Instruments and General Methods

A microwave apparatus (Discover CP-D; CEM; Matthews, NC, USA) was used for microwave-promoted reactions where indicated. A Combi-Flash apparatus (Teledyne Labs, Lincoln, NE, USA) with a silica gel cartridge (4–80 g) was used in compound purifications, as later indicated. A Centrifan PE apparatus (Fisher Scientific; Waltham, MA, USA) was used for drying many isolated compounds. Melting points were measured with a glass capillary on a digital melting point apparatus (SMP 20; Cole-Parmer Ltd., Stone, UK). Preparative, analytical, and chiral HPLC methods are detailed in the Supporting Information (general methods A–F). Compound purities were calculated as the area percentage of all chromatogram peaks. HRMS (ESI or EI) was acquired from the Mass Spectrometry Lab (University of Illinois Urbana-Champaign, https://illinois.edu/ accessed on 6 May 2026). Optical rotation measurements were performed on a JP-1010 Polarimeter (Jasco Inc; Easton, MD, USA).

LC-MS was performed on an Acquity UPLC M-Class System (Waters; Milford, MA, USA), fitted with a UPLC BEH column (1.7 μm, 2.1 × 50 mm) eluted at 0.4 mL/min with 0.1% formic acid in H_2_O (A)/0.1% formic acid in acetonitrile (B) (B from 5 to 90% over 7 min).

^1^H-NMR spectra were obtained at 400 MHz and ^13^C-NMR spectra at 101 MHz at room temperature (RT) on a multinuclear instrument (Bruker Biospin Corp; Billerica, MA, USA) in deuterated solvent. TMS (*δ* = 0 ppm) was used as an internal standard for ^1^H and ^13^C-NMR spectroscopy. The abbreviations s, d, t, m, q, quint, dd, dt, brs, vs, vt, and AB denote singlet, doublet, triplet, multiplet, quartet, quintet, double doublet, double triplet, broad singlet, virtual singlet, virtual triplet, and AB coupling, respectively.

### 3.3. Chemistry

#### 3.3.1. Syntheses of Tosylates T1−T18

4-Phenylbutyl 4-methylbenzenesulfonate (**T1**). 4-Phenylbutan-1-ol (6.5 mL, *d* = 0.984 g/mL, 42.6 mmol), tosyl chloride (15.11 g, 79.3 mmol), and Et_3_N (21 mL, 151 mmol) were dissolved in dichlormethane (DCM;100 mL) and stirred at RT overnight. A saturated sodium bicarbonate solution (200 mL) was added and then extracted with DCM. The organic layer was dried (MgSO_4_), filtered, and then added to silica gel (60 mL). The solvent was removed under vacuum. The residue was purified (Combi-Flash, silica gel, hexane/ethyl acetate) to give **T1** as a colorless wax (10.0 g, yield 77%). ^1^H-NMR (CDCl_3_): *δ* 7.78 (d, ^3^*J*_HH_ = 12 Hz, 2H, Ar-*H*), 7.33 (d, ^3^*J*_HH_ = 8.0 Hz, 2H, Ar-*H*), 7.26 (vt, ^3^*J*_HH_ = 8.0 Hz, 2H, Ar-*H*), 7.17 (t, ^3^*J*_HH_ = 8.0 Hz, 1H, Ar-*H*), 7.10 (d, ^3^*J*_HH_ = 8.0 Hz, 2H, Ar-*H*), 4.04 (t, ^3^*J*_HH_ = 6.0 Hz, 2H, C*H_2_*O), 2.56 (t, ^3^*J*_HH_ = 6.0 Hz, 2H, C*H_2_*), 2.45 (s, 3H, C*H_3_*), and 1.66 (m, 4H, C*H_2_*C*H_2_*). ^13^C{^1^H}-NMR (CDCl_3_): *δ* 144.88, 141.75, 133.35, and 130.02 (*C*H), 128.54 (*C*H), 128.06 (*C*H), 126.10 (*C*H), 70.58 (*C*H_2_O), and 35.26, 28.51, 27.26, and 21.82 (*C*H_3_). HRMS calcd for C_17_H_24_NO_3_ [*M* + NH_4_]^+^: *m*/*z* = 322.1477, found 322.1476; error: −0.3 ppm.

4-(4-Iodophenyl)butyl 4-methylbenzenesulfonate (**T2**). 4-(4-Iodophenyl)butan-1-ol (1.20 g, 4.35 mmol), tosyl chloride (1.07 g, 5.61 mmol), and Et_3_N (2.0 mL, 14.3 mmol) were dissolved in DCM (20 mL) and stirred at RT for 24 h. Reaction progress was monitored with reversed phase HPLC (general method B). Silica gel (25 mL) was added and the solvent removed under vacuum. The residue was purified (Combi-Flash, silica gel, hexane/ethyl acetate) to give **T2** as white crystals (1.13 g, yield 61%). Mp: 58–60 °C. ^1^H-NMR (CDCl_3_): *δ* 7.77 (d, ^3^*J*_HH_ = 8.2 Hz, 2H, Ar-*H*), 7.57 (d, ^3^*J*_HH_ = 8.2 Hz, 2H, Ar-*H*), 7.33 (d, ^3^*J*_HH_ = 8.1 Hz, 2H, Ar-*H*), 6.86 (d, ^3^*J*_HH_ = 8.2 Hz, 2H, Ar-*H*), 4.03 (t, ^3^*J*_HH_ = 5.8 Hz, 2H, C*H_2_*O), 2.51 (t, ^3^*J*_HH_ = 5.8 Hz, 2H, C*H_2_*O), 2.45 (s, 3H, C*H_3_*), and 1.63–1.61 (m, 4H, C*H_2_*C*H_2_*). ^13^C{^1^H}-NMR (CDCl_3_): *δ* 144.96, 141.38, and 137.61 (*C*H), 133.32 and 130.67 (*C*H), 130.05 (*C*H), 128.08 (*C*H), 91.15 and 70.42 (*C*H_2_O), and 34.78, 28.45, 27.11, and 21.86 (*C*H_3_). HRMS calcd for C_17_H_23_NO_3_SI [*M* + NH_4_]^+^: *m*/*z* = 448.0443, found 448.0441; error: −0.4 ppm.

4-(4-(Methylthio)phenyl)butyl 4-methylbenzenesulfonate (**T3**). 4-(4-Methylthiophenyl)butan-1-ol (0.51 g, 2.60 mmol), tosyl chloride (0.56 g, 2.94 mmol), and Et_3_N (2.1 mL, 15.0 mmol) were dissolved in DCM (20 mL) and stirred at RT for 24 h. Reaction progress was monitored with reversed phase HPLC (general method B). Silica gel (10 mL) then was added and the solvent removed under vacuum. The residue was purified (Combi-Flash, silica gel, hexane/ethyl acetate) to give **T3** as a colorless oil (0.43 g, yield 47%). ^1^H-NMR (CDCl_3_): *δ* 7.77 (d, ^3^*J*_HH_ = 12 Hz, 2H, Ar-*H*), 7.33 (d, ^3^*J*_HH_ = 8.0 Hz, 2H, Ar-*H*), 7.17 (d, ^3^*J*_HH_ = 8.0 Hz, 2H, Ar-*H*), 7.03 (d, ^3^*J*_HH_ = 12 Hz, 2H, Ar-*H*), 4.03 (t, ^3^*J*_HH_ = 6.0 Hz, 2H, C*H_2_*O), 2.52 (t, ^3^*J*_HH_ = 6.0 Hz, 2H, C*H_2_*), 2.46 (s, 3H, C*H_3_*), 2.44 (s, 3H, C*H_3_*), and 1.64 (m, 4H, C*H_2_*C*H_2_*). ^13^C{^1^H}-NMR (CDCl_3_): *δ* 144.91, 138.88, 135.70, 133.38, 130.04, 129.11, 128.09, 127.34, and 70.54 (*C*H_2_O), 34.73, 28.49, 27.27, and 21.86 (*C*H_3_), and 16.51 (S*C*H_3_). HRMS calcd for C_18_H_26_NO_3_S_2_ [*M* + NH_4_]^+^: *m*/*z* = 368.1354, found 368.1348; error: −1.6 ppm.

4-(4-(Trifluoromethyl)phenyl)butyl 4-methylbenzenesulfonate (**T4**). Use of the method for **T3** in same molar proportions to **5** (1.0 g, 4.58 mmol) gave **T4** as white crystals (1.26 g, yield 74%). Mp: 52–54 °C. ^1^H-NMR (CDCl_3_): *δ* 7.78 (d, ^3^*J*_HH_ = 8.0 Hz, 2H, Ar-*H*), 7.51 (d, ^3^*J*_HH_ = 8.0 Hz, 2H, Ar-*H*), 7.33 (d, ^3^*J*_HH_ = 8.0 Hz, 2H, Ar-*H*), 7.22 (d, ^3^*J*_HH_ = 8.0 Hz, 2H, Ar-*H*), 4.04 (d, ^3^*J*_HH_ = 6.0 Hz, 2H, C*H_2_*), 2.65 (d, ^3^*J*_HH_ = 6.0 Hz, 2H, C*H_2_*), and 1.68 (m, 4H, C*H_2_*C*H*_2_). ^13^C{^1^H}-NMR (CDCl_3_): *δ* 145.87, 144.99, 133.31, 130.05, 128.85, and 128.55 (^2^*J*_CF_ = 32.2 Hz), 128.07 and 125.50 (^3^*J*_CF_ = 4.0 Hz), 124.51 (^1^*J*_CF_ = 272.7 Hz), 70.33 (*C*H_2_O), and 35.11, 28.49, 27.06, and 21.82 (*C*H_3_). HRMS calcd for C_18_H_23_NO_3_SF_3_ [*M* + NH_4_]^+^: *m*/*z* = 390.1351, found 390.1347; error: −1.0 ppm.

4-(4-Methoxyphenyl)butyl 4-methylbenzenesulfonate (**T5**). Use of the method for **T3** in the same molar proportions to 4-(4-methoxyphenyl)butan-1-ol (1.0 g, 5.5 mmol) gave **T5** as a pale-yellow oil (0.39 g, yield 21%). ^1^H-NMR (CDCl_3_): *δ* 7.77 (d, ^3^*J*_HH_ = 8.1 Hz, 2H, Ar-*H*), 7.33 (d, ^3^*J*_HH_ = 8.0 Hz, 2H, Ar-*H*), 7.01 (d, ^3^*J*_HH_ = 8.4 Hz, 2H, Ar-*H*), 7.28 (d, ^3^*J*_HH_ = 8.5 Hz, 2H, Ar-*H*), 4.03 (t, ^3^*J*_HH_ = 6.0 Hz, 2H, C*H_2_*), 3.78 (s, 3H, OC*H_3_*), 2.50 (t, ^3^*J*_HH_ = 6.0 Hz, 2H, C*H_2_*), 2.44 (s, C*H_3_*), and 1.65–1.58 (m, 4H, C*H_2_*C*H_2_*). ^13^C{^1^H}-NMR (CDCl_3_): *δ* 158.03, 144.87, 133.82, 133.38, and 130.01 (*C*H), 129.42 (*C*H), 128.07 (*C*H), 113.96 (*C*H), 70.63 (*C*H_2_O), 55.45 (O*C*H_3_), and 34.35, 28.47, 27.49, and 21.83 (*C*H_3_). HRMS calcd for C_18_H_26_NO_4_S [*M* + NH_4_]^+^: *m*/*z* = 352.1583, found 352.1581; error: −0.6 ppm.

4-(*p*-Tolyl)butyl 4-methylbenzenesulfonate (**T6**). Use of the method for **T3** in the same molar proportions to 4-(*p*-tolyl)butan-1-ol (1.0 g, 6.1 mmol) gave **T6** as a colorless wax (0.46 g, yield 24%). ^1^H-NMR (CDCl_3_): *δ* 7.78 (d, ^3^*J*_HH_ = 8.0 Hz, 2H, Ar-*H*), 7.33 (d, ^3^*J*_HH_ = 8.0 Hz, 2H, Ar-*H*), 7.07 (d, ^3^*J*_HH_ = 8.0 Hz, 2H, Ar-*H*), 6.99 (d, ^3^*J*_HH_ = 8.0 Hz, 2H, Ar-*H*), 4.03 (t, ^3^*J*_HH_ = 6.0 Hz, 2H, C*H_2_*O), 2.52 (t, ^3^*J*_HH_ = 6.0 Hz, 2H, C*H_2_*), 2.44 (s, 3H, C*H_3_*), 2.31 (s, 3H, C*H_3_*), and 1.66–1.61 (m, 4H, C*H_2_*C*H_2_*). ^13^C{^1^H}-NMR (CDCl_3_): *δ* 144.87, 138.67, 135.57, 133.40, and 130.02 (*C*H), 129.24 (*C*H), 128.43 (*C*H), 128.09 (*C*H), 70.63 (*C*H_2_O), 34.83, 28.53, 27.38, and 21.85 (*C*H_3_), and 21.19 (*C*H_3_). HRMS calcd for C_18_H_26_NO_3_S [*M* + NH_4_]^+^: *m*/*z* = 336.1633, found 366.1633; error: 0.0 ppm.

4-(4-Bromophenyl)butyl 4-methylbenzenesulfonate (**T7**). 4-(4-Bromophenyl)butan-1-ol (1.0 g, 4.4 mmol), tosyl chloride (0.99 g, 5.2 mmol) and Et_3_N (2 mL, 14.3 mmol) were dissolved in DCM (20 mL). The reaction mixture was stirred at RT for 5 d. Reaction progress was monitored with HPLC (general method A). Silica gel (60 mL) was then added and the solvent removed under vacuum. The residue was then purified (Combi-Flash, silica gel, hexane/ethyl acetate) to give **T7** as a pale-yellow wax (0.97 g, yield 58%). ^1^H-NMR (CDCl_3_): *δ* 7.77 (d, ^3^*J*_HH_ = 8.0 Hz, 2H, Ar-*H*), 7.36 (d, ^3^*J*_HH_ = 8.0 Hz, 2H, Ar-*H*), 7.33 (d, ^3^*J*_HH_ = 8.0 Hz, 2H, Ar-*H*), 6.97 (d, ^3^*J*_HH_ = 8.0 Hz, 2H, Ar-*H*), 4.02 (t, ^3^*J*_HH_ = 6.0 Hz, 2H, C*H_2_*O), 2.51 (t, ^3^*J*_HH_ = 6.0 Hz, 2H, C*H_2_*), 2.44 (s, 3H, C*H_3_*), and 1.64–1.62 (m, 4H, C*H_2_*C*H_2_*). ^13^C{^1^H}-NMR (CDCl_3_): *δ* 144.93, 140.68, 133.27, and 131.56 (*C*H), 130.28 (*C*H), 130.01 (Ts, *C*H), 128.02 (Ts, *C*H), 119.80 and 70.40 (*C*H_2_O), and 34.63, 28.40, 27.10, and 21.81 (*C*H_3_). HRMS calcd for C_17_H_23_NO_3_SBr [*M* + NH_4_]^+^: *m*/*z* = 400.0582, found 400.0588; error: 1.5 ppm.

4-(4-Fluorophenyl)butyl 4-methylbenzenesulfonate (**T8**). 4-(4-Fluorophenyl)butan-1-ol (1.0 g, 4.4 mmol), tosyl chloride (1.22 g, 6.4 mmol), and Et_3_N (2.5 mL, *d* = 0.726 g/mL, 17.9 mmol) were dissolved in DCM (20 mL). The reaction mixture was stirred at RT for 5 d. Silica gel (20 mL) was then added and the solvent was removed under vacuum. The residue was then purified (Combi-Flash, silica gel, hexane/ethyl acetate) to give **T8** as a colorless oil (0.99 g, yield 52%). ^1^H-NMR (CDCl_3_): *δ* 7.78 (d, ^3^*J*_HH_ = 12.0 Hz, 2H, Ar-H), 7.33 (d, ^3^*J*_HH_ = 8.0 Hz, 2H, Ar-*H*), 7.06 (dd, ^3^*J*_HH_ = 8.0 Hz, ^3^*J*_HF_ = 4.0 Hz, 2H, Ar-*H*), 6.94 (t, ^3^*J*_HH_ = 8.0 Hz, 2H, Ar-*H*), 4.03 (t, ^3^*J*_HH_ = 6.0 Hz, 2H, C*H_2_*), 2.54 (t, ^3^*J*_HH_ = 6.0 Hz, 2H, C*H_2_*), 2.44 (s, C*H_3_*), and 1.64 (m, 4H, C*H_2_*C*H_2_*). ^13^C{^1^H}-NMR (CDCl_3_): *δ* 161.49 (d, ^1^*J*_CF_ = 243.5 Hz, Fph, *C*-F), 144.92 and 137.6 (d, ^4^*J*_CF_ = 3.0 Hz, Fph, *C*HCHCHCF), 133.35 and 130.03 (*C*H), 129.85 (d, ^3^*J*_CF_ = 8.0 Hz, Fph, *C*HCHCF), 128.07 (*C*H), 115.29 (d, ^2^*J*_CF_ = 21.1 Hz, Fph, *C*HCF), 70.49 (*C*H_2_O), and 34.47, 28.46, 27.42, and 21.83 (*C*H_3_). HRMS calcd for C_17_H_23_NO_3_SF [*M* + NH_4_]^+^: *m*/*z* = 340.1383, found 340.1378; error: −1.5 ppm.

4-(Naphthalen-2-yl)butyl 4-methylbenzenesulfonate (**T9**). Use of the method for **T8** in the same molar proportions to 4-(naphthalen-2-yl)butan-1-ol (1.0 g, 5.0 mmol) gave **T9** as tan crystals (0.39 g, yield 22%). Mp: 34–36 °C). ^1^H-NMR (CDCl_3_): *δ* 7.81–7.75 (m, 5H, Ar-*H*), 7.54 (s, 1H, Ar-*H*), 7.46 (t, ^3^*J*_HH_ = 6.5 Hz, 1H, Ar-*H*), 7.42 (t, ^3^*J*_HH_ = 6.5 Hz, 1H, Ar-*H*), 7.30 (d, ^3^*J*_HH_ = 8.0 Hz, 2H, Ts, Ts-*H*), 7.26 (d, ^3^*J*_HH_ = 8.3 Hz, 1H, Np-*H*), 4.06 (t, ^3^*J*_HH_ = 5.8 Hz, 2H, C*H_2_*), 2.74 (t, ^3^*J*_HH_ = 5.8 Hz, 2H, C*H_2_*), 2.42 (s, C*H_3_*), and 1.75–1.70 (m, 4H, C*H_2_*C*H_2_*). ^13^C{^1^H}-NMR (CDCl_3_): *δ* 144.87, 139.24, 133.75, 133.34, 132.22, and 130.01 (Ts, *C*H), 128.14 (*C*H), 128.06 (Ts, *C*H), 127.80 (*C*H), 127.58 (*C*H), 127.32 (*C*H), 126.63 (*C*H), 126.16 (*C*H), 125.42 (*C*H), 70.59 (*C*H_2_O), and 35.40, 28.52, 27.13, and 21.80 (*C*H_3_). HRMS calcd for C_21_H_26_NO_3_S [*M* + NH_4_]^+^: *m*/*z* = 372.1633, found 372.1638; error: 1.3 ppm.

4-(Pyridin-2-yl)butyl 4-methylbenzenesulfonate (**T10**). 4-(Pyridin-2-yl)butan-1-ol (5.0 g, 33.1 mmol), tosyl chloride (7.66 g, 40.2 mmol), and Et_3_N (12 mL, 86.1 mmol) were dissolved in ethyl acetate (100 mL). The reaction mixture was stirred at RT for 24 h. Reaction progress was monitored with HPLC. Silica gel (60 mL) was then added and the solvent removed under vacuum. The residue was purified (Combi-Flash, silica gel, hexane/ethyl acetate) to give **T10** as a thick colorless oil (2.09 g, yield 21%). ^1^H-NMR (CDCl_3_): *δ* 8.42 (d, ^3^*J*_HH_ = 4.0 Hz, 1H, py-*H*), 8.36 (s, 1H, py-*H*), 7.76 (d, ^3^*J*_HH_ = 12.0 Hz, 2H, Ts-*H*), 7.42 (d, ^3^*J*_HH_ = 8.0 Hz, 1H, py-*H*), 7.32 (d, ^3^*J*_HH_ = 12.0 Hz, 2H, Ts-*H*), 7.18 (dd, ^3^*J*_HH_ = 8.0 Hz, ^3^*J*_HH_ = 4.0 Hz, 1H, py-*H*), 4.02 (t, ^3^*J*_HH_ = 6.0 Hz, 2H, C*H_2_*), 2.55 (t, ^3^*J*_HH_ = 6.0 Hz, 2H, C*H_2_*), 2.42 (s, 3H, C*H_3_*), and 1.66–1.64 (m, 4H, C*H_2_*C*H_2_*). ^13^C{^1^H}-NMR (CDCl_3_): *δ* 149.99 (*C*H), 147.70 (*C*H), 144.96, 136.88, and 135.85 (*C*H), 133.18 and 130.00 (Ts, *C*H), 127.99 (Ts, *C*H), 123.46 and 70.22 (*C*H_2_O), and 32.34, 28.40, 27.00, and 21.77 (*C*H_3_). HRMS calcd for C_7_H_7_O_3_S^-^: *m*/*z* = 171.0116, found 171.0119. Error (ppm): 1.8. HRMS calcd for C_9_H_12_N^+^: *m*/*z* = 134.0970, found 134.0967; error: −2.2 ppm.

4-(Pyridin-2-yl)but-3-yn-1-yl 4-methylbenzenesulfonate (**T11**). 4-(Pyridin-2-yl)but-3-yn-1-ol (1.99 g, 13.5 mmol), tosyl chloride (3.11 g, 16.3 mmol), and Et_3_N (6.0 mL, 43.0 mmol) were dissolved in DCM (100 mL). The reaction mixture was stirred at RT for 24 h. Reaction progress was monitored with HPLC (general method B). Silica gel (60 mL) was then added and the solvent removed under vacuum. The residue was purified (Combi-Flash, silica gel, hexane/ethyl acetate) to give **T11** as a clear wax (0.88 g, yield 22%). ^1^H-NMR (CDCl_3_): *δ* 8.54 (d, ^3^*J*_HH_ = 8.0 Hz, 1H, py-*H*), 7.82 (d, ^3^*J*_HH_ = 8.0 Hz, 2H, Ts-*H*), 7.63 (dt, ^3^*J*_HH_ = 8.0 Hz, ^4^*J*_HH_ = 2.0 Hz, 1H, py-*H*), 7.34 (d, ^3^*J*_HH_ = 8.0 Hz, 1H, py-*H*), 7.32 (d, ^3^*J*_HH_ = 8.0 Hz, 2H, Ts-*H*), 7.21 (dd, ^3^*J*_HH_ = 8.0 Hz, ^3^*J*_HH_ = 4.0 Hz, 1H, py-*H*), 4.20 (t, ^3^*J*_HH_ = 6.0 Hz, 2H, C*H_2_*), 2.82 (t, ^3^*J*_HH_ = 6.0 Hz, 2H, C*H_2_*), and 2.43 (s, 3H, C*H_3_*). ^13^C{^1^H}-NMR (CDCl_3_): *δ* 150.13 (py, *C*H), 145.21, 143.20, and 136.33 (py, *C*H), 132.98 and 130.13 (Ts, *C*H), 128.22 (Ts, *C*H), 127.20 (py, *C*H), 123.08 (py, *C*H), 84.36 (*C*2), 82.48 (*C*2), 67.49 (*C*H_2_O), 21.86 (*C*H_3_), and 20.50. HRMS calcd for C_16_H_16_NO_3_S [*M* + H]^+^: *m*/*z* = 302.0851, found 302.0854; error: 1.0 ppm.

4-(6-Fluoropyridin-2-yl)but-3-yn-1-yl 4-methylbenzenesulfonate (**T12**). 4-(6-Fluoropyridin-2-yl)but-3-yn-1-ol (**3**, 2.65 g, 16.0 mmol), tosyl chloride (3.18 g, 16.7 mmol), and Et_3_N (10 mL, *d* = 0.726 g/mL, 71.7 mmol) were dissolved in DCM (40 mL). The reaction mixture was stirred at RT for 24 h. Reaction progress was monitored with HPLC (general method B). Silica gel (60 mL) was added and the solvent removed under vacuum. The reaction mixture was then purified (Combi-Flash, silica gel, hexane/ethyl acetate) to give **T12** as a clear wax (0.68 g, yield 13%). ^1^H-NMR (CDCl_3_): *δ* 7.82 (d, ^3^*J*_HH_ = 8.1 Hz, 2H, Ar-*H*), 7.73 (pseudo-q, ^3^*J*_HH_ = ^3^*J*_HF_ = 7.9 Hz, 1H, Ar-*H*), 7.34 (d, ^3^*J*_HH_ = 8.0 Hz, 2H, Ar-*H*), 7.23 (dd, ^3^*J*_HH_ = 7.4 Hz, ^5^*J*_HF_ = 1.9 Hz, 1H, Ar-*H*), 6.88 (dd, ^3^*J*_HH_ = 8.3 Hz, ^3^*J*_HF_ = 2.7 Hz, 1H, Ar-*H*), 4.19 (t, ^3^*J*_HH_ = 6.0 Hz, 2H, C*H_2_*), 2.81 (t, ^3^*J*_HH_ = 6.0 Hz, 2H, C*H_2_*), and 2.44 (s, C*H_3_*). ^13^C{^1^H}-NMR (CDCl_3_): *δ* 163.1 (d, ^1^*J*_CF_ = 250 Hz), 145.28 and 141.38 (d, *J*_CF_ = 8.1 Hz), 141.25 (d, *J*_CF_ = 37.5 Hz), 132.93 and 130.18 (Ts, *C*H), 128.22 (Ts, *C*H), 124.69 (d, *J*_CF_ = 4.0 Hz), 109.66 (d, ^1^*J*_CF_ = 36.6 Hz), 85.87 (*C*_2_), 81.23 (*C*_2_), 67.28 (*C*H_2_O), and 21.86 and 20.50 (*C*H_3_). HRMS calcd for C_16_H_15_NO_3_FS [*M* + H]^+^: *m*/*z* = 320.0757, found 320.0763; error: 1.9 ppm.

2-(Benzofuran-2-yl)ethyl 4-methylbenzenesulfonate (**T13**). Use of the method for **T12** in the same molar proportions to **6** (910 mg, 5.61 mmol) gave **T13** as white crystals (799 mg, yield 45%). Mp: 76–77 °C. ^1^H-NMR (CDCl_3_): *δ* 7.67 (d, ^3^*J*_HH_ = 8.0 Hz, 2H, Ts, Ar-*H*), 7.46 (d, ^3^*J*_HH_ = 7.3 Hz, 1H, BFu, Ar-*H*), 7.30 (d, ^3^*J*_HH_ = 7.7 Hz, 1H, BFu, Ar-*H*,), 7.24–7.18 (m, 2H, BFu Ar-*H*), 7.17 (d, ^3^*J*_HH_ = 8.0 Hz, 2H, Ts, Ar-*H*), 6.42 (s, 1H, BFu, Ar-*H*), 4.36 (t, ^3^*J*_HH_ = 6.3 Hz, 2H, C*H_2_*), 3.12 (t, ^3^*J*_HH_ = 6.3 Hz, 2H, C*H_2_*), and 2.37 (s, 3H, C*H_3_*). ^13^C{^1^H}-NMR (CDCl_3_): *δ* 154.90, 153.29, 144.98, 132.83, and 129.92 (Ts, *C*H), 128.64 and 127.99 (Ts, *C*H), 123.92 (BFu, *C*H), 122.87 (BFu, *C*H), 120.77 (BFu, *C*H), 111.02 (BFu, *C*H), 104.54 (BFu, *C*H), 67.55 (*C*H_2_O), 28.75 (*C*H_2_), and 21.81 (*C*H_3_). HRMS calcd for C_17_H_20_NO_4_S [*M* + NH_4_]^+^: *m*/*z* = 334.1113, found 334.1114; error: 0.3 ppm.

2-(5-Bromobenzofuran-2-yl)ethyl 4-methylbenzenesulfonate (**T14**). Use of the method for **T12** in the same molar proportions to **7** (790 mg, 3.28 mmol) gave **T14** as white crystals (699 mg, yield 54%). Mp: 88–90 °C. ^1^H-NMR (CDCl_3_): *δ* 7.67 (d, ^3^*J*_HH_ = 8.0 Hz, 2H, Ts, Ar-*H*), 7.58 (s, 1H, BFu, Ar-*H*), 7.31 (d, ^3^*J*_HH_ = 8.7 Hz, 1H, BFu, Ar-*H*), 7.18 (d, ^3^*J*_HH_ = 8.0 Hz, ^3^*J*_HH_ = 8.7 Hz, 3H, BFu-Ts, Ar-*H*), 6.36 (s, 1H, BFu, Ar-*H*), 4.36 (t, ^3^*J*_HH_ = 6.3 Hz, 2H, CH_2_), 3.11 (t, ^3^*J*_HH_ = 6.3 Hz, 2H, C*H_2_*), and 2.38 (s, 3H, C*H_3_*). ^13^C{^1^H}-NMR (CDCl_3_): *δ* 154.91, 153.65, 145.06, 132.84, 130.64, and 129.93 (Ts, *C*H), 127.97 (Ts, *C*H), 126.82 (BFu, *C*H), 123.44 (BFu, *C*H), 115.93 and 112.48 (BFu, *C*H), 104.10 (BFu, *C*H), 67.32 (*C*H_2_O), 28.76 (*C*H_2_), and 21.83 (*C*H_3_). HRMS calcd for C_17_H_19_NO_4_SBr [*M* + NH_4_]^+^: *m*/*z* = 412.0218, found 412.0225; error: 1.7 ppm.

2-(5-Fluorobenzofuran-2-yl)ethyl 4-methylbenzenesulfonate (**T15**). Use of the method for **T12** in the same molar proportions to **8** (610 mg, 3.39 mmol) gave **T15** as white crystals (815 mg, yield 72%). Mp: 76–78 °C. ^1^H-NMR (CDCl_3_): *δ* 7.68 (d, ^3^*J*_HH_ = 8.1 Hz, 2H, Ts, Ar-*H*), 7.23 (dd, ^3^*J*_HF_ = 4.1 Hz, ^3^*J*_HH_ = 8.9 Hz, 1H, BFu), 7.20 (d, ^3^*J*_HH_ = 8.1 Hz, 2H, Ts, Ar-*H*), 7.11 (dd, ^4^*J*_HF_ = 2.5 Hz, ^3^*J*_HH_ = 9.0 Hz, 1H, BFu, Ar-*H*), 6.93 (td, ^3^*J*_HF_ = ^4^*J*_HH_ = 8.6 Hz, ^4^*J*_HH_ = 2.5 Hz, 1H, BFu, Ar-*H*), 6.40 (s, 1H), 4.36 (t, ^3^*J*_HH_ = 6.4 Hz, 2H, C*H_2_*), 3.11 (t, ^3^*J*_HH_ = 6.4 Hz, 2H, C*H_2_*), and 2.38 (s, 3H, C*H_3_*). ^13^C{^1^H}-NMR (CDCl_3_): *δ* 154.35 (d, ^1^*J*_CF_ = 236.4 Hz), 155.32, 151.13, 145.04, 132.87, and 129.94 (Ts, *C*H), 129.45 (d, *J*_CF_ = 10.8 Hz, *C*H), 128.01 (Ts, *C*H), 111.64 (d, *J*_CF_ = 2.7 Hz, *C*H), 111.46 (d, *J*_CF_ = 13.8 Hz, *C*H), 106.35 (d, *J*_CF_ = 25.0 Hz, *C*H), 104.81 (d, *J*_CF_ = 4.0 Hz, *C*H), 67.37 (*C*H_2_O), 28.84 (*C*H_2_), and 21.82 (*C*H_3_). HRMS calcd for C_17_H_19_NO_4_FS [*M* + NH_4_]^+^: *m*/*z* = 352.1019, found 352.1017; error: −0.6 ppm.

2-(5-Trifluoromethylbenzofuran-2-yl)ethyl 4-methylbenzenesulfonate (**T16**). Use of the method for **T12** in the same molar proportions to **9** (1.07 g, 4.65 mmol) gave **T16** as white crystals (697 mg, yield 39%). Mp: 94–95 °C. ^1^H-NMR (CD_3_OD): *δ* 7.74 (s, 1H, Ar-*H*), 7.53 (dd, ^3^*J*_HH_ = 6.6 Hz, ^4^*J*_HH_ = 1.6 Hz, 2H, Ar-*H*), 7.44 (AB, ^3^*J*_HH_ = 8.7 Hz, ^4^*J*_HH_ = 1.7 Hz, 1H, Ar-*H*), 7.40 (AB, ^4^*J*_HH_ = 8.7 Hz, 1H, Ar-*H*), 7.09 (d, ^3^*J*_HH_ = 8.0 Hz, 2H, Ar-*H*), 6.52 (d, ^4^*J*_HH_ = 0.7 Hz, 1H, Ar-*H*), 4.32 (t, ^3^*J*_HH_ = 5.7 Hz, 2H, C*H_2_*), 3.07 (t, ^3^*J*_HH_ = 5.7 Hz, 2H, C*H_2_*), and 2.24 (s, 3H, Ar-C*H_3_*). ^13^C{^1^H}-NMR (CD_3_OD): *δ* 157.93, 157.68, 146.47, 134.19, and 130.96 (Ts, *C*H), 130.44 and 128.95 (Ts, *C*H), 126.37 (q, ^1^*J*_CF_ = 271.0 Hz, 1C, *C*F), 126.51 (q, ^2^*J*_CF_ = 32.0 Hz, 1C), 122.06 (q, ^3^*J*_CF_ = 3.6 Hz, 1C, BFu, *C*H), 119.38 (q, ^3^*J*_CF_ = 4.3 Hz, 1C, BFu *C*H), 112.58 (BFu, *C*H), 105.70 (BFu, *C*H), 69.02, (*C*H_2_O), 29.40 (*C*H_2_), and 21.66 (*C*H_3_). HRMS calcd for C_18_H_19_NO_4_F_3_S [*M* + NH_4_]^+^: *m*/*z* = 402.0987, found 402.0992; error: 1.2 ppm.

4-(Benzofuran-2-yl)butyl 4-methylbenzenesulfonate (**T17**). Use of the method for **T12** in the same molar proportions to **10** (710 mg, 3.73 mmol) gave **T17** as a clear wax (977 mg, yield 76%). ^1^H-NMR (CDCl_3_): *δ* 7.78 (d, ^3^*J*_HH_ = 8.0 Hz, 2H, Ts, Ar-*H*), 7.47 (d, ^3^*J*_HH_ = 7.4 Hz, 1H, BFu, Ar-*H*), 7.39 (d, ^3^*J*_HH_ = 7.6 Hz, 1H, BFu, Ar-*H*), 7.32 (d, ^3^*J*_HH_ = 8.0 Hz, 2H, Ts, Ar-*H*), 7.21–7.17 (m, 2H, BFu, Ar-*H*), 6.34 (s, 1H, BFu, Ar-*H*), 4.07 (t, ^3^*J*_HH_ = 5.7 Hz, 2H, C*H_2_*), 2.72 (t, ^3^*J*_HH_ = 5.7 Hz, 2H, C*H_2_*), 2.43 (s, 3H, C*H_3_*), and 1.76 (m, 4H, C*H_2_*C*H_2_*). ^13^C{^1^H}-NMR (CDCl_3_): *δ* 158.49, 154.83, 144.95, 132.83, and 130.05 (Ts, *C*H), 128.98 and 128.08 (Ts, *C*H), 123.48 (BFu, *C*H), 122.69 (BFu, *C*H), 120.47 (BFu, *C*H), 111.93 (BFu, *C*H), 102.53 (BFu, *C*H), 70.29 (*C*H_2_O), 28.41 (*C*H_2_), 27.84 (*C*H_2_), 23.82 (*C*H_2_), and 21.82 (*C*H_3_). HRMS calcd for C_19_H_24_NO_4_S [*M* + NH_4_]^+^: *m*/*z* = 362.1426, found 362.1419; error: −1.9 ppm.

4-(5-(Trifluoromethyl)benzofuran-2-yl)butyl 4-methylbenzenesulfonate (**T18**). Use of the method for **T12** in the same molar proportions to **11** (425 mg, 1.65 mmol) gave **T18** as a clear wax (380 mg, yield 56%). ^1^H-NMR (CDCl_3_): *δ* 7.78 (d, ^3^*J*_HH_ = 8.0 Hz, 2H, Ts-*H*), 7.76 (s, 1H, Ar-*H*), 7.49–7.45 (AB, ^3^*J*_HH_ = 8.0 Hz, 2H, Ar-*H*), 7.32 (d, ^3^*J*_HH_ = 8.0 Hz, 2H, Ts-*H*), 6.42 (s, 1H, Ar-*H*), 4.07 (t, ^3^*J*_HH_ = 6.0 Hz, 2H, C*H_2_*), 2.77 (t, ^3^*J*_HH_ = 6.0 Hz, 2H, C*H_2_*), 2.43 (s, 3H, C*H_3_*), and 1.83–1.61 (m, 4H, C*H_2_*C*H_2_*). ^13^C{^1^H}-NMR (CDCl_3_): *δ* 160.62, 156.22, and 145.02 (Ts), 133.26 (Ts), 130.06 (Ts, *C*H), 129.07 and 128.08 (Ts, *C*H), 125.44 (q, ^2^*J*_CF_ = 32 Hz), 124.93 (q, ^1^*J*_CF_ = 271 Hz, *C*F_3_), 120.72 (q, ^2^*J*_CF_ = 3.0 Hz, *C*HCF), 110.13 (q, ^2^*J*_CF_ = 4.0 Hz, *C*HCF), 70.14 (*C*H_2_O), and 28.43, 27.87, 23.73, and 21.82. HRMS calcd for C_20_H_23_NO_4_F_3_S [*M* + NH_4_]^+^: *m*/*z* = 430.1300, found 430.1305; error: 1.2 ppm.

#### 3.3.2. Syntheses of Racemic GluN2B Ligands L1–L20

7-Methoxy-3-(4-(phenyl)butyl)-2,3,4,5-tetrahydro-1*H*-benzo[*d*]azepin-1-ol (**L1**). 7-Methoxy-2,3,4,5-tetrahydro-1*H*-benzo[*d*]azepin-1-ol (190 mg, 0.98 mmol), **T1** (390 mg, 1.28 mmol), and K_2_CO_3_ (610 mg, 4.4 mmol) were suspended in acetonitrile (10 mL) and refluxed for 6 d. Reaction progress was monitored with HPLC (general method A). The reaction mixture was cooled to RT, passed through a 0.2 μm syringe filter, and then purified with HPLC (general method C). The solvent was then removed under vacuum. The residue was dissolved in acetonitrile, passed through a 0.2 μm syringe filter, and dried with Centrifan to give **L1** as white crystals (0.23 g, yield 72%). Mp: 78–79 °C. ^1^H-NMR (CDCl_3_): *δ* 7.28 (t, ^3^*J*_HH_ = 8.0 Hz, 2H, *m*-Ar-*H*), 7.18 (t, ^3^*J*_HH_ = 8.0 Hz, 1H, *p*-Ar-*H*), 7.18 (d, ^3^*J*_HH_ = 8.0 Hz, 2H, *o*-Ar-*H*), 7.10 (d, ^3^*J*_HH_ = 8.0 Hz, 1H, Ar-*H*), 6.64 (dd, ^3^*J*_HH_ = 8.0 Hz, ^4^*J*_HH_ = 4.0 Hz, 1H, Ar-*H*), 6.63 (brs, 1H, Ar-*H*), 4.59 (d, ^3^*J*_HH_ = 4.0 Hz, 1H, C*H*-OH), 3.76 (s, 3H, OC*H_3_*), 3.26 (vt, ^2^*J*_HH_ = 12 Hz, 1H, C*H_2_*), 3.18–3.14 (m, 1H, C*H_2_*), 3.02–2.97 (m, 1H, C*H_2_*), 2.68–2.59 (m, 5H, C*H_2_*), 2.53 (d, ^2^*J*_HH_ = 12 Hz, 1H, C*H_2_*), 2.43 (vt, ^2^*J*_HH_ = 12 Hz, 1H, C*H_2_*), 1.69–1.62 (m, 2H, C*H_2_*), and 1.61–1.54 (m, 2H, C*H_2_*). ^13^C{^1^H}-NMR (CDCl_3_): *δ* 159.09, 142.43, 141.25, 135.57, 129.86, and 128.57 (*C*H), 128.52 (*C*H), 125.97 (*C*H), 116.74 (*C*H), 110.39 (*C*H), 72.42 (*C*HO), 60.88 (*C*N), 59.76 (*C*N), 56.19 (*C*N), 55.38 (O*C*H_3_), and 36.52, 35.92, 29.28, and 26.66. HRMS calcd for C_21_H_28_NO_2_ [*M* + H]^+^: *m*/*z* = 326.2120, found 326.2126; error: 1.8 ppm. HPLC (general method A): *t*_R_ = 5.14 min, purity 99.62%.

7-Methoxy-3-(4-(4-iodophenyl)butyl)-2,3,4,5-tetrahydro-1*H*-benzo[*d*]azepin-1-ol (**L2**). 7-Methoxy-2,3,4,5-tetrahydro-1*H*-benzo[*d*]azepin-1-ol (420 mg, 2.17 mmol), **T2** (1.13 g, 2.63 mmol), and Na_2_HPO_4_ (1.30 g, 9.2 mmol) were suspended in acetonitrile (8 mL) and heated at 75 °C for 5 d. Reaction progress was monitored with HPLC (general method A). The reaction mixture was then cooled to RT, passed through a 0.2 μm syringe filter, and purified with HPLC (general method C). The solvent was then removed under vacuum. The residue was dissolved in ethanol, passed through a 0.2 µm syringe filter, and dried with Centrifan to give **L2** as white crystals (0.714 g, yield 73%). Mp: 100–101 °C. ^1^H-NMR (CDCl_3_): *δ* 7.60 (d, ^3^*J*_HH_ = 8.0 Hz, 2H, Ar-*H*), 7.16 (d, ^3^*J*_HH_ = 8.0 Hz, 1H, Ar-*H*), 6.92 (d, ^3^*J*_HH_ = 8.0 Hz, 2H, Ar-*H*), 6.69 (dd, ^3^*J*_HH_ = 8.0 Hz, ^4^*J*_HH_ = 4.0 Hz, 1H, Ar-*H*), 6.65 (d, ^4^*J*_HH_ = 4.0 Hz, 1H, Ar-*H*), 4.76 (brs, 1H, C*H*OH), 3.78 (s, 3H, OC*H_3_*), 3.39–3.32 (m, 2H, C*H_2_*), 3.16–3.12 (m, 1H, C*H_2_*), 2.75 (brs, 4H, C*H_2_*), 2.60–2.58 (m, 3H, C*H_2_*), and 1.63 (m, 4H, C*H_2_*). ^13^C{^1^H}-NMR (CDCl_3_): *δ* 159.34, 141.60, 140.31, and 137.64 (*C*H), 134.50 and 130.67 (*C*H), 129.80 and 116.62 (*C*H), 110.89 (*C*H), 91.16 (*C*_Ar_-I), 71.47 (*C*HO), 60.33 (*C*N), 59.45 (*C*N), 55.79 (*C*N), 55.46 (O*C*H_3_), and 42.47, 35.20, 28.81, 25.46, and 11.26. HRMS calcd for C_21_H_27_INO_2_ [*M* + H]^+^: *m*/*z* = 452.1086, found 452.1092; error: 1.3 ppm. HPLC (general method A): *t*_R_ = 7.31 min, purity 100%.

7-Methoxy-3-(4-(4-(methylthio)phenyl)butyl)-2,3,4,5-tetrahydro-1*H*-benzo[*d*]azepin-1-ol (**L3**). 7-Methoxy-2,3,4,5-tetrahydro-1*H*-benzo[*d*]azepin-1-ol (200 mg, 1.03 mmol), **T3** (450 mg, 1.28 mmol), and Na_2_HPO_4_ (620 mg, 4.4 mmol) were suspended in acetonitrile (2 mL) and heated at 75 °C for 2 d. Reaction progress was monitored with HPLC (general method A). The reaction mixture was cooled to RT, passed through a 0.2 μm syringe filter, and then purified with HPLC (general method C). The solvent was removed under vacuum. The residue was dissolved in ethanol and Na_2_CO_3_ (1.0 g, 9.4 mmol) was added. The mixture was sonicated for 5 min and then stirred at RT for 1 min before finally being passed through a 0.2 µm syringe filter and dried with a Centrifan to give **L3** as white crystals (0.288 g, yield 75%). Mp: 89–91 °C. ^1^H-NMR (CDCl_3_): *δ* 7.18 (AB, ^3^*J*_HH_ = 8.0 Hz, 2H, Ar-*H*), 7.09 (AB, ^3^*J*_HH_ = 8.0 Hz, 1H, Ar-*H*), 7.08 (d, ^3^*J*_HH_ = 8.0 Hz, 1H, Ar-*H*), 6.63 (dd, ^3^*J*_HH_ = 8.0 Hz, ^4^*J*_HH_ = 4.0 Hz, 1H, Ar-*H*), 6.62 (brs, 1H, Ar-*H*), 4.56 (d, ^3^*J*_HH_ = 4.0 Hz, 1H, C*H*OH), 3.75 (s, 3H, OC*H_3_*), 3.25 (vt, ^2^*J*_HH_ = 12 Hz, 1H, C*H_2_*), 3.17–3.12 (m, 1H, C*H_2_*), 3.00–2.95 (m, 1H, C*H_2_*), 2.66–2.60 (m, 1H, C*H_2_*), 2.58 (t, ^3^*J*_HH_ = 8.0 Hz, 4H, C*H_2_*), 2.50 (d, ^2^*J*_HH_ = 12 Hz, 1H, C*H_2_*), 2.45 (s, 3H, SC*H_3_*), 2.39 (vt, ^2^*J*_HH_ = 12 Hz, 1H, C*H_2_*), 1.62 (quint, ^3^*J*_HH_ = 8.0 Hz, 2H, C*H_2_*), and 1.54 (quint, ^3^*J*_HH_ = 8.0 Hz, 2H, C*H_2_*). ^13^C{^1^H}-NMR (CDCl_3_): *δ* 159.14, 141.36, 139.64, 135.69, 135.45, 129.94, and 129.15 (*C*H), 127.39 (*C*H), 116.82 (*C*H), 110.40 (*C*H), 72.60 (*C*HO), 60.97 (*C*N), 59.79 (*C*N), 56.31 (*C*N), 55.42 (O*C*H_3_), and 37.04, 35.40, 29.28, 26.77, and 16.57 (S*C*H_3_). HRMS calcd for C_22_H_30_NO_2_S [*M* + H]^+^: *m*/z = 372.1997, found 372.1999; error: 0.51 ppm. HPLC (general method A): *t*_R_ = 7.31 min, purity 100%.

7-Methoxy-3-(4-(4-trifluoromethylphenyl)butyl)-2,3,4,5-tetrahydro-1*H*-benzo[*d*]azepin-1-ol (**L4**). Use of the method for **L3** in the same molar proportions to **T4** (240 mg, 0.64 mmol) gave **L4** as white crystals. (0.232 g, yield 71%). Mp: 79–81 °C. ^1^H-NMR (CDCl_3_): *δ* 7.52 (d, ^3^*J*_HH_ = 8.0 Hz, 2H, Ar-*H*), 7.26 (d, ^3^*J*_HH_ = 8.0 Hz, 2H, Ar-*H*), 7.14 (d, ^3^*J*_HH_ = 8.0 Hz, 1H, Ar-*H*), 6.67 (dd, ^3^*J*_HH_ = 8.0 Hz, ^4^*J*_HH_ = 4.0 Hz, 1H, Ar-*H*), 6.63 (d, ^4^*J*_HH_ = 4.0 Hz, 1H, Ar-*H*), 4.74 (brs, 1H, C*H*OH), 3.76 (s, 3H, OC*H_3_*), 3.35–3.27 (m, 2H, C*H_2_*), 3.17–3.08 (m, 1H, C*H_2_*), 2.73–2.49 (m, 7H, C*H_2_*), and 1.65 (brs, 4H, C*H_2_*). ^13^C{^1^H}-NMR (CD_3_OD): *δ* 160.26, 148.55, 141.78, 137.22, and 130.23 (2C, *C*H), 129.29 (q, ^2^*J*_CF_ = 31.9 Hz, 1C, *C*CF), 127.67 *(C*H), 126.34 (q,^3^*J*_CF_ = 3.9 Hz, 2C, *C*HCHCF), 126.08 (q, ^1^*J*_CF_ = 271.0 Hz, 1C, *C*F), 116.57 (*C*H), 111.72 (*C*H), 72.37 (O*C*H), 63.71 (*C*N), 60.11 (*C*N), 56.53 (*C*N), 55.75 (O*C*H_3_), 36.58 (*C*H_2_), 30.31 (*C*H_2_), and 27.24 (*C*H_2_). HRMS calcd for C_22_H_27_F_3_NO_2_ [*M* + H]^+^: *m*/*z* = 394.1994, found 394.1998; error: 1.0 ppm. HPLC (general method A): *t*_R_ = 7.21 min, purity 100%.

7-Methoxy-3-(4-(4-methoxyphenyl)butyl)-2,3,4,5-tetrahydro-1*H*-benzo[*d*]azepin-1-ol (**L5**). 7-Methoxy-2,3,4,5-tetrahydro-1*H*-benzo[*d*]azepin-1-ol (100 mg, 0.52 mmol), **T5** (270 mg, 0.81 mmol), and Na_2_HPO_4_ (430 mg, 3.0 mmol) were suspended in acetonitrile (2 mL) and heated at 90 °C for 2 d. Reaction progress was monitored with HPLC (general method A). The reaction mixture was then cooled to RT, passed through a 0.2 μm syringe filter, and purified with HPLC (general method C). The solvent was then removed under vacuum. The residue was then dissolved in acetonitrile, passed through a 0.2 μm syringe filter, and dried with Centrifan to give **L5** as white crystals (0.21 g, yield 87%). Mp: 86–89 °C. ^1^H-NMR (CDCl_3_): *δ* 7.18 (d, ^3^*J*_HH_ = 8.0 Hz, 1H, Ar-*H*), 7.06 (^3^*J*_HH_ = 8 Hz, 2H, Ar-*H*), 6.81 (^3^*J*_HH_ = 8.0 Hz, 2H, Ar-*H*), 6.69 (dd, ^3^*J*_HH_ = 8.0 Hz, ^4^*J*_HH_ = 4.0 Hz, 1H, Ar-*H*), 6.62 (d, ^4^*J*_HH_ = 4.0 Hz, 1H, Ar-*H*), 4.83 (d, ^3^*J*_HH_ = 4.0 Hz, 1H, C*H*OH), 3.77 (s, 3H, OC*H_3_*), 3.76 (s, 3H, OC*H_3_*), 3.37 (vt, ^2^*J*_HH_ = 12 Hz, 1H, C*H_2_*), 3.20–3.29 (m, 1H, C*H_2_*), 3.15 (m, 1H, C*H_2_*), 2.86–2.76 (m, 5H, C*H_2_*), 2.57 (vt, ^2^*J*_HH_ = 12 Hz, 2H, C*H_2_*), and 1.61 (m, 4H, C*H_2_*). ^13^C{^1^H}-NMR (CDCl_3_): *δ* 159.31, 158.02, 139.94, 134.24, 133.88, 129.56, and 129.40 (2C, *C*H), 116.43 (*C*H), 114.00 (2C, *C*H), 111.01 (*C*H), 70.85 (*C*HO), 60.08 (*C*N), 59.39 (*C*N), 55.48 (*C*N), 55.41 (O*C*H_3_), and 34.65, 34.33, 29.09, 24.92, and 21.20. HRMS calcd for C_22_H_30_NO_3_ [*M* + H]^+^: *m*/*z* = 356.2226, found 356.2223; error: −0.8 ppm. HPLC (general method A): *t*_R_ = 5.05 min, purity 99.52%.

7-Methoxy-3-(4-(4-methylphenyl)butyl)-2,3,4,5-tetrahydro-1*H*-benzo[*d*]azepin-1-ol (**L6**). Use of the method for **L5** with 7-methoxy-2,3,4,5-tetrahydro-1*H*-benzo[*d*]azepin-1-ol (130 mg, 0.67 mmol), **T6** (260 mg, 0.82 mmol), and Na_2_HPO_4_ (630 mg, 4.4 mmol) in acetonitrile (10 mL) gave **L6** as white crystals (88 mg, 37%). Mp: 86–87 °C. ^1^H-NMR (CDCl_3_): *δ* 7.11 (d, ^3^*J*_HH_ = 12 Hz, 1H, Ar-*H*), 7.10 (AB, ^3^*J*_HH_ = 8 Hz, 2H, Ar-*H*), 7.07 (AB, ^3^*J*_HH_ = 8.0 Hz, 2H, Ar-*H*), 6.66 (dd, ^3^*J*_HH_ = 8.0 Hz, ^4^*J*_HH_ = 4.0 Hz, 1H, Ar-*H*), 6.54 (d, ^4^*J*_HH_ = 4.0 Hz, 1H, Ar-*H*), 4.60 (d, ^3^*J*_HH_ = 4.0 Hz, 1H, C*H*OH), 3.78 (s, 3H, OC*H_3_*), 3.28 (vt, ^2^*J*_HH_ = 12 Hz, 1H, C*H_2_*), 3.20–3.16 (m, 1H, C*H_2_*), 3.04–2.99 (m, 1H, C*H*_2_), 2.69–2.58 (m, 5H, C*H_2_*), 2.54 (d, ^2^*J*_HH_ = 12 Hz, 1H, C*H_2_*), 2.43 (vt, ^2^*J*_HH_ = 12 Hz, 1H, C*H_2_*), 2.32 (s, 3H, C*H_3_*), 1.68–1.61 (m, 2H, C*H_2_*), and 1.59–1.53 (m, 2H, C*H_2_*). ^13^C{^1^H}-NMR (CDCl_3_): *δ* 159.14, 141.31, 139.36, 135.63, 135.41, 129.91, and 129.23 (*C*H), 128.46 (*C*H), 116.79 (*C*H), 110.43 (*C*H), 72.49 (*C*HO), 60.92 (*C*N), 59.83 (*C*N), 56.24 (*C*N), 55.42 (O*C*H_3_), and 35.89, 35.48, 29.42, 26.70, and 21.20 (*C*H_3_). HRMS calcd for C_22_H_30_NO_2_ [*M* + H]^+^: *m*/z = 340.2277, found 340.2271; error: −1.8 ppm. HPLC (general method A): *t*_R_ = 6.27 min, purity 100%.

7-Methoxy-3-(4-(4-bromophenyl)butyl)-2,3,4,5-tetrahydro-1*H*-benzo[*d*]azepin-1-ol (**L7**). Use of the method for **L5** with 7-methoxy-2,3,4,5-tetrahydro-1*H*-benzo[*d*]azepin-1-ol (210 mg, 1.1 mmol), **T7** (400 mg, 1.04 mmol), and Na_2_HPO_4_ (700 mg, 4.9 mmol) in acetonitrile (2 mL) with heating at 90 °C for 5 d gave **L7** as white crystals (0.35 g, yield 62%). Mp: 93–96 °C. ^1^H-NMR (CDCl_3_): *δ* 7.40 (d, ^3^*J*_HH_ = 8.0 Hz, 2H, Ar-*H*), 7.11 (d, ^3^*J*_HH_ = 8.0 Hz, 1H, Ar-*H*), 7.05 (d, ^3^*J*_HH_ = 8.0 Hz, 2H, Ar-*H*), 6.66 (dd, ^3^*J*_HH_ = 8.0 Hz, ^4^*J*_HH_ = 4.0 Hz, 1H, Ar-*H*), 6.64 (d, ^4^*J*_HH_ = 4.0 Hz, 1H, Ar-*H*), 4.62 (d, ^3^*J*_HH_ = 4.0 Hz, 1H, C*H*OH), 3.77 (s, 3H, O*C*H_3_), 3.28 (vt, ^2^*J*_HH_ = 12 Hz, 1H, C*H_2_*), 3.20–3.15 (m, 1H, C*H_2_*), 3.04–2.99 (m, 1H, C*H_2_*), 2.70–2.55 (m, 6H, C*H_2_*), 2.46 (vt, ^2^*J*_HH_ = 12 Hz, 1H, C*H_2_*), 1.66–1.60 (m, 2H, C*H_2_*), and 1.58–1.53 (m, 2H, C*H_2_*). ^13^C{^1^H}-NMR (CDCl_3_): *δ* 159.16, 141.31, 141.13, 135.44, and 131.59 (*C*H), 130.34 (*C*H), 129.87 and 119.72 (*C*H), 116.76 (*C*H), 110.49 (*C*H), 72.36 (*C*HO), 60.85 (*C*N), 59.65 (*C*N), 56.19 (*C*N), 55.41 (O*C*H_3_), and 36.64, 35.29, 29.08, and 26.48. HRMS calcd for C_21_H_27_BrNO_2_ [*M* + H]^+^: *m*/*z* = 404.1225, found 404.1229; error: 1.0 ppm. HPLC (general method A): *t*_R_ = 6.90 min, purity 96.64%.

7-Methoxy-3-(4-(4-fluorophenyl)butyl)-2,3,4,5-tetrahydro-1*H-*benzo[d]azepin-1-ol (**L8**). Use of the method for **L5** with 7-methoxy-2,3,4,5-tetrahydro-1*H*-benzo[*d*]azepin-1-ol (120 mg, 0.62 mmol), **T8** (260 mg, 0.81 mmol), and Na_2_HPO_4_ (450 mg, 3.2 mmol) in acetonitrile (2 mL) gave **L8** as a brown oil (0.23 g, yield 79%). ^1^H-NMR (CDCl_3_): *δ* 7.13 (d, ^3^*J*_HH_ = 8.0 Hz, 2H, Ar-*H*), 7.11 (d, ^3^*J*_HH_ = 8.0 Hz, 2H, Ar-*H*), 6.96 (t, ^3^*J*_HH_ = ^3^*J*_HF_ = 8.0 Hz, 2H, Ar-*H*), 6.65 (d, ^3^*J*_HH_ = 8.0 Hz, 1H, Ar-*H*), 6.63 (brs, 1H, Ar-*H*), 4.62 (d, ^3^*J*_HH_ = 4.0 Hz, 1H, C*H*OH), 3.77 (s, 3H, OC*H_3_*), 3.27 (vt, ^2^*J*_HH_ = 12 Hz, 1H, C*H_2_*), 3.19–3.15 (m, 1H, C*H_2_*), 3.03–2.99 (m, 1H, C*H_2_*), 2.70–2.55 (m, 6H, C*H_2_*), 2.46 (vt, ^2^*J*_HH_ = 12 Hz, 1H, C*H_2_*), 1.67–1.60 (m, 2H, C*H_2_*), and 1.58–1.53 (m, 2H, C*H_2_*). ^13^C{^1^H}-NMR (CDCl_3_): *δ* 162.64 (*C*-F), 160.23 (*C*-F), 159.16, 141.13, 137.97, 137.94, 135.46, and 129.89 (*C*H, *J*_CF_), 129.81 (*C*H, J_CF_), 116.75 (*C*H), 115.36 (*C*H, J_CF_), 115.15 (*C*H, *J*_CF_), 110.49 (*C*H), 72.34 (*C*HO), 60.86 (*C*N), 59.69 (*C*N), 56.17 (*C*N), 55.41 (O*C*H_3_), and 36.65, 35.07, 29.37, and 26.47. HRMS calcd for C_21_H_27_NO_2_F [*M* + H]^+^: *m*/*z* = 344.2026, found 344.2025; error: −0.3 ppm. HPLC (general method A): *t*_R_ = 5.55 min, purity 98.74%.

7-Methoxy-3-(4-(naphthalen-2-yl)butyl)-2,3,4,5-tetrahydro-1*H*-benzo[*d*]azepin-1-ol (**L9**). Use of the method for **L7** with 7-methoxy-2,3,4,5-tetrahydro-1*H*-benzo[*d*]azepin-1-ol (100 mg, 0.52 mmol), **T9** (250 mg, 0.71 mmol), and Na_2_HPO_4_ (280 mg, 2.0 mmol) in acetonitrile (10 mL) gave **L9** as white crystals (0.14 g, yield 74%). Mp: 86–88 °C. ^1^H-NMR (CDCl_3_): *δ* 7.81 (d, ^3^*J*_HH_ = 8.0 Hz, 1H, Nap-*H*), 7.78 (d, ^3^*J*_HH_ = 8.0 Hz, 1H, Nap-*H*), 7.77 (d, ^3^*J*_HH_ = 8.0 Hz, 1H, Nap-*H*), 7.60 (s, 1H, Nap-*H*), 7.44 (p, ^3^*J*_HH_ = 7.0 Hz, 2H, Nap-*H*), 7.31 (d, ^3^*J*_HH_ = 8.0 Hz, 1H, Ar-*H*), 7.14 (d, ^3^*J*_HH_ = 8.0 Hz, 1H, Ar-*H*), 6.68 (dd, ^3^*J*_HH_ = 8.0 Hz, ^4^*J*_HH_ = 4.0 Hz, 1H, Ar-*H*), 6.63 (d, ^4^*J*_HH_ = 4.0 Hz, 1H, Ar-*H*), 4.72 (d, ^3^*J*_HH_ = 4.0 Hz, 1H, C*H*OH), 3.76 (s, 3H, OC*H_3_*), 3.34 (vt, ^2^*J*_HH_ = 12 Hz, 1H, C*H_2_*), 3.29–3.24 (m, 1H, C*H_2_*), 3.11–3.07 (m, 1H, C*H_2_*), 2.81–2.66 (m, 6H, C*H_2_*), 2.57 (vt, ^2^*J*_HH_ = 12 Hz, 1H, C*H_2_*), 1.78–1.70 (m, 2H, C*H_2_*), and 1.69–1.62 (m, 2H, C*H_2_*). ^13^C{^1^H}-NMR (CDCl_3_): *δ* 159.20, 139.69, 134.96, 133.78, 132.19, and 129.79 (*C*H), 129.09 and 128.12 (*C*H), 127.79 (*C*H), 127.61 (*C*H), 127.40 (*C*H), 126.68 (*C*H), 126.11 (*C*H), 125.34 (*C*H), 116.61 (*C*H), 110.67 (*C*H), 71.66 (*C*HO), 60.57 (*C*N), 59.62 (*C*N), 55.91 (*C*N), 55.40 (O*C*H_3_), and 35.89, 35.69, 28.91, and 25.89. HRMS calcd for C_25_H_30_NO_2_ [*M* + H]^+^: *m*/*z* = 376.2277, found 376.2272; error: −1.3 ppm. HPLC (general method A): *t*_R_ = 5.69 min, purity 99.13%.

3-(4-(Pyridin-2-yl)butyl)-7-methoxy-2,3,4,5-tetrahydro-1*H*-benzo[*d*]azepin-1-ol (**L10**). Method 1. 7-Methoxy-2,3,4,5-tetrahydro-1*H*-benzo[*d*]azepin-1-ol (15.3 mg, 0.52 mmol), **T10** (39.7 mg, 0.66 mmol), and Na_2_HPO_4_ (73.3 mg, 2.0 mmol) were suspended in DMSO (1.0 mL). The reaction mixture was then heated in a microwave reactor (three conditions were utilized: (i) 80 °C, 10 min, 30 W, 250 psi; (ii) 120 °C, 10 min, 50 W, 250 psi; and (iii) 150 °C, 10 min, 60 W, 250 psi). Only a by-product was formed. LC-MS (Scheme 4) indicated this to be a cyclized product from the attack of the pyridinyl nitrogen on the tosylate leaving group of **T10**.

Method 2. 7-Methoxy-2,3,4,5-tetrahydro-1*H*-benzo[*d*]azepin-1-ol (150 mg, 0.52 mmol), **T10** (300 mg, 0.66 mmol) and Na_2_HPO_4_ (510 mg, 2.0 mmol) were suspended in acetonitrile (10 mL) and heated at 90 °C for 22 d. Only one by-product was formed. Again, LC-MS indicated that this was produced by intramolecular cyclization of **T10** (Scheme 4).

7-Methoxy-3-(4-(pyridin-2-yl)but-3-yn-1-yl)-2,3,4,5-tetrahydro-1*H*-benzo[*d*]azepin-1-ol (**L11**). Use of the method for **L7** in the same molar proportions with **T11** (240 mg, 0.80 mmol) gave **L11** a brown oil (0.268 g, yield 77%). ^1^H-NMR (CD_3_OD): *δ* 8.34 (ddd, ^3^*J*_HH_ = 5.0 Hz, 1 H, Ar-*H*), 7.68 (td, ^3^*J*_HH_ = 7.8 Hz, ^4^*J*_HH_ = 1.8 Hz, 1H, Ar-*H*), 7.37 (vt, ^3^*J*_HH_ = 7.9 Hz, 1H, Ar-*H*), 7.23 (dd, ^3^*J*_HH_ = 7.7 Hz, 1 H, Ar-*H*), 7.17 (d, ^3^*J*_HH_ = 8.4 Hz, 1H, Ar-*H*), 6.62 (dd, ^3^*J*_HH_ = 8.3 Hz, ^4^*J*_HH_ = 2.7 Hz, 1H, Ar-*H*), 6.57 (d, ^4^*J*_HH_ = 2.6 Hz, 1H, Ar-*H*), 4.67 (d, ^3^*J*_HH_ = 7.6 Hz, 1H, C*H*OH), 3.66 (s, 3H, OC*H_3_*), 2.92 (m, 1H, C*H_2_*), 2.83 (m, 3H, C*H_2_*), 2.73 (m, 3H, C*H_2_*), and 2.59 (m, 3H, C*H_2_*). ^13^C{^1^H}-NMR in (CD_3_OD): *δ* 160.35 and 150.42 (*C*H), 144.54, 142.03, 138.66, 137.06, and 128.66 (*C*H), 128.37 (*C*H), 124.42 (*C*H), 116.76 (*C*H), 111.69 (*C*H), 91.08, 81.69, and 72.97 (*C*HO), 63.09 (*C*N), 58.77 (*C*N), 56.21 (*C*N), 55.76 (O*C*H_3_), 36.99 (*C*H_2_), and 18.12 (*C*H_2_). HRMS calcd for C_20_H_23_N_2_O_2_ [*M* + H]^+^: *m*/*z* = 323.1760, found 323.1757; error: −0.9 ppm. HPLC (general method B): *t*_R_ = 6.50 min, purity 100%.

3-(4-(6-Fluoropyridin-2-yl)but-3-yn-1-yl)-7-methoxy-2,3,4,5-tetrahydro-1*H*-benzo[*d*]azepin-1-ol (**L12**). 7-Methoxy-2,3,4,5-tetrahydro-1*H*-benzo[*d*]azepin-1-ol (134 mg, 0.69 mmol) and **T12** (274 mg, 0.86 mmol) were mixed and then heated in a microwave reactor (135 °C, 10 min, 60 W, 250 psi) to give a black solid upon cooling to RT. The solid was dissolved in DMF (3 mL), passed through a 0.2 μm syringe, and then purified with HPLC (general method C). The solvent was then removed under vacuum. The residue was redissolved in acetonitrile, passed through a 0.2 μm syringe filter, and then dried with Centrifan to give **L12** as a brown oil (29 mg, yield 12%). ^1^H-NMR (CDCl_3_) *δ* 7.70 (dd, ^3^*J*_HH_ = 8.0 Hz, ^3^*J*_HF_ = 16.0, 1H, Ar-*H*), 7.28 (dd, ^3^*J*_HH_ = 7.1 Hz, ^4^*J*_HH_ = 1.8 Hz, 1H, Ar-*H*), 7.11 (d, ^3^*J*_HH_ = 7.9 Hz, 1H, Ar-*H*), 6.86 (dd, ^3^*J*_HH_ = 8.3 Hz, ^4^*J*_HH_ = 2.5 Hz, 1H, Ar-*H*), 6.67 (dd, ^3^*J*_HH_ = 8.3 Hz, ^4^*J*_HH_ = 2.6 Hz, 1H, Ar-*H*), 6.65 (s, 1H, Ar-*H*), 4.63 (d, ^3^*J*_HH_ = 6.8 Hz, 1H, C*H*-OH), 3.78 (s, 3H, OC*H_3_*), 3.27 (m, 2H, C*H_2_*), 3.09 (m, 1H, C*H_2_*), 2.96 (t, ^3^*J*_HH_ = 7.1 Hz, 2H, C*H_2_*), 2.68 (m, 4H, C*H_2_*), and 2.58 (t, ^3^*J*_HH_ = 12.0 Hz, 1H, C*H_2_*). ^13^C{^1^H}-NMR(CDCl_3_): *δ* 163.08 (d, ^1^*J*_CF_ = 240 Hz, 1C, *C*F), 159.18 and 141.62 (d, ^3^*J*_CF_ = 15.1 Hz, 1C, *C*NCF), 141.37 (d, ^3^*J*_CF_ = 8.2 Hz, 1C, *C*HCHCF), 141.19, 135.50, 130.06 and 124.54 (d, ^4^*J*_CF_ = 4.2 Hz, 1C, *C*HCHCHCF), 116.87 (*C*H), 110.50 (*C*H), 109.22 (d, ^2^*J*_CF_ = 36.7 Hz, 1C, *C*HCF), 90.15, 80.64, and 72.73 (*C*HO), 60.70 (*C*N), 58.07 (*C*N), 55.96 (*C*N), 55.42 (O*C*H_3_), 37.32 (*C*H_2_), and 18.27 (*C*H_2_). HRMS calcd for C_20_H_22_N_2_O_2_F [*M* + H]^+^: *m*/*z* = 341.1665, found 323.1667, error: 0.6 ppm. HPLC (general method B): *t*_R_ = 7.60 min, purity 98.69%.

3-(2-(Benzofuran-2-yl)ethyl)-7-methoxy-2,3,4,5-tetrahydro-1*H*-benzo[*d*]azepin-1-ol (**L13**). 7-Methoxy-2,3,4,5-tetrahydro-1*H*-benzo[*d*]azepin-1-ol (100 mg, 0.52 mmol), **T13** (184 mg, 0.582 mmol) and Na_2_HPO_4_ (250 mg, 1.76 mmol) were suspended in acetonitrile (10 mL) and heated at 90 °C for 22 d to give **L13** as a brown oil (139 mg, yield 71%). ^1^H-NMR (CD_3_OD): *δ* 7.48 (dd, ^3^*J*_HH_ = 7.1 Hz, ^4^*J*_HH_ = 1.4, 1H, Ar-*H*), 7.39 (d, *^3^J*_HH_ = 8.1 Hz, 1H, Ar-*H*), 7.28 (d, ^3^*J*_HH_ = 8.3 Hz, 1H, Ar-*H*), 7.20 (td, ^3^*J*_HH_ = 7.3 Hz, ^4^*J*_HH_ = 1.4 Hz, 1H, Ar-*H*), 7.16 (td, ^3^*J*_HH_ = 7.4 Hz, ^4^*J*_HH_ = 1.2 Hz, 1H, Ar-*H*), 6.73 (dd, ^3^*J*_HH_ = 8.3 Hz, ^4^*J*_HH_ = 2.6 Hz, 1H, Ar-*H*), 6.68 (d, ^4^*J*_HH_ = 2.6 Hz, 1H, Ar-*H*), 6.53 (s, 1H, Ar-*H*), 4.79 (d, ^3^*J*_HH_ = 8.0 Hz, 1H, C*H*OH), 3.77 (s, 3H, OC*H_3_*), 3.03 (t, ^3^*J*_HH_ = 3.1 Hz, 4H, C*H_2_*), 2.97 (m, 2H, C*H_2_*), 2.86 (m, 2H, C*H_2_*), 2.78 (t, ^3^*J*_HH_ = 11.7 Hz, 1H, C*H_2_*), and 2.66 (t, ^3^*J*_HH_ = 10.5 Hz, 1H, C*H_2_*). ^13^C{^1^H}-NMR (CD_3_OD): *δ* 160.31, 158.78, 156.28, 141.96, 137.06, 130.40, and 128.27 (*C*H), 124.54 (*C*H), 123.72 (*C*H) and 121.51 (*C*H), 116.75 (*C*H), 111.67 (*C*H), 103.90 (*C*H), 72.82 (*C*HO), 63.21 (*C*N), 58.41 (*C*N), 56.28 (*C*N), 55.75 (O*C*H_3_), 36.87 (*C*H_2_), and 27.00 (*C*H_2_). HRMS calcd for C_21_H_24_NO_3_ [*M* + H]^+^: *m*/*z* = 338.1756, found 338.1751; error: −1.5 ppm. HPLC (general method A): *t*_R_ = 4.59 min, purity 97.52%

3-(2-(5-Bromobenzofuran-2-yl)ethyl)-7-methoxy-2,3,4,5-tetrahydro-1*H*-benzo[*d*]azepin-1-ol (**L14**). Use of the method for **L12** in the same molar proportions with **T14** (142 mg, 0.359 mmol) gave **L14** as a brown oil (76 mg, yield 51%). ^1^H-NMR (CDCl_3_): *δ* 7.61 (s, 1H, Ar-*H*), 7.33 (AB, ^3^*J*_HH_ = 8.0 Hz, 1H, Ar-*H*), 7.27 (AB, ^3^*J*_HH_ = 8.0 Hz, 1H, Ar-*H*), 7.21 (d, ^3^*J*_HH_ = 8.0 Hz, 1H, Ar-*H*), 7.13 (d, ^3^*J*_HH_ = 8.0 Hz, 1H, Ar-*H*), 6.72 (d, ^3^*J*_HH_ = 8.0 Hz, 1H, Ar-*H*), 6.65 (s, 1H, Ar-*H*), 6.46 (s, 1H, Ar-*H*), 4.92 (brs, 1H, C*H*OH), 3.78 (s, 3H, OC*H_3_*), 3.34 (brs, 2H, C*H_2_*), 3.21 (brs, 2H, C*H_2_*), 3.13 (q, ^3^*J*_HH_ = 8.0 Hz, 2H, C*H_2_*), 2.75–2.69 (m, 2H, C*H_2_*), and 1.32 (t, ^3^*J*_HH_ = 8.0 Hz, 2H, C*H_2_*). ^13^C{^1^H}-NMR (CDCl_3_): *δ* 159.30, 153.68, and 130.84 (*C*H), 130.07, 129.12, and 126.67 (CH), 126.13 and 123.35 (*C*H), 116.77 (*C*H), 115.95 (*C*H), 112.46 (*C*H), 110.73 (*C*H), 103.10 (*C*Br), 72.03 (*C*HO), 60.52 (*C*N), 57.72 (*C*N), 56.00 (*C*N), 55.45 (O*C*H_3_), and 45.90 and 26.33. HRMS calcd for C_21_H_23_BrNO_3_ [*M* + H]^+^: *m*/*z* = 416.0861, found 416.0865; error: 1.0 ppm. HPLC (general method A): *t*_R_ = 6.52 min, purity 100%.

3-(2-(5-Fluorobenzofuran-2-yl)ethyl)-7-methoxy-2,3,4,5-tetrahydro-1*H*-benzo[*d*]azepin-1-ol (**L15**). Use of the method for **L12** in the same molar proportions with **T15** (214 mg, 0.640 mmol) gave **L15** as a brown wax (134 mg, yield 59%). ^1^H-NMR (CD_3_OD): *δ* 7.38 (dd, ^3^*J*_HH_ = 3.9 Hz, 1H, Ar-*H*), 7.26 (d, ^3^*J*_HH_ = 8.3 Hz, 1H, Ar-*H*), 7.20 (dd, ^3^*J*_HH_ = 8.8, ^4^J_HH_ = 2.4 Hz, 1H, Ar-*H*), 6.96 (td, ^3^*J*_HH_ = 9.3 Hz, ^4^*J*_HH_ = 2.9 Hz, 1H, Ar-*H*), 6.74 (dd, ^3^*J*_HH_ = 8.4 Hz, ^4^*J*_HH_ = 2.4 Hz, 1H, Ar-*H*), 6.71 (d, ^4^*J*_HH_ = 2.4 Hz, 1H, Ar-*H*), 6.58 (s, 1H, Ar-*H*), 4.82 (t, ^3^*J*_HH_ = 4.6 Hz C*H*-OH), 3.77 (s, 3H, O-C*H_3_*), 3.13 (br, 5H, C*H_2_*), and 2.95 (br, 5H, C*H_2_*). ^13^C{^1^H}-NMR (CD_3_OD): *δ* 160.78 (d, ^1^*J*_CF_ = 235 Hz, 1C, *C*F), 160.66, 159.93, 152.60, 141.64, 136.24, and 131.29 (d, ^3^*J*_CF_ = 11.0 H, 1C, *C*HCHCF), 129.17 and 116.94 (*C*H), 112.53 (d, ^3^*J*_CF_ = 9.9 Hz, 1C, *C*HCHCF), 112.20 (*C*H), 111.95 (*C*H), 107.06 (d, ^2^*J*_CF_ = 25.2 Hz, 1C, *C*HCF), 104.71 (d, ^4^*J*_CF_ = 3.7 Hz, 1C, *C*HCHCHCF), 72.34 (*C*HO), 62.21 (*C*N), 58.09 (*C*N), 56.62 *(C*N), 55.80 (O*C*H_3_), 35.75 (*C*H_2_), and 26.43 (*C*H_2_). HRMS calcd for C_21_H_23_NO_3_F [*M* + H]^+^: *m*/*z* = 356.1662, found 356.1658; error: −1.1 ppm. HPLC (general method A): *t*_R_ = 5.38 min, purity 99.57%.

3-(2-(5-Trifluoromethylbenzofuran-2-yl)ethyl)-7-methoxy-2,3,4,5-tetrahydro-1*H*-benzo[*d*]azepin-1-ol (**L16**). Use of the method for **L11** in the same molar proportions with **T16** (178 mg, 0.463 mmol) gave **L16** as a thick brown oil (120 mg, yield 64%). ^1^H-NMR (CD_3_OD): *δ* 7.85 (s, 1H, Ar-*H*), 7.58 (AB, ^3^*J*_HH_ = 8.6 Hz, 1H, Ar-*H*), 7.52 (AB, ^3^*J*_HH_ = 8.6 Hz, ^4^*J*_HH_ = 1.5 Hz, 1H, Ar-*H*), 7.28 (d, ^3^*J*_HH_ = 8.4 Hz, 1H, Ar-*H*), 6.73 (dd, ^3^*J*_HH_ = 8.4 Hz, ^4^*J*_HH_ = 2.6 Hz, 1H, Ar-*H*), 6.68 (m, ^3^*J*_HH_ = 4.0 Hz, 2H, Ar-*H*), 4.78 (d, ^3^*J*_HH_ = 7.6 Hz, 1H, C*H*-OH), 3.77 (s, 3H, OC*H_3_*), 3.06 (m, 4H, C*H_2_*), 2.99 (m, 1H, C*H_2_*), 2.95 (m, 1H, C*H_2_*), 2.83 (m, 2H, C*H_2_*), 2.77 (m, 1H, C*H_2_*), and 2.67 (m, 1H, C*H_2_*). ^13^C{^1^H}-NMR (CD_3_OD): *δ* 161.42, 160.34, and 157.71 (d, ^4^*J*_CF_ = 1.67 Hz, 1C, *C*HCHCHCF), 141.96, 130.75, and 128.28 (*C*H), 126.41 (q, *^1^J_CF_* = 270.0 Hz, 1C, *C*F), 126.41 (q, ^2^*J*_CF_ = 31.4 Hz, 1C, *C*HCF), 121.64 (q, ^3^*J*_CF_ = 3.6 Hz, 1C, *C*HCHCF), 119.15 (q, ^3^*J*_CF_ = 4.3 Hz, 1C, *C*HCHCF), 116.74 (*C*H), 112.35 (*C*H), 111.68 (*C*H), 104.31 (*C*H), 72.89 (*C*HO), 63.23 (*C*N), 58.14 (*C*N), 56.28 (*C*N), 55.75 (O*C*H_3_), 36.87 (*C*H_2_), and 27.03 (*C*H_2_). HRMS calcd for C_22_H_23_NO_3_F_3_ [*M* + H]^+^: *m*/*z* = 406.1630, found 406.1625; error: −1.2 ppm. HPLC (general method A): *t*_R_ = 6.81 min, purity 96.43%

3-(4-(Benzofuran-2-yl)butyl)-7-methoxy-2,3,4,5-tetrahydro-1*H*-benzo[*d*]azepin-1-ol (**L17**). Use of the method for **L12** in the same molar proportions with **T17** (257 mg, 0.746 mmol) gave **L17** as white crystals (194 mg, yield 71%). Mp: 90–92 °C. ^1^H-NMR (CD_3_OD): *δ* 7.47 (dd, ^3^*J*_HH_ = 7.0 Hz, ^4^*J*_HH_ = 1.9 Hz, 1H, Ar-*H*), 7.37 (d, ^3^*J*_HH_ = 8.0 Hz, 1H, Ar-*H*), 7.29 (d, ^3^*J*_HH_ = 8.5 Hz, 1H, Ar-*H*), 7.18 (td, ^3^*J*_HH_ = 7.2 Hz, ^4^*J*_HH_ = 1.5 Hz, 1H, Ar-*H*), 7.14 (td, ^3^*J*_HH_ = 7.3 Hz, ^4^*J*_HH_ = 1.3 Hz, 1H, Ar-*H*), 6.72 (dd, ^3^*J*_HH_ = 8.4 Hz, ^4^*J*_HH_ = 2.6 Hz, 1H, Ar-*H*), 6.66 (d, ^4^*J*_HH_ = 2.6 Hz, 1H, Ar-*H*), 6.47 (d, ^4^*J*_HH_ = 0.6 Hz, 1H, Ar-*H*), 4.79 (d, ^3^*J*_HH_ = 8.1 Hz, 1H, C*H*-OH), 3.75 (s, 3H, OC*H_3_*), 2.89 (m, 6H, C*H_2_*), 2.60 (m, 3H, C*H_2_*), 2.44 (br, 1H, C*H_2_*), 1.79 (quint, 2H, C*H_2_*), and 1.65 (quint, 2H, C*H_2_*). ^13^C{^1^H}-NMR (CD_3_OD): *δ* 160.59, 160.24, 156.28, 141.79, 137.22, and 130.50 (*C*H), 127.68 (*C*H), 124.38, 123.64, and 121.41 (*C*H), 116.57 (*C*H), 111.71 (*C*H), 111.62 (*C*H), 103.26 (*C*H), 72.37 (*C*HO), 63.70 (*C*N), 60.01 (*C*N), 56.52 (*C*N), 55.75 (O*C*H_3_), 36.51 (*C*H_2_), 29.18 (*C*H_2_), 27.20 (*C*H_2_), and 26.89 (*C*H_2_). HRMS: calcd for C_23_H_28_NO_3_ [*M* + H]^+^: *m*/*z* = 366.2069, found 366.2069; error: 0.0 ppm. HPLC (general method A): *t*_R_ = 6.34 min, purity 99.00%.

7-Methoxy-3-(4-(5-(trifluoromethyl)benzofuran-2-yl)butyl)-2,3,4,5-tetrahydro-1*H*-benzo[*d*]azepin-1-ol (**L18**). Use of the method for **L12** in the same molar proportions with **T18** (197 mg, 0.478 mmol) gave **L18** as a thick brown oil (141 mg, yield 68%). ^1^H-NMR (CD_3_OD): *δ* 7.83 (s, 1H, Ar-*H*), 7.56 (AB, ^3^*J*_HH_ = 8.6 Hz, 1H, Ar-*H*), 7.51 (AB, ^3^*J*_HH_ = 8.6 Hz, ^4^*J*_HH_ = 1.3 Hz, 1 H, Ar-*H*), 7.29 (d, ^3^*J*_HH_ = 8.4 Hz, 1 H, Ar-*H*), 6.72 (dd, ^3^*J*_HH_ = 8.4 Hz, ^4^*J*_HH_ = 2.6 Hz, 1 H, Ar-*H*), 6.67 (d, ^4^*J*_HH_ = 2.5 Hz, 1 H, Ar-*H*), 6.63 (d, ^4^*J*_HH_ = 0.8 Hz, 1 H, Ar-*H*), 4.79 (d, ^3^*J*_HH_ = 7.8 Hz, 1H, C*H*-OH), 3.76 (s, 3H, OC*H_3_*), 2.90 (m, 6H, C*H_2_*), 2.61 (br, 3H, C*H_2_*), 2.51 (br, 1H, C*H_2_*), 1.82 (quint, ^3^*J*_HH_ = 7.8 Hz, 2H, C*H_2_*), and 1.67 (quint, ^3^*J*_HH_ = 7.8 Hz, 2H, C*H_2_*). ^13^C{^1^H}-NMR (CD_3_OD): *δ* 163.13, 160.28, 157.71, 144.77, 137.15, 130.83, and 127.78 (*C*H), 126.44 (q, ^1^*J*_CF_ = 270.8 Hz, 1C, *C*F), 126.35 (q, ^2^*J*_CF_ = 31.6 Hz, 1C, *C*HCF), 121.52 (q, ^3^*J*_CF_ = 3.7 Hz, 1C, *C*HCHCF), 119.06 (q, ^3^*J*_CF_ = 4.1 Hz, 1C, *C*HCHCF), 116.59 (*C*H), 112.30 (*C*H), 111.73 (*C*H), 103.64 (*C*H), 72.39 (*C*HO), 63.60 (*C*N), 59.94 (*C*N), 56.56 (*C*N), 55.75 (O*C*H_3_), 36.46 (*C*H_2_), 29.14 (*C*H_2_), 27.15 (*C*H_2_), and 26.70 (*C*H_2_). HRMS calcd for C_23_H_28_NO_3_ [*M* + H]^+^: *m*/*z* = 434.1943, found 434.1948; error: 1.2 ppm. HPLC (general method A): *t*_R_ = 8.06 min, purity 98.37%.

Methyl 3-((4-(4-(1-hydroxy-7-methoxy-1,2,4,5-tetrahydro-3*H*-benzo[*d*]azepin-3-yl)butyl)phenyl)thio)propanoate (**L19**). **L2** (0.2067 g, 0.458 mmol), methyl 3-mercaptopropanoate (62 µL, *d* = 1.085 g/mL, 0.560 mmol), *N,N*,1,1,1-pentamethylstannanamine (90 µL, 0.552 mmol), and Pd(PPh_3_)_4_ (120 mg, 0.104 mmol) were dissolved in DMSO (4.0 mL). and then heated in a microwave reactor (110 °C, 10 min, 50 W, 250 psi). LC-MS analysis showed consumption of the starting material **L2**, and this was confirmed with HPLC (general method A). The reaction mixture was purified with HPLC (general method D). The product fractions were collected, dried under vacuum, redissolved in ethanol, passed through a 0.2 μm syringe filter, and then dried with Centrifan to give **L19** as a brown wax (174.3 mg, yield 86%). ^1^H-NMR (CD_3_OD): *δ* 7.30 (d, ^3^*J*_HH_ = 6.7 Hz, 2H, Ar-*H*), 7.30 (t, ^3^*J*_HH_ = 4.5 Hz, 1H, Ar-*H*), 7.16 (d, ^3^*J*_HH_ = 8.2 Hz, 2H, Ar-*H*), 6.73 (dd, ^3^*J*_HH_ = 8.3 Hz, ^4^*J*_HH_ = 2.6 Hz, 1H, Ar-*H*), 6.67 (d, ^4^*J*_HH_ = 2.5 Hz, 1H. Ar-*H*), 4.79 (d, ^3^*J*_HH_ = 8.0 Hz, 1H, C*H*OH), 3.76 (s, 3H, OC*H_3_*), 3.65 (s, 3H, OC*H_3_*), 3.11 (t, ^3^*J*_HH_ = 7.1 Hz, 2H, C*H_2_*), 2.88 (m, 4H, C*H_2_*), 2.61 (m, 8H, C*H_2_*), and 1.63 (m, 4H, C*H_2_*). ^13^C{^1^H}-NMR (CD_3_OD): *δ* 174.10 (*C*=O), 160.31, 142.71, 141.71, 138.65, 137.04, and 133.59 (*C*H), 132.03 (*C*H), 130.43 (*C*H), 127.88 (*C*H), 116.61 (*C*H), 111.76 (*C*H), 72.24 (O*C*H), 63.45 (*C*N), 60.18 (*C*N), 56.55 (*C*N), 55.77 (O*C*H_3_), 52.36 (O*C*H_3_), 36.29 (*C*H_2_), 35.34 (*C*H_2_), 30.92 (*C*H_2_), 30.62 (*C*H_2_), 30.44 (*C*H_2_), and 27.04 (*C*H_2_). HRMS calcd for C_25_H_34_NO_4_S [*M* + H]^+^: *m*/*z* = 444.2209, found 444.2203; error: −1.4 ppm. HPLC (general method A): *t*_R_ = 6.46 min, purity 97.56%.

7-Methoxy-3-(4-(4-(4,4,5,5-tetramethyl-1,3,2-dioxaborolan-2-yl)phenyl)butyl)-2,3,4,5-tetrahydro-1*H*-benzo[*d*]azepin-1-ol (**L20**). Method 1. Iodo compound **L2** (105.7 mg, 0.234 mmol), 4,4,4′,4′,5,5,5′,5′-octamethyl-2,2′-bi(1,3,2-dioxaborolane) (B_2_Pin_2_) (86.9 mg, 0.342 mmol), KOAc (83.7 mg, 0.853 mmol), and Pd(DPPF)Cl_2_·CH_2_Cl_2_ (34.8 mg, 0.0426 mmol) were dissolved in DMSO (3.0 mL) and heated in a microwave reactor (80 °C, 60 min, 60 W, 250 psi). LC-MS analysis showed consumption of the starting material **L2** and this was confirmed with HPLC (general method A). The reaction mixture was purified with HPLC (general method D). A saturated NaCl solution was immediately added to each product fraction as they eluted, followed by a saturated NaHCO_3_ solution to prevent decomposition. The aqueous phase was then extracted with acetonitrile and dried (MgSO_4_), filtered, and then dried under vacuum. The residue was redissolved in ethanol, passed through a 0.2 μm syringe filter, and then dried with Centrifan to give **L20** as a purple wax (39.0 mg, yield 37%). ^1^H-NMR (CD_3_CN): *δ* 7.63 (d, ^3^*J*_HH_ = 7.7 Hz, 2H, Ar-*H*), 7.23 (d, *^3^J*_HH_ = 7.6 Hz, 2H, Ar-*H*), 7.17 (d, ^3^*J*_HH_ = 8.0 Hz, 1H, Ar-*H*), 6.68 (m, 2H, Ar-*H*), 4.61 (d, ^3^*J*_HH_ = 7.3 Hz, 1H, C*H*OH), 3.74 (s, 3H, OC*H_3_*), 2.85 (m, 1H, C*H_2_*), 2.73 (m, 2H, C*H_2_*), 2.65 (m, 2H, C*H_2_*), 2.58 (m, 2H, C*H_2_*), 1.64 (q, ^3^*J*_HH_ = 7.6 Hz, 2H, C*H_2_*), 1.54 (m, 2H, C*H_2_*), and 1.31 (s, 12H, C*H_3_*). ^13^C{^1^H}-NMR (CD_3_CN): *δ* 159.27, 146.85, 136.67, 135.12, 128.70 (*C*H), 128.57 (*C*H), 117.88 (*C*H), 116.35 (*C*H), 110.76 (*C*H), 84.23 (*C*OC_3_), 72.02 (*C*OH), 62.06 (*C*N), 59.21 (*C*N), 55.98 (*C*N), 55.35 (O*C*H_3_), 36.29 (*C*H_2_), 35.94 (*C*H_2_), 29.27 (*C*H_2_), 26.68 (*C*H_2_), and 24.77 (*C*H_3_). HRMS calcd for C_27_H_39_BNO_4_ [*M*+H]^+^: *m*/*z* = 452.2972, found 452.2976; error: 0.9 ppm. HPLC (general method A): *t*_R_ = 7.64 min, purity 76.67%.

Method 2. Bromo compound **L7** (18.7 mg, 0.0462 mmol), Bpin2 (57 mg, 0.224 mmol), KOAc (7.9 mg, 0.0805 mmol), and Pd(DPPF)Cl_2_·CH_2_Cl_2_ (13.2 mg, 0.0162 mmol) were dissolved in DMSO (0.5 mL) and then heated in a microwave reactor (110 °C, 30 min, 50 W, 250 psi). LC-MS analysis showed consumption of the starting material **L7** and this was confirmed with HPLC (general method A). The reaction mixture was purified with HPLC (general method D), and the collected fractions were dried under vacuum. The product was redissolved in ethanol, passed through a 0.2 μm syringe filter, and then dried with Centrifan to give **L20** as a purple wax (11.2 mg, yield 54%).

#### 3.3.3. Infrared/Vibrational Circular Dichroism

The enantiomers of **L3** were subjected to a computational infrared (IR) and vibrational circular dichroism (VCD) study to allow comparison of computed spectra with those determined experimentally, and to define the absolute configurations of the enantiomers (see Supplementary Information). Chiral HPLC (general method E) showed two enantiomers: enantiomer 1 (*t*_R_ = 8.05 min, 48.03%) and enantiomer 2 (*t*_R_ = 13.22 min, 48.06%). The enantiomers were purified with chiral HPLC (general method F). Enantiomer 1: chiral HPLC (general method E) (*t*_R_ = 8.10 min, 98.01%), [α]D^20^ = –39.02° (*c* 1.0, CHCl_3_), and [α]D^20^ = +8.89° (*c* 1.0, EtOH). Enantiomer 2: chiral HPLC (general method E) (*t*_R_ = 13.27 min, 99.19%), [α]D^20^ = +34.03° (*c* 1.0, CHCl_3_), and [α]D^20^ = −3.67° (*c* 1.0, EtOH). The first eluting enantiomer in chiral HPLC analysis (general method E) was found to be the *S*-enantiomer.

#### 3.3.4. Verification of the Absolute Configuration of Enantiomers of L6, L2, L19, and L20 by Stereo-Retentive Transformations Originating from (S)-L3

Synthesis of (*S*)-**L19** from (*S*)-**L2**. Chiral HPLC analysis (general method E) of racemic **L2** showed (*S*)**-L2** (*t*_R_ = 6.97 min, 50.35%) and (*R*)**-L2** (*t*_R_ = 10.96 min, 47.92%). The enantiomers were separated with preparative chiral HPLC (general method F). Analytical chiral HPLC (general method E) for (*S*)**-L2** showed *t*_R_ = 6.89 min. (HPLC purity 98.14%); [α]D^20^ = −24.45° (*c* 1.0, CHCl_3_). Chiral HPLC (general method E) for (*R*)-**L2** showed *t*_R_ = 11.24 min (HPLC purity 99.2%); [α]D^20^ = +28.57° (*c* 1.0, CHCl_3_).

For the synthesis of (*S*)-**L19**, compound (*S*)-**L2** (46.8 mg, 103.7 μmol), methyl 3-((trimethylstannyl)thio)propanoate (26.7 μL, 124.4 μmol), triethylamine (14.5 μL, 103.7 μmol), and Pd(DPPF)Cl_2_ (35.5 mg, 48.52 μmol) were dissolved in acetonitrile (1 mL). The reaction mixture was then heated in a microwave reactor (90 °C. 20 min, 120 W, 250 psi) and purified with HPLC (method G) to give compound (*S*)-**L19** as a brown oil (38.8 mg, 84.4%). Analytical chiral HPLC (general method E) showed *t*_R_ = 14.04 min (purity 97.24%). This synthesis was repeated on (*R*)-**L2** to give (*R*)-**L19**.

Analytical chiral HPLC (general method E): (*S*)-**L19**, *t*_R_ = 13.91 min, 50.24%; (*R*)-**L19**, *t*_R_ = 21.48 min, 48.23%, [α]D^20^ = +2.47° (*c* 1.0, CHCl_3_). Analysis of synthesized (*R*)-**L19** showed *t*_R_ = 20.25 min (97.71%), [α]D^20^ = −1.81° (*c* 1.0, CHCl_3_).

Synthesis of (*S*)-**L3** from (*S*)-**L19**. Analytical chiral HPLC (general method E) of racemic **L3** showed two compounds: enantiomer 1 (*t*_R_ = 8.05 min, 48.03%) and enantiomer 2 (*t*_R_ = 13.22 min, 48.06%). (*S*)-**L3** was prepared from (*S*)-**L19** to assign an absolute configuration to each enantiomer, as follows.

Compound (*S*)**-L19** (27.4 mg, 61.77 μmol) in DCM (1 mL) was mixed with methyl iodide (3.86 μL, 61.77 μmol) and 1 M tetra-*n*-butylammonium hydroxide (TBAOH; 129.7 μL, 129.7 μmol) and stirred at RT for 20 min. The reaction mixture was then diluted with DCM (5 mL) and washed thrice with water (10 mL). The solvent was removed by rotary evaporation. The residue was dissolved in DCM (1 mL) and purified with HPLC (general method F) to give compound (*S*)-**L3** as white crystals (17.3 mg, 75.4%); m.pt. 88−90 °C; analytical chiral HPLC (general method E): *t*_R_ = 8.10 min, purity 98.01%. [α]D^20^ = −39.02° (*c* 1.0, CHCl_3_), [α]D^20^ = +8.89° (*c* 1.0, EtOH). The synthesis was repeated with (*R*)-**L19** to give (*R*)-**L3**. Analytical chiral HPLC (general method E): *t*_R_ = 13.27 min, 99.19%. [α]D^20^ = +34.03° (*c* 1.0, CHCl_3_), [α]D^20^ = −3.67° (*c* 1.0, EtOH).

The enantiomers of racemic **L3** were separated with chiral HPLC (general method F). Co-injections of racemic **L3** with each synthesized **L3** enantiomer confirmed their retention times.

Synthesis of (*S*)-**L20** from (*S*)-**L2**. Compound (*S*)**-L2** (138 mg, 306 μmol), *bis*(pinacolato)diborane (B_2_Pin_2_, 200 mg, 788 μmol), potassium acetate (51.2 mg, 521 μmol), and Pd(DPPF)Cl_2_ (62.8 mg, 85.8 μmol) were dissolved in acetonitrile (3 mL). The reaction mixture was then heated in a microwave reactor (80 °C, 20 min, 150 W, 250 psi) and passed through a 0.2 μm syringe filter and purified with normal phase HPLC in three equal portions (method G). Fractions containing the product were immediately neutralized with saturated aqueous sodium bicarbonate (1 mL), extracted with ethyl acetate, and dried to give (*S*)-**L20** (43 mg, 31%) as a purple wax. Analytical chiral HPLC (general method E): *t*_R_ = 6.0 min, purity 93.5% (see Supporting Information). (*R*)-**L20** was likewise synthesized from (*R*)-**L2**. Analytical chiral HPLC (general method E): *t*_R_ = 8.81 min, purity 92.0% (see Supporting Information).

Synthesis of (*S*)-**L6** from (*S*)-**L20**. Analytical chiral HPLC of **L6** (general method E) showed enantiomer 1 (*t*_R_ = 5.76 min, 44.43%) and enantiomer 2 (*t*_R_ = 9.44 min, 51.46%). The assignment of the absolute configuration of each **L6** enantiomer was achieved by preparing (*S*)-**L6** from (*S*)-**L20**, as follows.

Compound (*S*)-**L20** (20.0 mg, 44.3 μmol) was dissolved in methanol (0.4 mL). Cesium fluoride (199.4 μmol) in methanol (1 M; 199.4 μL), methyl iodide (3.04 μL, 48.8 μmol), and tetrakis(triphenylphosphine)palladium(0) (Pd(PPh_3_)_4_; 5.2 mg, 4.50 μmol) were dissolved in acetonitrile (0.6 mL). The reaction mixture was then heated in a microwave reactor (90 °C, 20 min, 80 W, 250 psi), then passed through a 0.2 μm syringe filter, and purified by HPLC (method G) to give (*S*)-**L6** as a brown oil (12.6 mg, 83.9%); [α]D^20^ = −24.23° (*c* 1.0, CHCl_3_). Analytical chiral HPLC (general method E): *t*_R_ = 5.93 min, purity 98.28%.

(*R*)-**L6** was likewise synthesized from (*R*)-**L20**; [α]D^20^ = +28.23° (*c* 1.0, CHCl_3_). Chiral HPLC analysis (general method E): *t*_R_ = 9.57 min (99.28%).

#### 3.3.5. Methylation of L19 to Synthesize L3 Under Conditions Mimicking a ^11^C-Labeling Reaction 

A stock solution of **L19** in DMF (1.25 mg/mL) was prepared and 0.4 mL of this solution was transferred to a V-vial. A stock solution of iodomethane in DMF (1% *v*/*v*; 1 mL) was prepared and 7.0 µL of this solution was added to the **L19** solution and stirred for 2 min. TBAOH in methanol (1 M; 5.0 µL) was then added and this was stirred at RT for 5 min. LC-MS showed complete conversion of **L19** into **L3**. The solution was then dried with Centrifan, and the reaction mixture was dissolved in ethanol (0.1 mL) for chiral HPLC (general method E) and LC-MS analysis.

#### 3.3.6. Methylation of L20 to L6 Under Conditions Mimicking a ^11^C-Labeling Reaction

Pd_2_(dba)_3_ and P(*o*,*p*-tol)_3_ were mixed in the weight ratio of 1: 1.33. A portion of this mixture (0.6 mg; 0.562 µmol Pd) was taken and placed in a vial. A solution of **L20** in methanol (1.25 mg/mL; 0.40 mL) was added, followed by a solution of iodomethane in methanol (10% *w*/*w*; 7 µL). A stock solution (20 µL) of cesium fluoride in methanol (1 M) was then added to the reaction mixture, which was heated at 80 °C for 90 min. LC-MS showed consumption of the starting material (**L20**) and product formation (**L6**). The reaction mixture was then purified with reversed-phase HPLC (general method C) and the product was dried with Centrifan. The purified product was analyzed with chiral HPLC (general method E) and LC-MS.

### 3.4. In Vitro Binding Assays

#### 3.4.1. PDSP Binding Assays

Selected compounds from early in the project were assayed by the National Institutes of Health (NIH) Psychoactive Drug Screening Program (PDSP) for *K*_i_ at the rat brain GluN2B subtype (https://pdspdb.unc.edu/pdspweb/ accessed on 4 August 2017). The assay was performed either manually or automatically.

A suspension of transiently transfected mouse fibroblast cell membrane homogenates was prepared as reported at a concentration of ~500,000 cells/mL. This suspension was sonicated, and aliquots (100 μL) were added to each of four tubes (a total of 48 in a rack). A solution of [^3^H]ifenprodil (2.22 TBq/mmol, 0.01 kBq/μL; American Radiochemical Company, Inc.; St. Louis, MO, USA) in phosphare-buffered saline (PBS; 100 μL) was added to each tube. Ifenprodil or other displacer (test compound) was dissolved in DMSO to give a 1 mM stock solution, which was further diluted with DMSO to give solutions ranging in known concentration from 10^−5^ to 10^−10^ M. Then, 10 μL of each solution was added to a separate tube. The content of each tube was then diluted to 1.0 mL with PBS, vortexed, and incubated at 37 °C for 2 h. After separation of tube contents with a cell harvester, filter paper (GF/B; Whatman; Derwood, MD, USA), pretreated with 0.5% polyethyleneimine solution, was washed with PBS (3 mL × 3). Each filter was then placed in a 7-mL plastic vial. Scintillation fluid (4 mL) was added to each vial. The scintillation vials were incubated overnight and then counted for radioactivity. The data were analyzed with Prism 7 version 7.03 (GraphPad Software; San Diego, CA, USA) with ‘one site competition’ curve-fitting. *K*_i_ values were calculated according to the Cheng–Prusoff equation [37]: *K*_i_ = *IC*_50_/(1 + [L]/*K*_D_), where [L] is the concentration (0.4 nM) and *K*_D_ the equilibrium dissociation constant (7.6 nM) of the reference radioligand. The latter was determined with ‘Scatchard analysis’ of homologous displacement from multiple runs with self-displacement from membrane homogenates.

#### 3.4.2. In-House Binding Assay

The assay procedure with [^3^H]ifenprodil as reference radioligand was the same as above, but with a different protein source. The rat brain was homogenized in PBS (1×)-water (1:24 *v*/*v*), and the protein concentration was measured. The protein concentration used for the binding assay ranged from 0.8 to 3.5 mg/mL. A solution of [^3^H]ifenprodil (2.22 TBq/mmol, 0.01 kBq/μL) in PBS (100 μL) was added to each tube (10 nM).

## 4. Conclusions

In this study, we designed, synthesized, and evaluated a series of 3-alkylaryl derivatives of 7-methoxy-2,3,4,5-tetrahydro-1*H*-benzo[*d*]azepin-1-ol as ligands for the GluN2B subunit of the NMDA receptor. Systematic structural variation revealed that binding affinity is governed primarily by linker length, with a flexible four-carbon tether providing optimal positioning within the binding site, while the terminal aryl group is broadly tolerant to electronic and steric modification. The lack of correlation between affinity and lipophilicity further indicates that productive binding is driven by specific spatial and local interactions rather than global physicochemical properties. GluN2B binding affinity showed sensitivity to ligand absolute configuration, as explicable in docking studies.

From this series, ligands **L3** and **L6**, together with their enantiomers, emerged as promising candidates for PET radiotracer development, combining nanomolar affinity, favorable properties for brain imaging, and compatibility with carbon-11 labeling. Radiolabeled analogs demonstrated strong and specific binding in vivo, supporting their suitability for further translational evaluation.

Overall, these findings define key structure–activity relationships for this scaffold and establish 3-substituted 7-methoxy benzazepinols as a robust platform for the development of GluN2B PET radioligands.

## Figures and Tables

**Table 1:** *K*_i_ values for the binding of ligands **L1**–**L9** and **L11**–**L19** to rat brain GluN2B, clog*D* values, and CNS PET MPO scores.

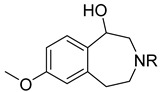						
Ligand	R	*K*_i_ from Automated PDSP Assay ^a^ (nM)	*K*_i_ from Manual PDSP Assay ^a^ (nM)	*K*_i_ from In-House Assay ^b^ (nM)	clog*D* ^c^	CNS PET MPO ^d^
**L1** (WMS 1405)	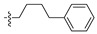	44 ± 7	9.8 ± 2	263 ± 50	3.13	2.5
**L2**	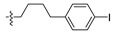	21 ± 4	2.2	1272 ± 250	4.12	1.7
**L3** (NR2B-SMe)	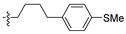	20 ± 5	2.0 ^e^		3.39 ^e^	1.8
(*S*)-**L3**	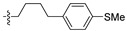			13 ± 3	3.39	1.8
**L4**	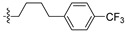			309 ± 60	3.83	1.7
**L5**	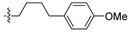	44 ± 9	9 ± 2		2.90	2.9
**L6** (NR2B-Me)	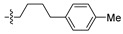	31 ± 5	4.9 ± 1 ^f^		3.34	2.0
(*S*)-**L6**	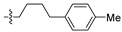			78 ± 15	3.34	2.0
(*R*)-**L6**	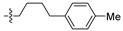			138 ± 30	3.34	2.0
**L7**	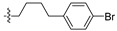	27 ± 5	2.5 ± 0.5		3.90	1.7
**L8**	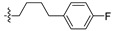	28 ± 6	3.7 ± 0.4		3.17	2.0
**L9**	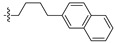	23 ± 6	7 ± 2		4.53	1.7
**L11**	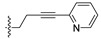	493 ± 100	97 ± 20		3.20	3.7
**L12**	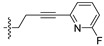	584 ± 100	129 ± 20		3.86	3.0
**L13**	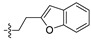	120 ± 24		1674 ± 300 ^g^	2.59	3.7
**L14**	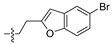	66 ± 14			4.41	2.5
**L15**	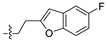	54 ± 14		2213 ± 400 ^g^	3.68	2.5
**L16**	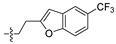	49 ± 12		288 ± 50 ^g^	4.39	2.5
**L17**	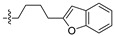	44 ±12			3.14	2.7
**L18**	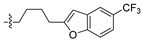	19 ± 4			3.67	2.5
**L19**	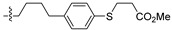	42 ± 9			3.23	2.9

^a^ Assays were performed by the Psychoactive Drug Screening Program with cells overexpressing GluN2B (see Section 3.4.1). ^b^ From in-house assay (see Experimental Information). Ro 25-6981 gave *K*_i_ values between 10 and 26 nM in the in-house assay. ^c^ Calculated from Pallas software. ^d^ Calculated according to the literature [17]. ^e^ First reported in Cai et al. [24]. ^f^ Measured to be 3.41 [25]. ^g^ Ro 25-6981 had a *K*_i_ of 13 ± 4 nM in the same assay session. All assay results are mean ± SD for *n* ≥ 3.

## Data Availability

The original contributions presented in this study are included in the article/Supplementary Material. Further inquiries can be directed to the corresponding author(s).

## Supplementary Materials

The following supplementary information can be downloaded at: https://www.mdpi.com/article/10.3390/molecules31091541/s1: syntheses of acohols, Tables S1 and S2, NMR spectra; HPLC general methods; analytical and preparative HPLC chromatograms, including chiral HPLC chromatograms (Figures S1–S122; ligand physicochemical properties used in CNS PET MPO scoring; docking of ifenprodil and (*R*)-**L19** and (S)-**L19**; and [38,39,40,41,42,43,44].

## Abbreviations

DCM, dichloromethane; DMF, *N,N*′-dimethylformamide; DMSO, dimethyl sulfoxide; MW, microwave; NIMH, National Institute of Mental Health; NMDA, *N*-methyl-D-aspartate; PDSP, psychoactive drug screening program; RT, room temperature; THF, tetrahydrofuran; TFA, trifluoroacetic acid; VCD/IR, vibrational circular dichroism/infrared; USP, United States Pharmacopeia.
