# Supplementary material for: A Study of 3-Substituted 7-Methoxy-2,3,4,5-tetrahydro-1H-benzo[d]azepin-1-ols Leading to Candidate PET Radioligands for Imaging Brain GluN2B: Design, Synthesis, and Structure–Activity Relationships
Source: Molecules. 2026 May 6;31(9):1541. doi: 10.3390/molecules31091541 (PMC13164588; doi:10.3390/molecules31091541)
Supplement: Supplementary file 1 [file molecules-31-01541-s001.zip › molecules-4273563-supplementary.pdf]

# Supplementary Information

# A Study of 3-Substituted 7-Methoxy-2,3,4,5-tetrahydro-1*H*-benzo[*d*]azepin-1-ols Leading to Candidate PET Radioligands for Imaging Brain GluN2B: Design, Synthesis, and Structure–Activity Relationships

Lisheng Cai [1],* Leah Noelle Millard [1], Sean Wallace Costner [1], Alyssa Wang [1], Yonglan Liu [2] and Victor William Pike [1]

1 PET Radiopharmaceutical Sciences Section, Molecular Imaging Branch, National Institute of Mental Health, National Institutes of Health, Bethesda, MD 20892, USA; leah.millard@nih.gov (L.N.M.); seancostner1@gmail.com (S.W.C.); ajw150@georgetown.edu (A.W.); pikev@mail.nih.gov (V.W.P.)

2 Bioinformatics and Computational Biosciences Branch, National Institute of Allergy and Infectious Diseases, National Institutes of Health, Bethesda, MD 20892, USA; yonglan.liu2@nih.gov

* Correspondence: lishengcai@mail.nih.gov; Tel.: +1-(301)-451-3905; Fax: +1-(301)-480-5112

*Correspondence to: Dr. Lisheng Cai, Molecular Imaging Branch, National Institute of Mental Health, National Institutes of Health, 10 Center Drive, Building 10, Room B3 C346, Bethesda, MD 20892, USA. Tel. (301) 451-3905; Fax (301) 480-5112; E-mail: LishengCai@mail.nih.gov.

**Table of Contents**


1. Syntheses of Alcohols S3
2. NMR Spectra of Tosylates **T1–T19** S9
3. General HPLC Methods S45
4. Analytical HPLC Chromatograms of Ligands **L1–L10** and **L11–L21** S48
5. NMR Spectra of Ligands **L1–L9** and **L11–L21** S67
6. Chiral Chromatograms for Ligands **L2**, **L3**, **L6**, **L20**, and **L21** S105
7. Determination of Absolute Configuration for **L3** (NR2B-SMe) Enantiomers S115
8. Radiochromatograms for Radiotracer Purification and Analyses S118
9. Physicochemical Parameters and CNS PET MPO Scores for Ligands **L1–L20** S114
10. GluN2B Ligand Binding Affinity Versus Computed Lipophilicity (cLog*D*) S121
11. Binding of Ligands to σ Receptors S122
12. Docking of (1*R*,2*S*)-Ifenprodil with the GluN1a/GluN2B Complex S123
13. Docking of (*R*)-**L19** *and (S)*-**L19** with the GluN1a/GluN2B Complex S124
14. References S126

## 1. Syntheses of Alcohols

**4-(4-Iodophenyl)butan-1-ol (1)** [1]. 4-(*p*-Iodophenyl)butyric acid (11.87 g, 40.9 mmol) was dissolved in THF (100 mL) from a SealSure bottle (Sigma Aldrich, Burlington, MA, USA). The solution was stirred while $BH_3$·THF (1 M, 100 mL) was added slowly, resulting in gas release. The addition was controlled and the reaction temperature was maintained below 40 °C. A clear, colorless solution formed after 5 min. Reversed phase HPLC (general method A) showed complete conversion of the starting acid to the alcohol. Extending the reaction time to 72 h showed no observable change. Methanol (100 mL) was then added slowly to quench any remaining borane, resulting in hydrogen gas evolution. Once gas evolution had ceased, water (100 mL) was added gradually; no additional gas or heat was generated. The reaction mixture was then stirred at RT for 1 h. A saturated solution of ammonium chloride (100 mL) was then added. The product was extracted with DCM (3 × 100 mL) and the combined organic layers were dried ($MgSO_4$) and the solvent removed under vacuum. The resultant oil was redissolved in acetonitrile, transferred, and then dried with Centrifan to give **1** as a pale yellow oil (14.87 g, yield 86%). $^1$H-NMR ($CDCl_3$): $\delta$ 7.59 (d, 2H, $^3J_{HH}$ = 12 Hz, Ar-*H*), 6.93 (d, 2H, $^3J_{HH}$ = 8.0 Hz, Ar-*H*), 3.64 (t, 2H, $^3J_{HH}$ = 6.0 Hz, C*H₂*OH), 2.58 (t, 2H, $^3J_{HH}$ = 8.0 Hz, C*H₂*OH), 1.68 (m, 2H, C*H₂*), 1.59 (m, 2H, C*H₂*). $^{13}$C-NMR ($CDCl_3$): $\delta$ 142.07 ($C_{Ar}$), 137.44 ($C_{Ar}$), 130.69 ($C_{Ar}$), 90.90 ($C_{Ar}$-I), 62.71 ($C$H$_2$OH), 36.24 ($C$H$_2$), 32.26 ($C$H$_2$), 27.49 ($C$H$_2$).

**4-(4-Methylthiophenyl)butan-1-ol (2)** [2]. 4-(*p*-Iodophenyl)butan-1-ol (**1**; 2.01 g, 7.3 mmol), NaSMe (0.60 g, 8.6 mmol), $Me_3SnCl$ (1.67 g, 8.4 mmol), and [1,1′-*bis*(diphenylphosphino)ferrocene]dichloropalladium(II)·$CH_2Cl_2$ (0.82 g, 1.1 mmol) were dissolved in DMSO (10 mL) in a 35-mL microwave tube. The reaction mixture was then heated in a microwave reactor (110 °C, 30 min, 50 W, 250 psi). Reversed phase HPLC (general method

A) showed complete conversion. The reaction mixture was then dissolved in DCM (200 mL) and washed with water. The organic layer was then dried over $MgSO_4$, filtered, and then added to silica gel (200 mesh, 50 mL). The solvent was removed from the silica gel slurry under vacuum and the reaction mixture was purified (Combi-Flash; silica, hexanes/ethyl acetate). LC-MS analysis confirmed product isolation. The product fractions were dried under vacuum. The resulting residue was then redissolved in acetonitrile, and passed through a 0.2 μm syringe filter, and then dried with Centrifan to give **2** as a brown oil (0.69 g, yield 48%). $^1$H-NMR ($CDCl_3$): $\delta$ 7.20 (d, 2H, $^3J_{HH}$ = 8.0 Hz, Ar-*H*), 7.11 (d, 2H, $^3J_{HH}$ = 8.0 Hz, Ar-*H*), 3.66 (t, 2H, $^3J_{HH}$ = 6.0 Hz, C*H*$_2$OH), 2.61 (t, 2H, $^3J_{HH}$ = 8.0 Hz, C*H*$_2$OH), 2.47 (s, 3H, C*H*$_3$), 1.68 (m, 2H, C*H*$_2$), 1.59 (m, 2H, C*H*$_2$). $^{13}$C{$^1$H}-NMR ($CDCl_3$): $\delta$ 139.69 (*C*$_{Ar}$), 135.41 (*C*$_{Ar}$), 129.17 (*C*$_{Ar}$), 127.39 (*C*$_{Ar}$-I), 63.01 (*C*H$_2$OH), 35.28 (*C*H$_2$), 32.47 (*C*H$_2$), 27.72 (*C*H$_2$), 16.59 (S*C*H$_3$). HRMS calcd for $C_{11}H_{17}O_2S$ $[M + OH]^+$: $m/z$ = 213.0952, found 213.0949; error: 1.4 ppm.

**4-(4-(Trifluoromethyl)phenyl)but-3-yn-1-ol (3)** [3]**.** 1-Iodo-4-(trifluoromethyl)benzene (20.0 g, 73.5 mmol), CuI (2.1 g, 11.0 mmol), $Pd(PPh_3)_2Cl_2{\cdot}CH_2Cl_2$ (2.9 g, 3.7 mmol), and but-3-yn-1-ol (10.3 g, 147.1 mmol) were added to triethylamine (300 mL) under nitrogen. The mixture was heated at 60 °C for 16 h. Reversed phase HPLC (general method B) showed near complete consumption of the starting material. The reaction solution was cooled to RT, ethyl acetate (550 mL) was added, stirred for 20 min, and then filtered. The filter cake was then washed with ethyl acetate (200 mL). The combined ethyl acetate washings were then dried under vacuum to give a black oil, which was purified (Combi-Flash; silica gel, petroleum ether/ethyl acetate, from 50: 1 to 3: 1 v/v) to give **3** as a light-yellow liquid (9.7 g, yield 62.0%). $^1$H-NMR ($CDCl_3$): $\delta$ 7.55 (d, 2H, $^3J_{HH}$ = 8.0 Hz, Ar-*H*), 7.50 (d, 2H, $^3J_{HH}$ = 8.0 Hz, Ar-*H*), 3.84 (t, 2H, $^3J_{HH}$ = 6.0 Hz, C*H*$_2$OH), 2.72 (t, 2H, $^3J_{HH}$ = 8.0 Hz, C*H*$_2$CH$_2$OH). $^{13}$C{$^1$H}-NMR ($CDCl_3$): $\delta$ 132.12 (*C*H),

129.92 (q, $^2J_{CF}$ = 32.3 Hz), 127.41, 125.39 (q, $^2J_{CF}$ = 4.0 Hz, *C*H), 124.14 (q, $^1J_{CF}$ = 270 Hz, *C*$F_3$), 89.41 (*C*2), 81.43 (*C*2), 61.22 (*C*$H_2$O), 23.99. HRMS calcd for $C_{11}H_{10}OF_3$ $[M + H]^+$: *m/z* = 215.0684, found 215.0681; error: –0.4 ppm.

**4-(4-(Trifluoromethyl)phenyl)butan-1-ol (4)** [3]**.** Compound **3** (9.7 g, 45.3 mmol) was dissolved in methanol (160 mL) and Pd/C (1.0 g, 10% wt) was added. The reaction flask was then evacuated and filled with hydrogen gas to 1 atmosphere. The reaction mixture was then stirred at 40 °C for 8 h. TLC showed consumption of the starting material. The reaction mixture was then cooled to RT and filtered. After solvent removal under vacuum, the reaction mixture was purified (Combi-Flash; silica gel, hexane/ethyl acetate) to give **4** as a pale yellow liquid (7.7 g, yield 78%). $^1$H-NMR ($CDCl_3$): $\delta$ 7.53 (d, 2H, $^3J_{HH}$ = 8.0 Hz, Ar-*H*), 7.29 (d, 2H, $^3J_{HH}$ = 8.0 Hz, Ar-*H*), 3.67 (t, 2H, $^3J_{HH}$ = 6.0 Hz, C*H*$_2$OH), 2.71 (t, 2H, $^3J_{HH}$ = 6.0 Hz, C*H*$_2$$CH_2$OH), 1.72 (quint, 2H, $^3J_{HH}$ = 6.0 Hz, C*H*$_2$$CH_2$OH), 1.62 (quint, 2H, $^3J_{HH}$ = 6.0 Hz, C*H*$_2$$CH_2$OH). $^{13}$C{$^1$H}-NMR ($CDCl_3$): $\delta$ 146.62, 128.90 (*C*H), 128.38 (q, $^2J_{CF}$ = 33.0 Hz, *C*CF), 127.41, 125.45 (q, $^2J_{CF}$ = 4.0 Hz, *C*HCF), 124.57 (q, $^1J_{CF}$ = 270 Hz, *C*$F_3$), 62.88 (*C*$H_2$O), 35.68, 32.37, 27.52.

**4-(6-Fluoropyridin-2-yl)but-3-yn-1-ol (5)**. 2-Bromo-6-fluoropyridine (6.11 g, 34.7 mmol), but-3-yn-1-ol (2.61g, 37.2 mmol), CuBr (509.6 mg, 3.6 mmol), $Pd(PPh_3)_2Cl_2$ (1.2 g, 1.7 mmol), and $PPh_3$ (0.96 g, 3.7 mmol) were dissolved in $Et_2NH$ (10 mL) resulting in a green solution and white precipitate.  This mixture was stirred at RT for 1 h and then heated at 88 ºC for 24 h. The solution turned brown after stirring at RT for 5 min, forming an off-white precipitate. HPLC showed complete conversion after heating for 3 h, however, heating continued for the full 24 h. The next day, solvent was removed under vacuum and the residue was redissolved in DCM (200 mL). Silica gel (80 mL) was added, the solvent was removed under vacuum, and the reaction mixture was purified (Combi-Flash; silica gel, hexane/ethyl acetate) to give **5** as a colorless oil (0.50 g,

yield 88%). $^1$H-NMR ($CDCl_3$): $\delta$ 7.73 (dd, 1H, $^3J_{HH}$ = 8.0 Hz, Ar-*H*), 7.27 (dd, 1H, $^3J_{HH}$ = 8.0 Hz, $^4J_{HH}$ = 4.0 Hz, Ar-*H*), 6.88 (dd, 1H, $^3J_{HH}$ = 8.0 Hz, $^4J_{HH}$ = 4.0 Hz, Ar-*H*), 3.86 (t, 2H, $^3J_{HH}$ = 6.0 Hz, C*H₂*OH), 2.71 (t, 2H, $^3J_{HH}$ = 8.0 Hz, C*H₂*OH), 3.20 (brs, 1H, OH). $^{13}$C{$^1$H}-NMR ($CDCl_3$): $\delta$ 163.03 (d, $^1J_{CF}$ = 241.5 Hz, Fpy, *C*F), 141.52 (d, $^4J_{CF}$ = 8.0 Hz, Fpy, *C*H), 141.37 (*C*H), 124.55 (d, $^4J_{CF}$ = 4.0 Hz, Fpy, *C*H), 89.31 (*C*2), 80.76 (*C*2), 60.85 (*C*$H_2$O), 23.90. HRMS calcd for $C_9H_9NOF$ $[M + H]^+$: $m/z$ = 166.0668, found 166.0665; error: –1.8 ppm.

**2-(Benzofuran-2-yl)ethan-1-ol (6)** [4,5]. 2-Iodophenol (1.069 g, 4.86 mmol), but-3-yn-1-ol (0.340 g, 4.85 mmol), CuBr (67.8 mg, 0.47 mmol), $(Ph_3P)_2$•$PdCl_2$ (172.4 mg, 0.246 mmol), and $Ph_3P$ (152.7 mg, 0.582 mmol) were dissolved in $Et_2NH$ (10 mL). The reaction mixture was heated at 90 °C for 4 h. Reversed phase HPLC (general method B) showed starting material consumption. The reaction mixture was left to stir overnight. The following morning DCM (200 mL) was added and this was stirred for 5 min. Silica gel (20 mL) was then added. After removal of the solvent under vacuum, the reaction mixture was purified (Combi-Flash, silica gel, ethyl acetate/hexane) to give **6** as a colorless oil (0.531 g, yield 67%). $^1$H-NMR ($CDCl_3$): $\delta$ 7.50 (dd, $^3J_{HH}$ = 8.0 Hz, $^4J_{HH}$ = 2.0 Hz, 1H, Ar-*H*), 7.42 (d, $^3J_{HH}$ = 8.0 Hz, 1H, Ar-*H*), 7.24 (dt, $^3J_{HH}$ = 8.0 Hz, $^4J_{HH}$ = 4.0 Hz, 1H, Ar-*H*), 7.19 (dt, $^3J_{HH}$ = 8.0 Hz, $^4J_{HH}$ = 4.0 Hz, 1H, Ar-*H*), 6.50 (s, 1H, Ar-*H*), 3.98 (t, $^3J_{HH}$ = 6.0 Hz, 2H, C*H₂*O), 3.04 (t, $^3J_{HH}$ = 6.0 Hz, 2H, C*H₂*). $^{13}$C{$^1$H}-NMR ($CDCl_3$): $\delta$ 156.14, 155.01, 128.88, 123.74 (*C*H), 122.84 (*C*H), 120.63 (*C*H), 111.06 (*C*H), 103.89 (*C*H), 60.95 (*C*$H_2$O), 32.26 (*C*$H_2$).

**2-(5-Bromobenzofuran-2-yl)ethan-1-ol (7)** [6]. Use of the method for **6** in same molar proportions to 4-bromo-2-iodophenol (2.12 g, 7.10 mmol) gave **7** as a colorless oil (1.26 g, yield 76%). $^1$H-NMR ($CDCl_3$): $\delta$ 7.61 (s, 1H, Ar-*H*), 7.32 (d, $^3J_{HH}$ = 8.9 Hz, 1H, Ar-*H*), 7.28 (d, $^3J_{HH}$ = 8.6 Hz, 1H, Ar-*H*), 6.45 (s, 1H, Ar-*H*), 3.98 (t, $^3J_{HH}$ = 6.2 Hz, 2H, C*H₂*O), 3.03 (t, $^3J_{HH}$ = 6.2 Hz,

2H, C$H_2$). $^{13}$C{$^1$H}-NMR (CDCl$_3$): $\delta$ 157.74, 153.76, 130.92, 126.58 (*C*H), 123.29 (*C*H), 115.88, 112.49 (*C*H), 103.41 (*C*H), 60.76 (*C*H$_2$O), 32.20 (*C*H$_2$).

**2-(5-Fluorobenzofuran-2-yl)ethan-1-ol (8).** Use of the method for **6** in same molar proportions to 4-fluoro-2-iodophenol (2.29 g, 9.61 mmol) gave **8** as a colorless oil (1.41 g, yield 81%). $^1$H-NMR (CDCl$_3$): $\delta$ 7.33 (dd, $^3J_{HF}$ = 8.0 Hz, $^4J_{HF}$ = 4.0 Hz, 1H, Ar-*H*), 7.14 (dd, $^3J_{HH}$ = 8.0 Hz, $^4J_{HF}$ = 2.0 Hz, 1H, Ar-*H*), 6.94 (dt, $^3J_{HF}$ = $^3J_{HH}$ = 8.0 Hz, $^4J_{HF}$ = 2.0 Hz, 1H, Ar-*H*), 6.47 (s, 1H, Ar-*H*), 3.98 (t, $^3J_{HH}$ = 6.0 Hz, 2H, C$H_2$O), 3.02 (t, $^3J_{HH}$ = 6.0 Hz, 2H, C$H_2$). $^{13}$C{$^1$H}-NMR (CDCl$_3$)$_3$: $\delta$ 159.36 (d, $^1J_{CF}$ = 236.0 Hz, *C*F), 158.18, 151.21, 129.71 (d, C, $J_{CF}$ = 11.0 Hz), 111.55 (d, CH, $J_{CF}$ = 9.0 Hz), 111.23 (d, *C*H, $J_{CF}$ = 26 Hz), 106.20 (d, *C*H, $J_{CF}$ = 25.0 Hz), 104.11 (d, *C*H, $J_{CF}$ = 4.0 Hz), 60.78 (*C*H$_2$O), 32.28 (*C*H$_2$).

**2-(5-(Trifluoromethyl)benzofuran-2-yl)ethan-1-ol (9).** Use of method for **6** in same molar proportions to 2-iodo-4-(trifluoromethyl)phenol (1.06 g, 3.68 mmol) gave **9** as a colorless oil (661 mg, yield 78%). $^1$H-NMR (CDCl$_3$): $\delta$ 7.79 (s, 1H, Ar-*H*), 7.50 (s, 2H, Ar-*H*), 6.59 (s, 1H, Ar-*H*), 4.02 (t, $^3J_{HH}$ = 6.2 Hz, 2H, C$H_2$O), 3.08 (t, $^3J_{HH}$ = 6.2 Hz, 2H, C$H_2$). $^{13}$C{$^1$H}-NMR (CDCl$_3$): $\delta$ 158.34, 156.36, 129.02, 126.25, 125.58 (q, $^2J_{CF}$ = 31.9 Hz, *C*CF$_3$), 123.55, 120.94 (q, $^3J_{CF}$ = 3.6 Hz, *C*HCCF$_3$), 118.30 (q, $^3J_{CF}$ = 3.6 Hz, *C*HCCF$_3$), 111.40 (*C*H), 104.10 (*C*H), 60.74 (*C*H$_2$O), 32.22 (*C*H$_2$).

**4-(Benzofuran-2-yl)butan-1-ol (10)** [7]. 2-Iodophenol (1.363 g, 6.20 mmol), hex-5-yn-1-ol (0.646 g, 6.58 mmol), CuBr (94.5 mg, 0.66 mmol), $(Ph_3P)_2PdCl_2$ (222.3 mg, 0.32 mmol), and $Ph_3P$ (161.6 mg, 0.62 mmol) were dissolved in $Et_2NH$ (10 mL). The reaction mixture was heated at 90 °C for 4 to 5 h, turning a deep red. HPLC (general method B) showed consumption of the starting material. The reaction mixture was stirred overnight. DCM (200 mL) was then added, and this was stirred for 5 min. Silica gel (20 mL) was then added and the solvent was removed

under vacuum. The reaction mixture was purified (Combi-Flash, silica gel, ethyl acetate/hexane) to give **10** as a colorless oil (986 mg, yield 84%). $^{1}$H-NMR ($CDCl_3$): $\delta$ 7.49 (d, $^{3}J_{HH}$ = 7.4 Hz, 1H, Ar-*H*), 7.42 (d, $^{3}J_{HH}$ = 7.6 Hz, 1H, Ar-*H*), 7.23–7.16 (m, 2H, Ar-*H*), 6.40 (s, 1H, Ar-*H*), 3.68 (t, $^{3}J_{HH}$ = 6.4 Hz, 2H, $CH_2O$), 2.81 (t, $^{3}J_{HH}$ = 6.4 Hz, 2H, $CH_2$), 1.86–1.82 (m, 2H, $CH_2$), 1.69–1.59 (m, 2H, $CH_2$). $^{13}$C{$^{1}$H}-NMR ($CDCl_3$): $\delta$ 159.31, 154.81, 129.10, 123.32 (*C*H), 122.60 (*C*H), 120.39 (*C*H), 110.89 (*C*H), 102.23 (*C*H), 62.70 ($CH_2O$), 32.29, 28.33, 24.14.

**4-(5-(Trifluoromethyl)benzofuran-2-yl)butan-1-ol (11).** Use of the method for **10** in same molar proportions to 2-iodo-4-(trifluoromethyl)phenol (1.06 g, 3.68 mmol) gave **11** as a colorless oil (637 mg, yield 67%). $^{1}$H-NMR ($CDCl_3$): $\delta$ 7.76 (vs, 1H, Ar-*H*), 7.47 (vs, 2H, Ar-*H*), 6.46 (s, 1H, Ar-*H*), 3.70 (t, $^{3}J_{HH}$ = 8.0 Hz, 2H, $CH_2O$), 2.83 (t, $^{3}J_{HH}$ = 8.0 Hz, 2H, $CH_2$), 1.89–1.82 (m, 2H, $CH_2$), 1.71–1.51 (m, 2H, $CH_2$). $^{13}$C{$^{1}$H}-NMR ($CDCl_3$): $\delta$ 161.44, 156.22, 129.28, 129.20 (*C*H), 129.10, 125.34 (q, $^{2}J_{CF}$ = 31.7 Hz), 124.97 (q, $^{1}J_{CF}$ = 270 Hz, $CF_3$), 120.56 (q, $^{2}J_{CF}$ = 3.7 Hz, *C*H), 110.05 (q, $^{2}J_{CF}$ = 4.0 Hz, *C*H), 62.66 ($CH_2O$), 32.26, 28.37, 24.06.

## 2. NMR Spectra of Tosylates T1–T18

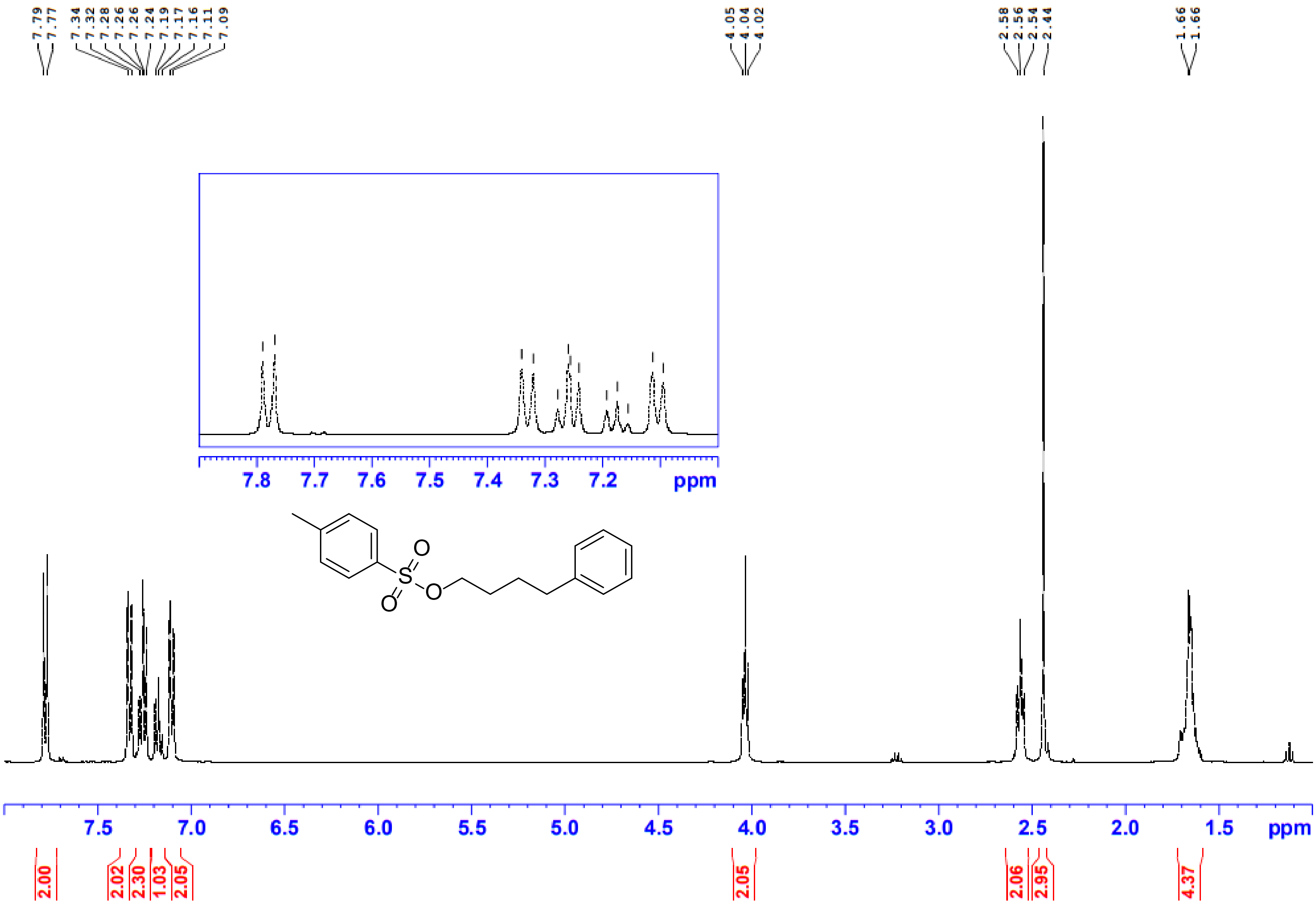


**Supplementary Figure 1.** $^{1}$H NMR Spectrum of **T1** (400 MHz, $CDCl_3$)

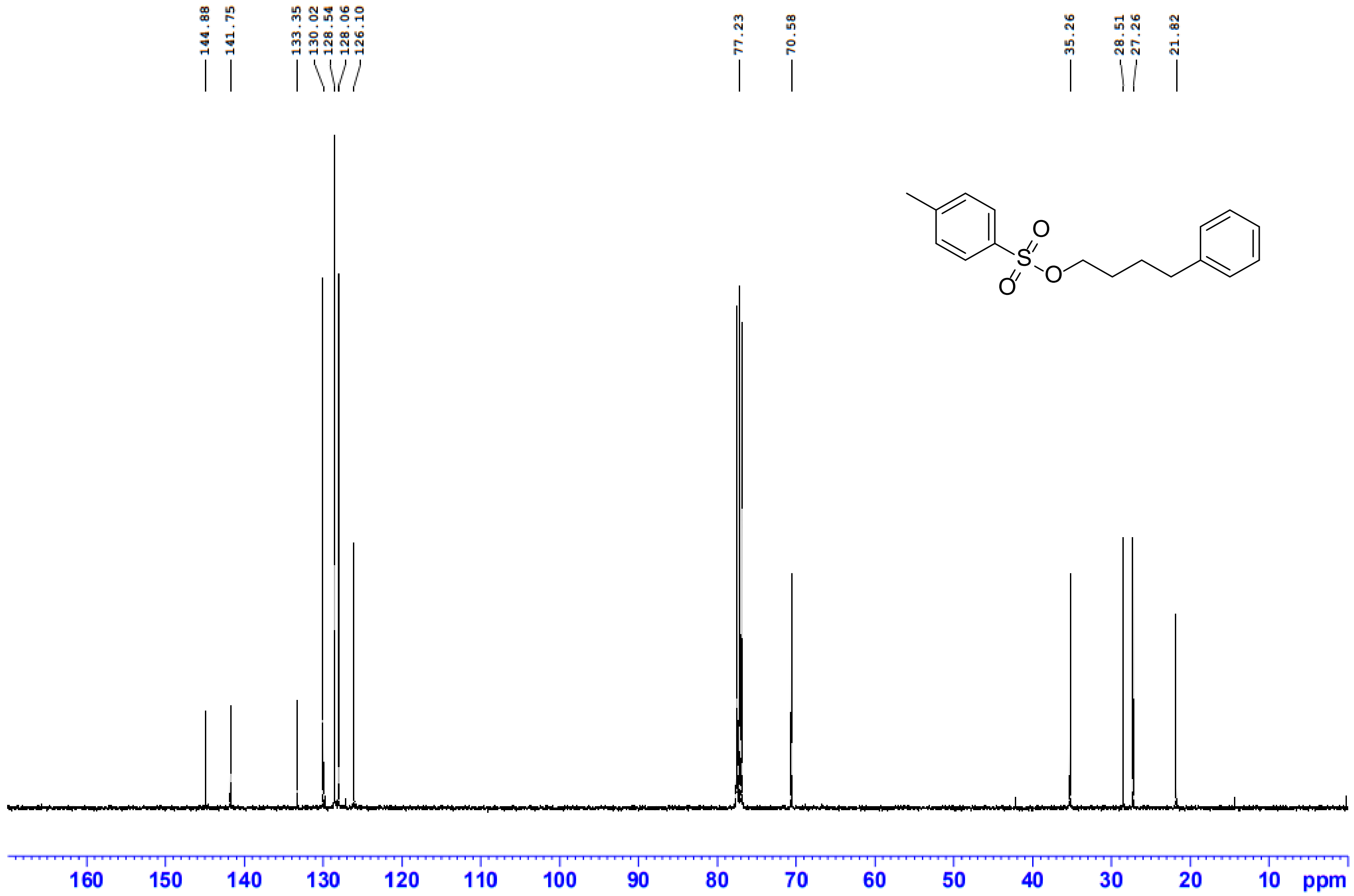


**Supplementary Figure 2.** $^{13}C$ NMR Spectrum of **T1** (101 MHz, $CDCl_3$)

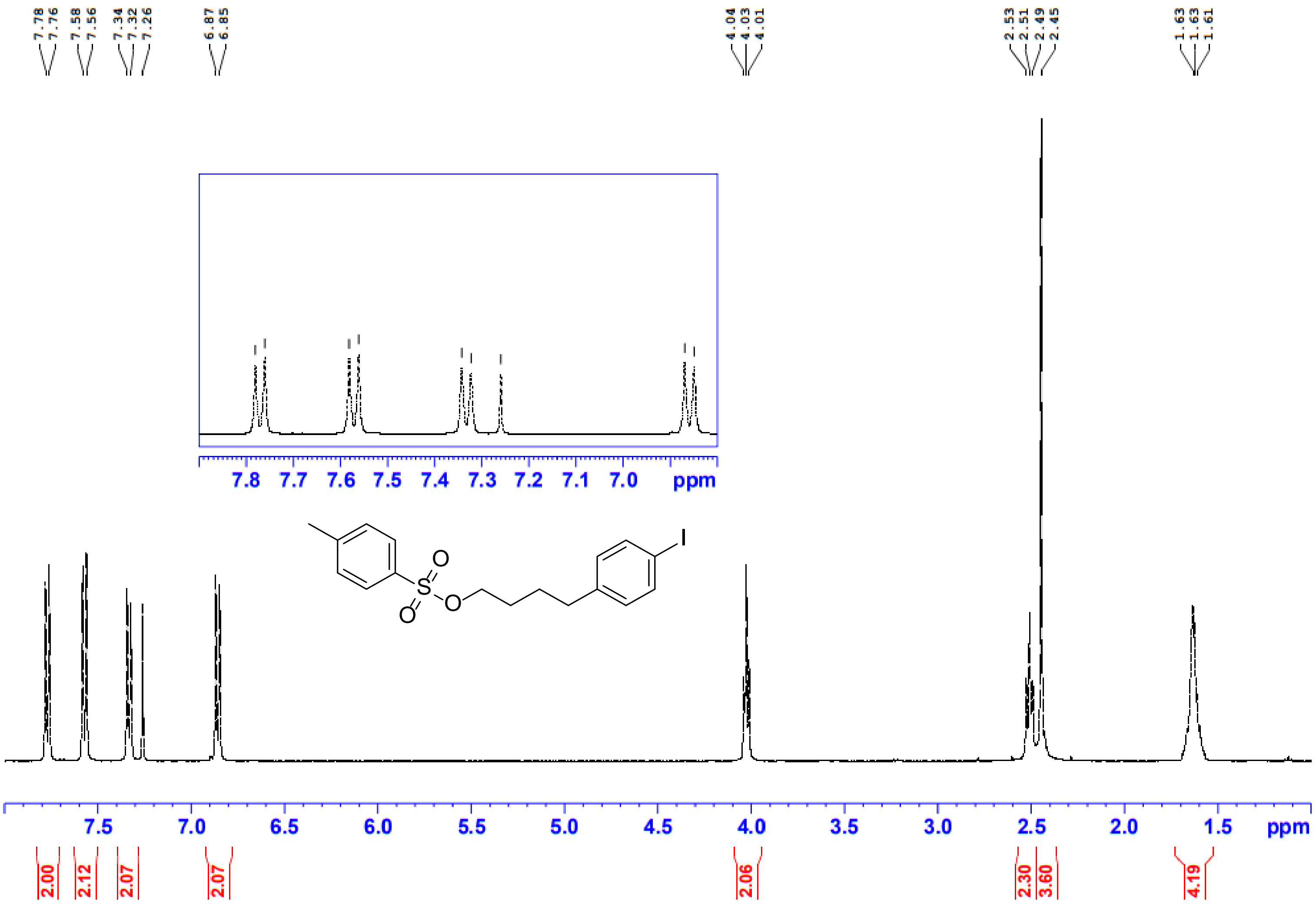


**Supplementary Figure 3.** [1]H NMR Spectrum of **T2** (400 MHz, $CDCl_3$)

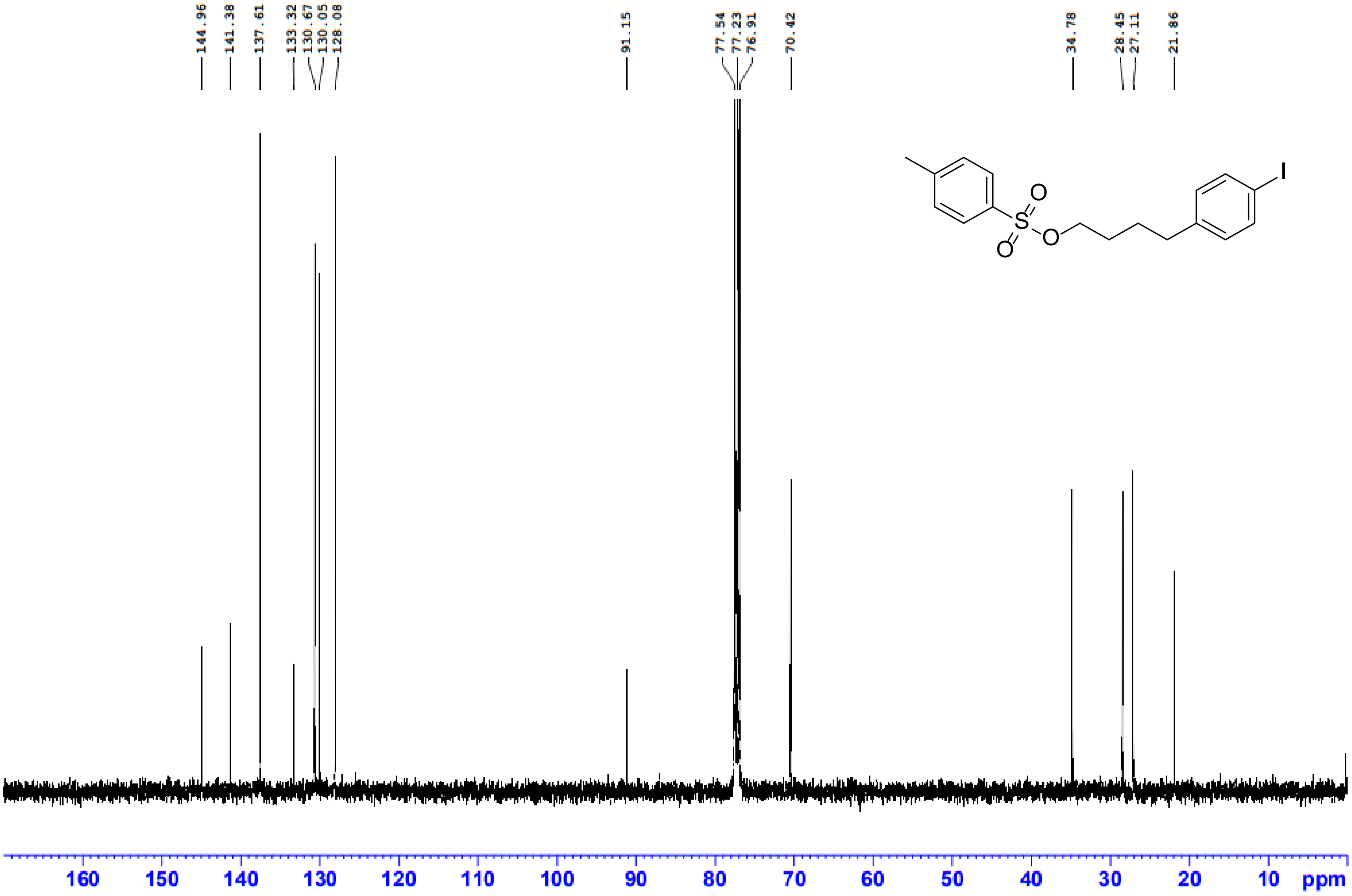


**Supplementary Figure 4.** $^{13}$C NMR Spectrum of **T2** (101 MHz, $CDCl_3$)

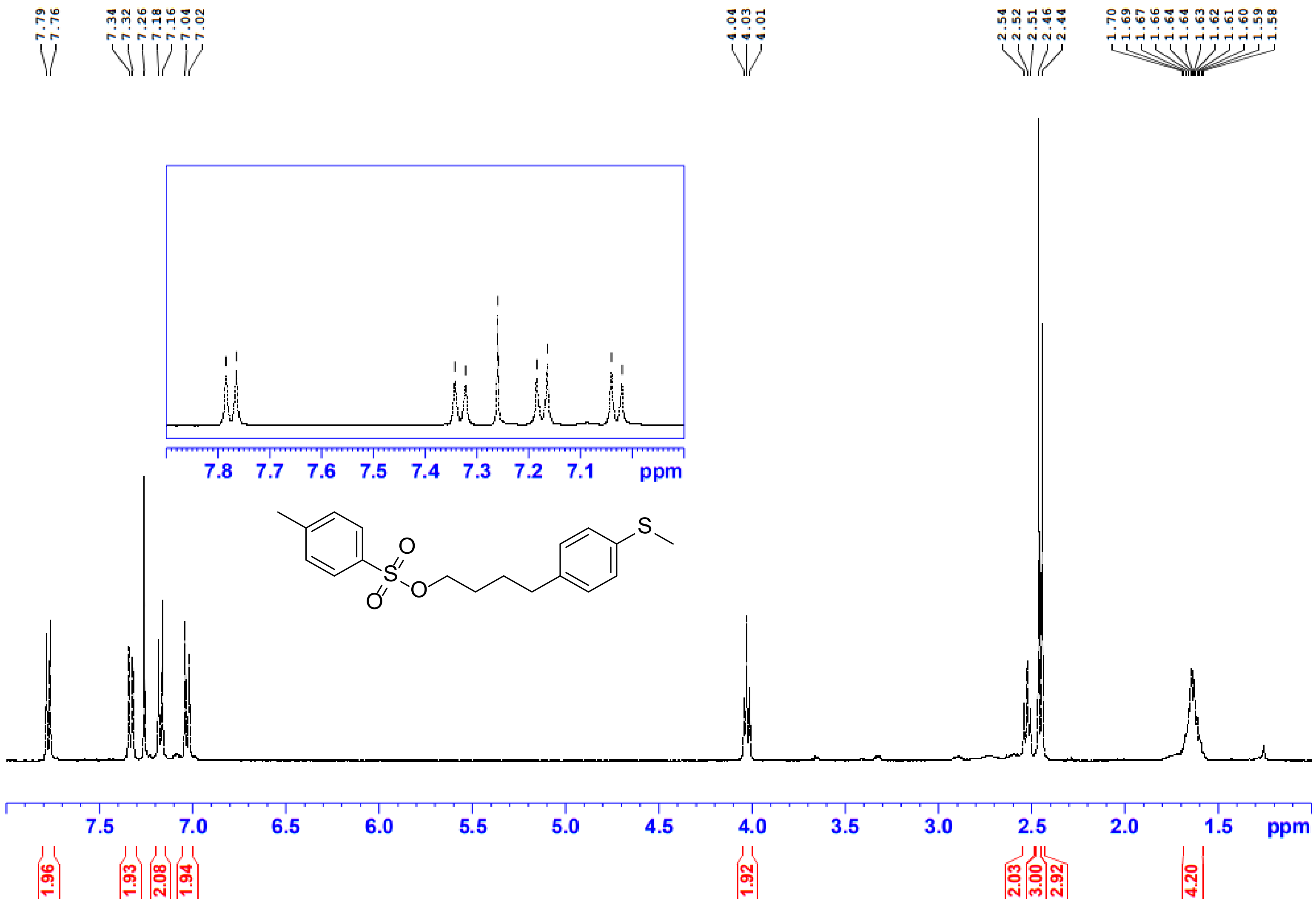


**Supplementary Figure 5.** $^{1}$H NMR Spectrum of **T3** (400 MHz, $CDCl_3$)

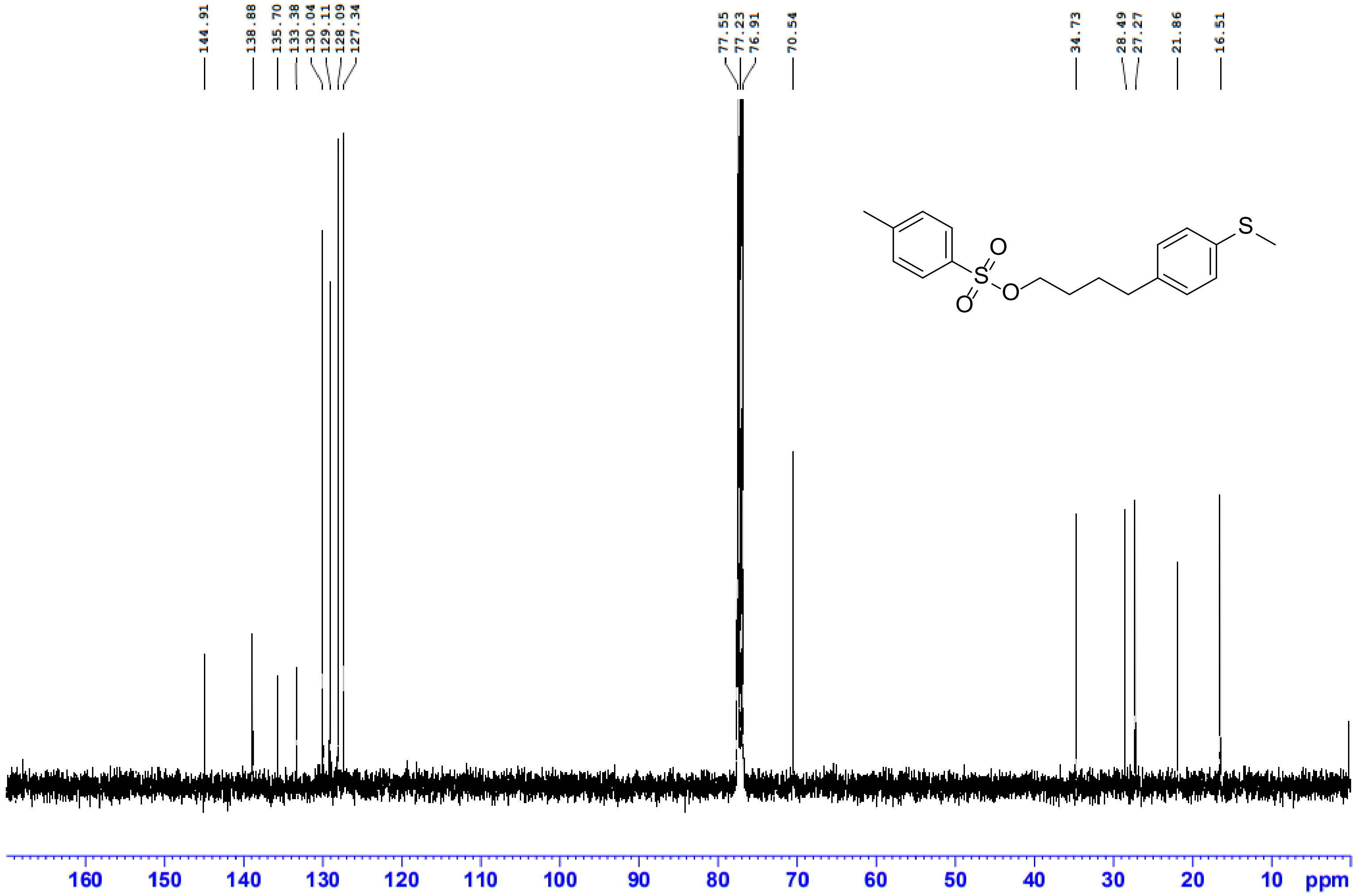


**Supplementary Figure 6.** $^{13}$C NMR Spectrum of **T3** (101 MHz, $CDCl_3$)

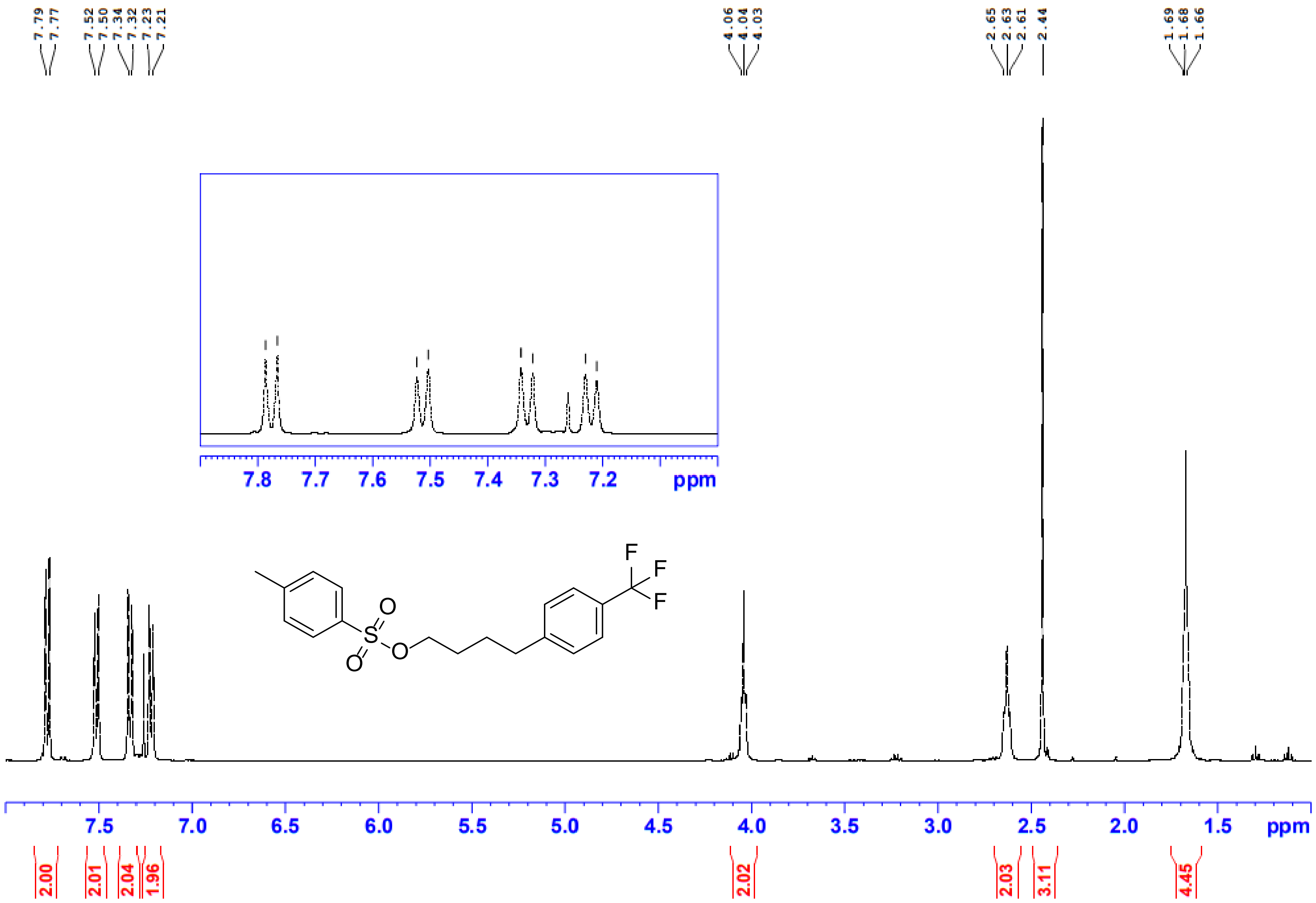


**Supplementary Figure 7.** $^{1}$H NMR Spectrum of **T4** (400 MHz, $CDCl_3$)

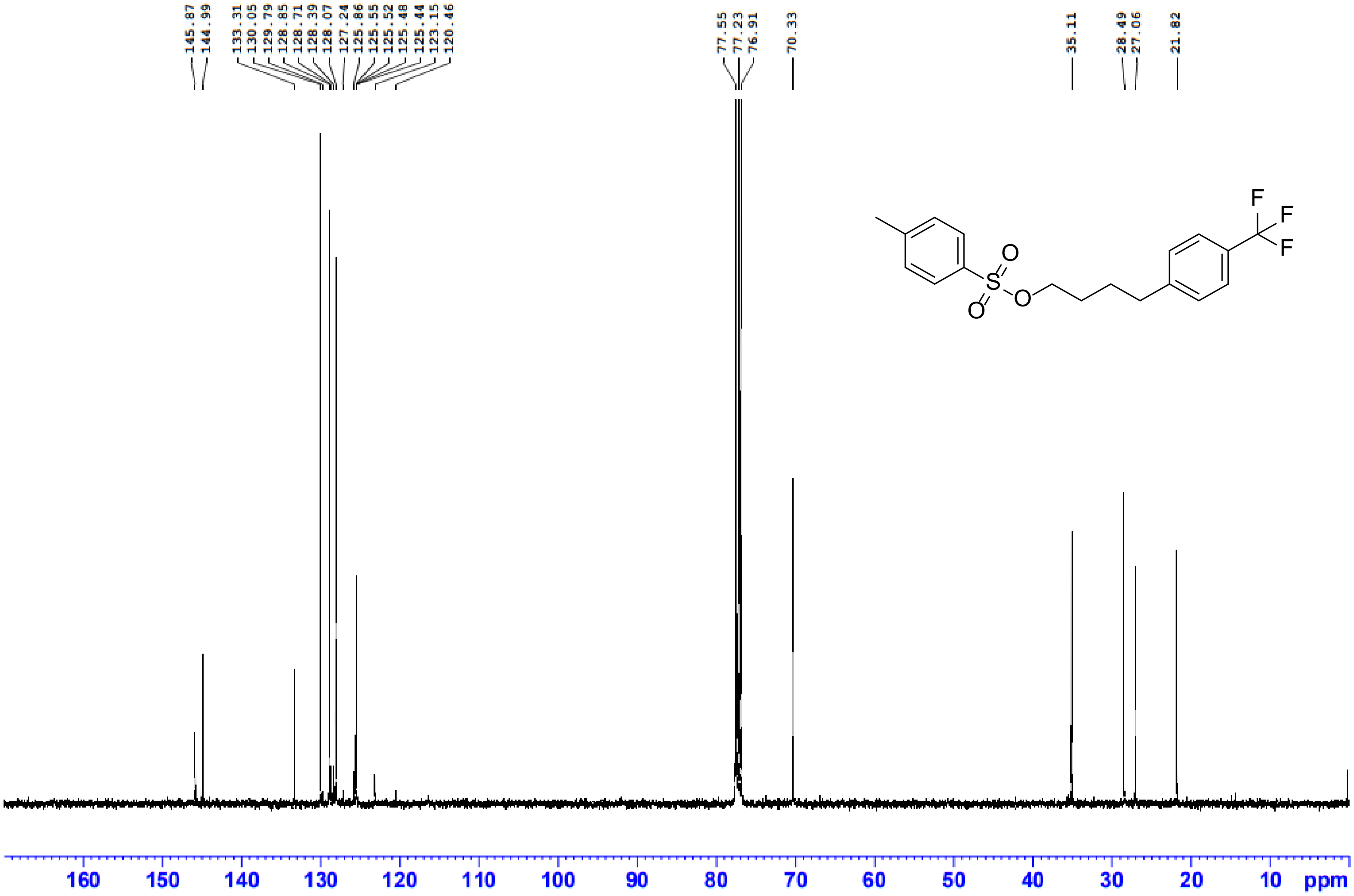


**Supplementary Figure 8.** $^{13}C$ NMR Spectrum of **T4** (101 MHz, $CDCl_3$)

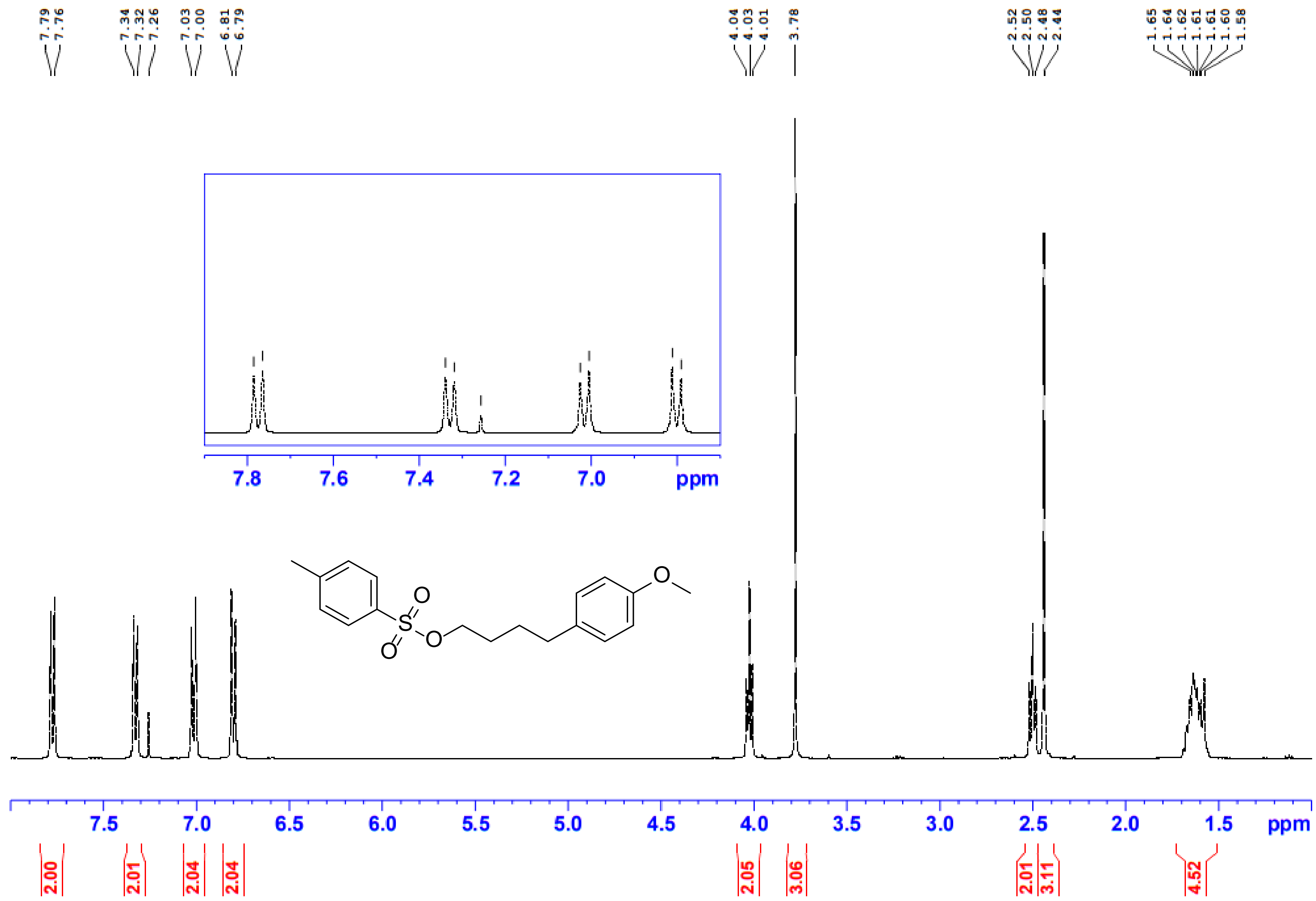


**Supplementary Figure 9.** $^1$H NMR Spectrum of **T5** (400 MHz, $CDCl_3$)

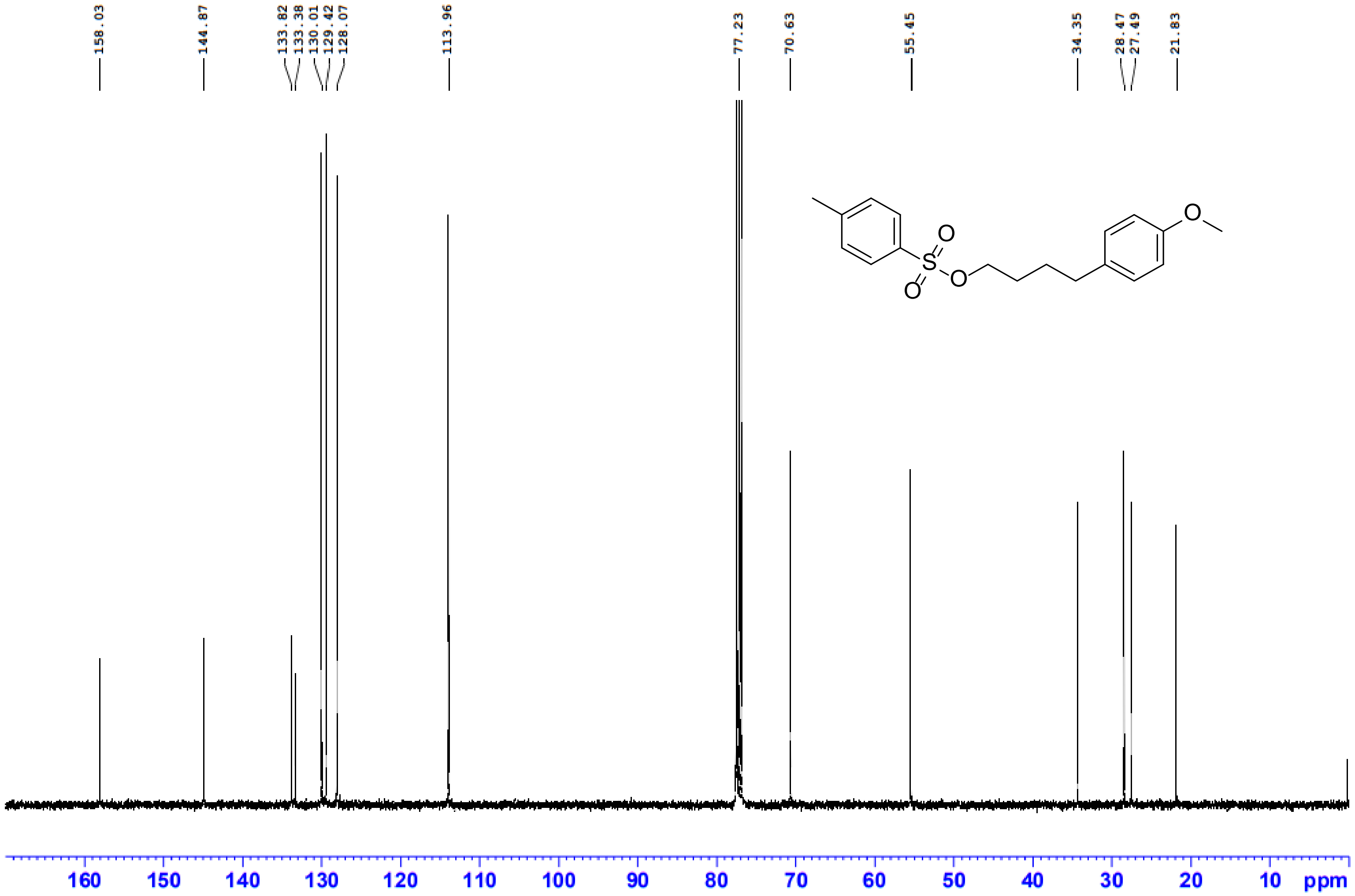


**Supplementary Figure 10.** $^{13}$C NMR Spectrum of **T5** (101 MHz, $CDCl_3$)

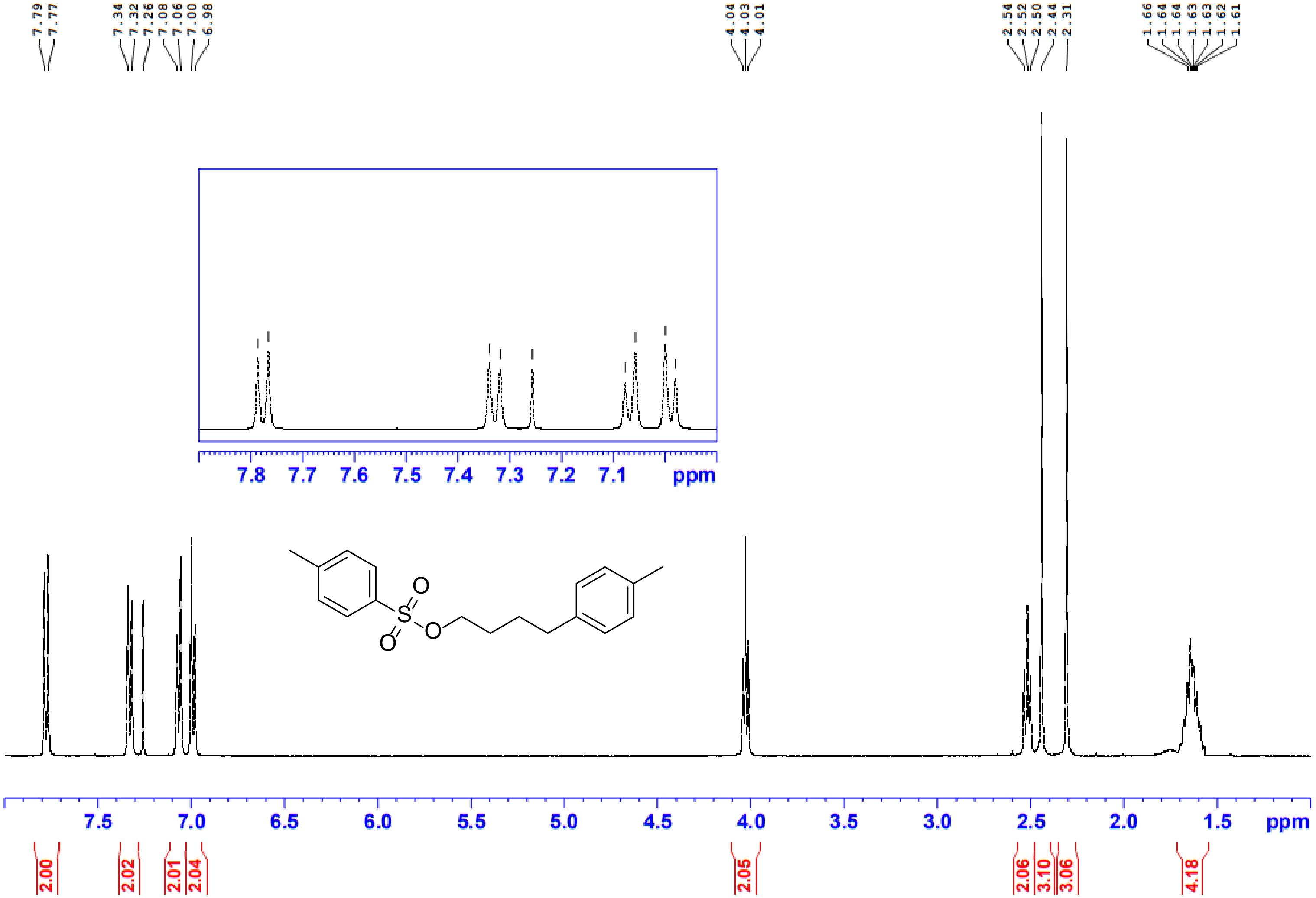


**Supplementary Figure 11.** $^{1}H$ NMR Spectrum of **T6** (400 MHz, $CDCl_3$)

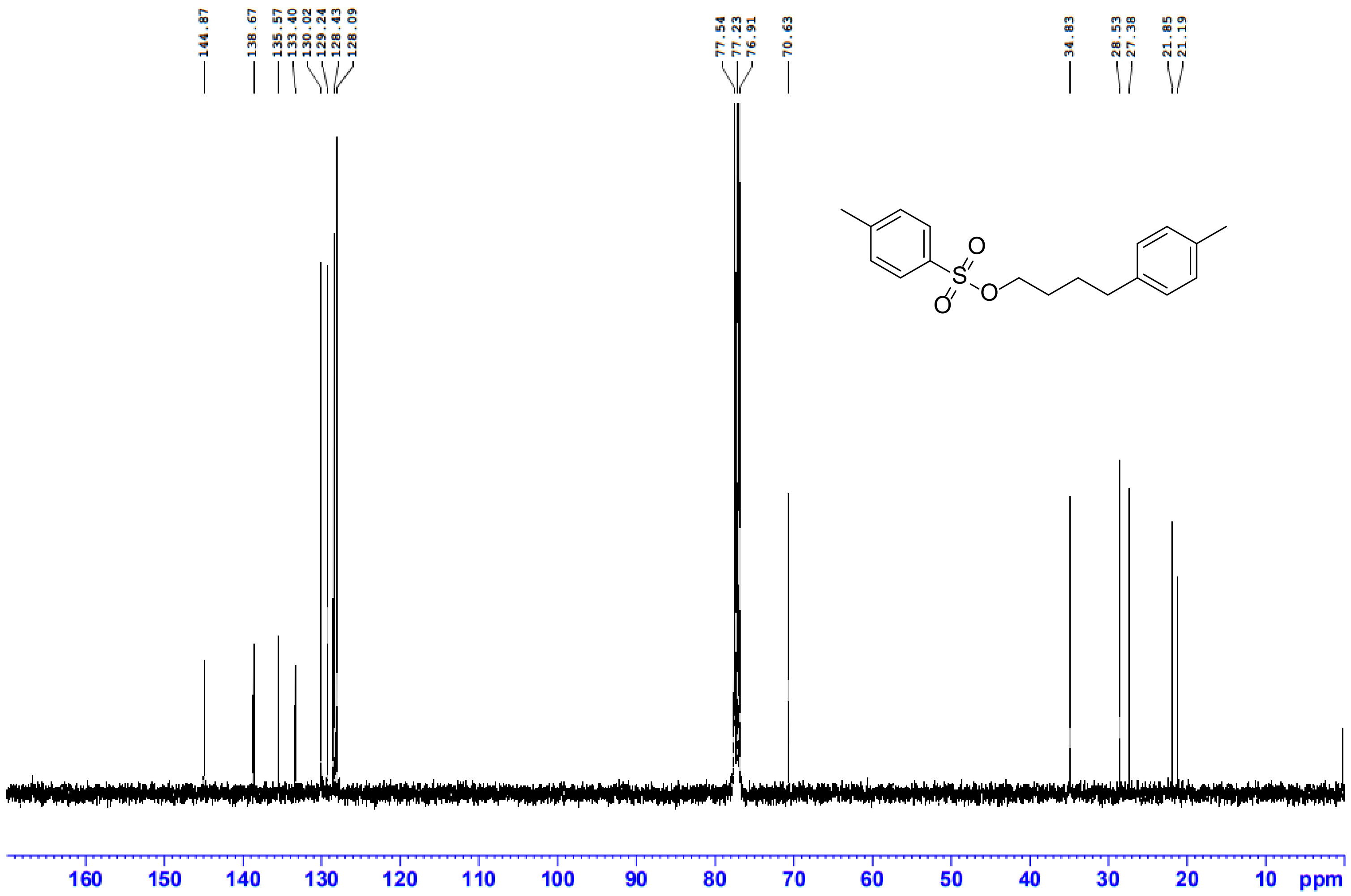


**Supplementary Figure 12.** $^{13}C$ NMR Spectrum of **T6** (101 MHz, $CDCl_3$)

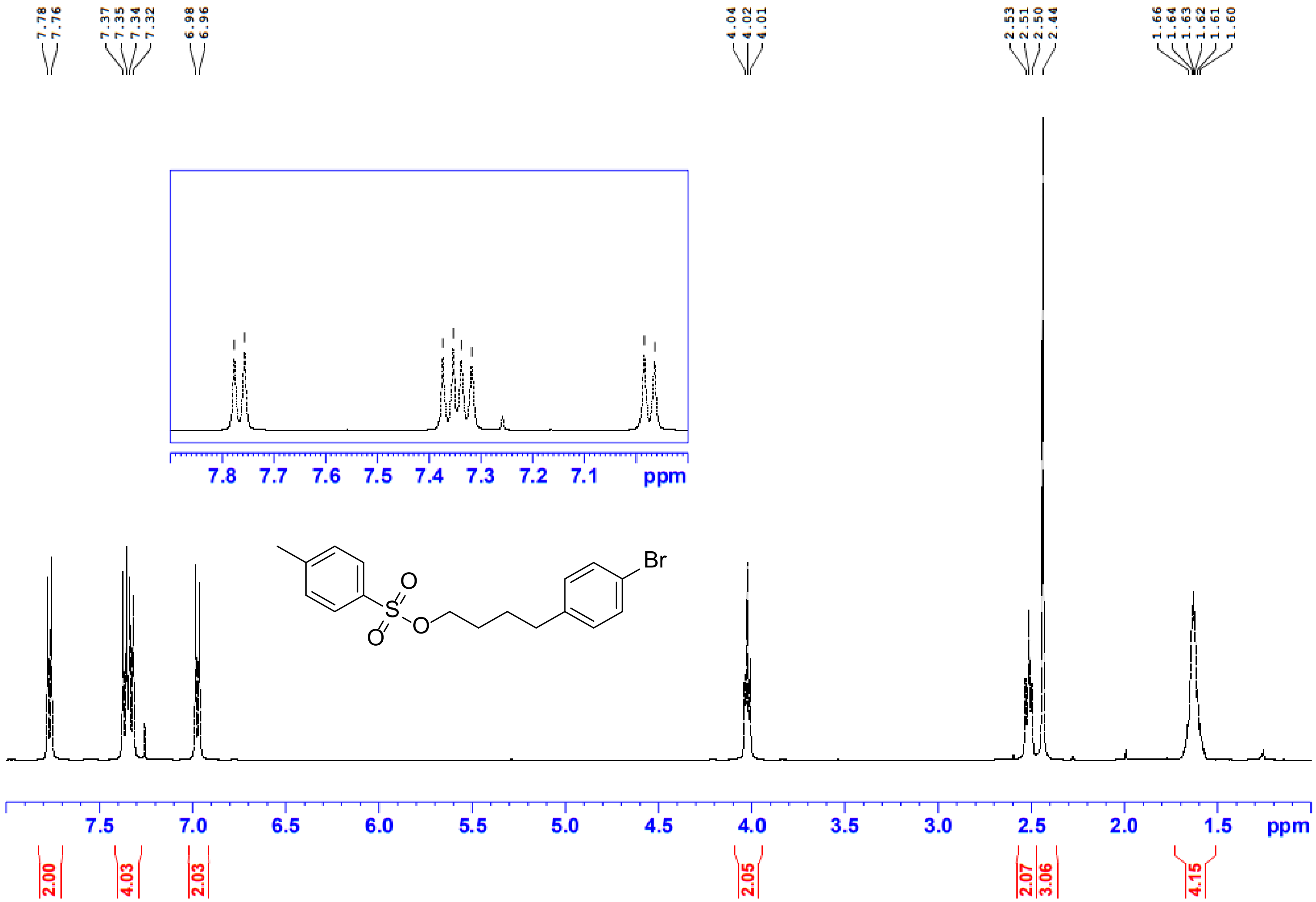


**Supplementary Figure 13.** $^{1}$H NMR Spectrum of **T7** (400 MHz, $CDCl_3$)

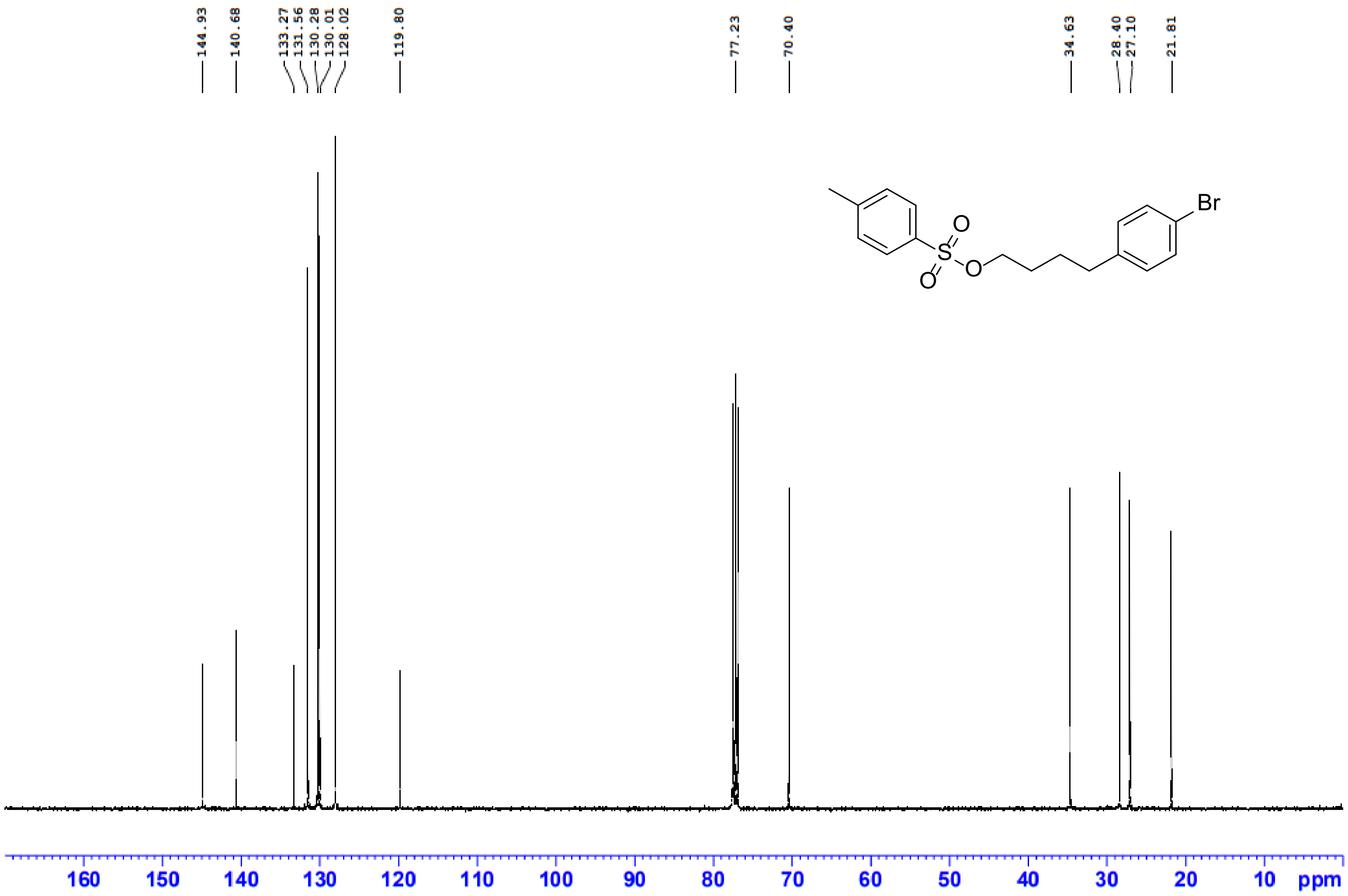


**Supplementary Figure 14.** $^{13}$C NMR Spectrum of **T7** (101 MHz, $CDCl_3$)

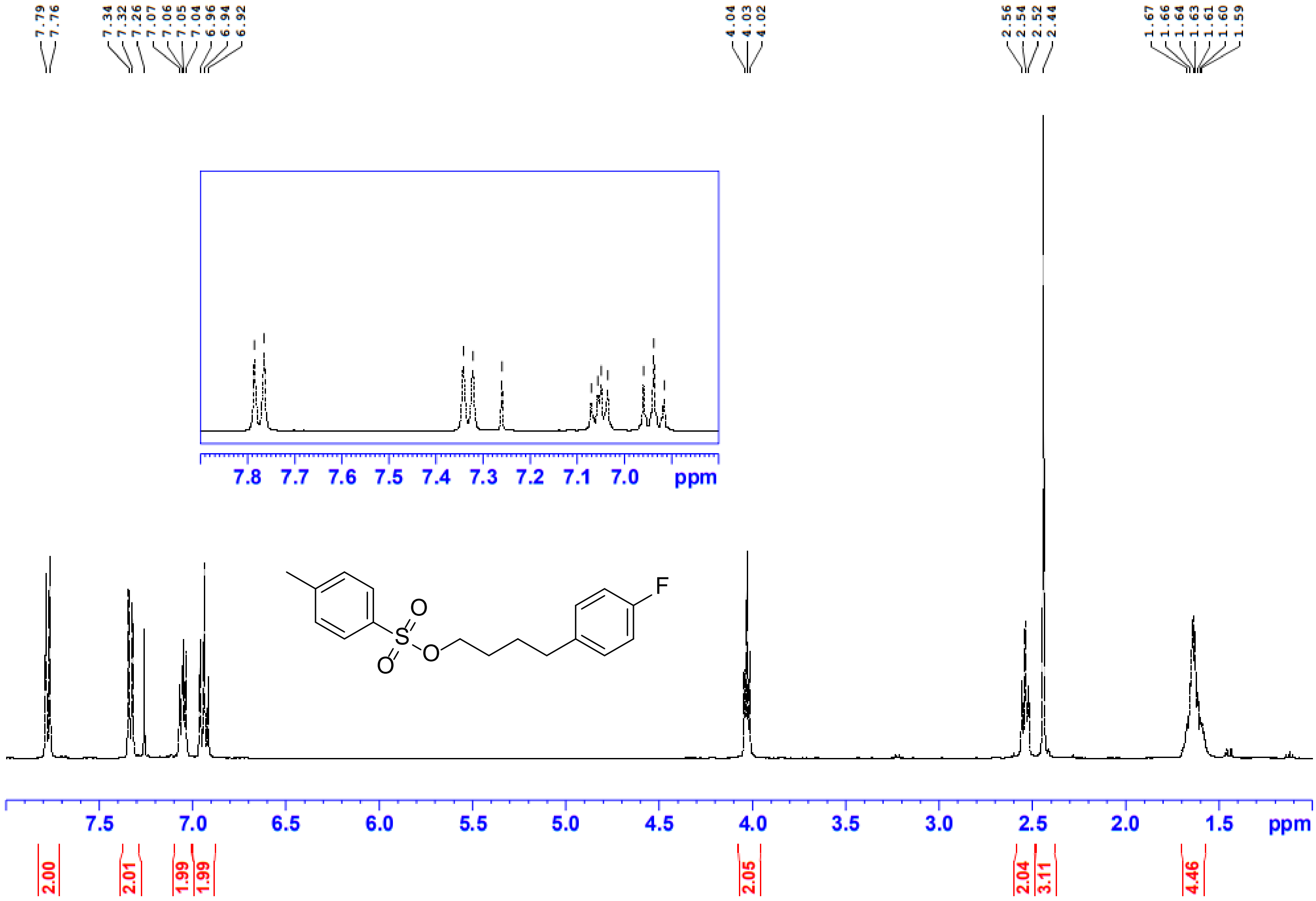


**Supplementary Figure 15.** $^{1}$H NMR Spectrum of **T8** (400 MHz, $CDCl_3$)

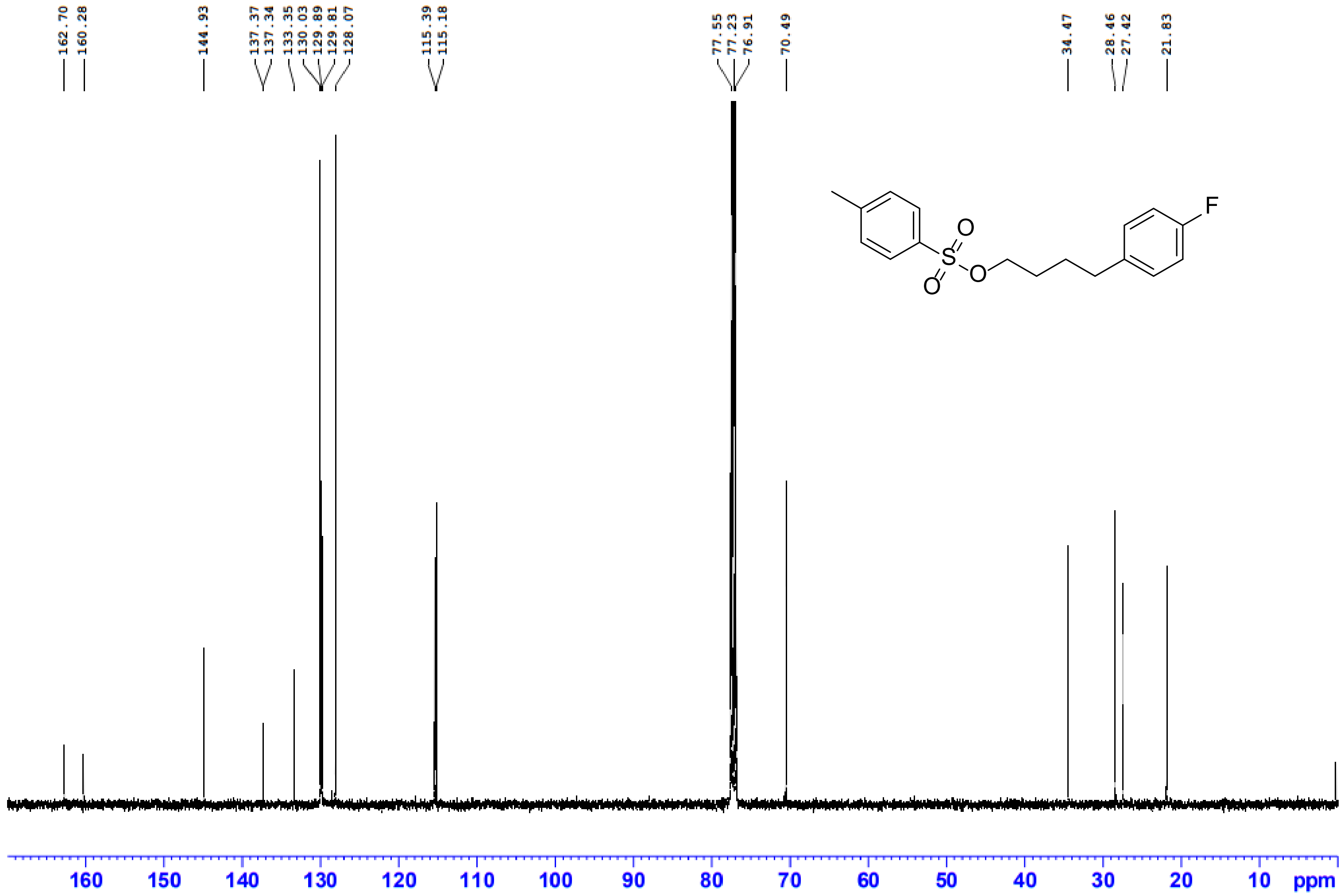


**Supplementary Figure 16.** $^{13}$C NMR Spectrum of **T8** (101 MHz, $CDCl_3$)

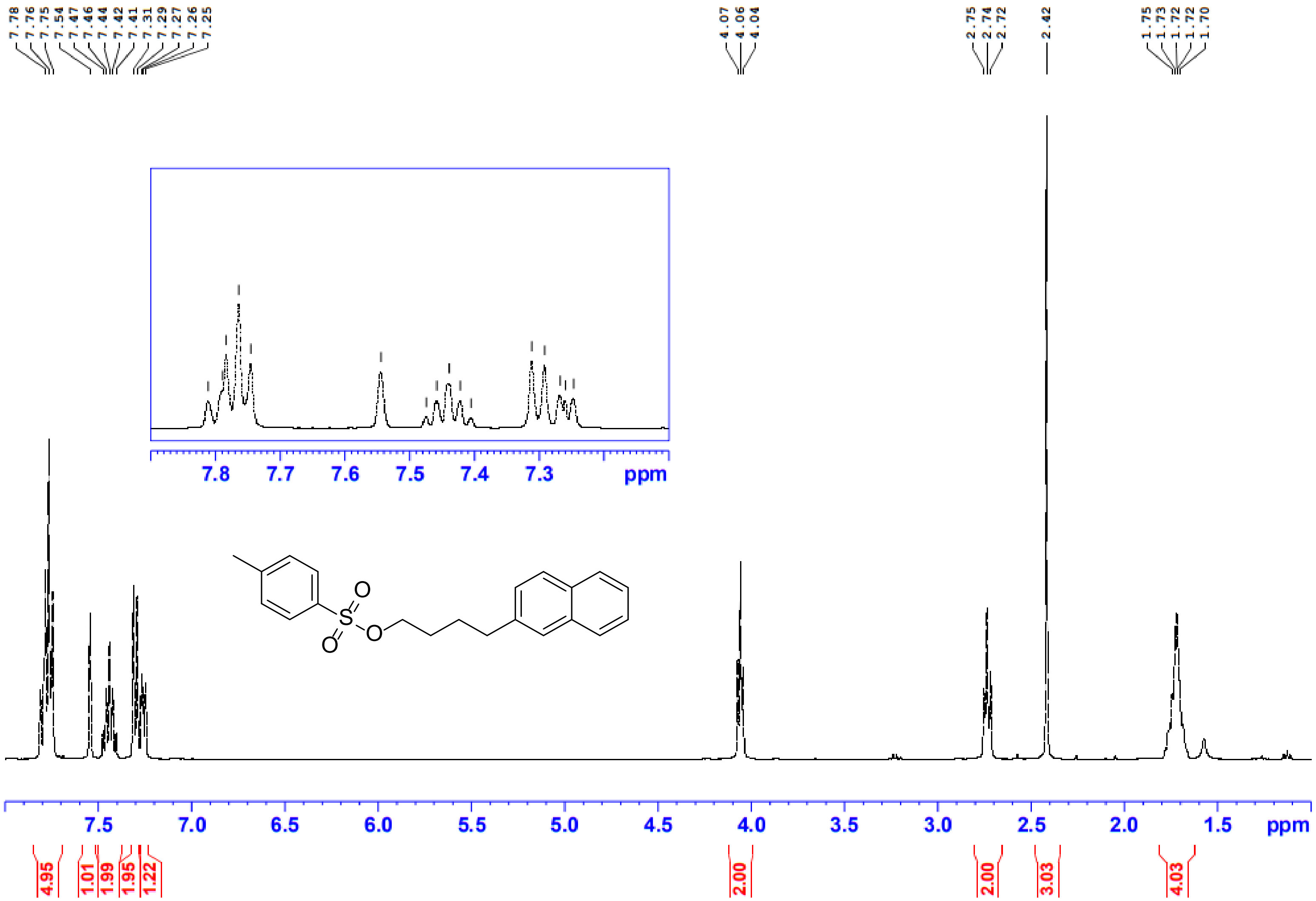

**Supplementary Figure 17.** $^{1}$H NMR Spectrum of **T9** (400 MHz, $CDCl_3$)

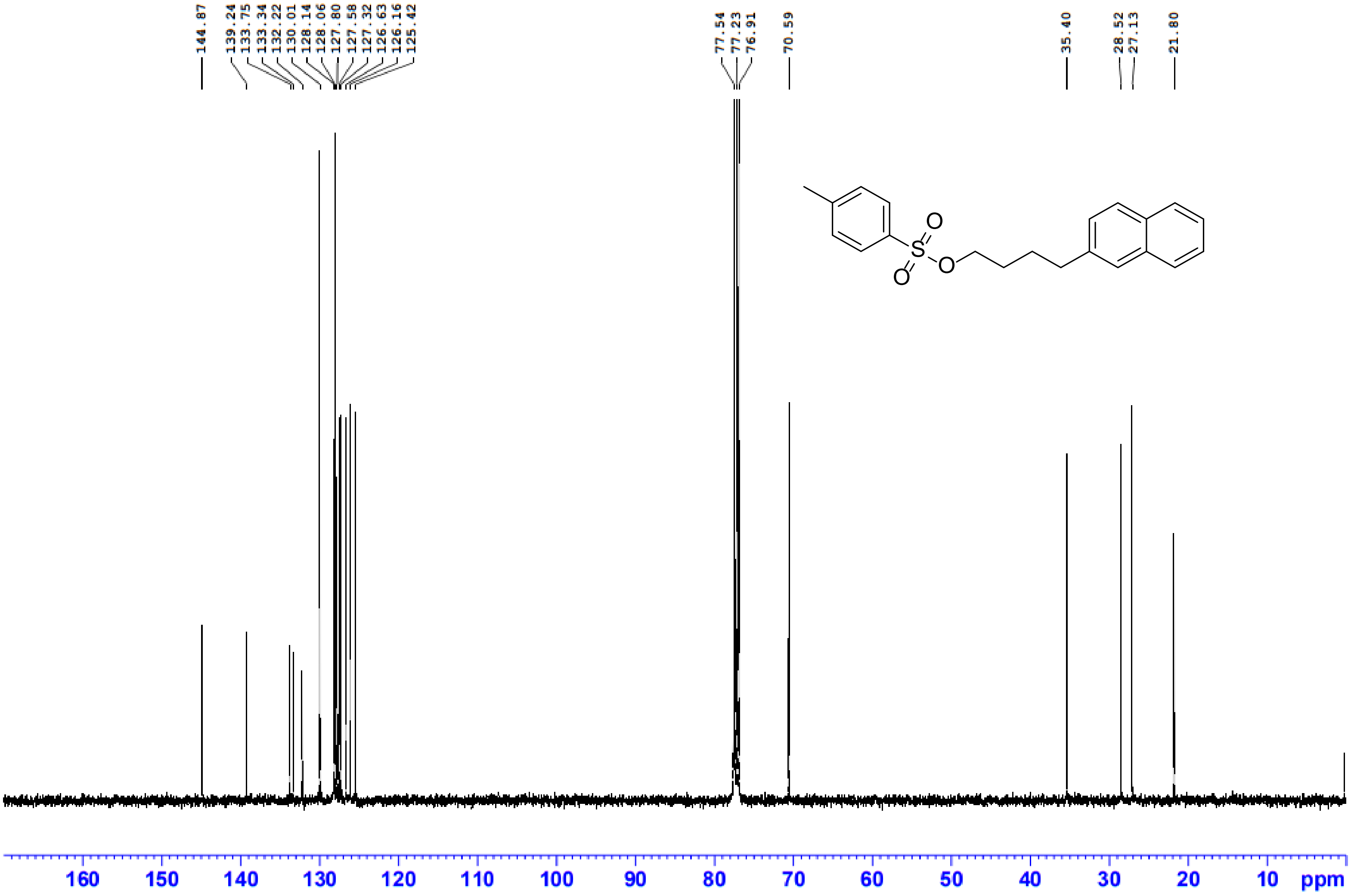


**Supplementary Figure 18.** $^{13}C$ NMR Spectrum of **T9** (101 MHz, $CDCl_3$)

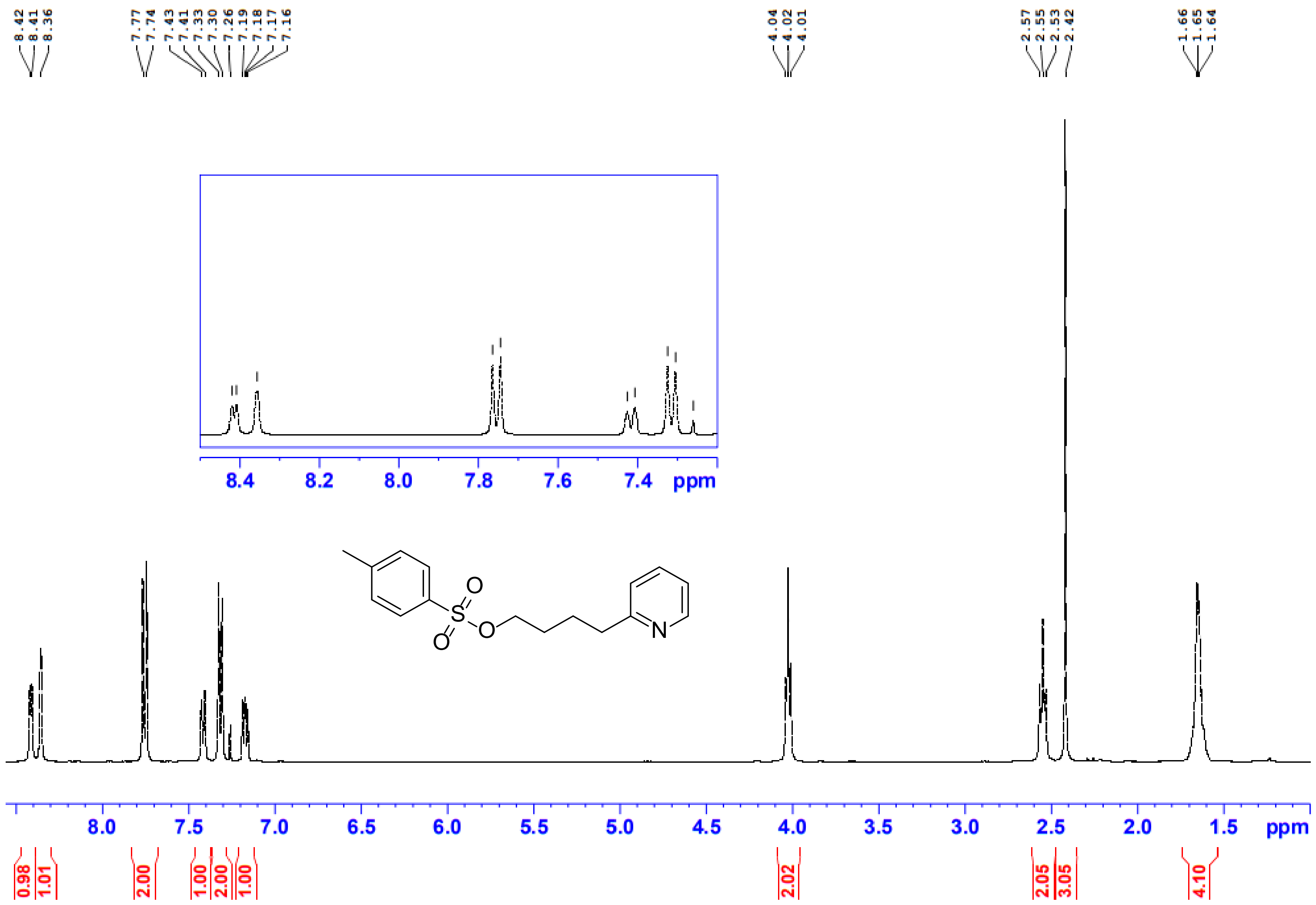


**Supplementary Figure 19.** $^{1}$H NMR Spectrum of **T10** (400 MHz, $CDCl_3$)

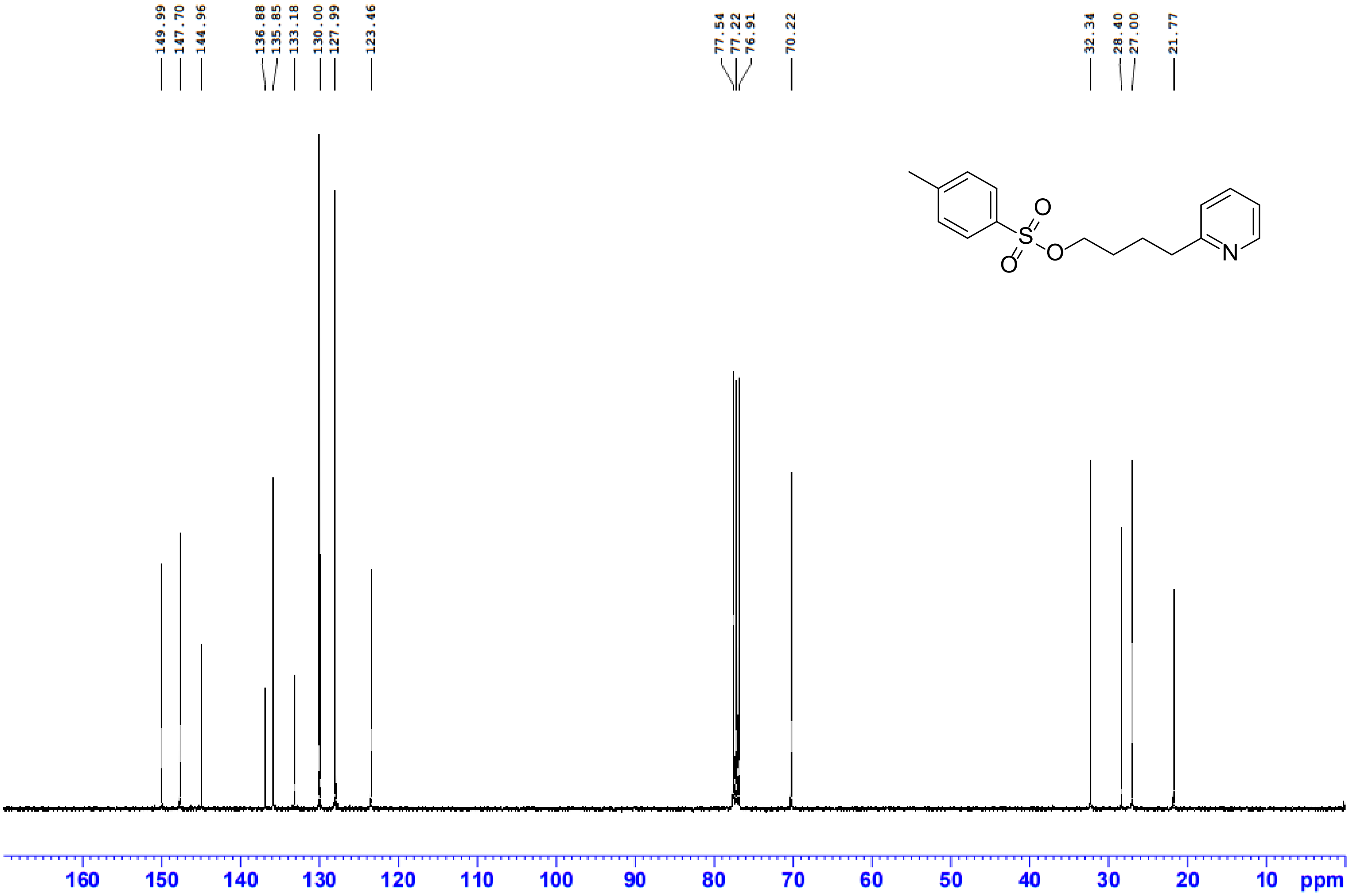


**Supplementary Figure 20.** $^{13}C$ NMR Spectrum of **T10** (101 MHz, $CDCl_3$)

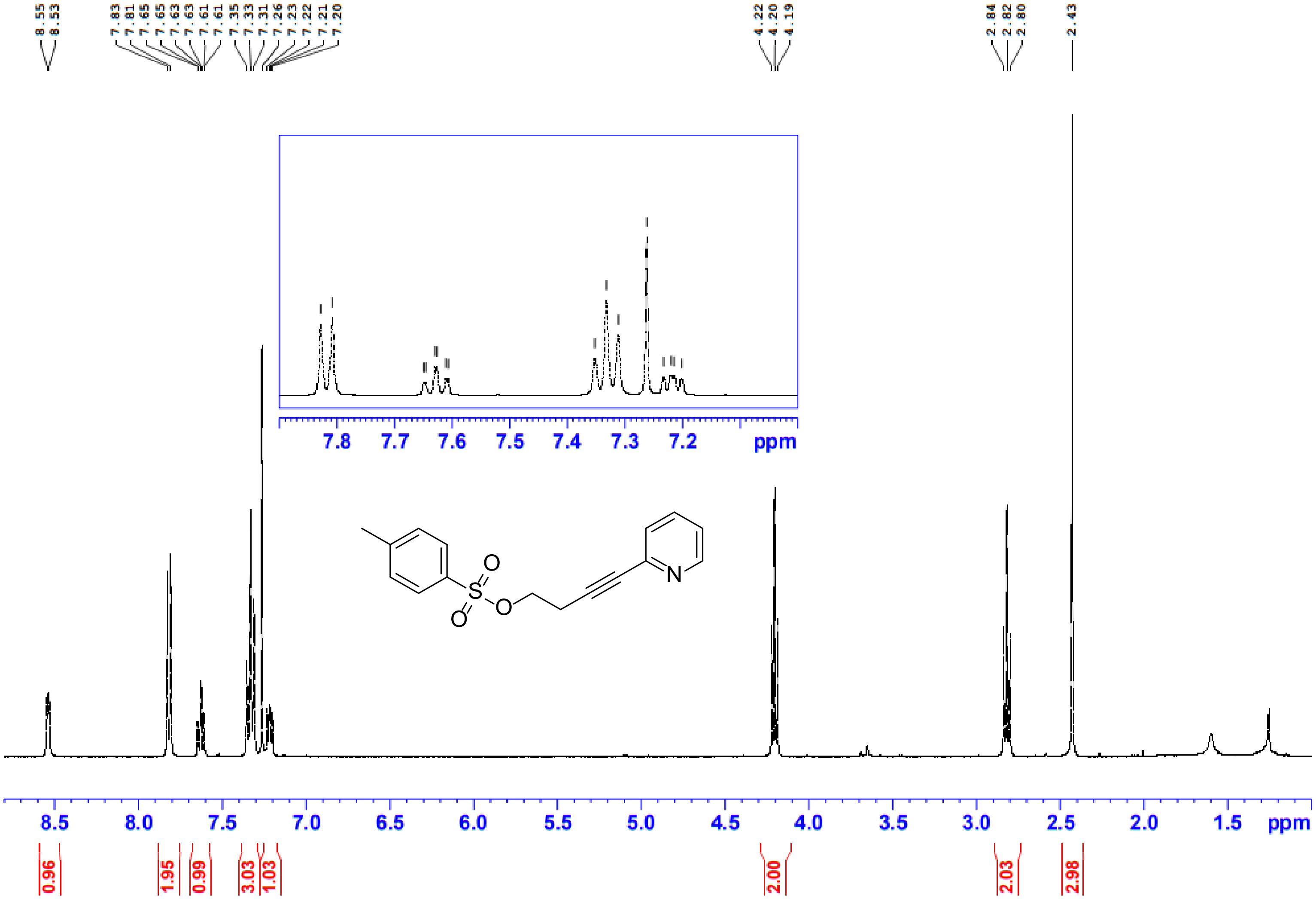


**Supplementary Figure 21.** $^1$H NMR Spectrum of **T11** (400 MHz, $CDCl_3$)

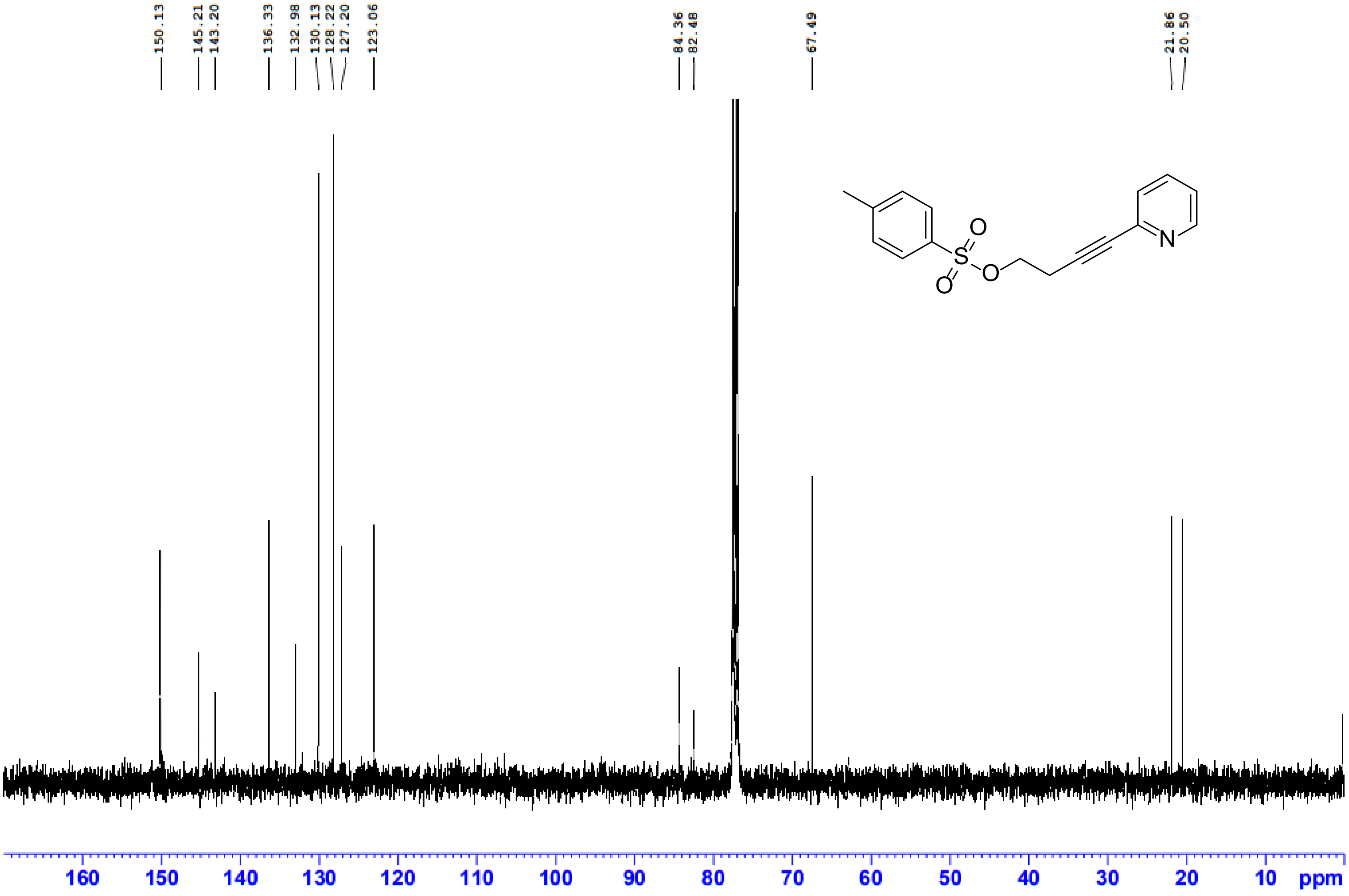


**Supplementary Figure 22.** $^{13}$C NMR Spectrum of **T11** (101 MHz, $CDCl_3$)

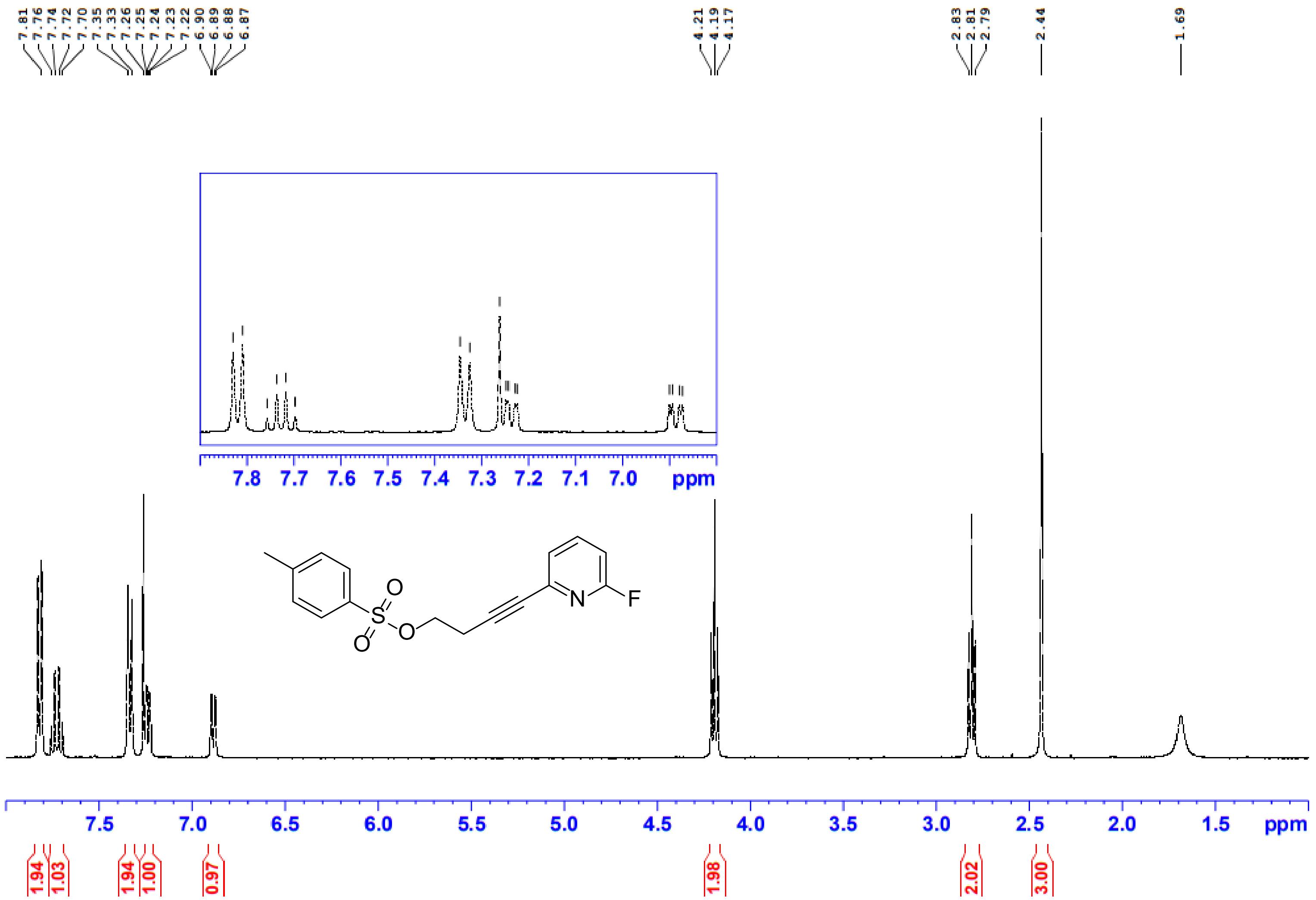


**Supplementary Figure 23.** $^{1}$H NMR Spectrum of **T12** (400 MHz, $CDCl_3$)

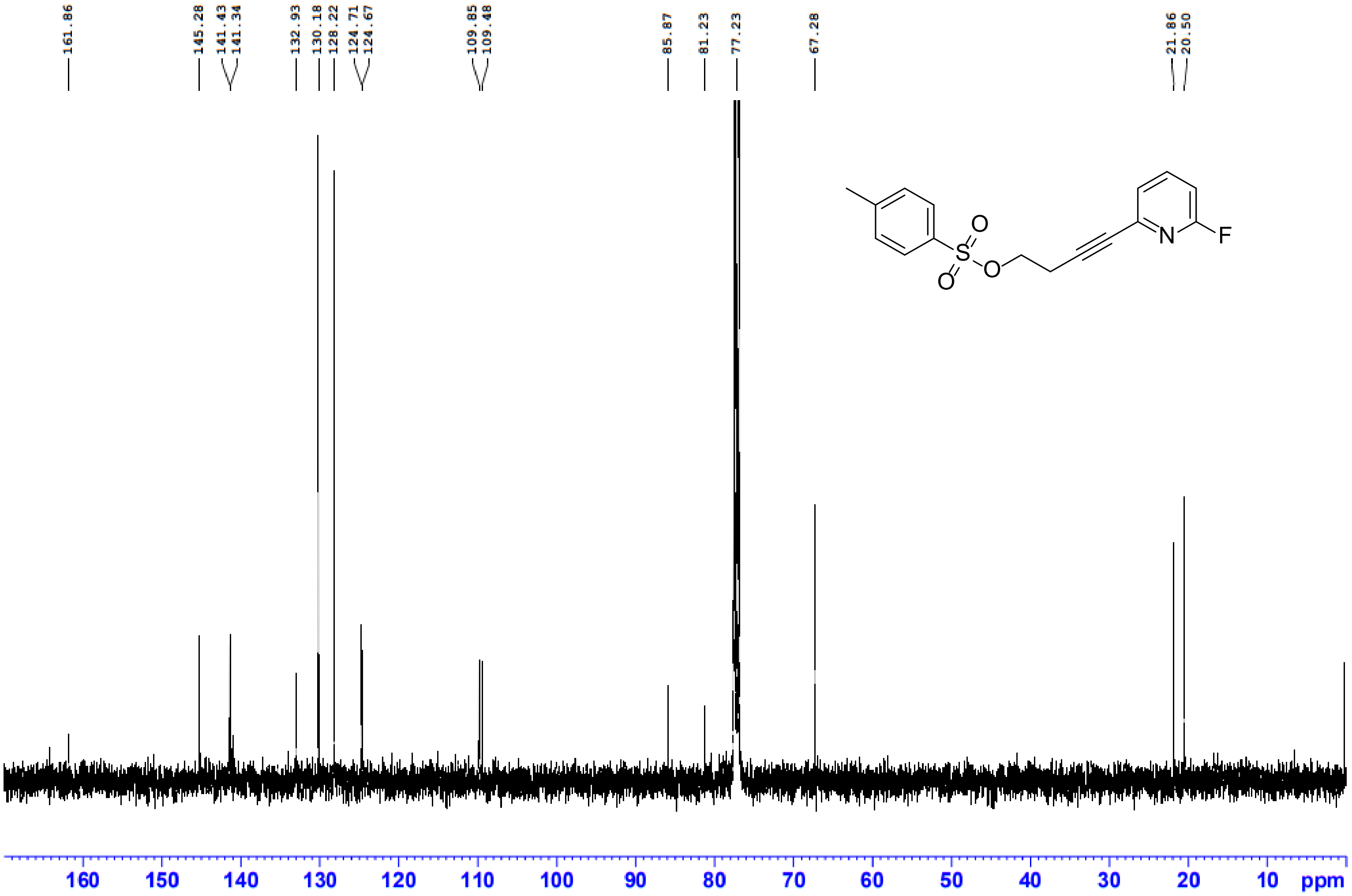


**Supplementary Figure 24.** $^{13}C$ NMR Spectrum of **T12** (101 MHz, $CDCl_3$)

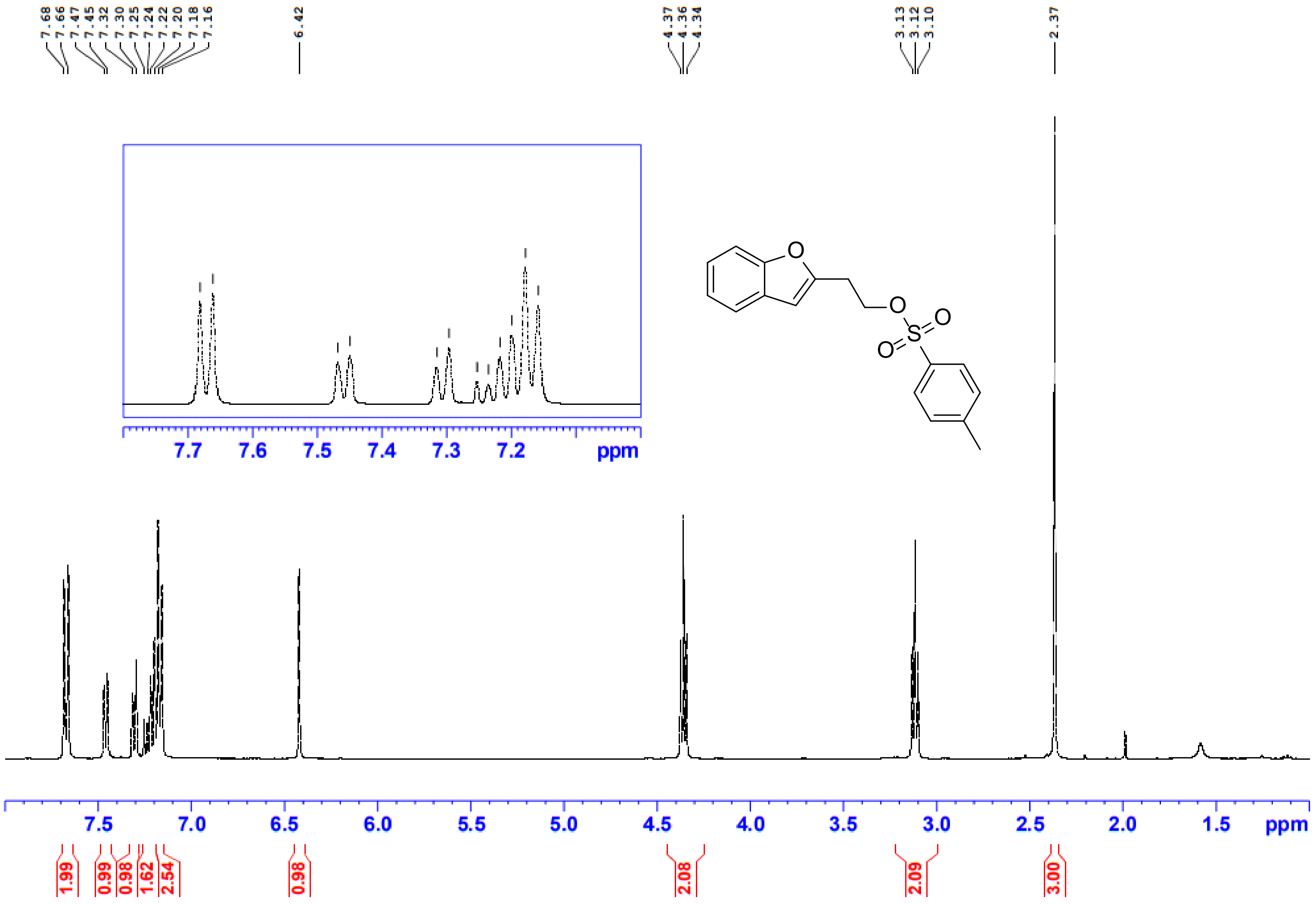


**Supplementary Figure 25.** $^{1}$H NMR Spectrum of **T13** (400 MHz, $CDCl_3$)

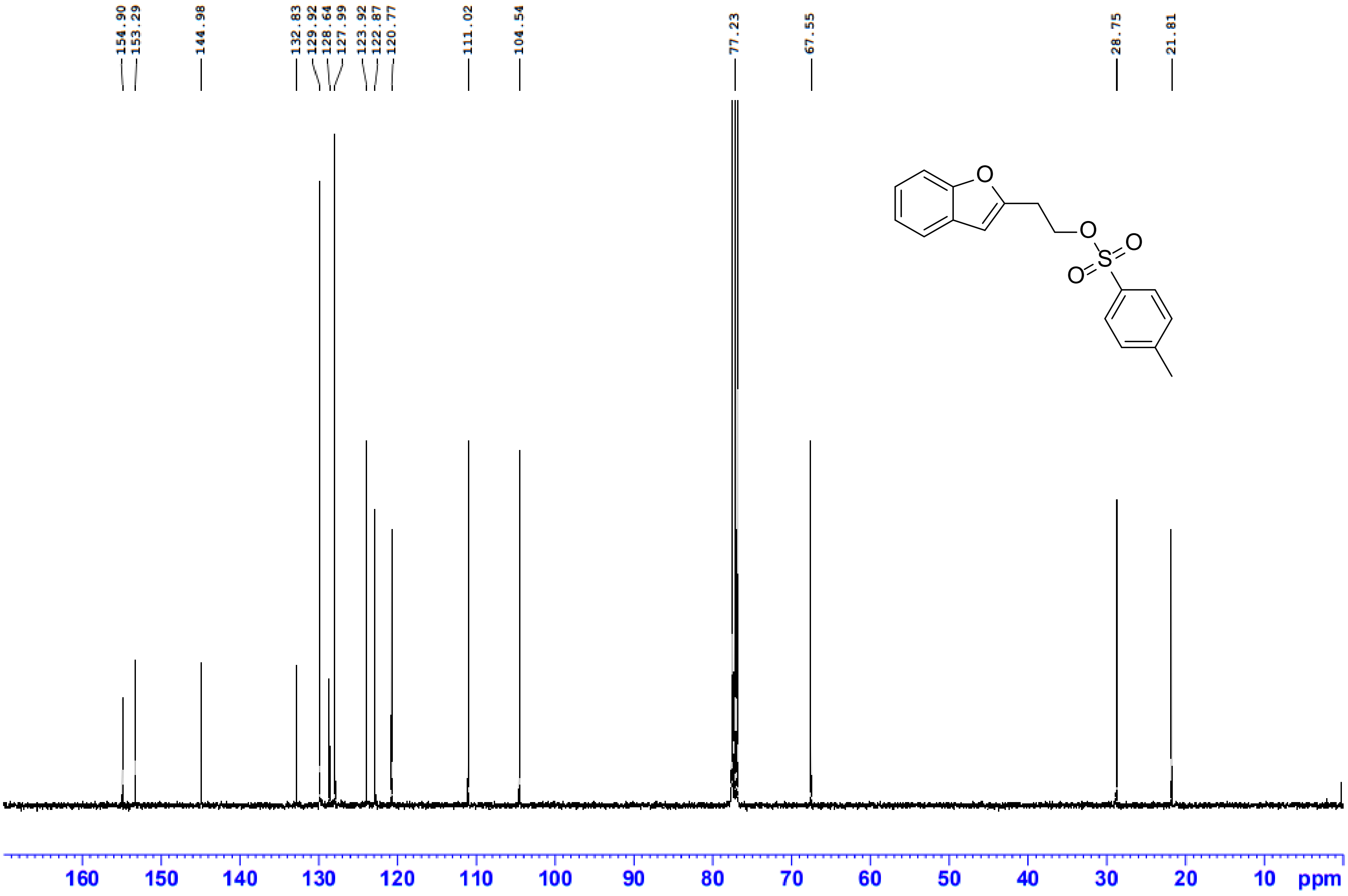


**Supplementary Figure 26.** $^{13}$C NMR Spectrum of **T13** (101 MHz, $CDCl_3$)

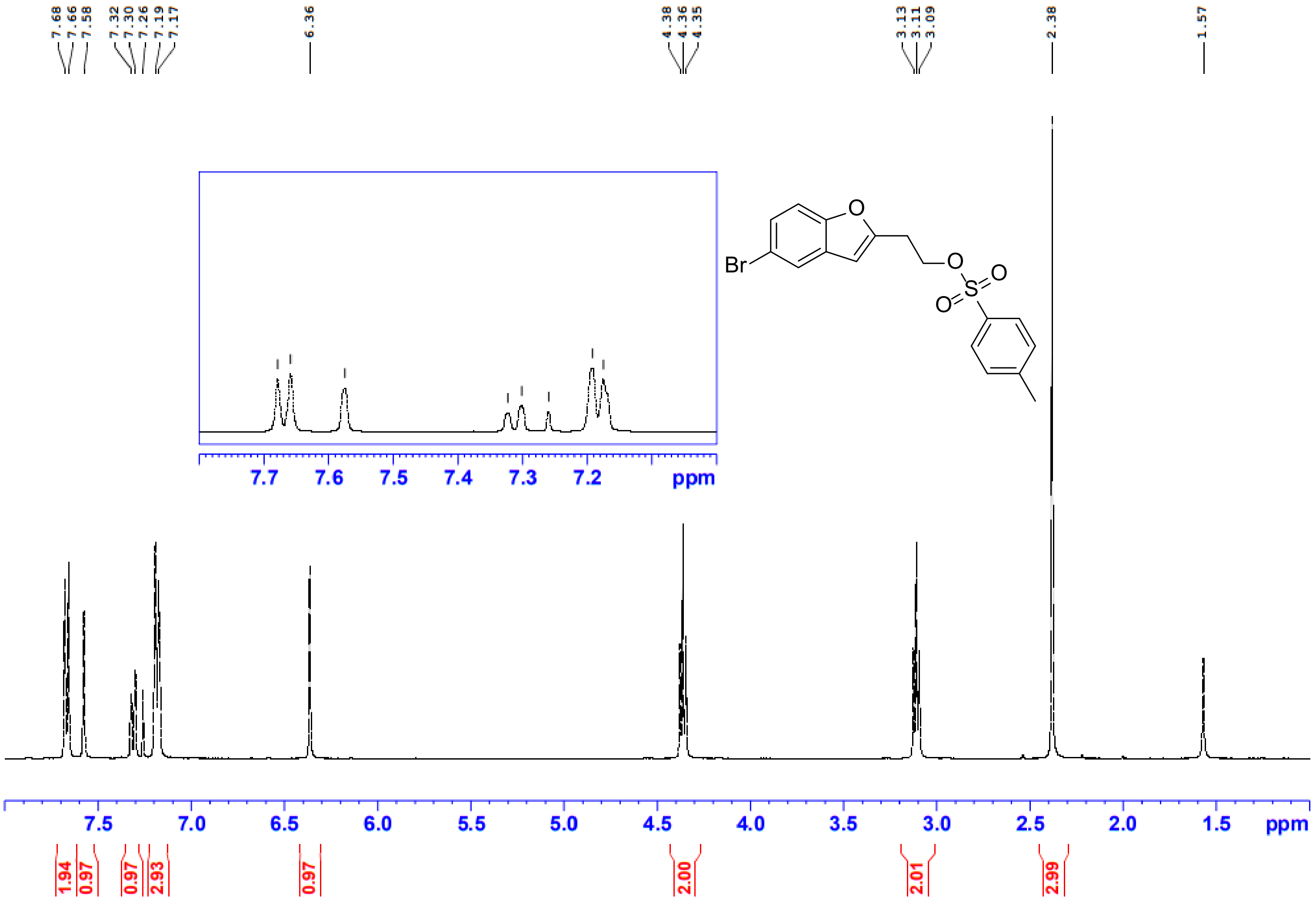


**Supplementary Figure 27.** $^{1}$H NMR Spectrum of **T14** (400 MHz, $CDCl_3$)

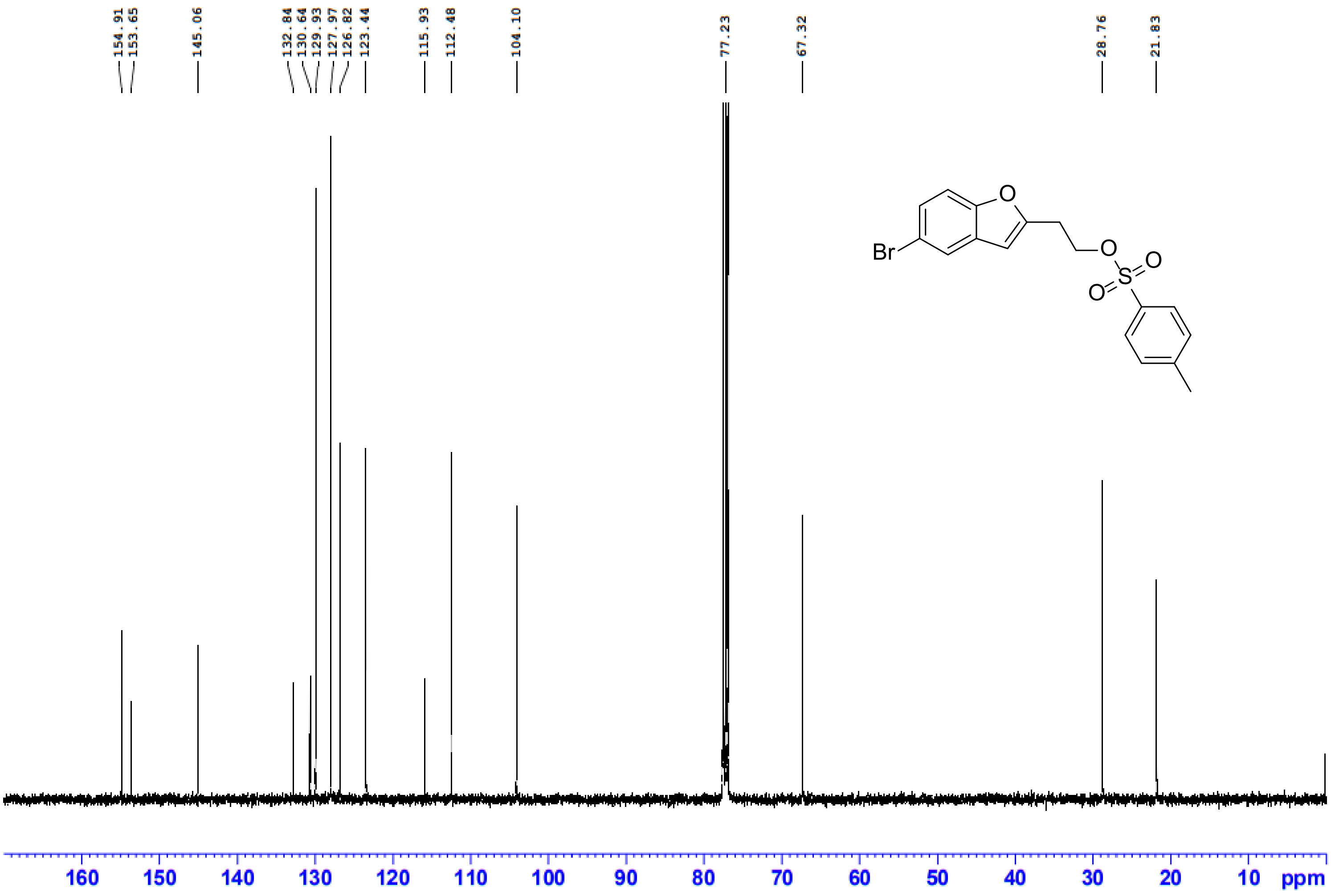


**Supplementary Figure 28.** $^{13}$C NMR Spectrum of **T14** (101 MHz, $CDCl_3$)

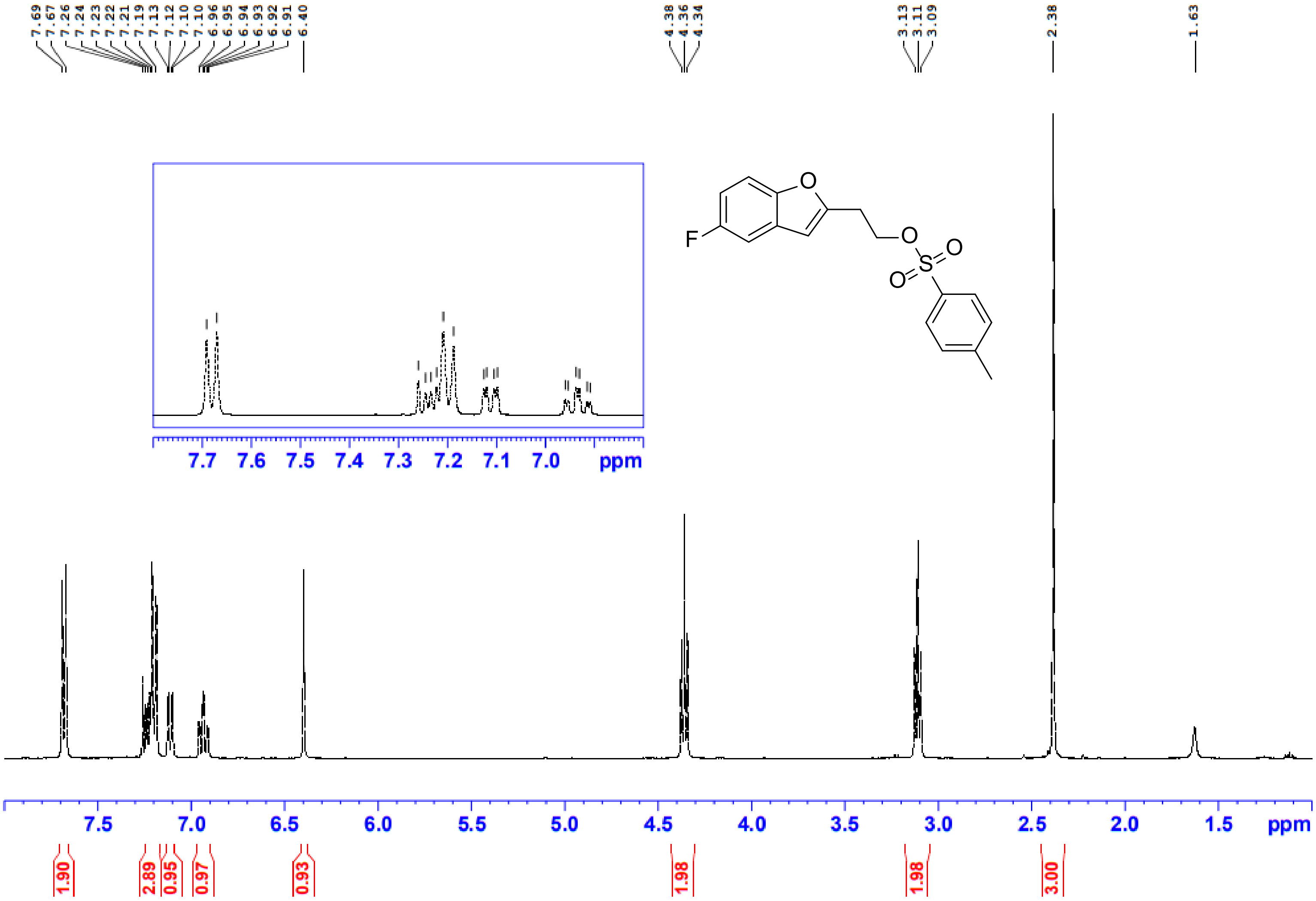

**Supplementary Figure 29.** $^1$H NMR Spectrum of **T15** (400 MHz, $CDCl_3$)

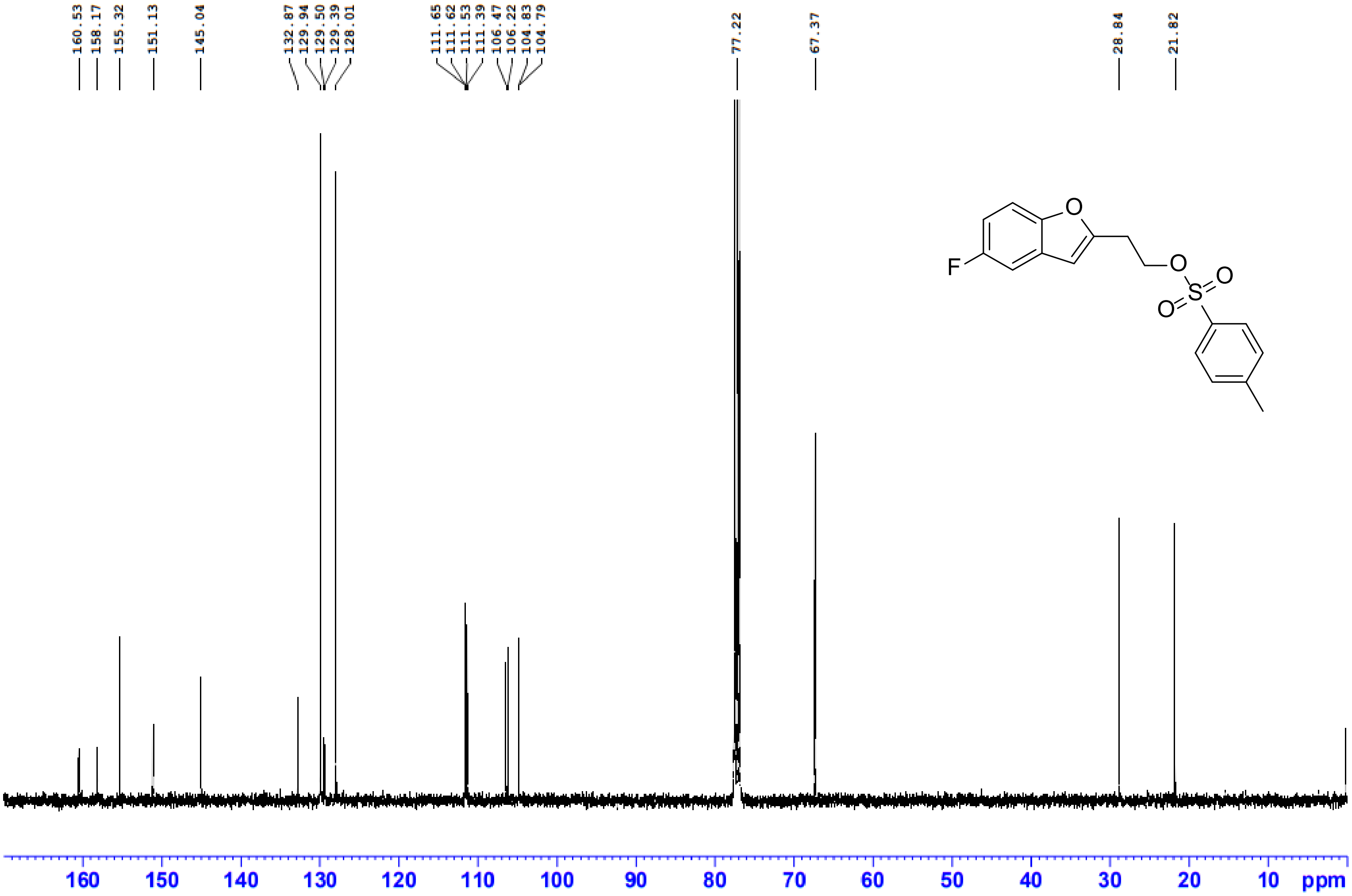


**Supplementary Figure 30.** $^{13}C$ NMR Spectrum of **T15** (101 MHz, $CDCl_3$)

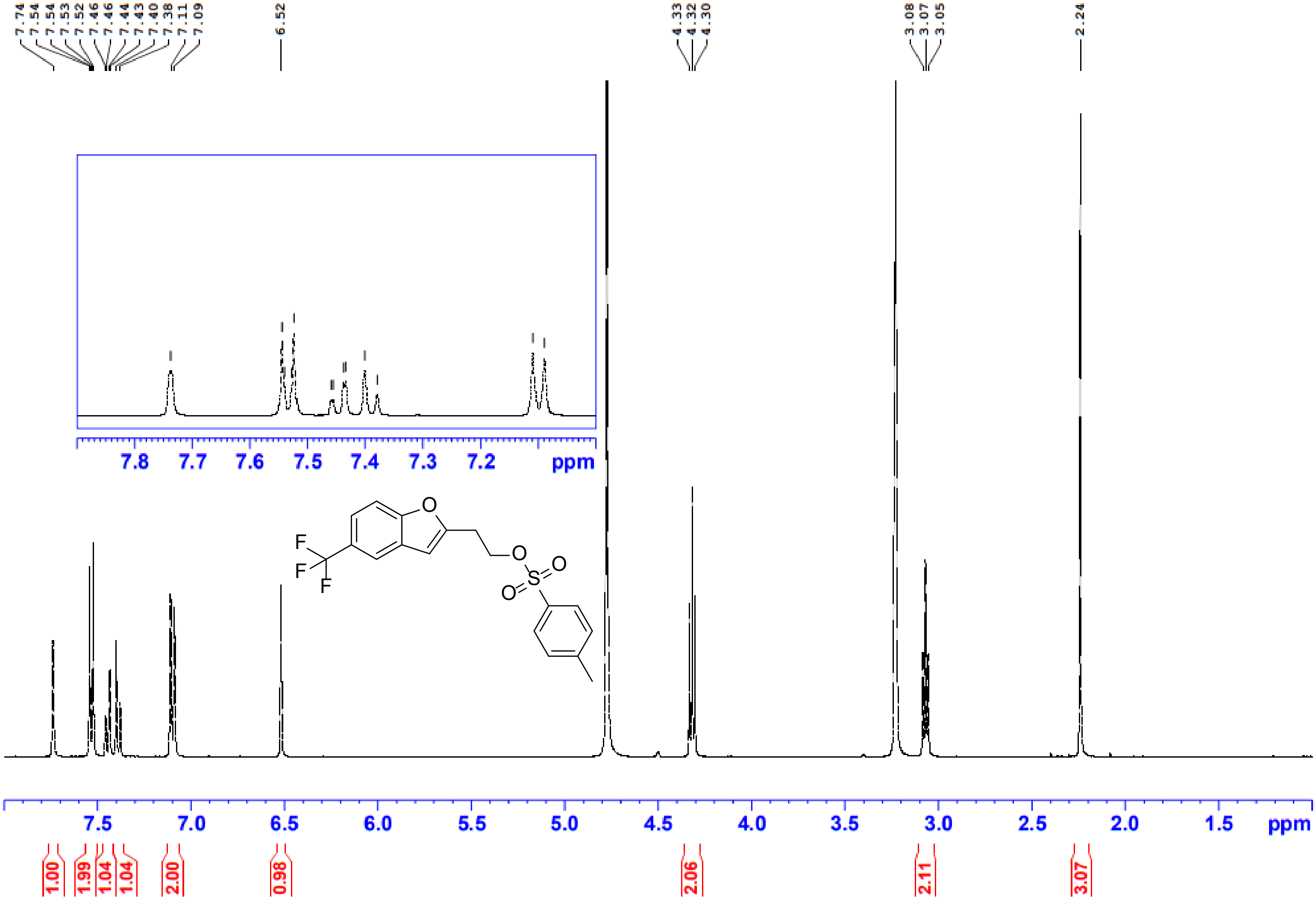


**Supplementary Figure 31.** $^{1}$H NMR Spectrum of **T16** (400 MHz, $CD_3OD$)

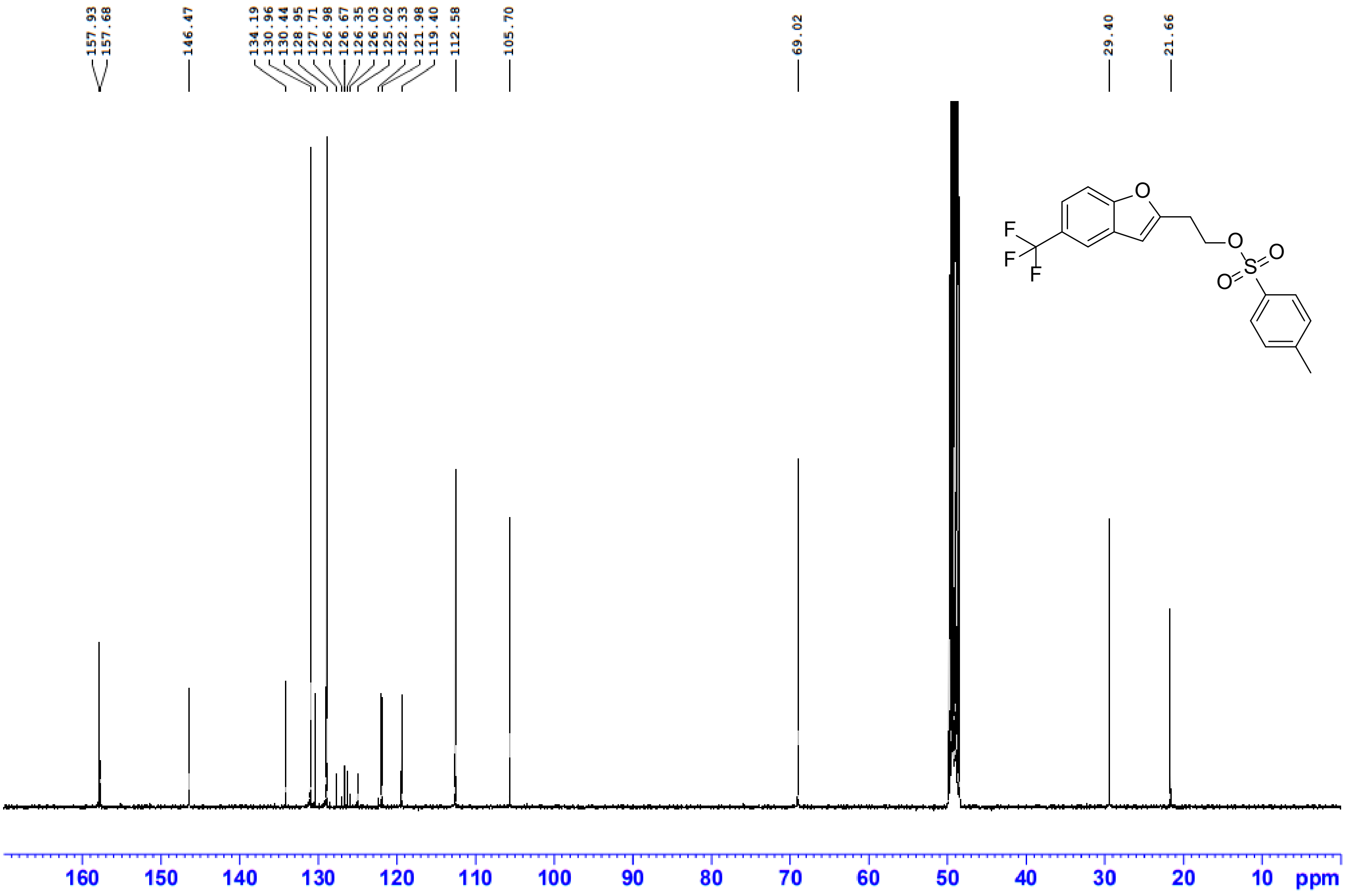


**Supplementary Figure 32.** $^{13}C$ NMR Spectrum of **T16** (101 MHz, $CD_3OD$)

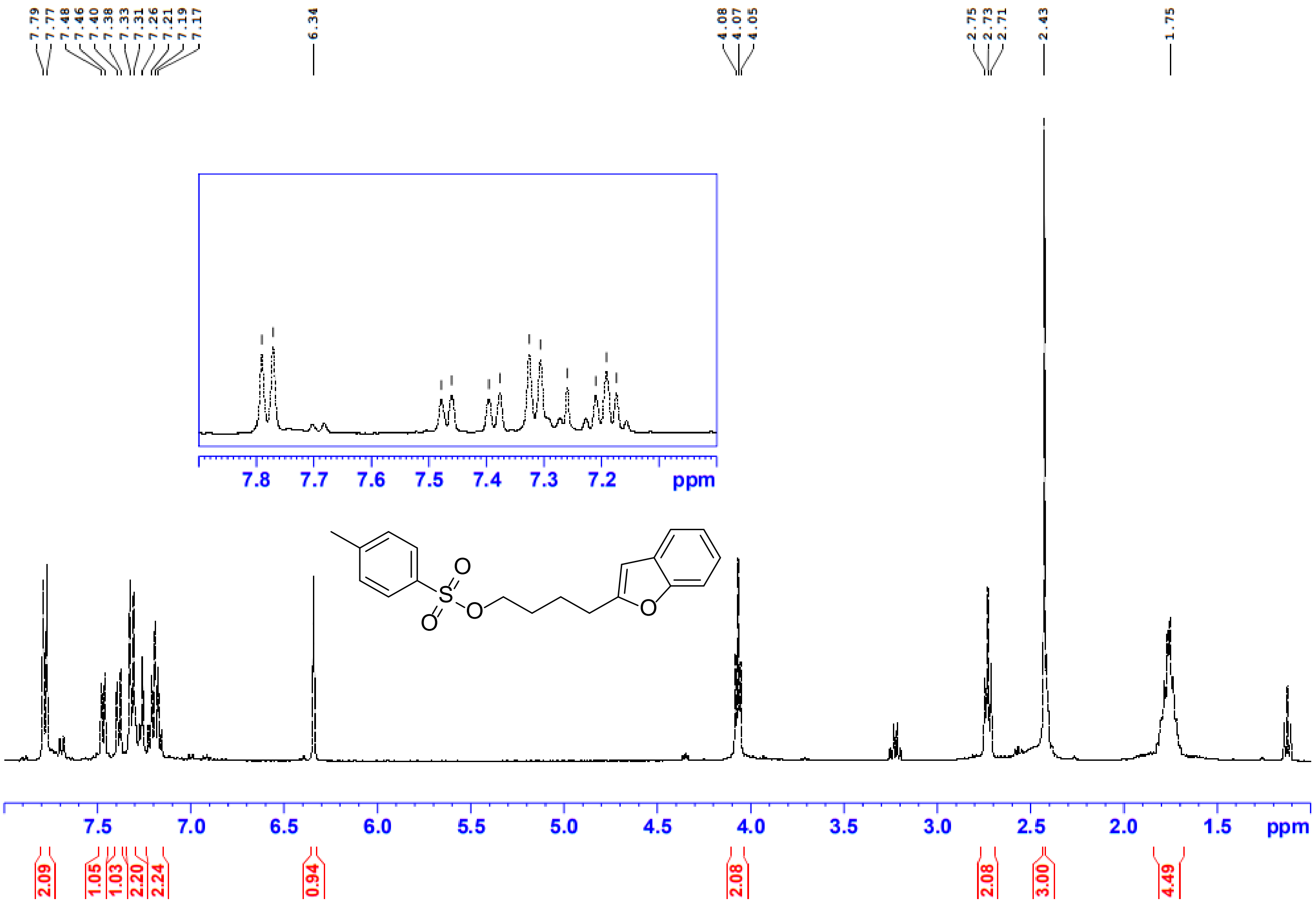

**Supplementary Figure 33.** $^{1}$H NMR Spectrum of **T17** (400 MHz, $CDCl_3$)

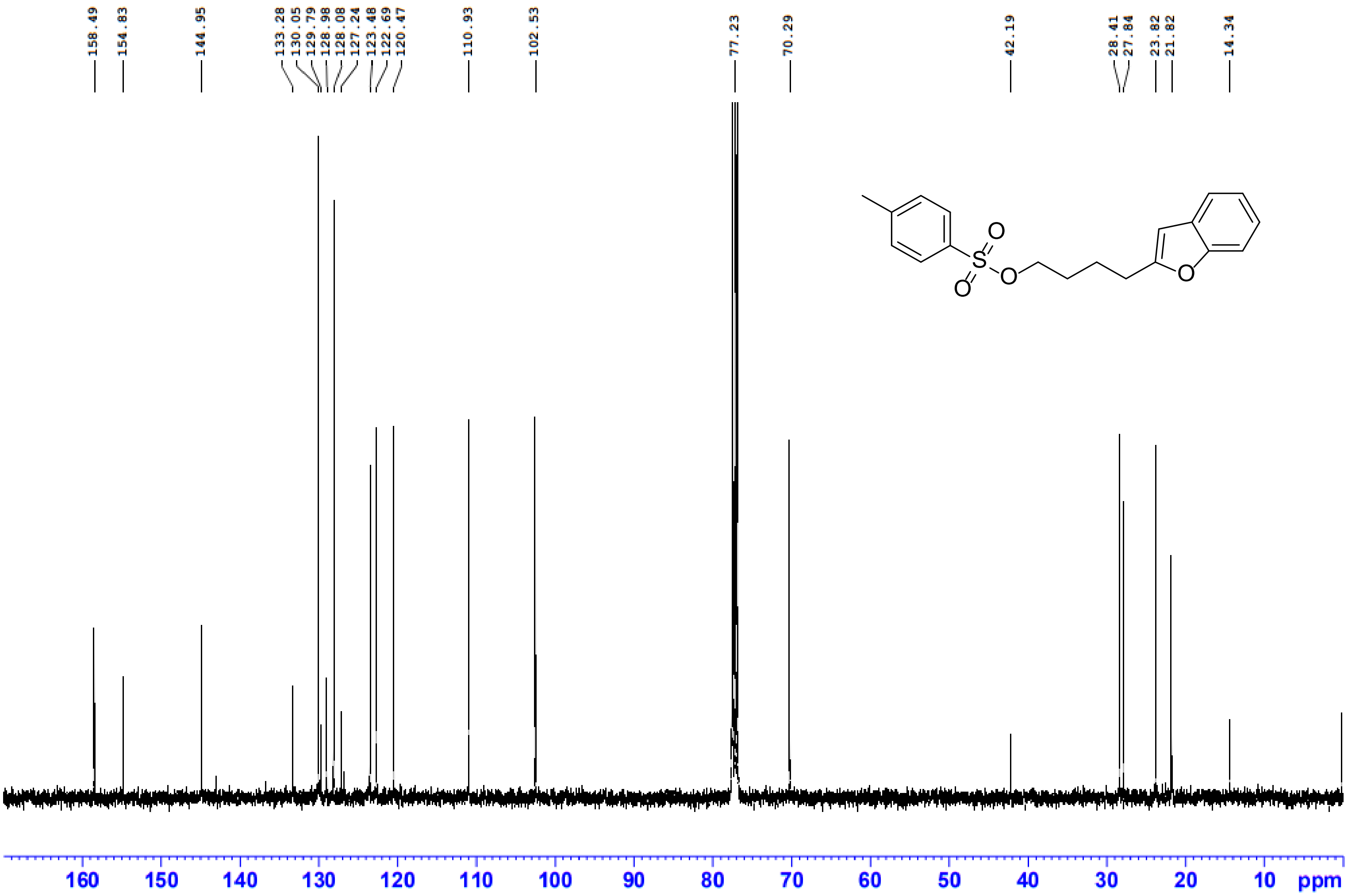


**Supplementary Figure 34.** $^{13}$C NMR Spectrum of **T17** (101 MHz, $CDCl_3$)

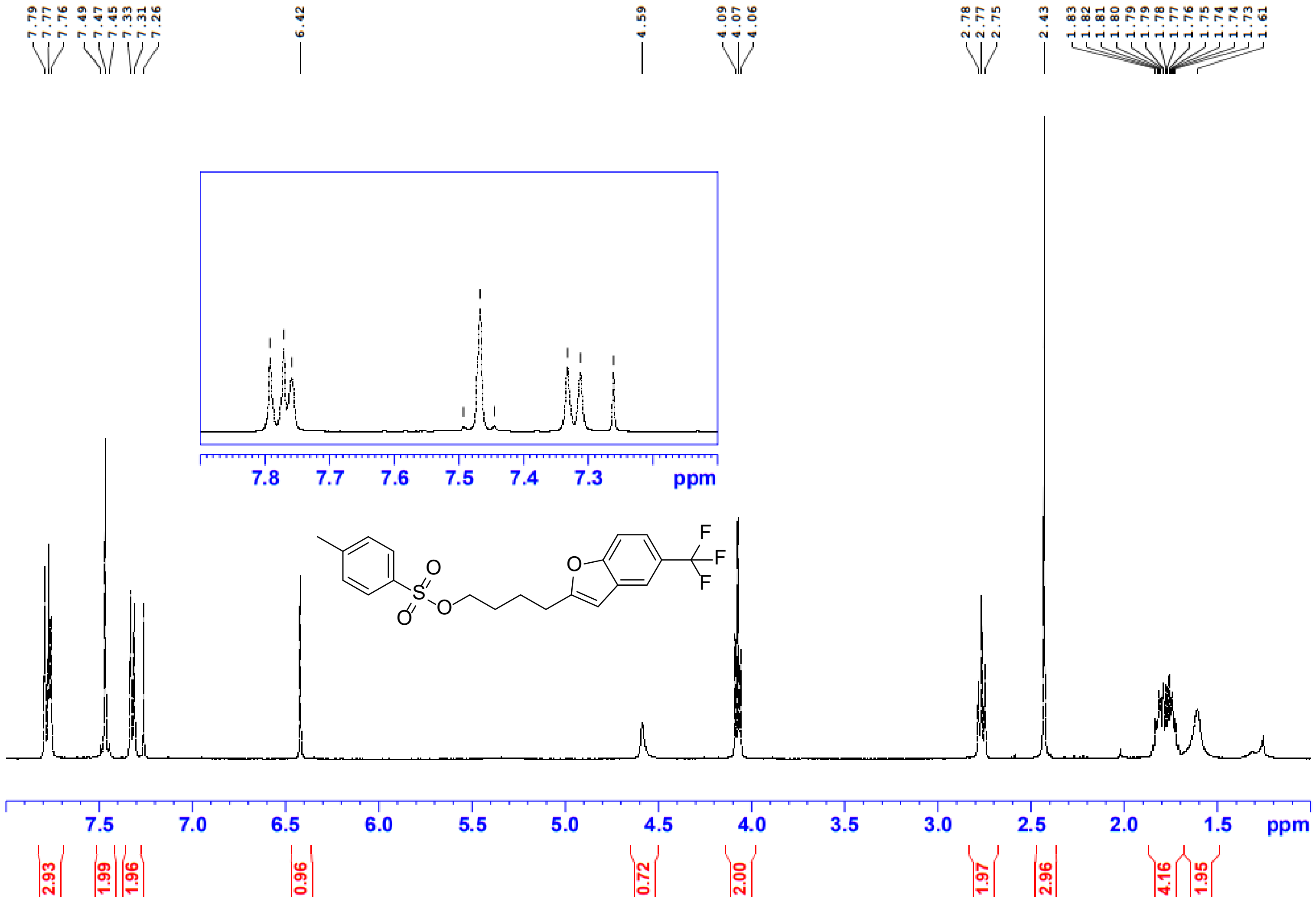

**Supplementary Figure 35.** $^1$H NMR Spectrum of **T18** (400 MHz, $CDCl_3$)

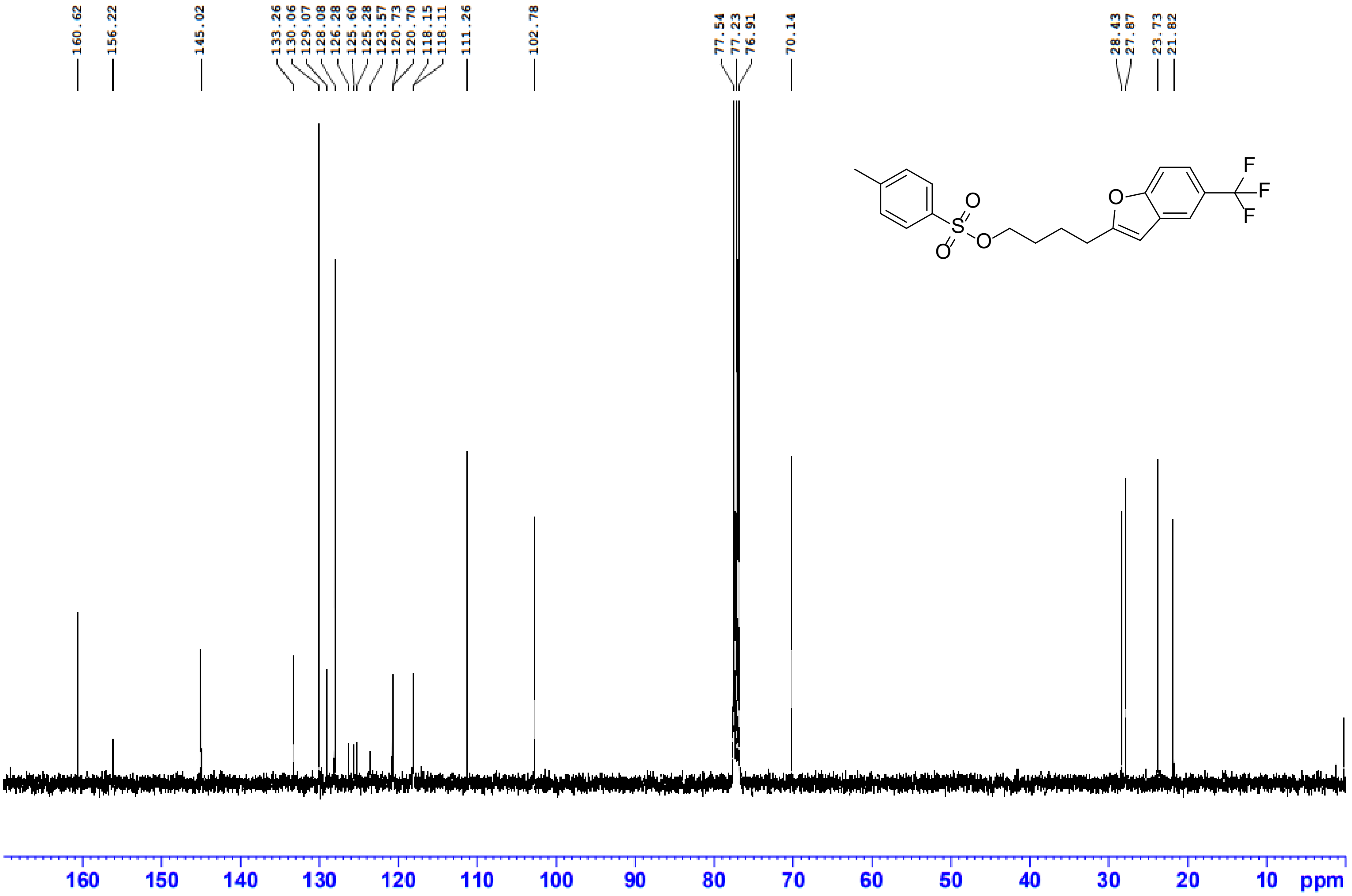


**Supplementary Figure 36.** $^{13}$C NMR Spectrum of **T18** (101 MHz, $CDCl_3$)

## 3. General HPLC Methods

**General method A**
Column: XBridge C18 (5 µm, 4.6 × 150 mm, 130 Å; Waters).
HPLC measurements were obtained at the maximum absorbance wavelength for each compound.
Solvent A: 0.025% trifluoroacetic acid in water; Solvent B: 0.025% trifluoroacetic acid in acetonitrile.
Flow rate: 6.10 mL/min
0–2 min: 30% B
2–17 min: 30 to 95% B
17–22 min: 95% B
22–23 min: 95–5% B
23–25 min: 5% B

**General method B**
Column: XBridge C18 (5 µm, 4.6 × 150 mm, 130 Å; Waters).
HPLC measurements were obtained at the maximum absorbance wavelength for each compound.
Solvent A: 0.025% trifluoroacetic acid in water; Solvent B: 0.025% trifluoroacetic acid in acetonitrile.
Flow rate: 6.10 mL/min
0–2 min: 5% B
2–17 min: 5 to 95% B
17–22 min: 95% B
22–23 min: 95–5% B
23–25 min: 5% B

**General method C**
Column: XBridge BEH C18 OBD (5 µm, 4.6 × 150 mm, 130 Å; Waters).
HPLC measurements were obtained at the maximum absorbance wavelength for each compound.
Solvent A: 0.025% trifluoroacetic acid in water; Solvent B: 0.025% trifluoroacetic acid in acetonitrile.
Flow rate: 22.2 mL/min
0–2 min: 10% B
2–22 min: 10 to 90% B
22–24 min: 90 to 95% B
24–28 min: 95% B
31–36 min: 10% B

**General method D**
Column: XBridge BEH C18 OBD (10 µm, 19 × 150 mm, 130 Å; Waters).
HPLC measurements were obtained at the maximum absorbance wavelength for each compound.
Solvent A: 0.025% trifluoroacetic acid in water; Solvent B: 0.025% trifluoroacetic acid in acetonitrile.
Flow rate: 22.2 mL/min
0–2 min: 10% B
2–22 min: 10 to 50% B

22–24 min: 50 to 95% B
24–28 min: 95% B
31–36 min: 10% B

**General method E**
Column: (*S,S*)-Whelk-O1 (5 µm, 250 × 4.6 mm, 100 Å; Regis Technologies).
HPLC measurements were obtained at the maximum absorbance wavelength for each compound.
Solvent A: 0.1% triethylamine in hexane; Solvent B: 0.1% triethylamine in ethanol.
Flow rate: 1.9 mL/min
0–2 min: 20% B
2–20 min: 20 to 40% B
20–25.1 min: 20% B
25.1–30 min: 20% B

**General method F**
Column: (*S,S*)-Whelk-O1 (250 × 21.1 mm; Regis Technologies).
HPLC measurements were obtained at the maximum absorbance wavelength for each compound.
Solvent A: 0.1% triethylamine in hexane; Solvent B: 0.1% triethylamine in ethanol
Flow rate: 18.2 mL/min
0–2 min: 20% B
2–25 min: 20 to 40% B
25–26 min: 40 to 95% B
26– 30 min: 95% B
30–31 min: 20% B

**General Method G**
Column: XBridge BEH C18 OBD (10 µm, 19 × 150 mm, 130 Å; Waters).
HPLC measurements were obtained at the maximum absorbance wavelength for each compound.
Solvent A: 0.025% trifluoroacetic acid in water; Solvent B: 0.025% trifluoroacetic acid in acetonitrile
Flow rate: 22.2 mL/min
0–2 min: 20% B
2–22 min: 20 to 60% B
22–24 min: 60 to 95% B
24–28 min: 95% B
28-31 min: 95 to 20% B
31–32 min: 20% B

**General Method H**
Column: XBridge C18 (5 µm, 10 × 250 mm, 130 Å; Waters)
HPLC measurements were obtained at 274 nm
Solvent A: 0.025% trifluoroacetic acid in water; Solvent B: 0.025% trifluoroacetic acid in acetonitrile.
Flow rate: 6.0 mL/min
0-40 min: 35% B

**General Method I**
Column: XBridge C18 (5 µm, 4.6 × 250 mm, 130 Å; Waters)
HPLC measurements were obtained at 274 nm.
Solvent A: 0.025% trifluoroacetic acid in water; Solvent B: 0.025% trifluoroacetic acid in methanol.
Flow rate: 1.50 mL/min
0-20 min: 50% B

## 4. Analytical HPLC Chromatograms for Ligands L1–L10 and L12–L21

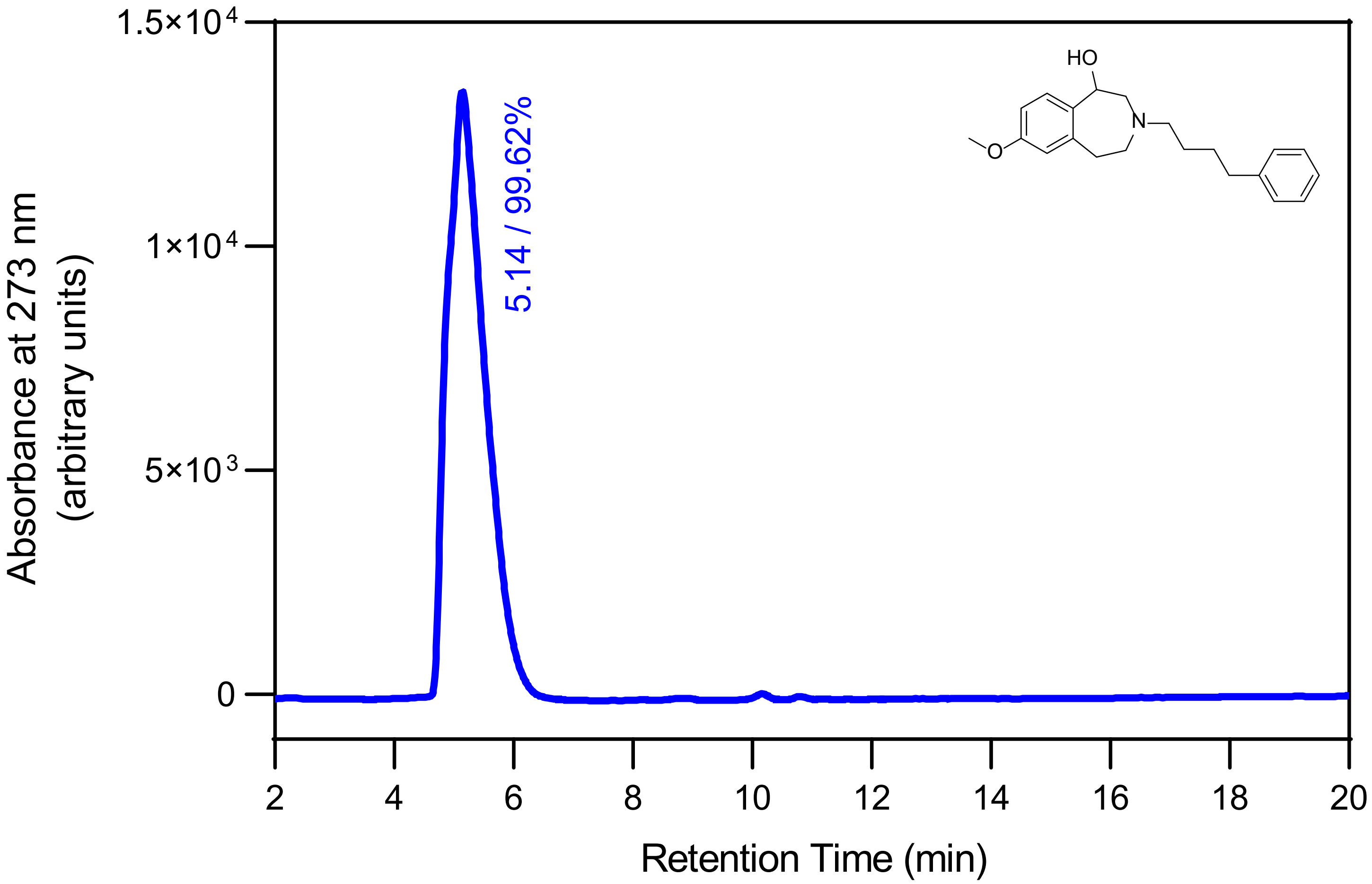


**Supplementary Figure 37.** Analytical HPLC chromatogram for **L1** (general method A).

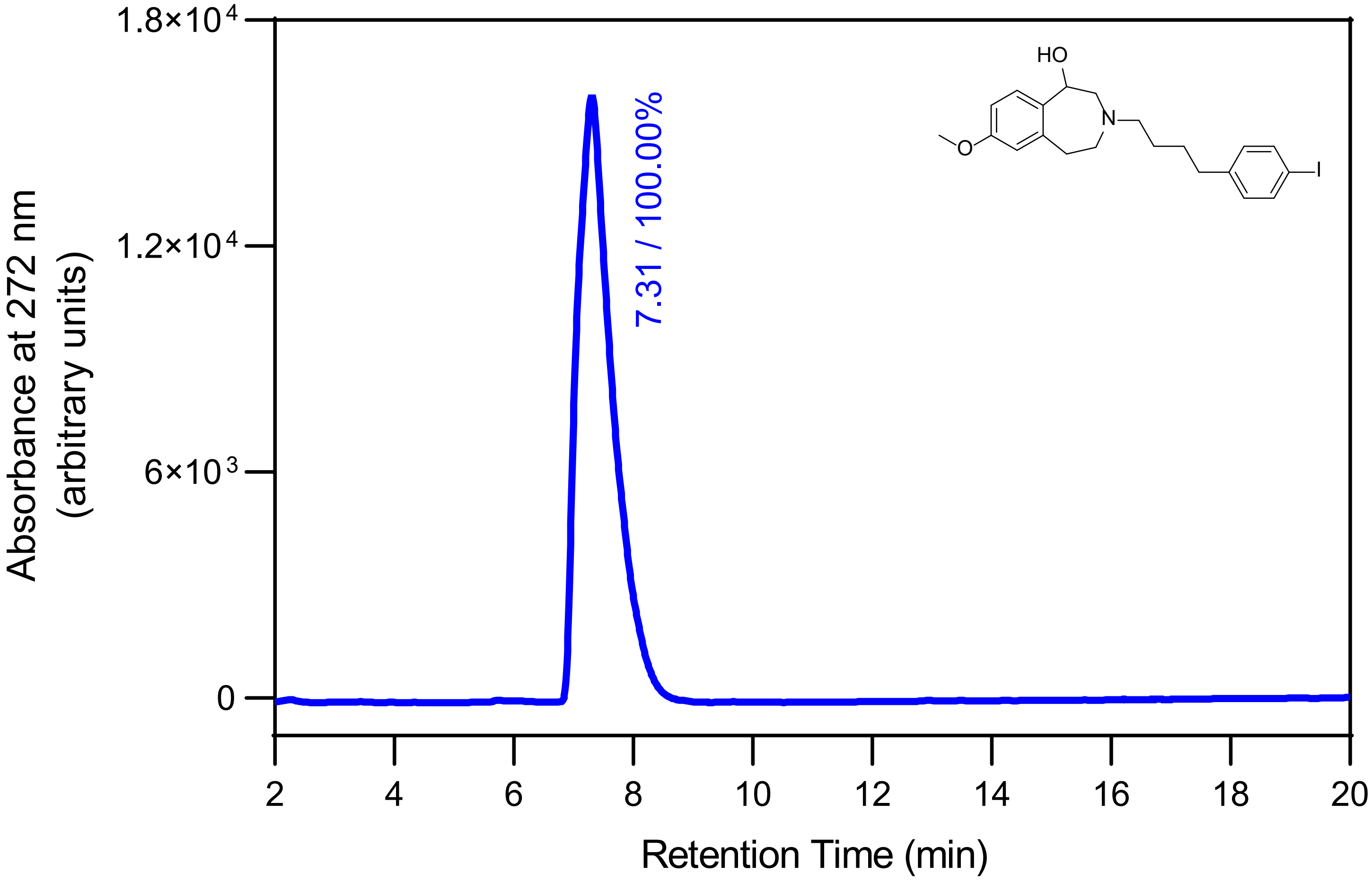


**Supplementary Figure 38.** Analytical HPLC chromatogram for **L2** using (general method A).

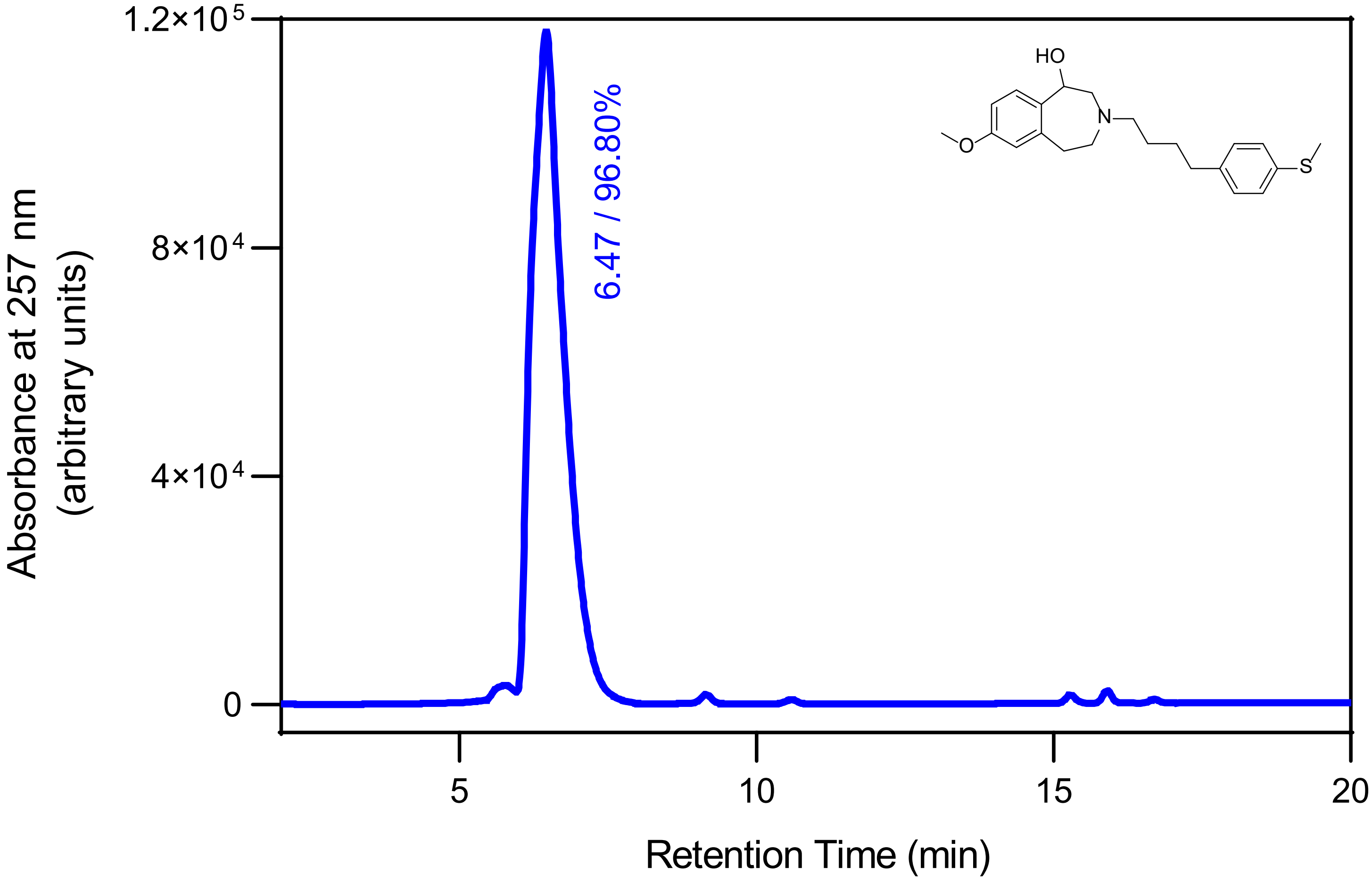


**Supplementary Figure 39.** Analytical HPLC chromatogram for **L3** (general method A).

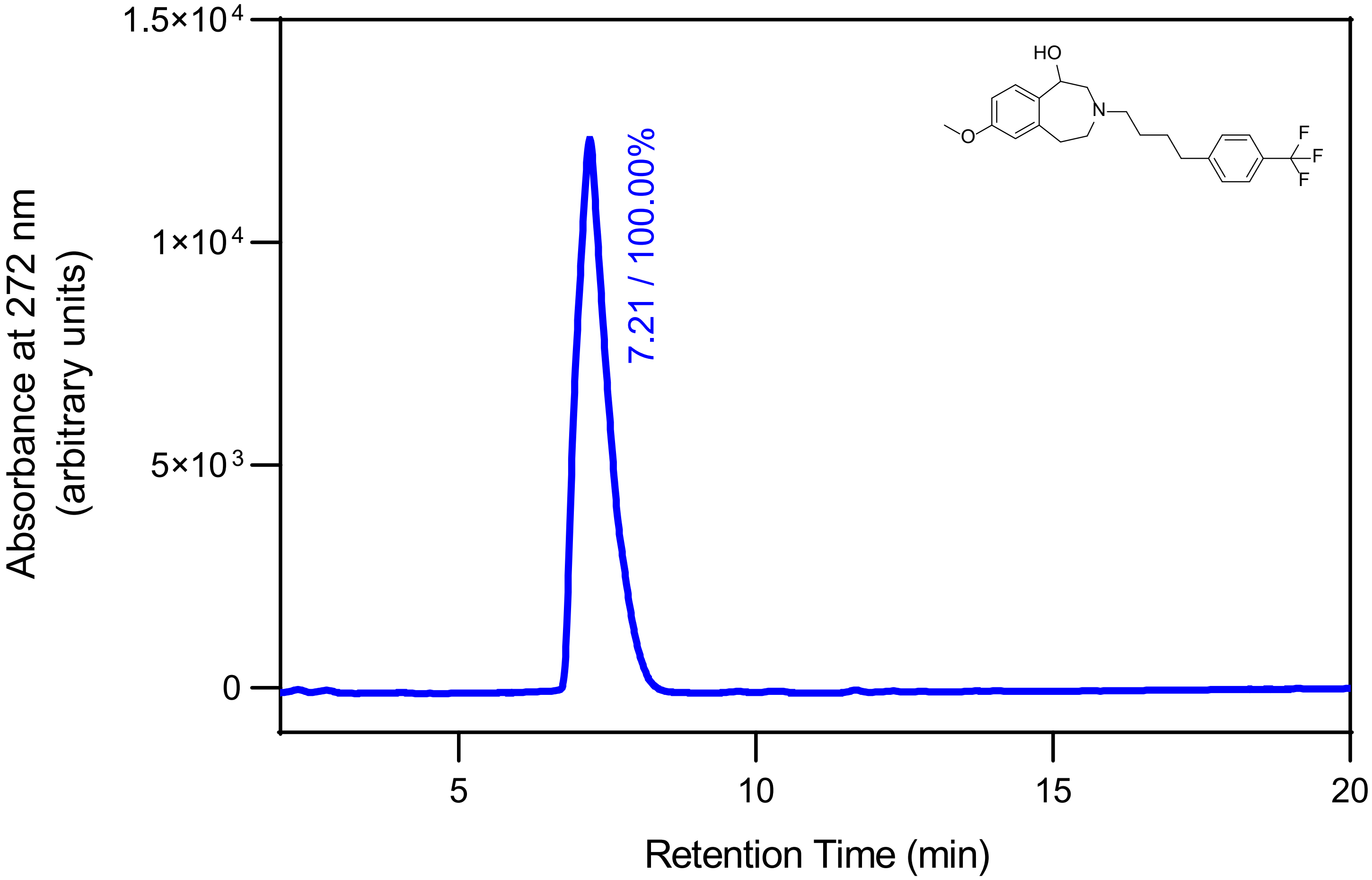


**Supplementary Figure 40.** Analytical HPLC chromatogram for **L4** (general method A).

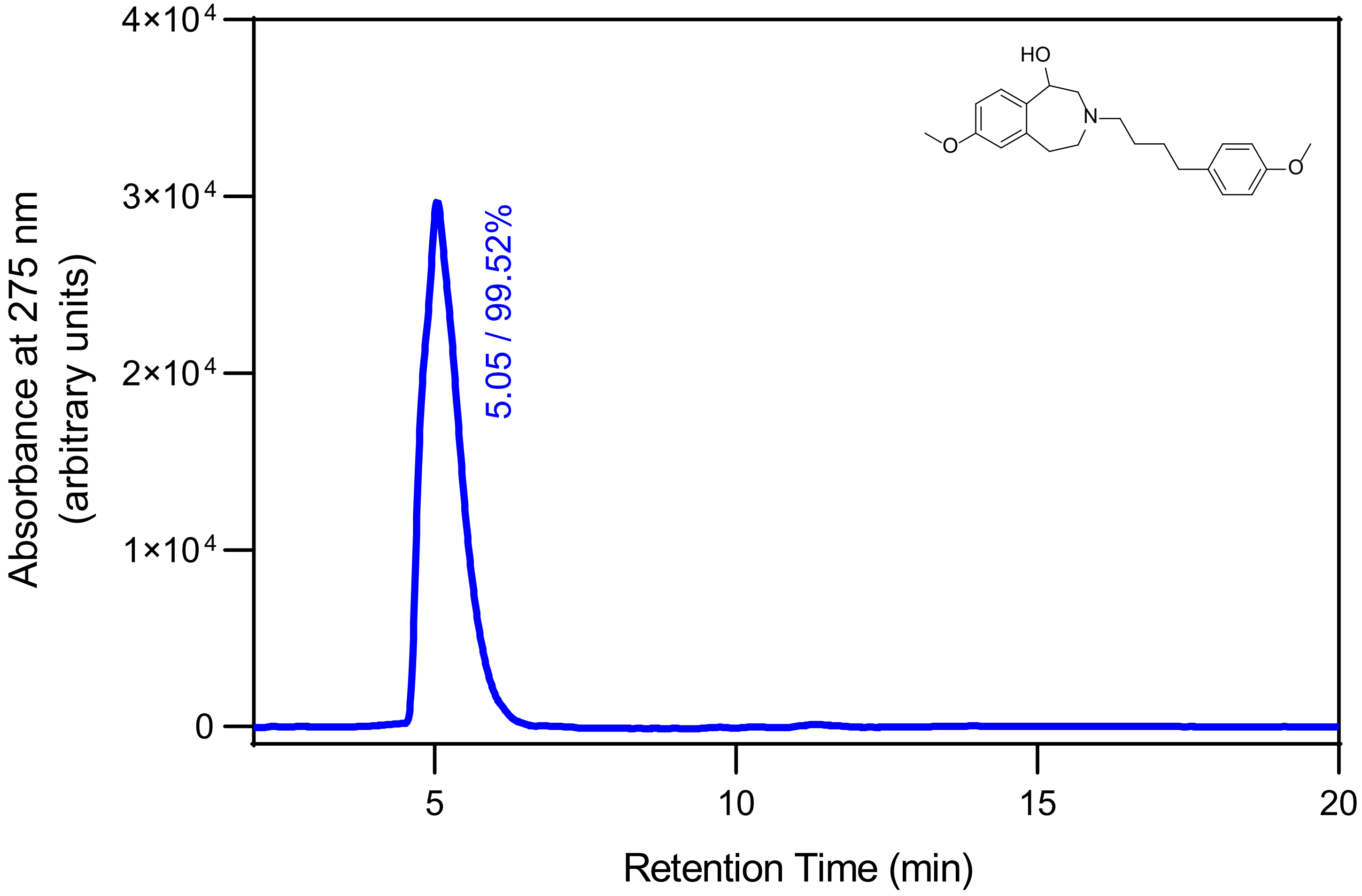


**Supplementary Figure 41.** Analytical HPLC chromatogram for **L5** (general method A).

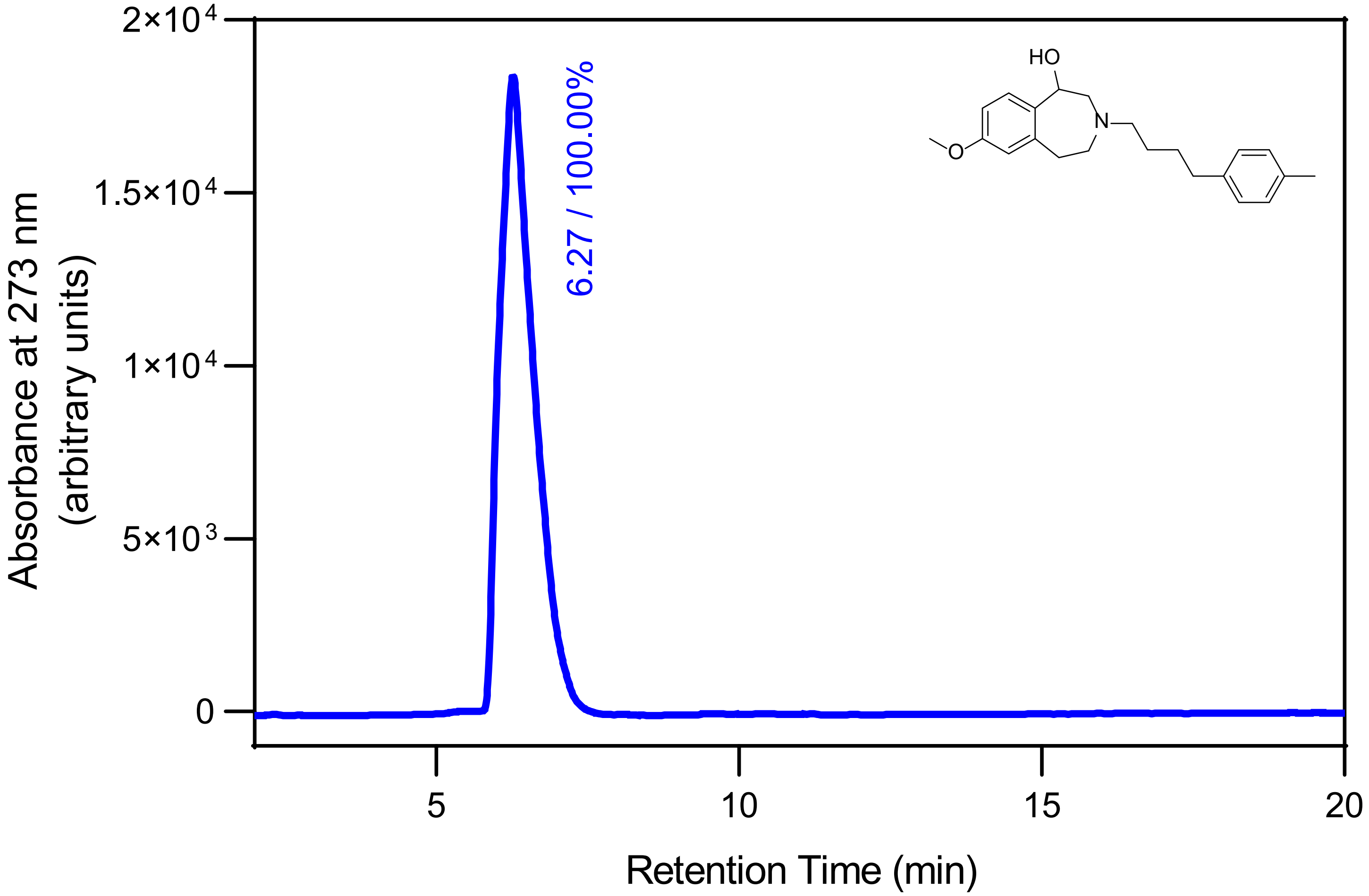


**Supplementary Figure 42.** Analytical HPLC chromatogram for **L6** (general method A).

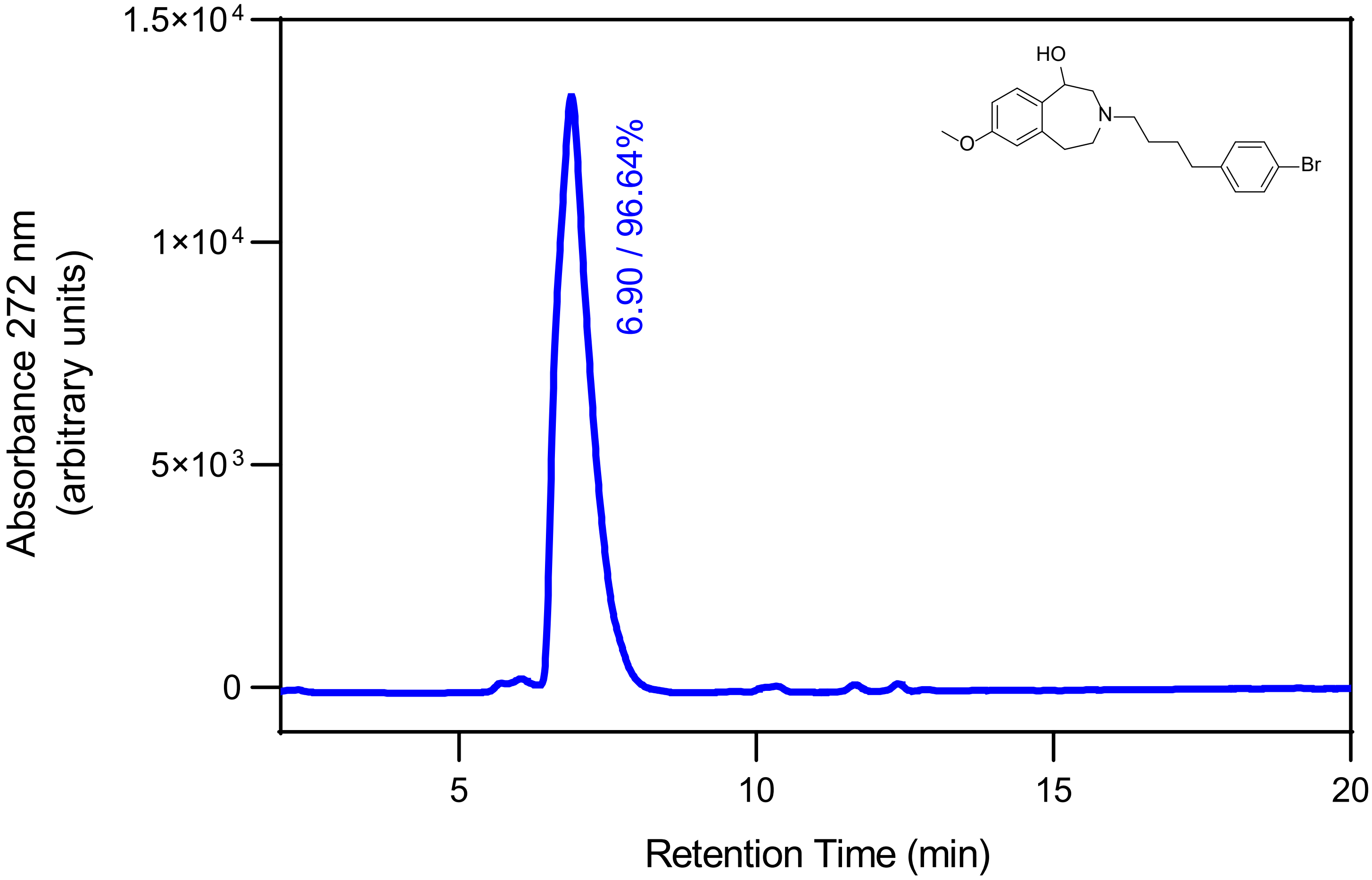


**Supplementary Figure 43.** Analytical HPLC chromatogram for **L7** (general method A).

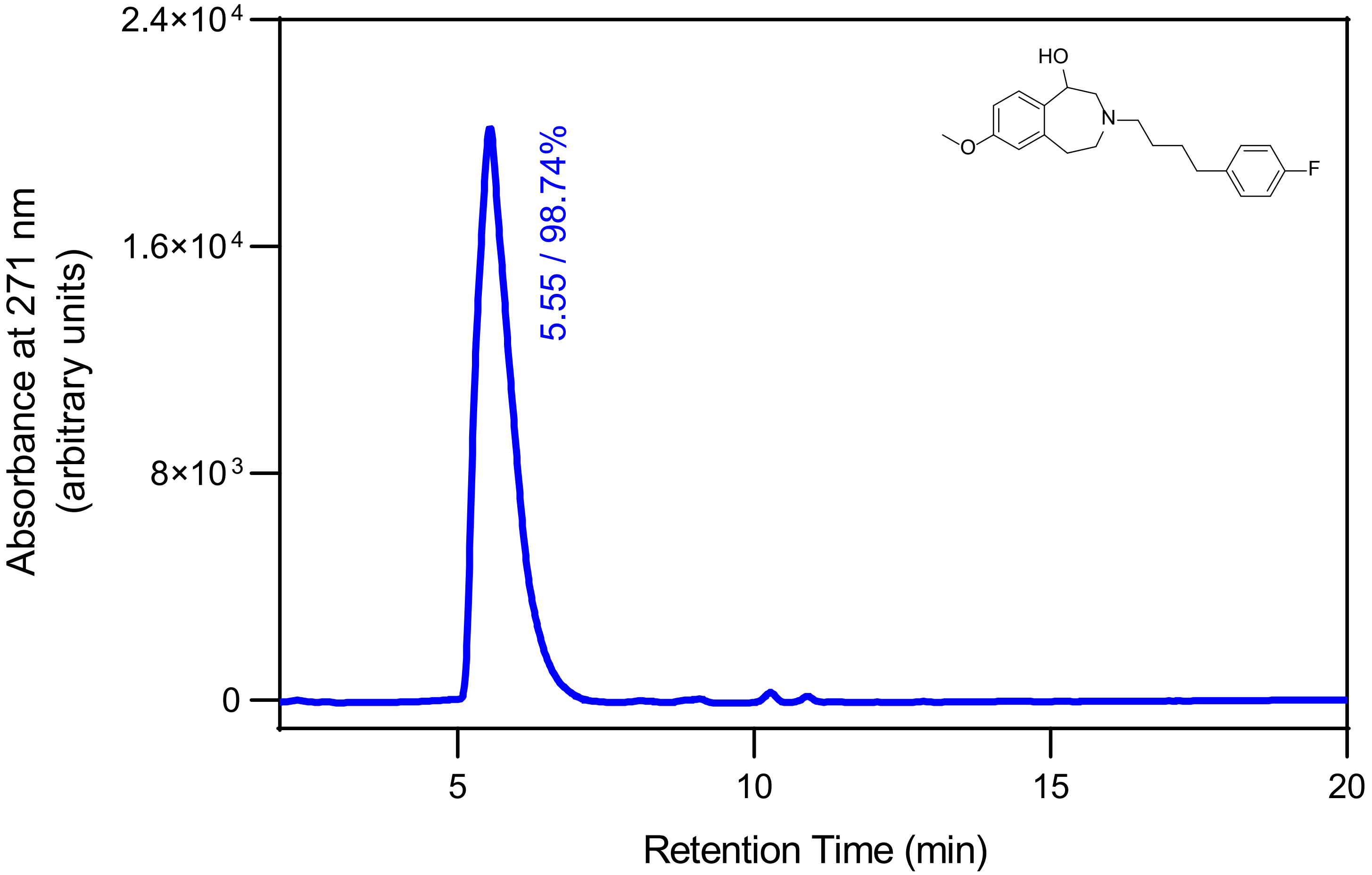


**Supplementary Figure 44.** Analytical HPLC chromatogram for **L8** (general method A).

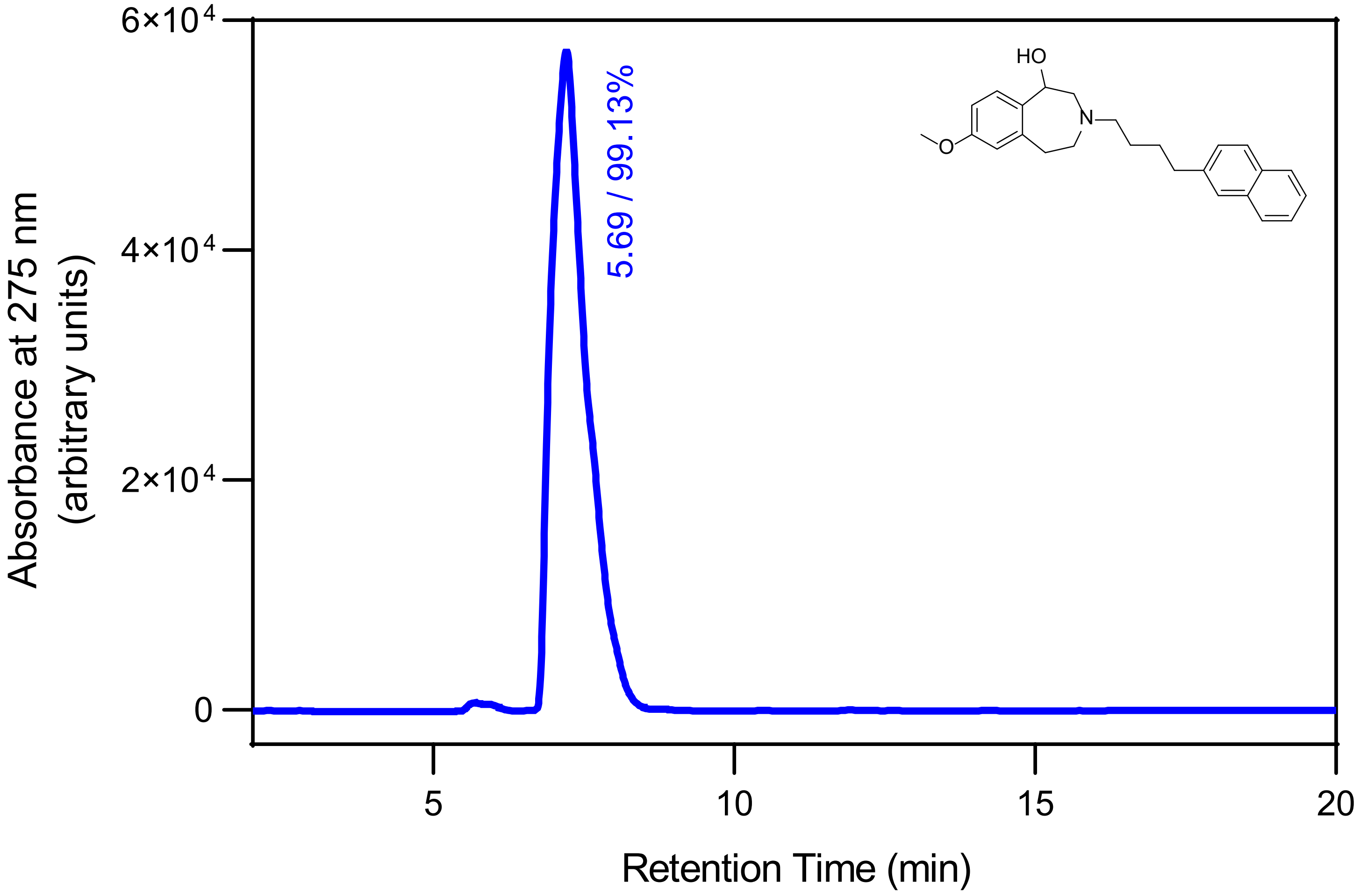


**Supplementary Figure 45.** Analytical HPLC chromatogram for **L9** (general method A).

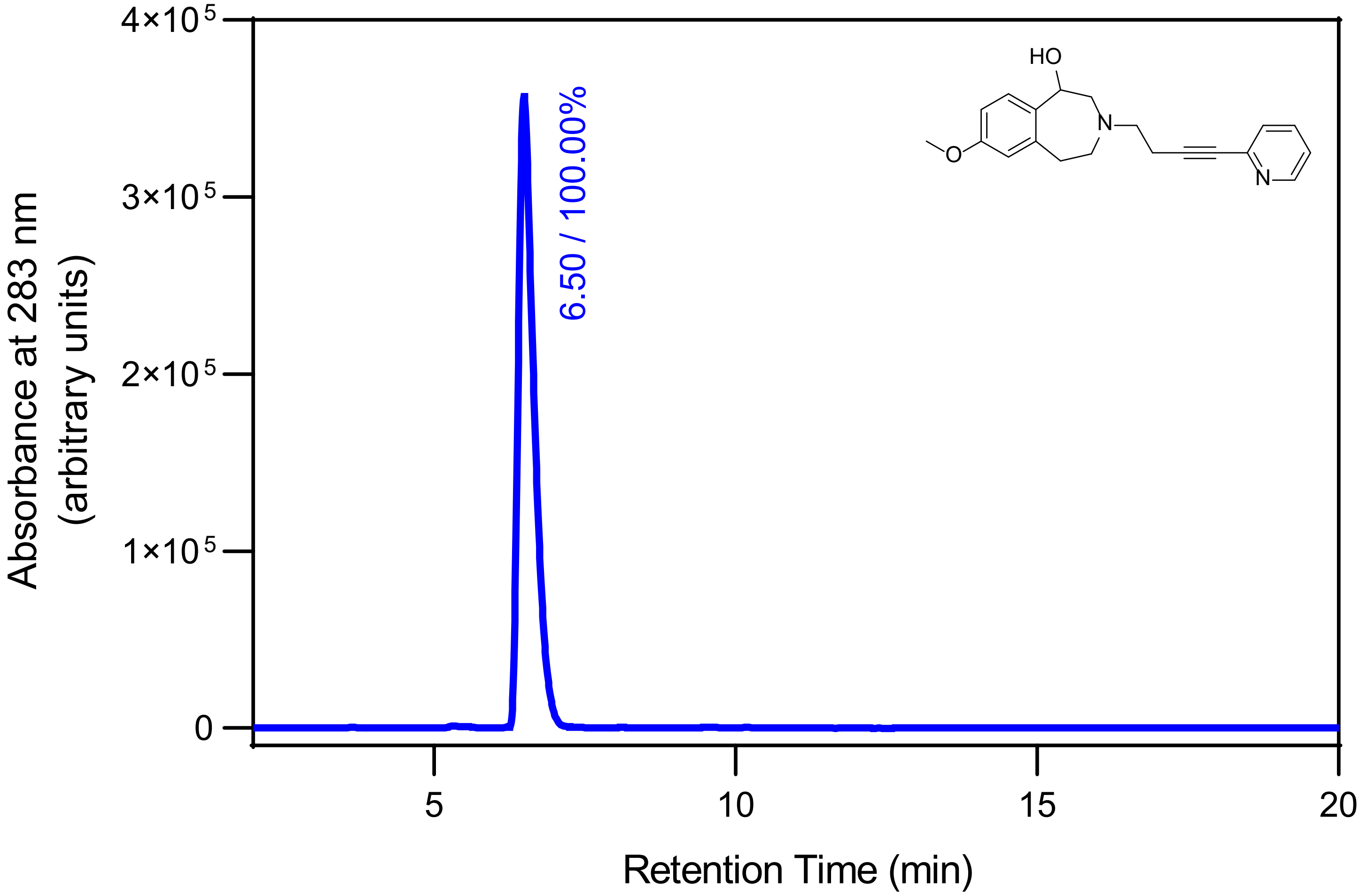


**Supplementary Figure 46.** Analytical HPLC chromatogram for **L11** (general method B).

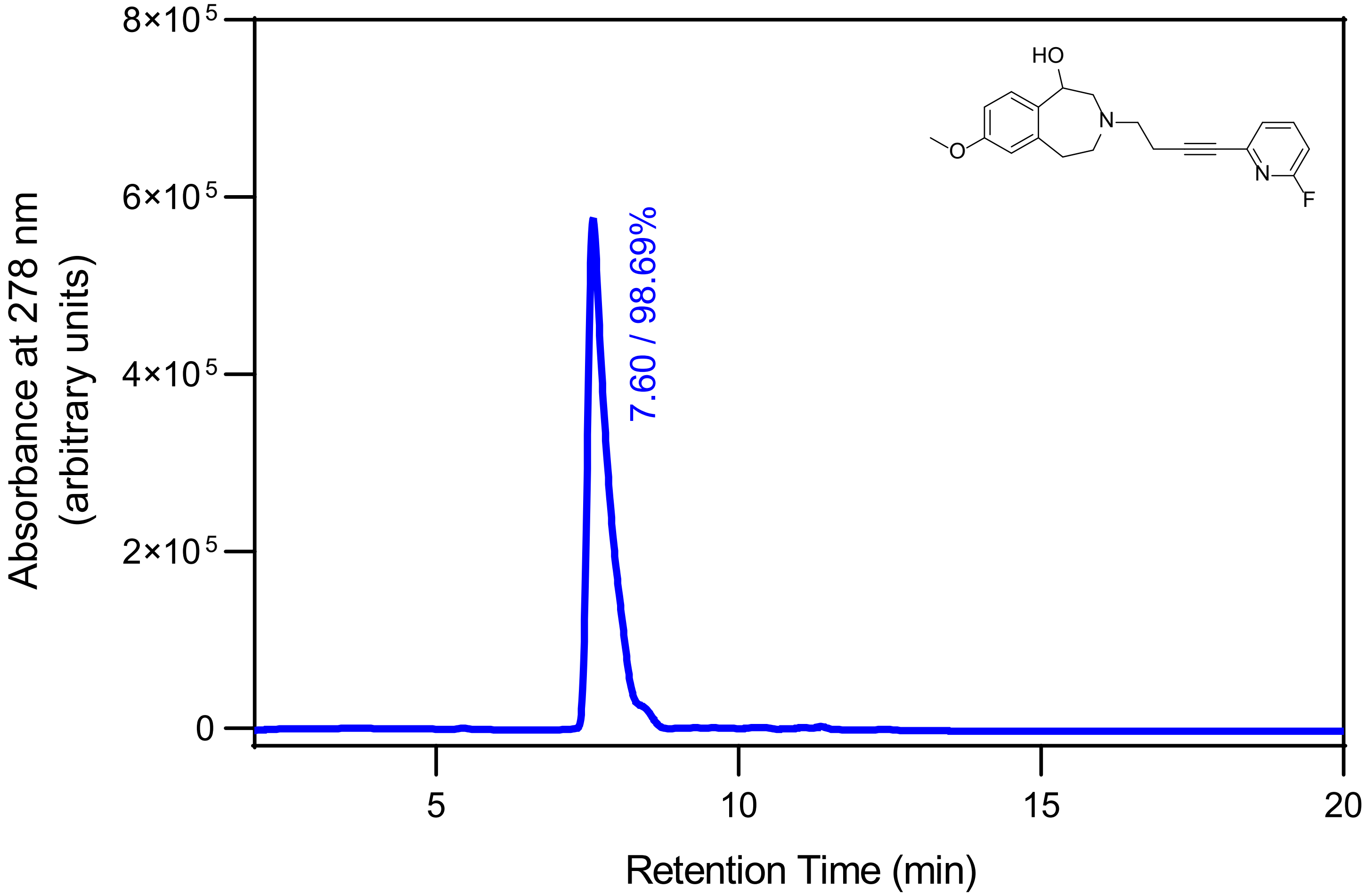


**Supplementary Figure 47.** Analytical HPLC chromatogram for **L12** (general method B).

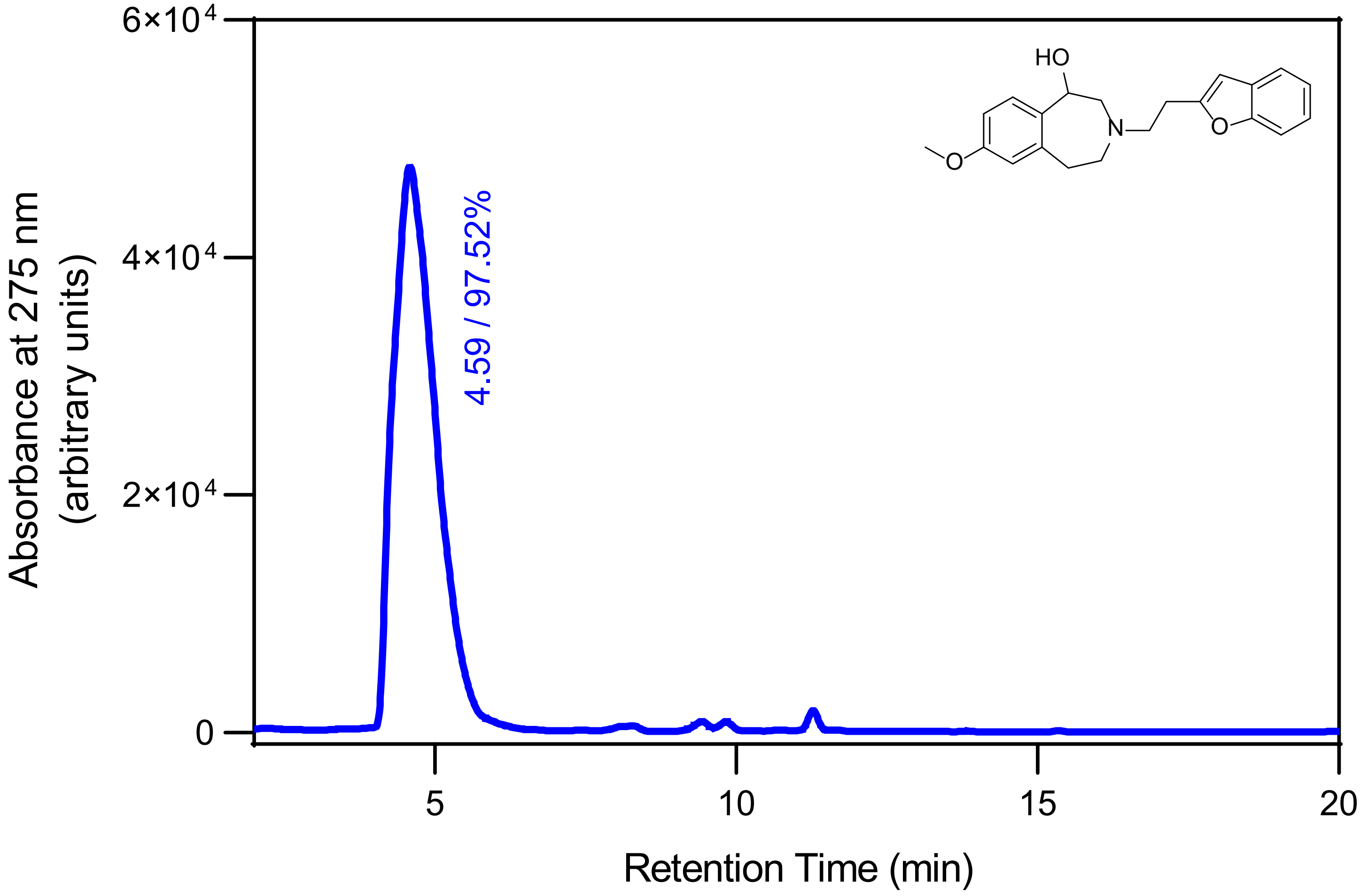


**Supplementary Figure 48.** Analytical HPLC chromatogram for **L13** (general method A).

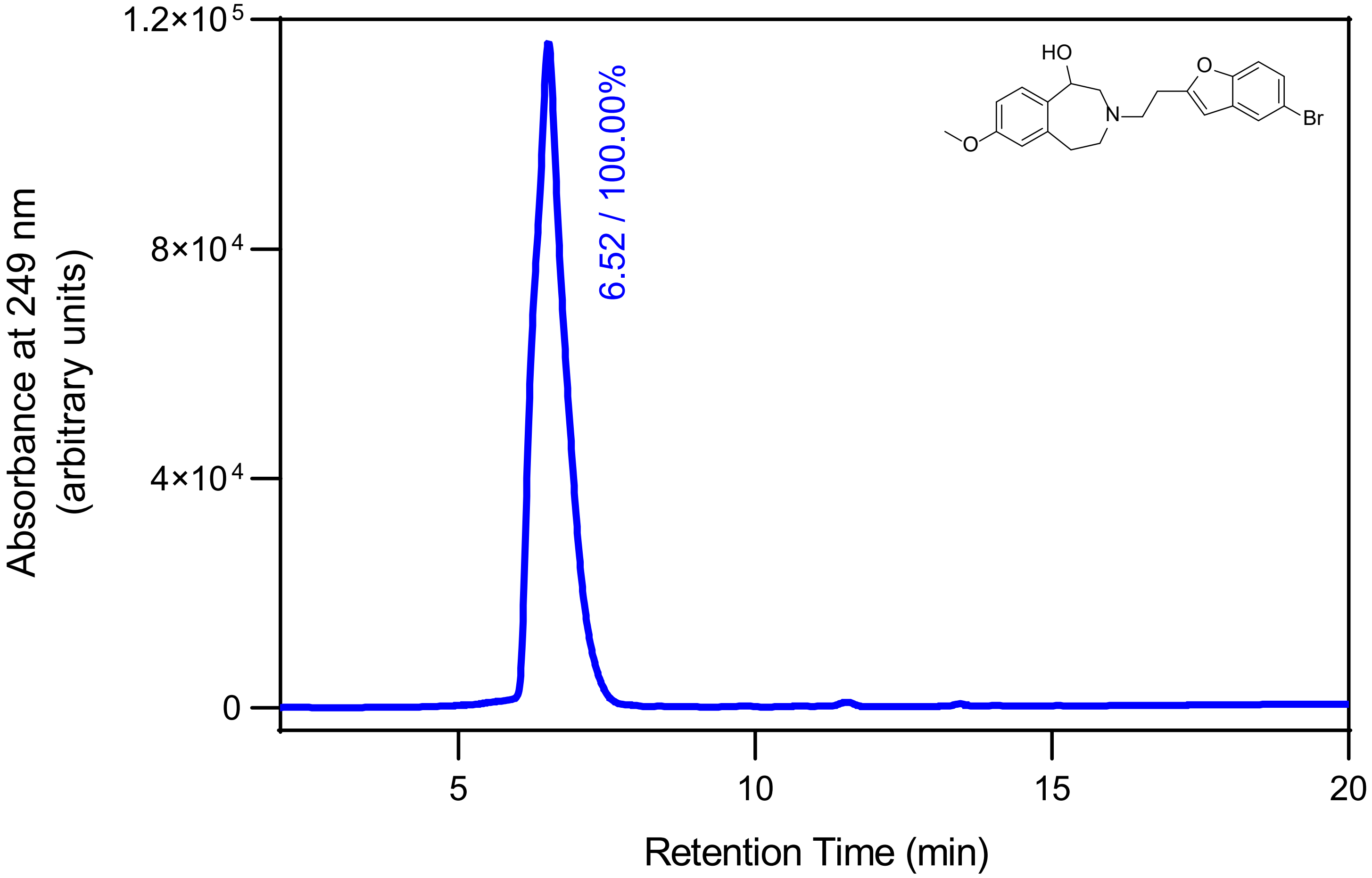


**Supplementary Figure 49.** Analytical HPLC chromatogram for **L14** (general method A).

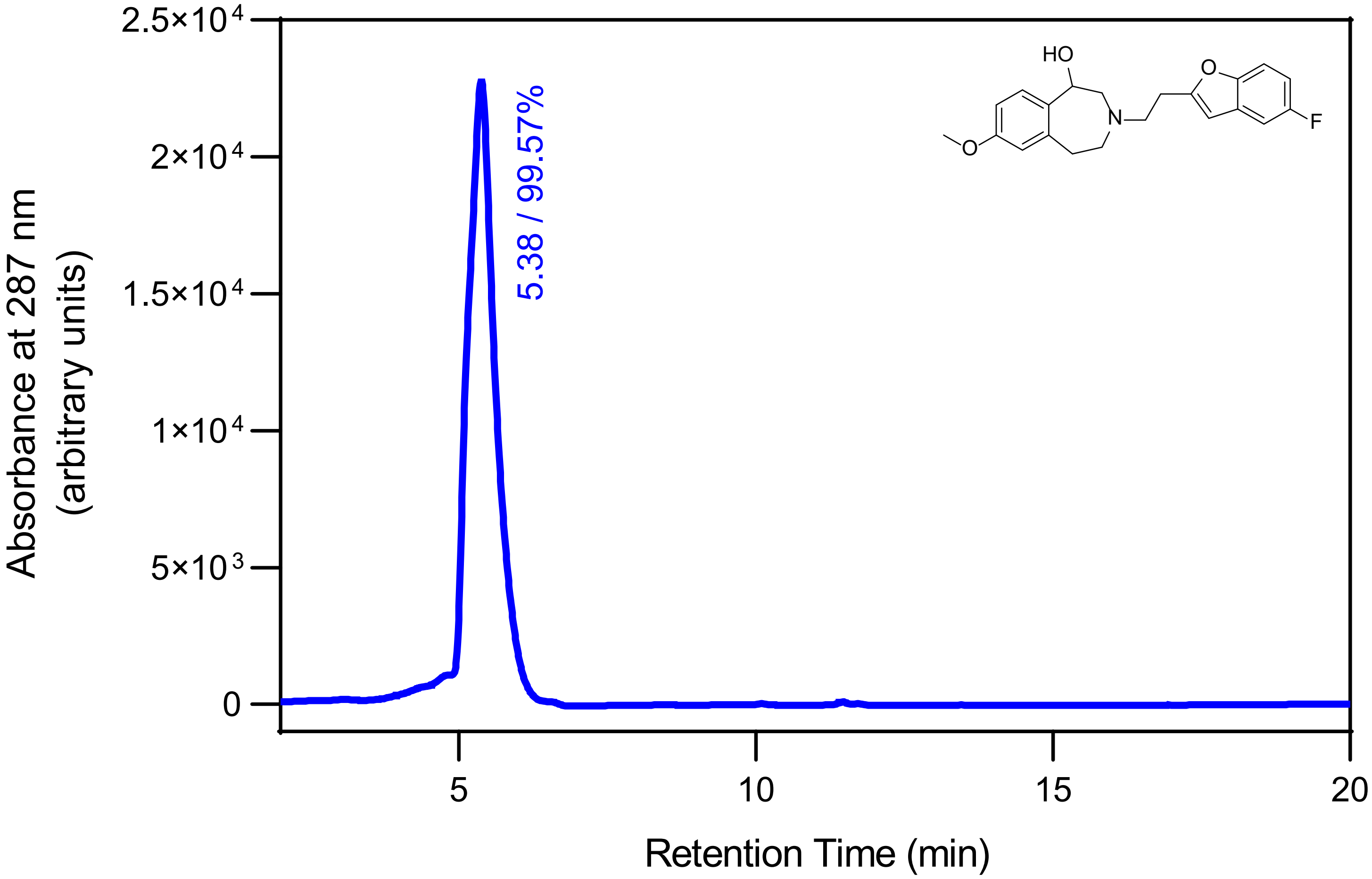


**Supplementary Figure 50.** Analytical HPLC chromatogram for **L15** (general method A).

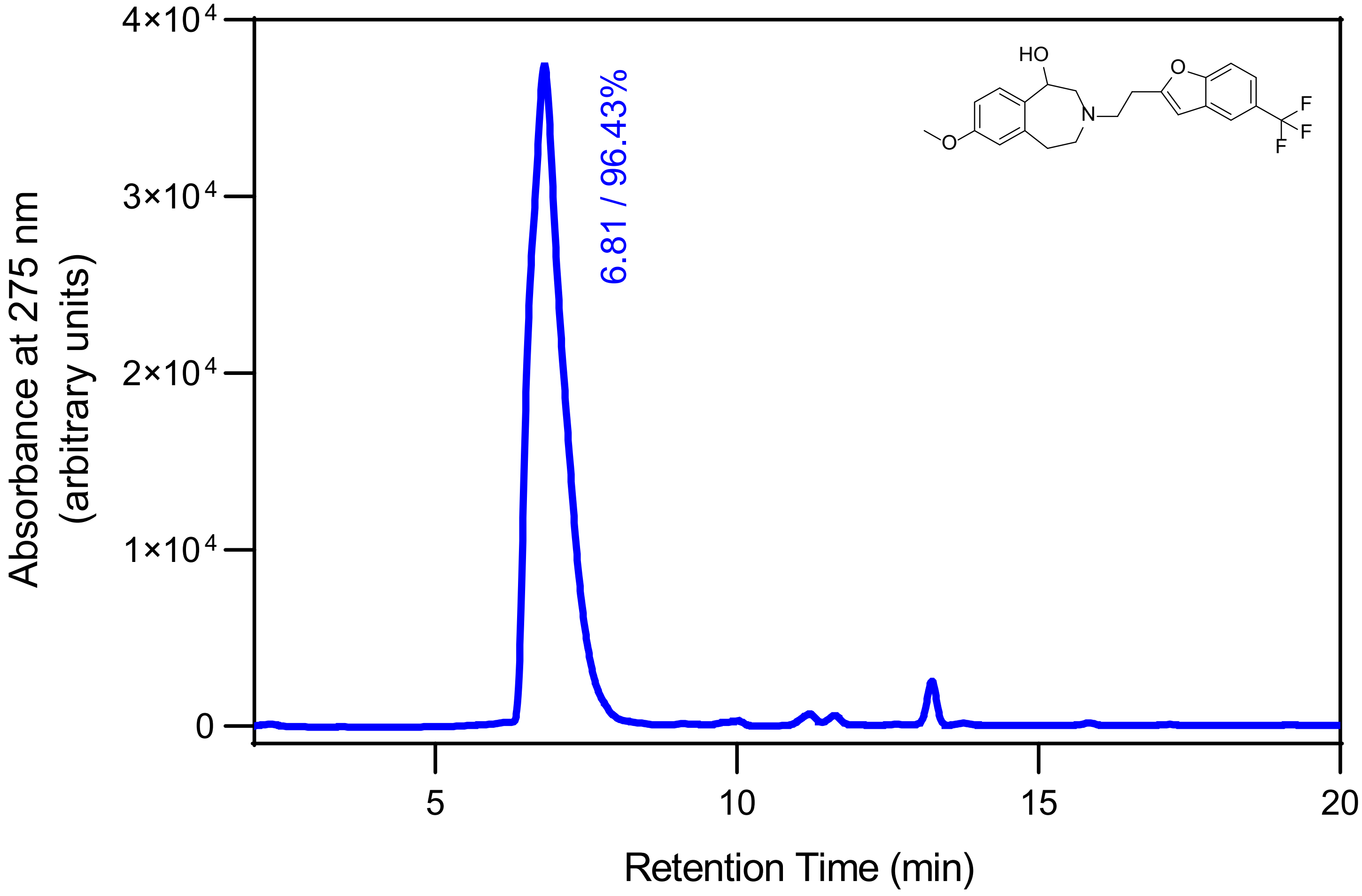


**Supplementary Figure 51.** Analytical HPLC chromatogram for **L16** (general method A).

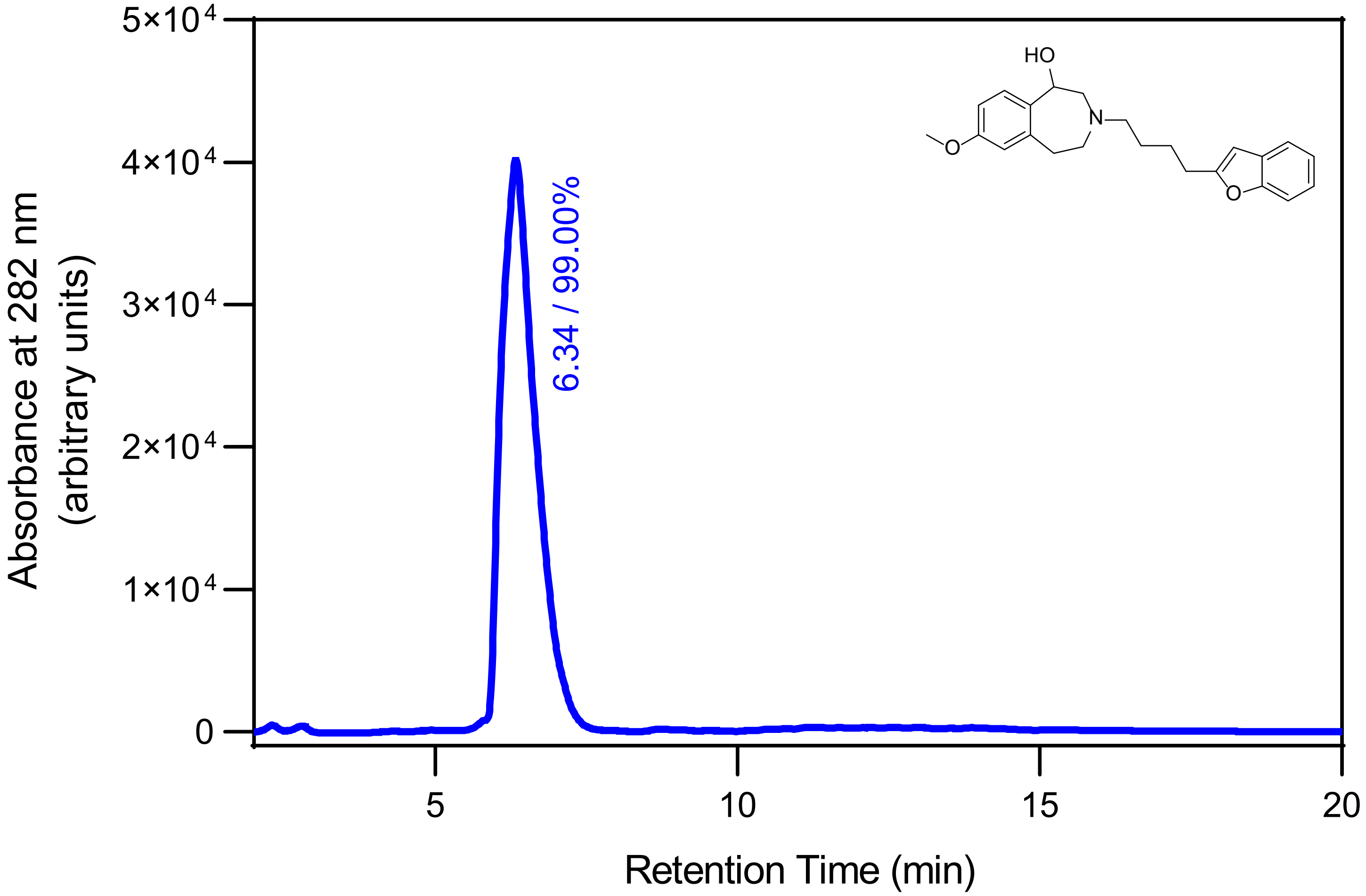


**Supplementary Figure 52.** Analytical HPLC chromatogram for **L17** (general method A).

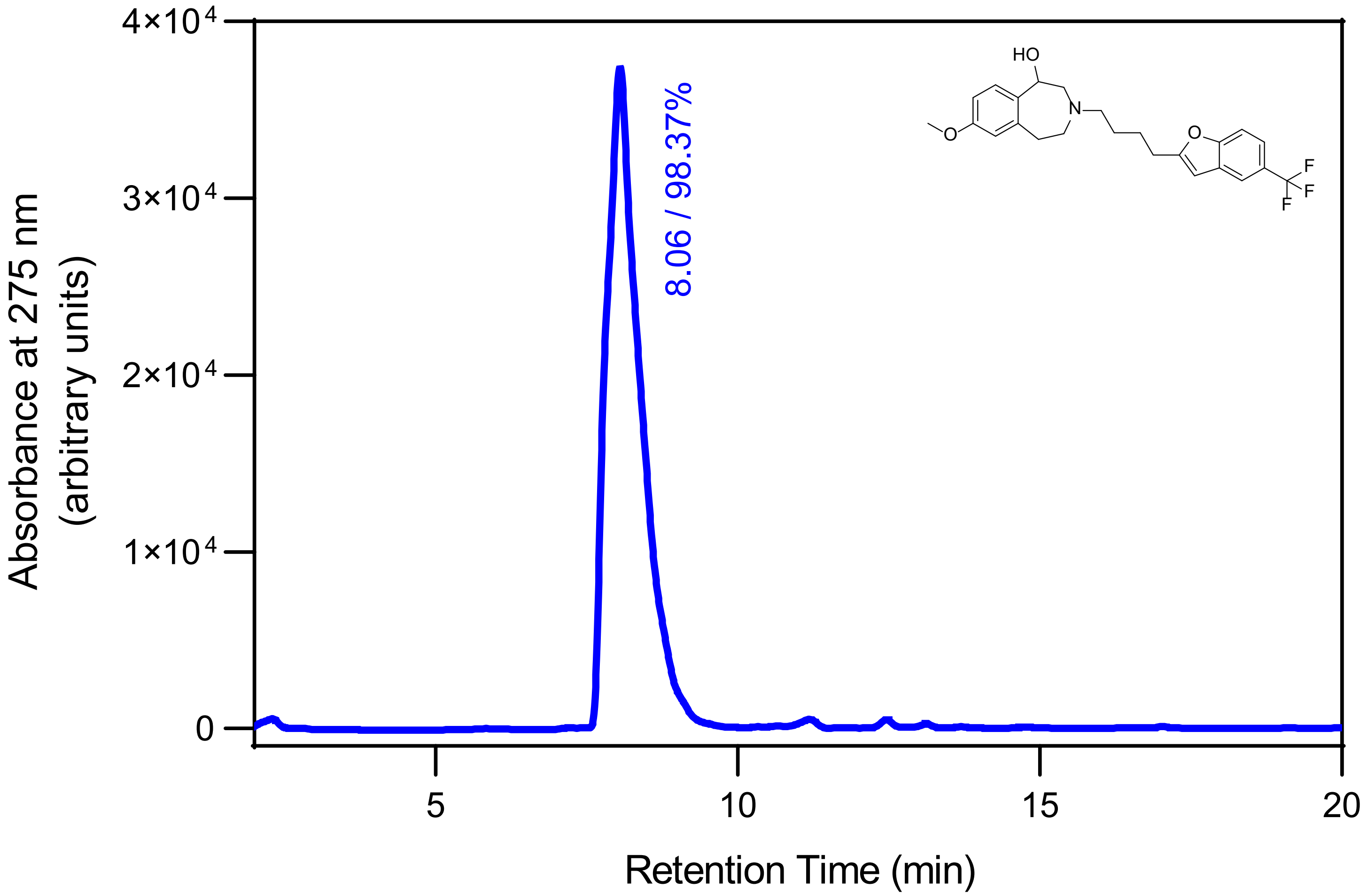


**Supplementary Figure 53.** Analytical HPLC chromatogram for **L18** (general method A).

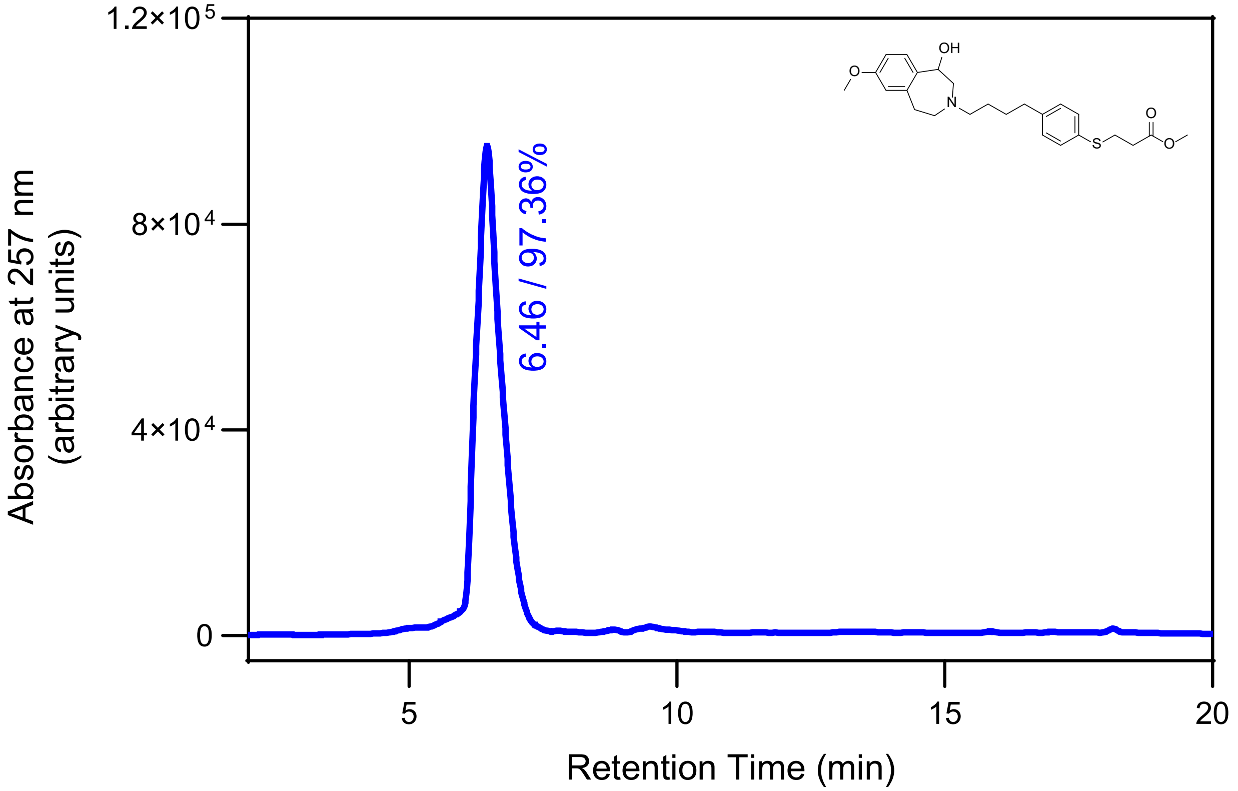


**Supplementary Figure 54.** Analytical HPLC chromatogram for **L19** (general method A).

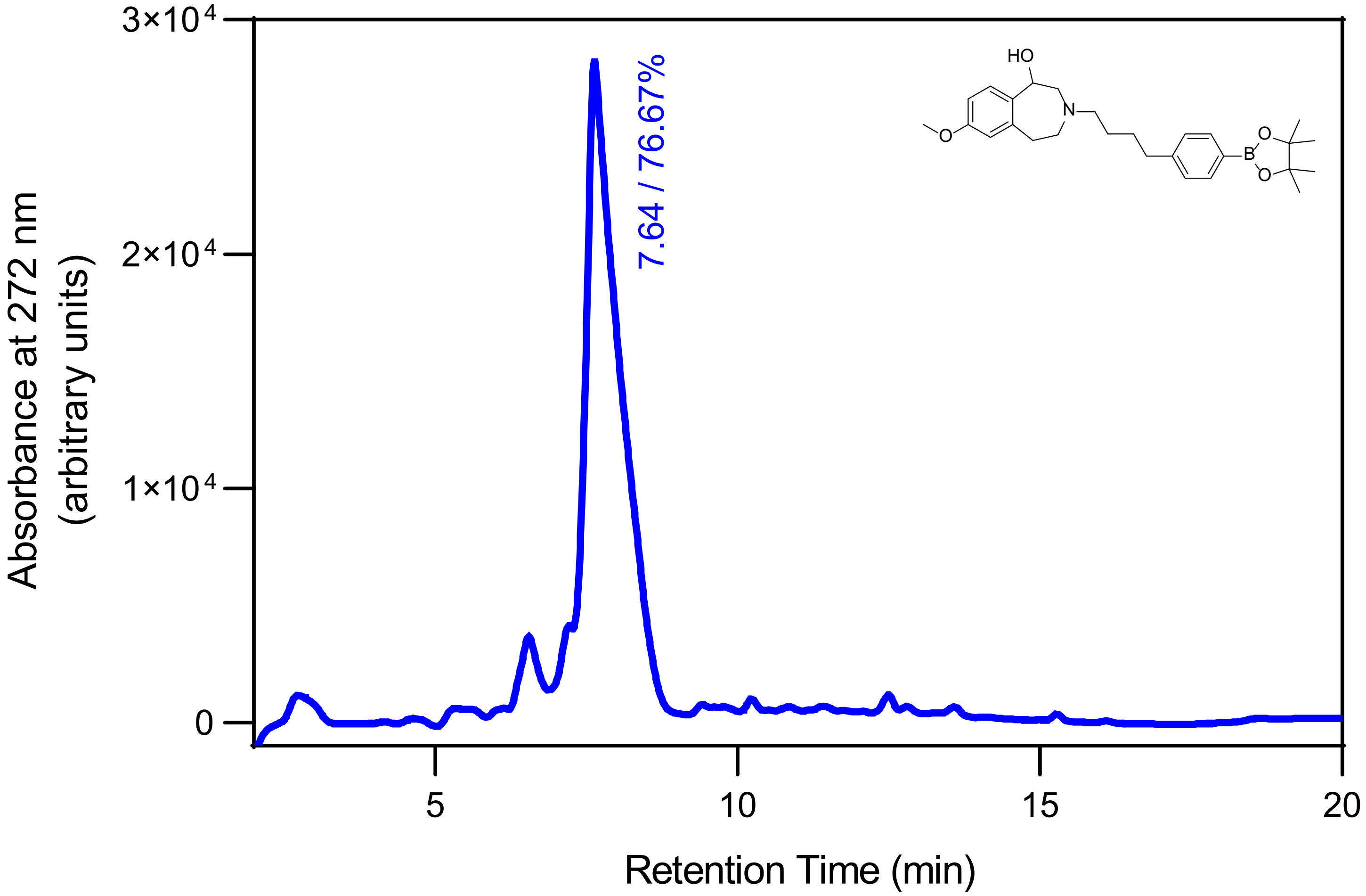


**Supplementary Figure 55.** Analytical HPLC chromatogram for **L20** (general method A).

## 5. NMR Spectra of Ligands L1–L9 and L11–L20

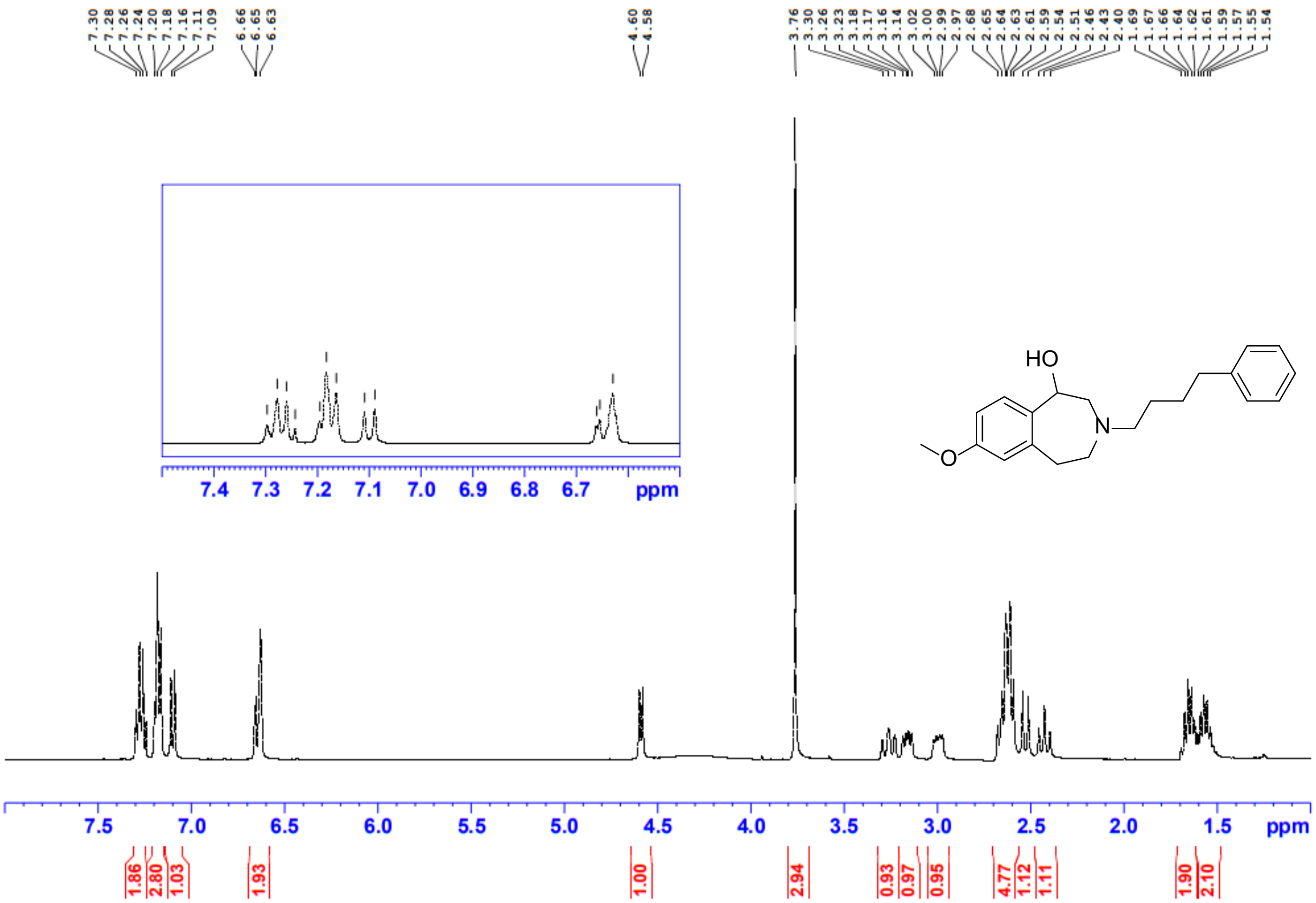

**Supplementary Figure 56.** $^{1}$H NMR Spectrum of **L1** (400 MHz, $CDCl_3$)

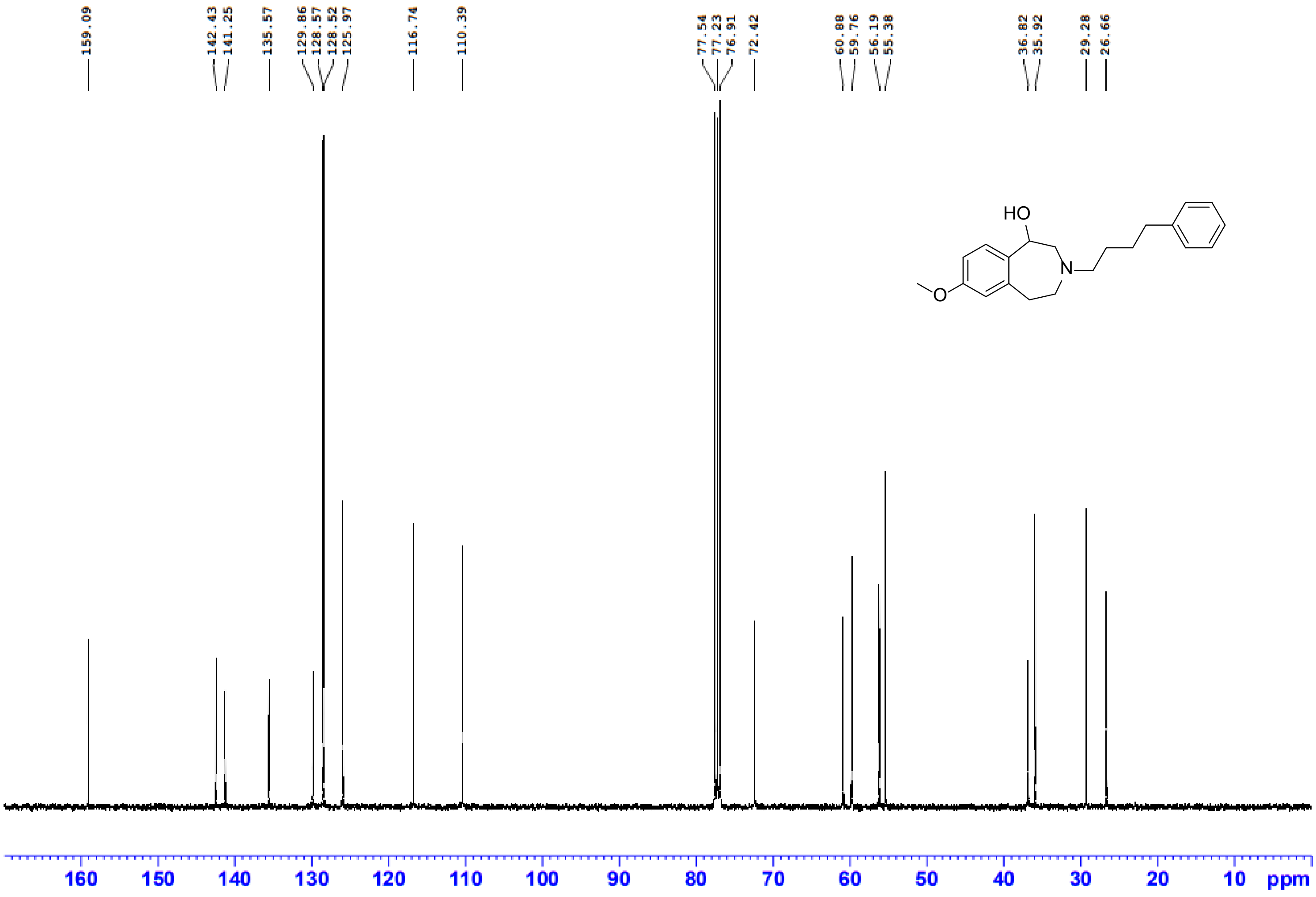


**Supplementary Figure 57.** $^{13}$C NMR Spectrum of **L1** (101 MHz, $CDCl_3$)

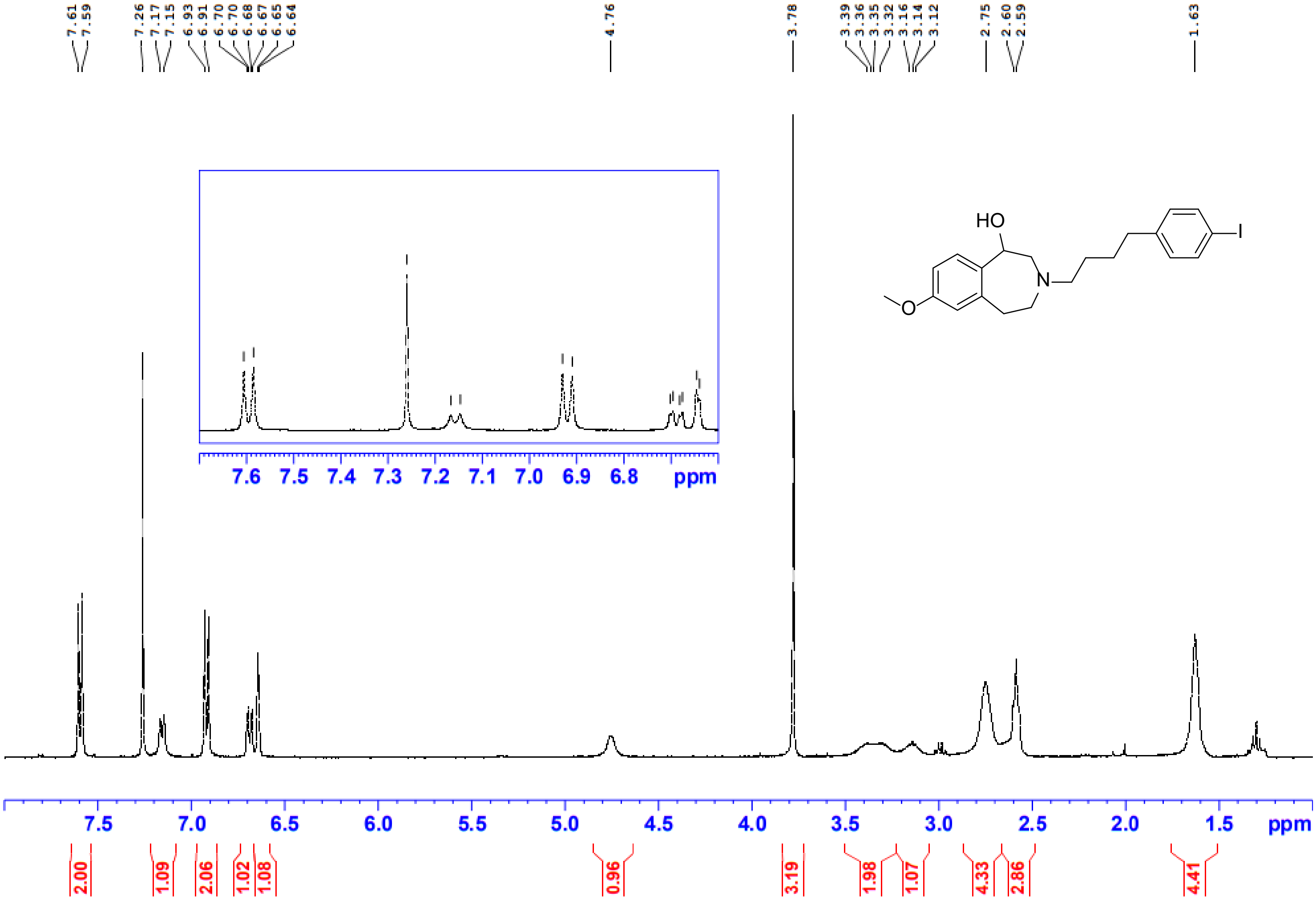


**Supplementary Figure 58.** $^{1}$H NMR Spectrum of **L2** (400 MHz, $CDCl_3$)

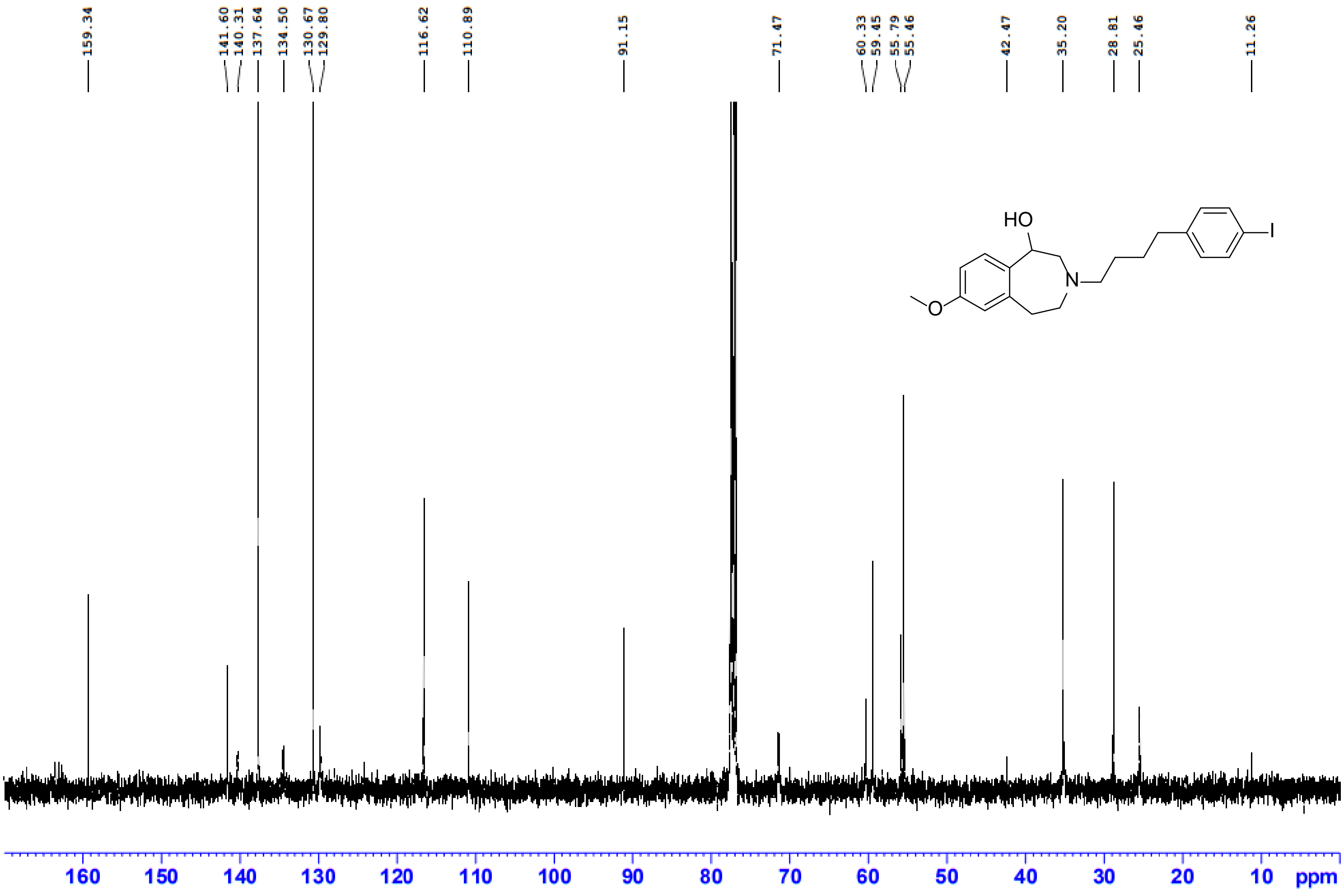


**Supplementary Figure 59.** $^{13}$C NMR Spectrum of **L2** (101 MHz, $CDCl_3$)

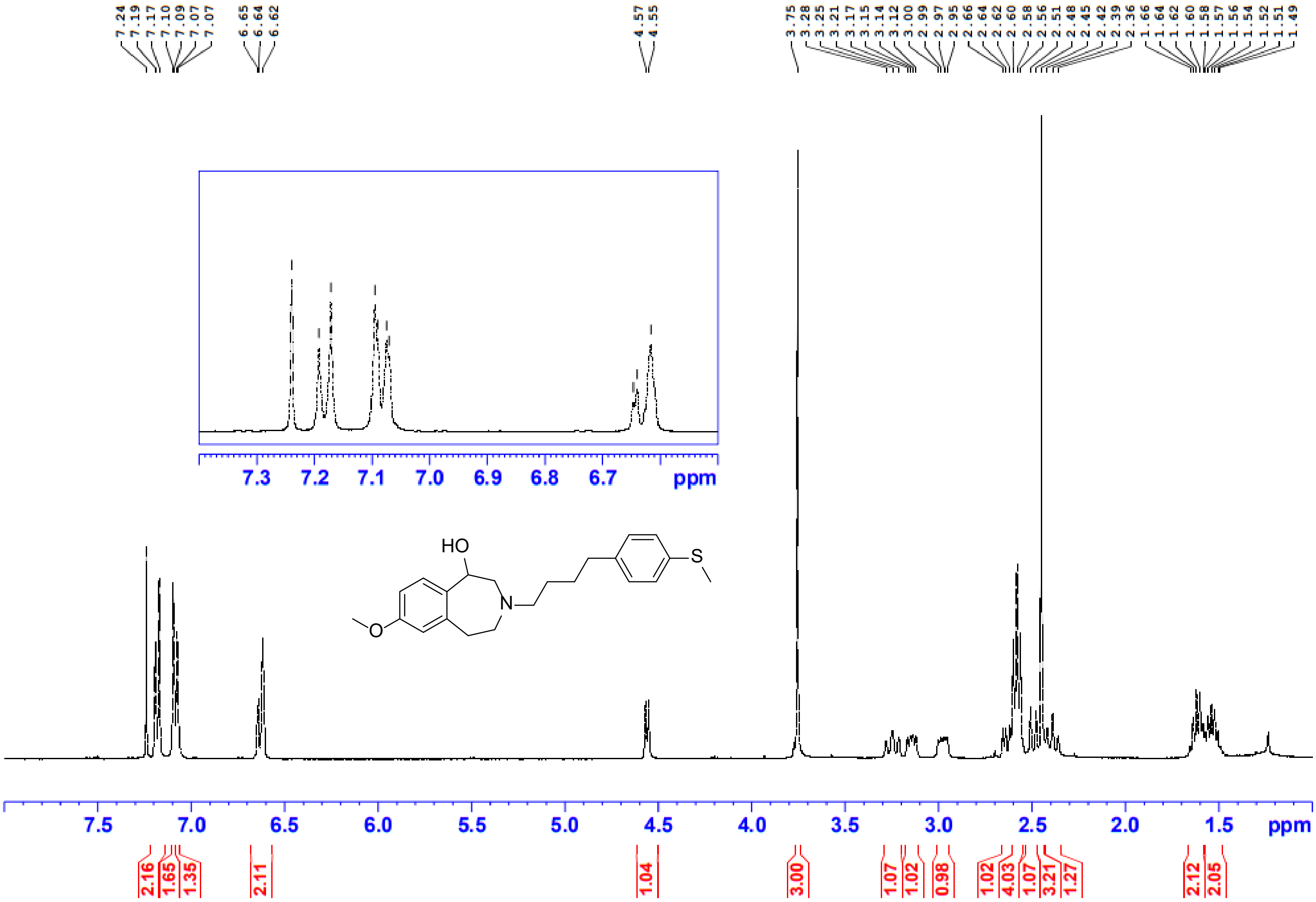

**Supplementary Figure 60.** $^1$H NMR Spectrum of **L3** (400 MHz, $CDCl_3$)

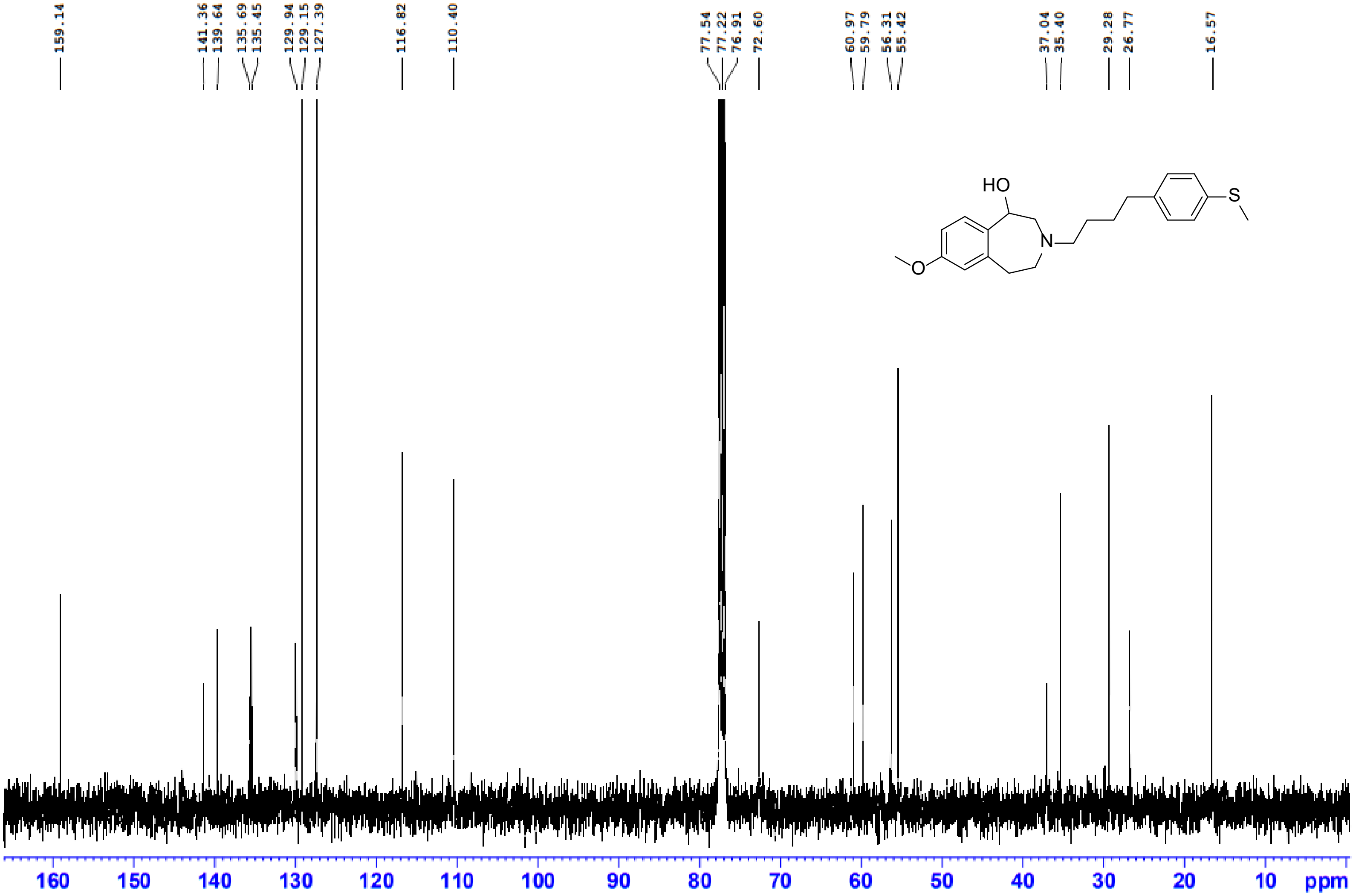


**Supplementary Figure 61.** $^{13}C$ NMR Spectrum of **L3** (101 MHz, $CDCl_3$)

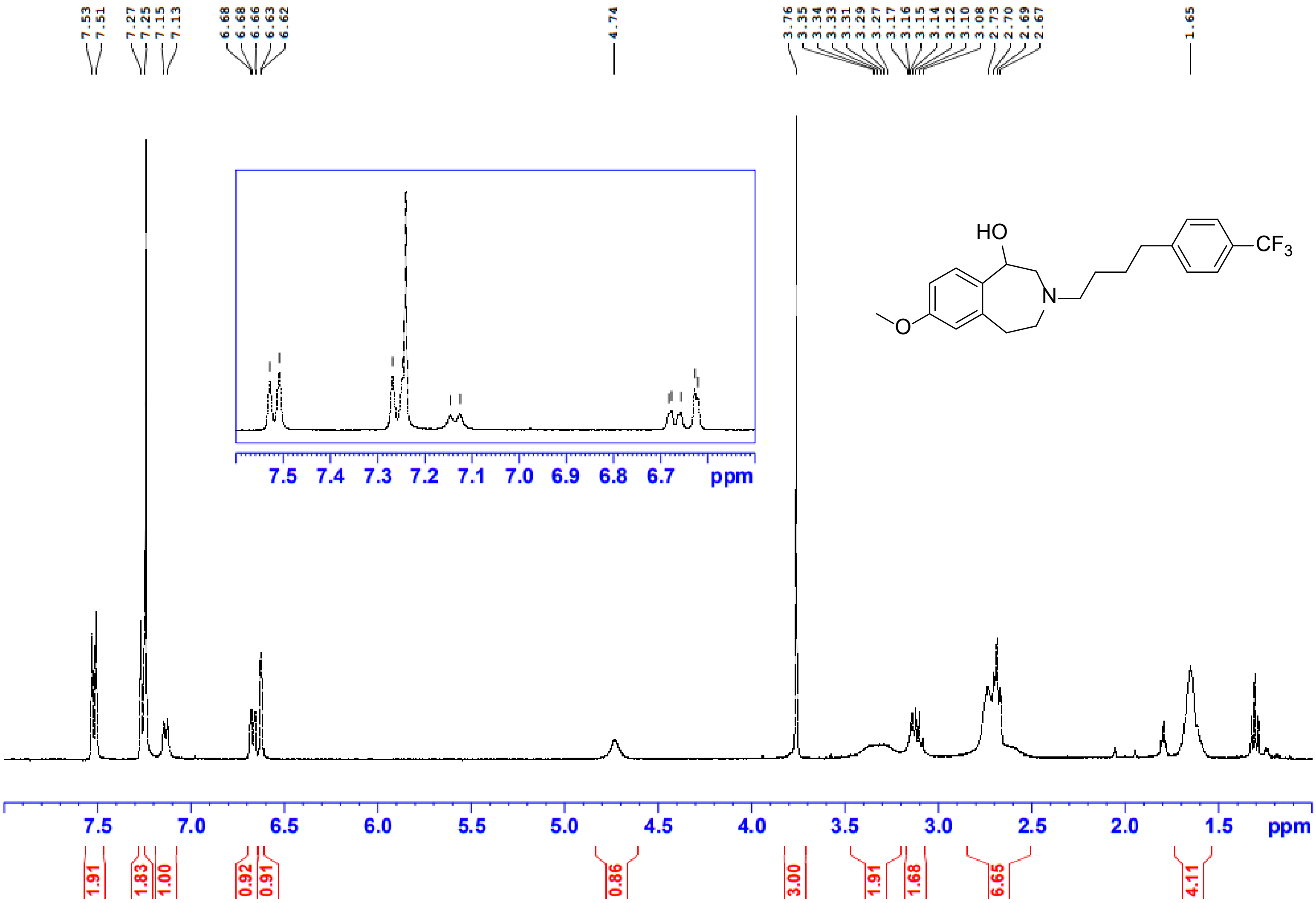

**Supplementary Figure 62.** $^{1}$H NMR Spectrum of **L4** (400 MHz, $CDCl_3$)

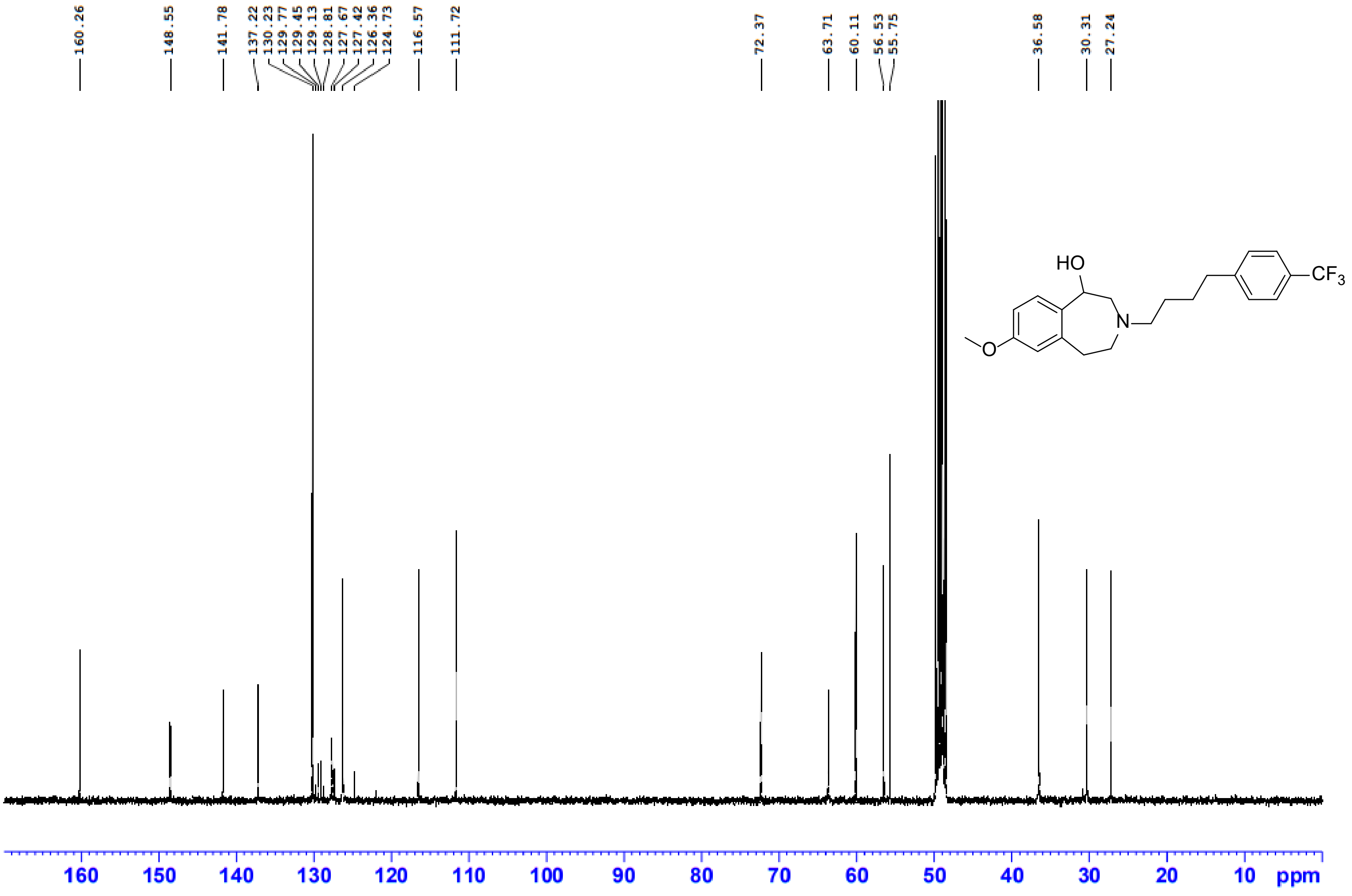


**Supplementary Figure 63.** $^{13}$C NMR Spectrum of **L4** (101 MHz, $CD_3OD$)

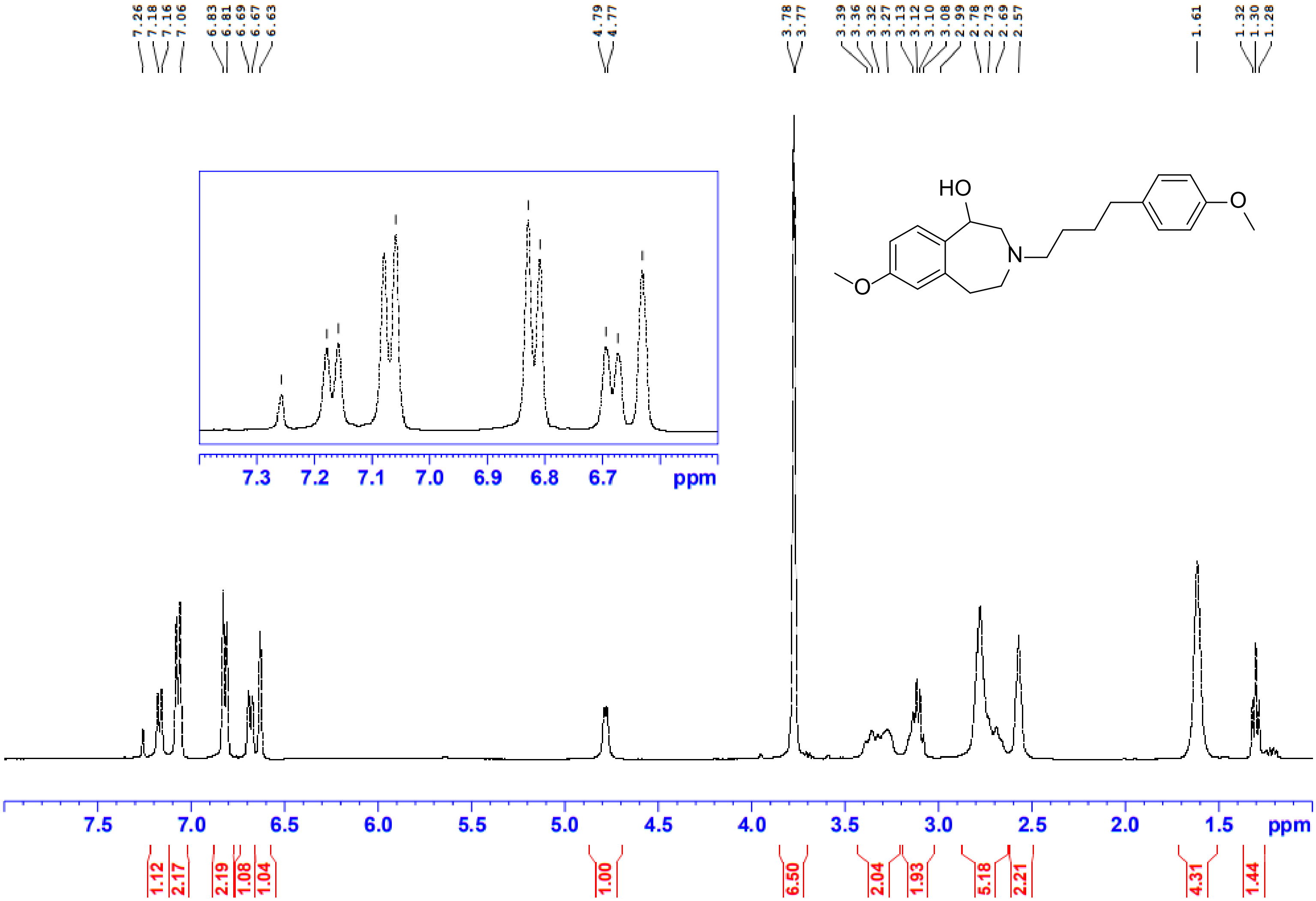

**Supplementary Figure 64.** $^{1}$H NMR Spectrum of **L5** (400 MHz, $CDCl_3$)

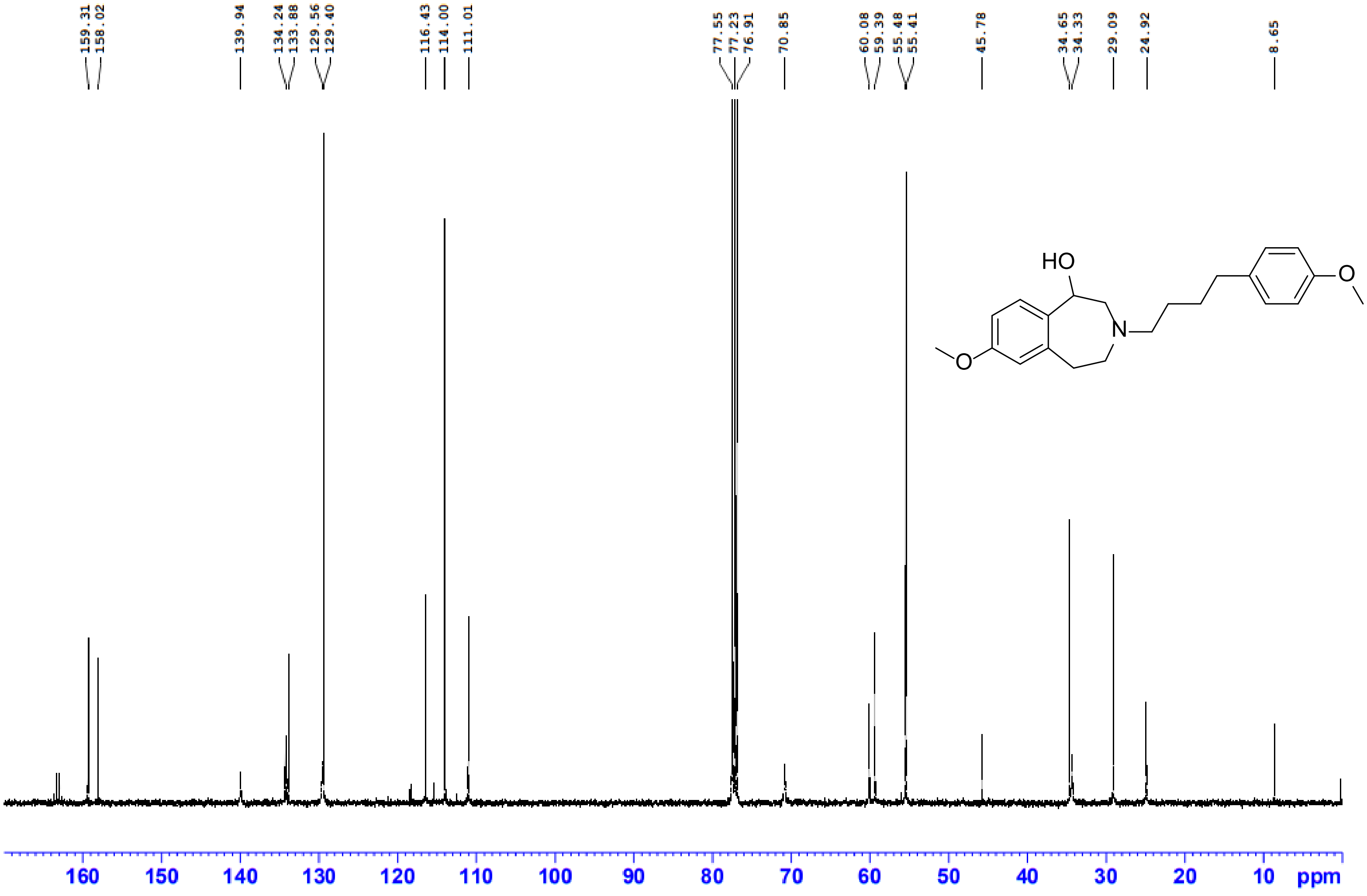


**Supplementary Figure 65.** $^{13}C$ NMR Spectrum of **L5** (101 MHz, $CDCl_3$)

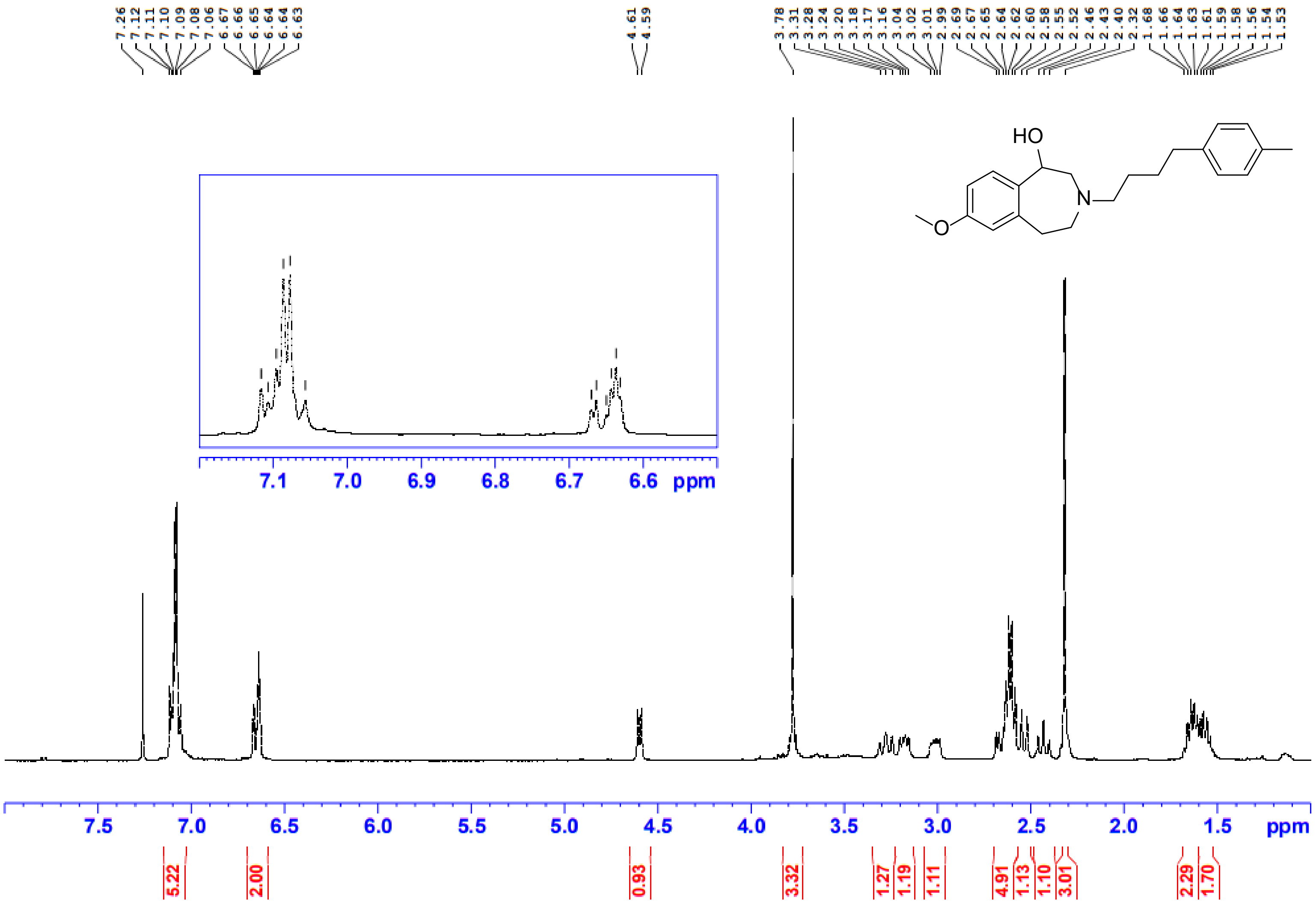

**Supplementary Figure 66.** $^{1}$H NMR Spectrum of **L6** (101 MHz, $CDCl_3$)

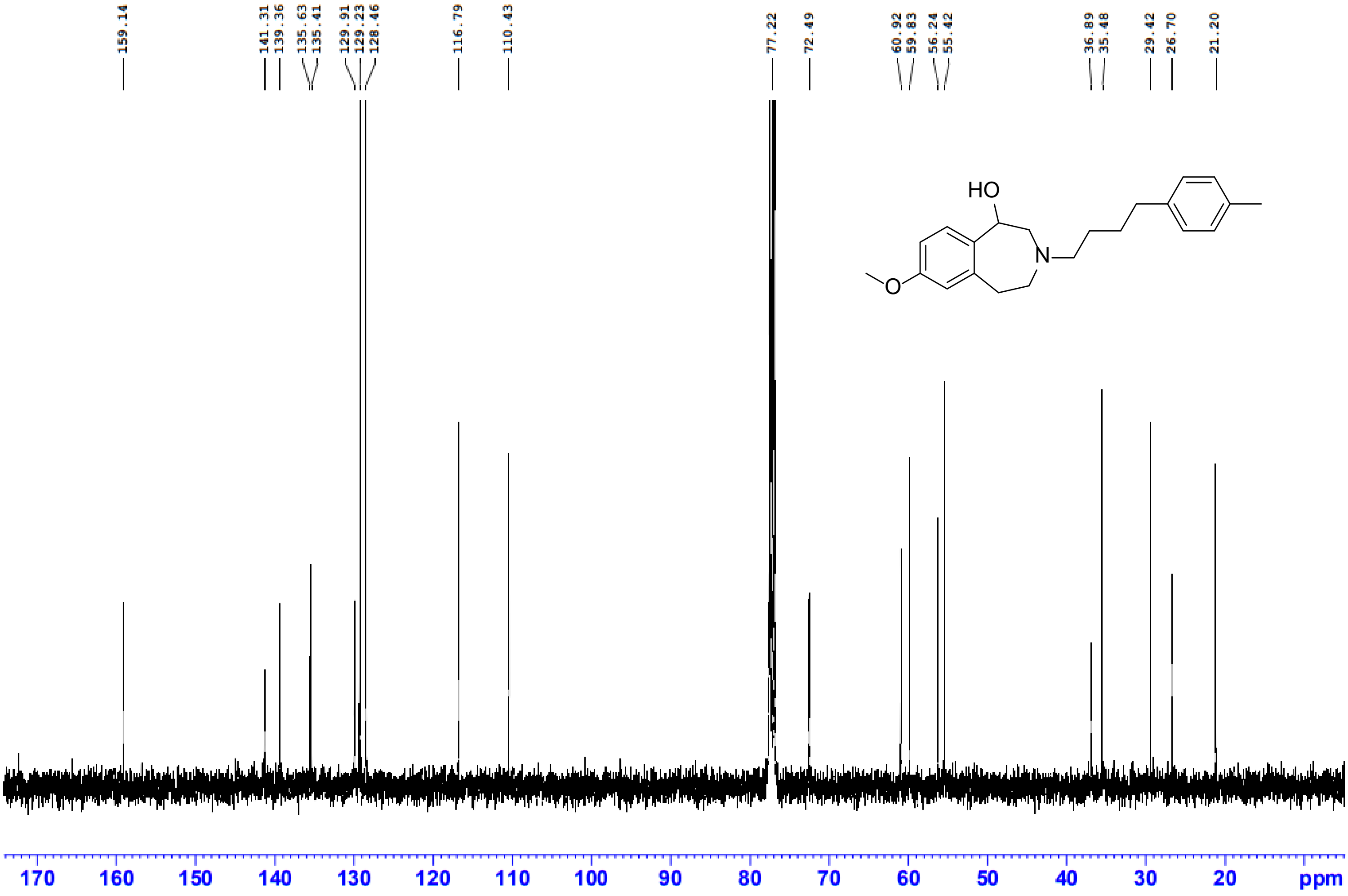


**Supplementary Figure 67.** $^{13}$C NMR Spectrum of **L6** (101 MHz, $CDCl_3$)

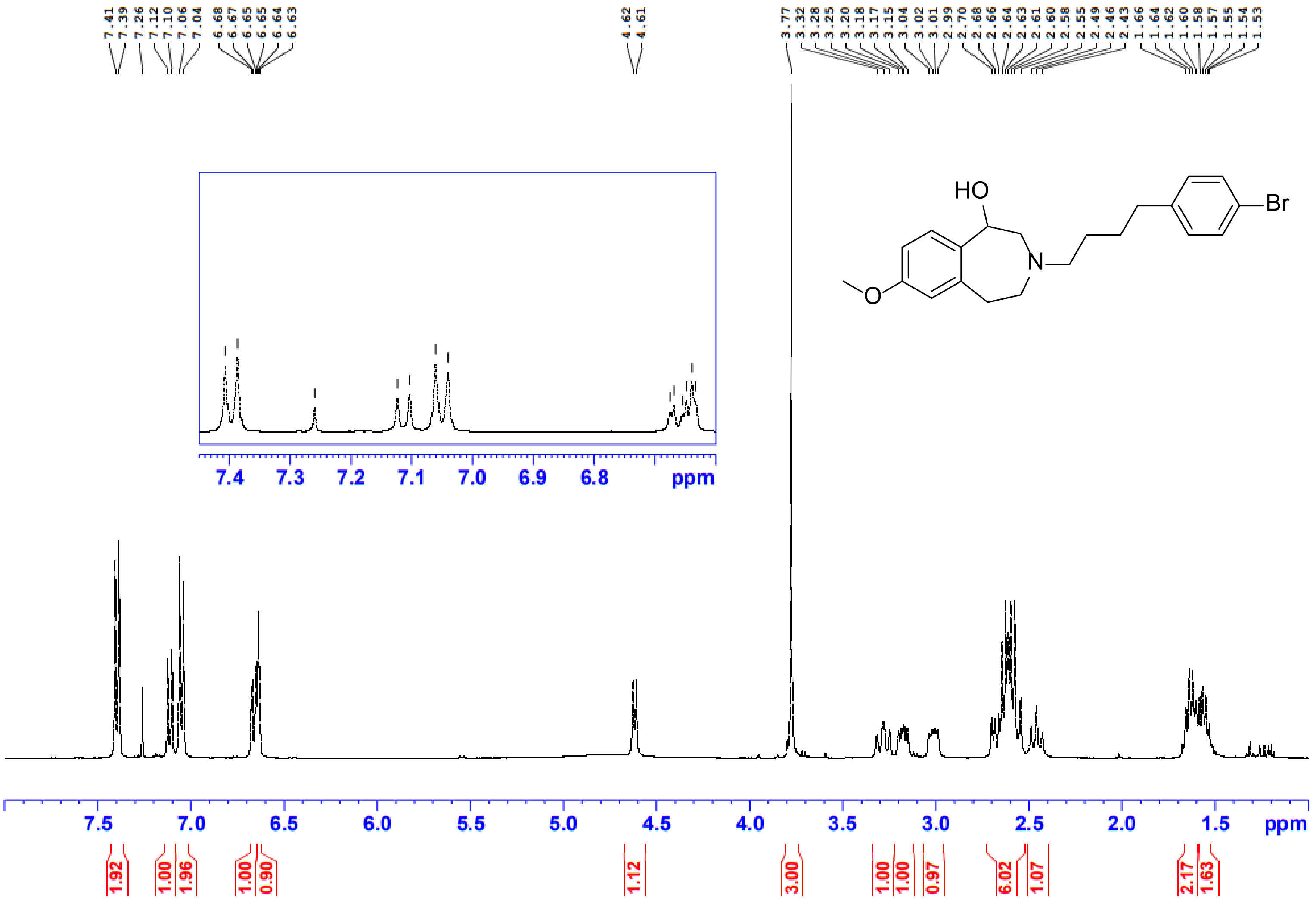

**Supplementary Figure 68.** $^{1}$H NMR Spectrum of **L7** (400 MHz, $CDCl_3$)

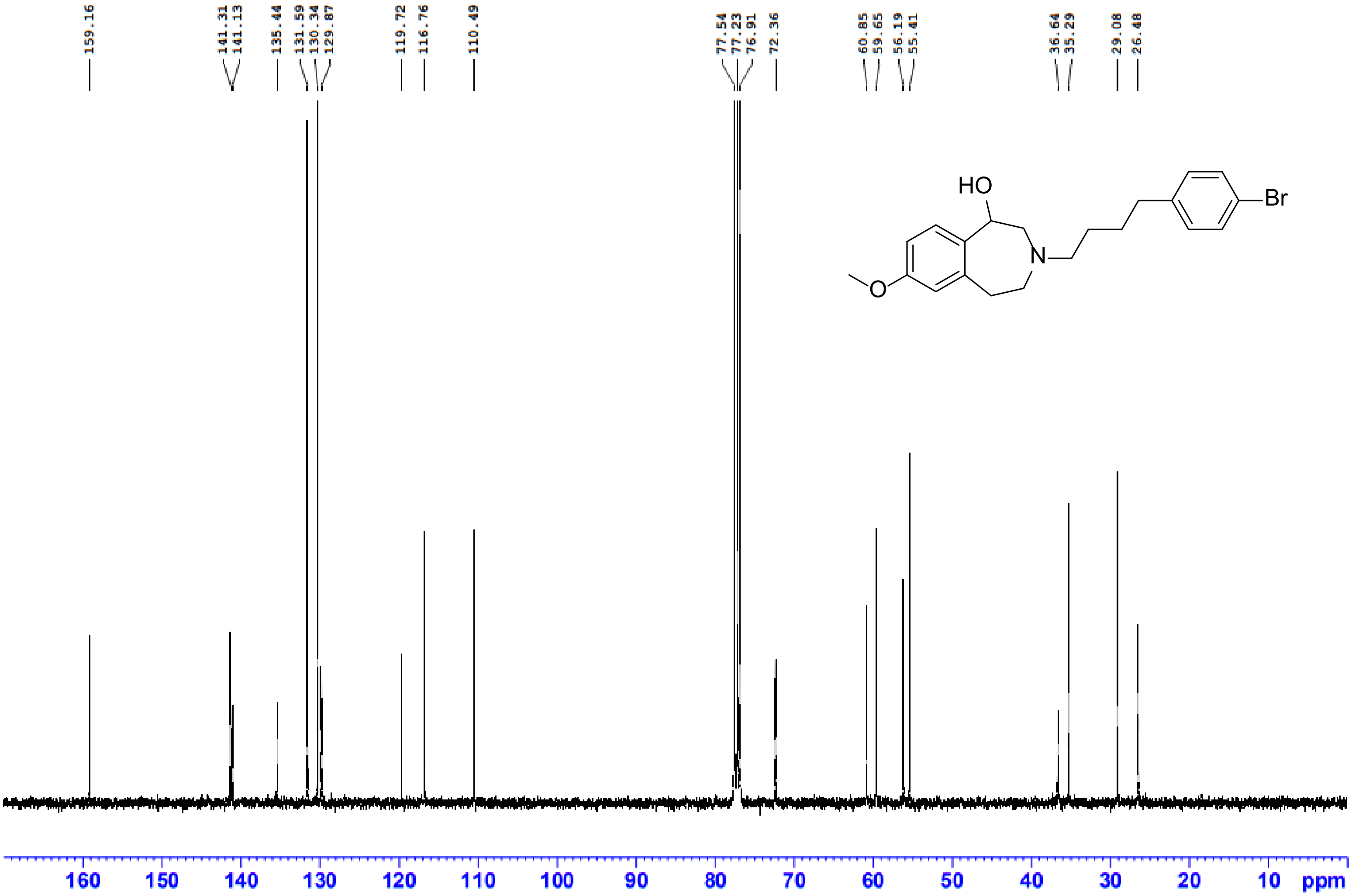


**Supplementary Figure 69.** $^{13}C$ NMR Spectrum of **L7** (101 MHz, $CDCl_3$)

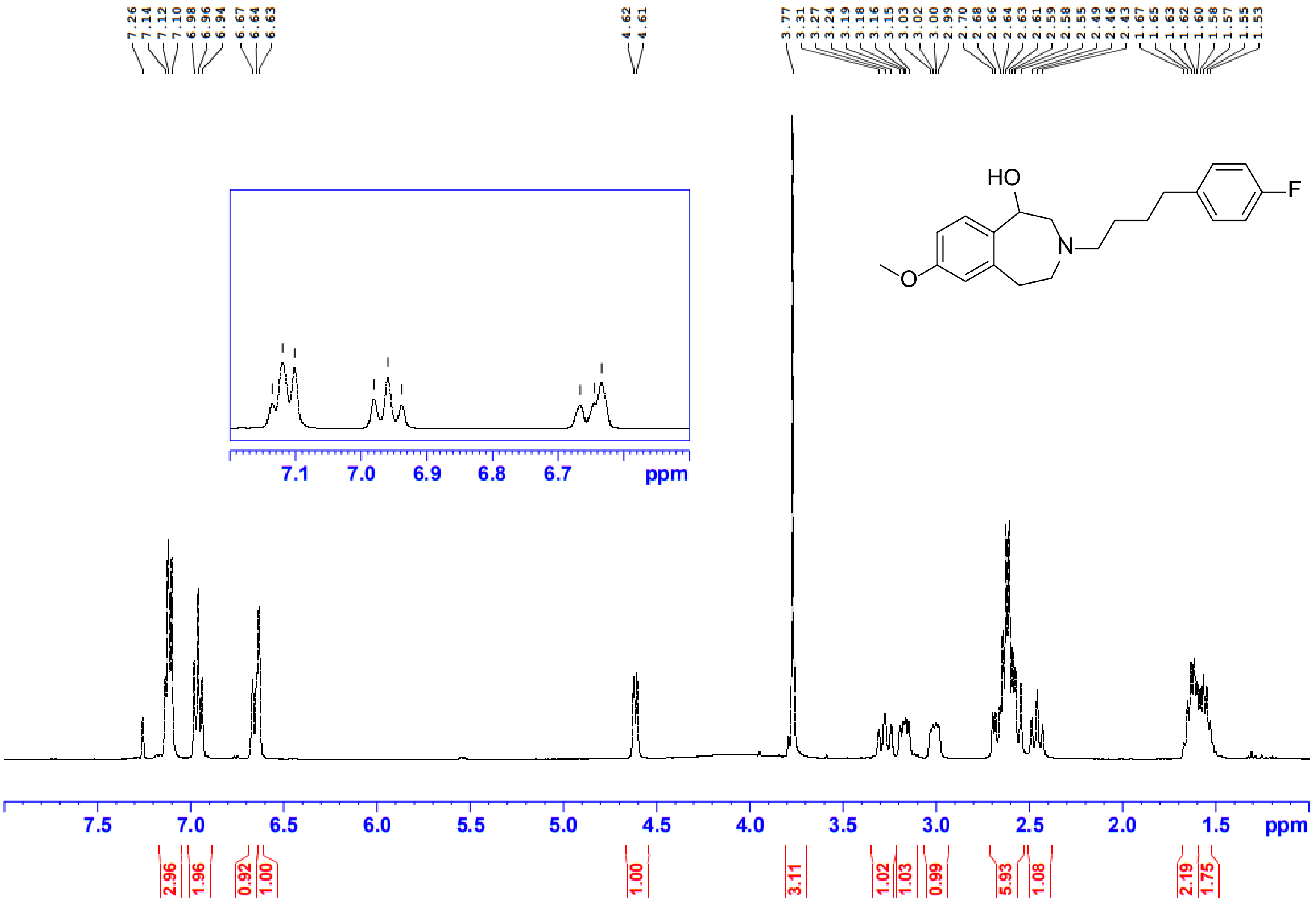

**Supplementary Figure 70.** $^{1}H$ NMR Spectrum of **L8** (400 MHz, $CDCl_3$)

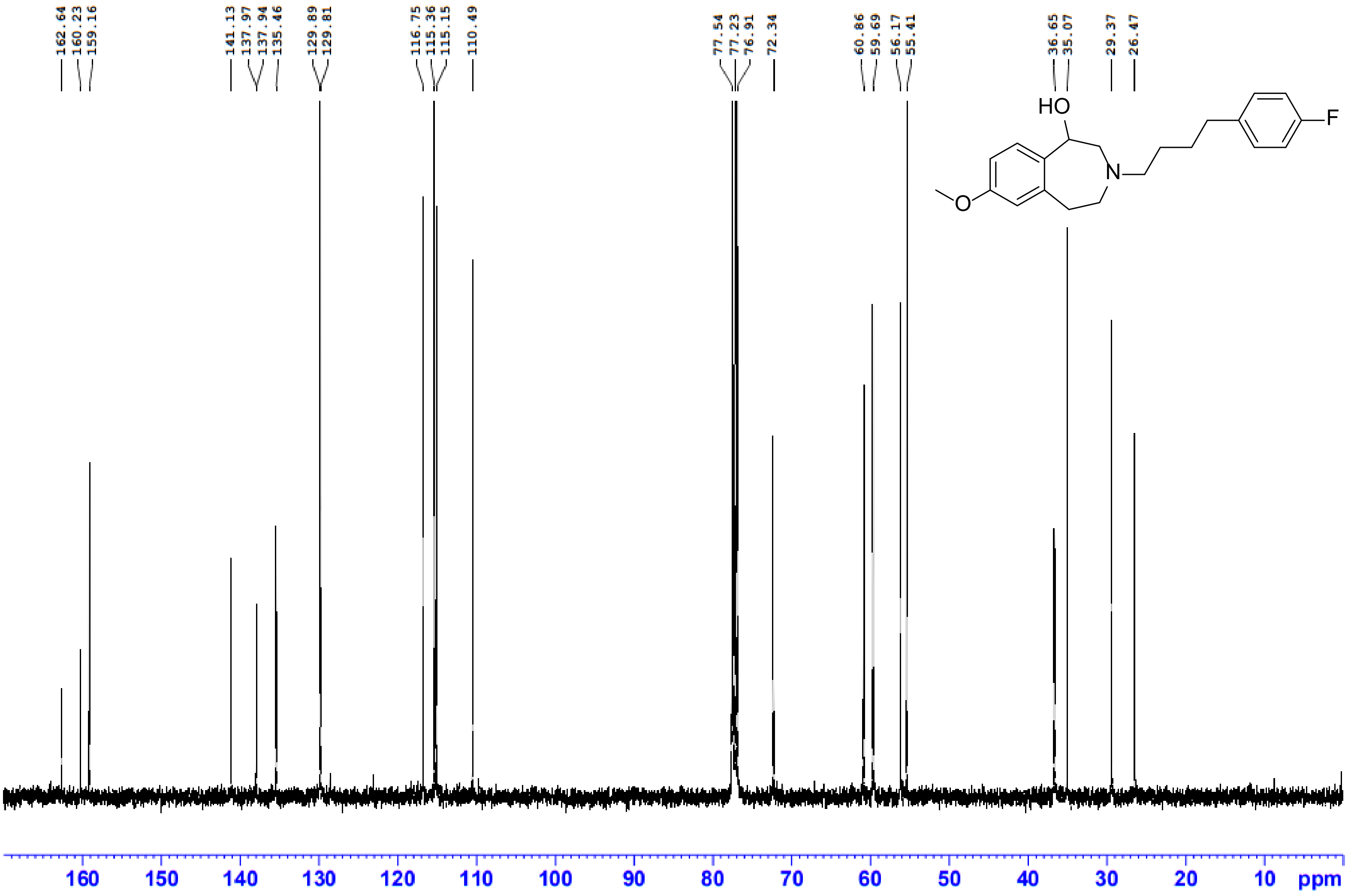


**Supplementary Figure 71.** $^{13}$C NMR Spectrum of **L8** (101 MHz, $CDCl_3$)

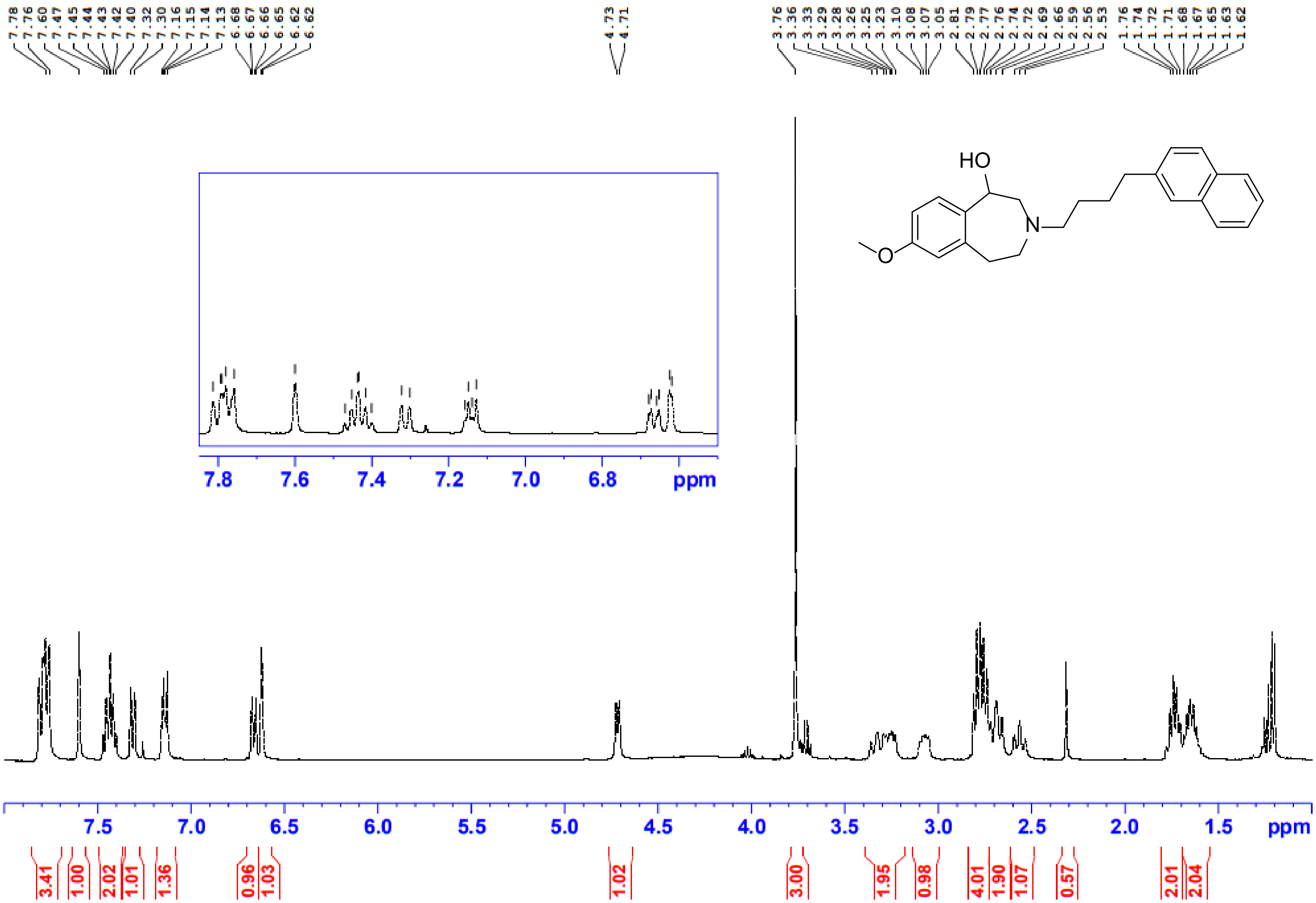

**Supplementary Figure 72.** $^1$H NMR Spectrum of **L9** (400 MHz, $CDCl_3$)

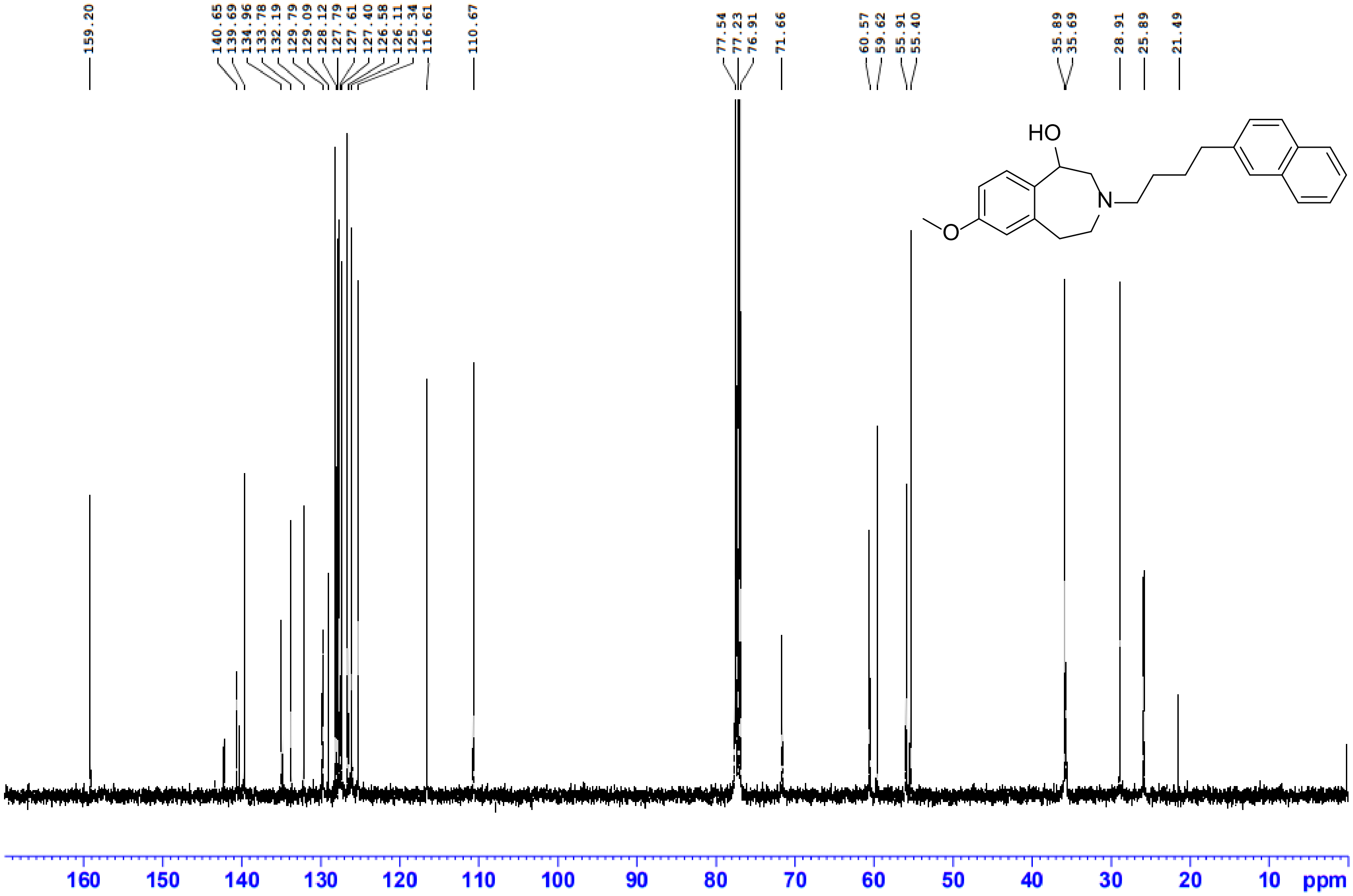


**Supplementary Figure 73.** $^{13}C$ NMR Spectrum of **L9** (101 MHz, $CDCl_3$)

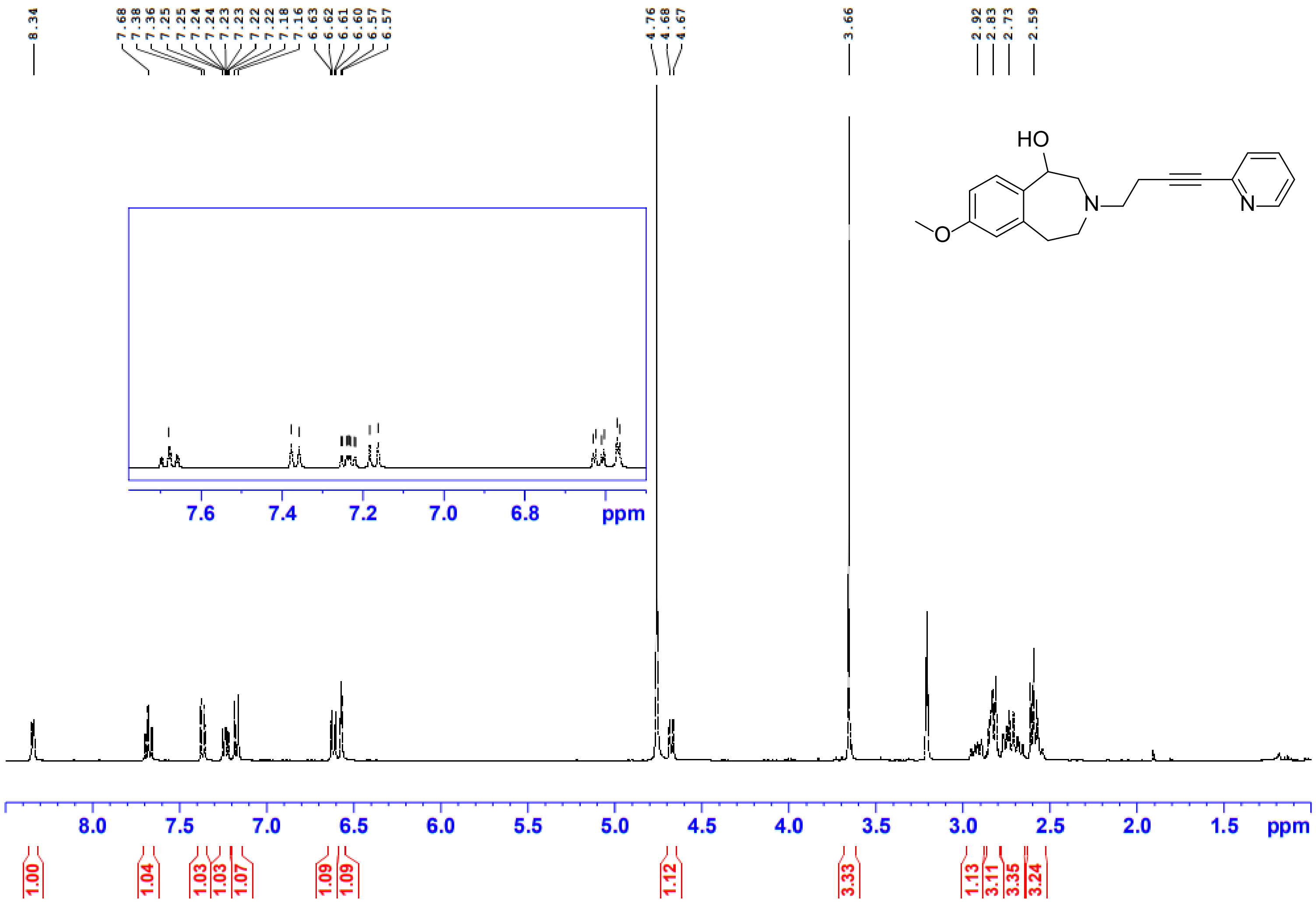


**Supplementary Figure 74.** $^{1}$H NMR Spectrum of **L11** (400 MHz, $CD_3OD$)

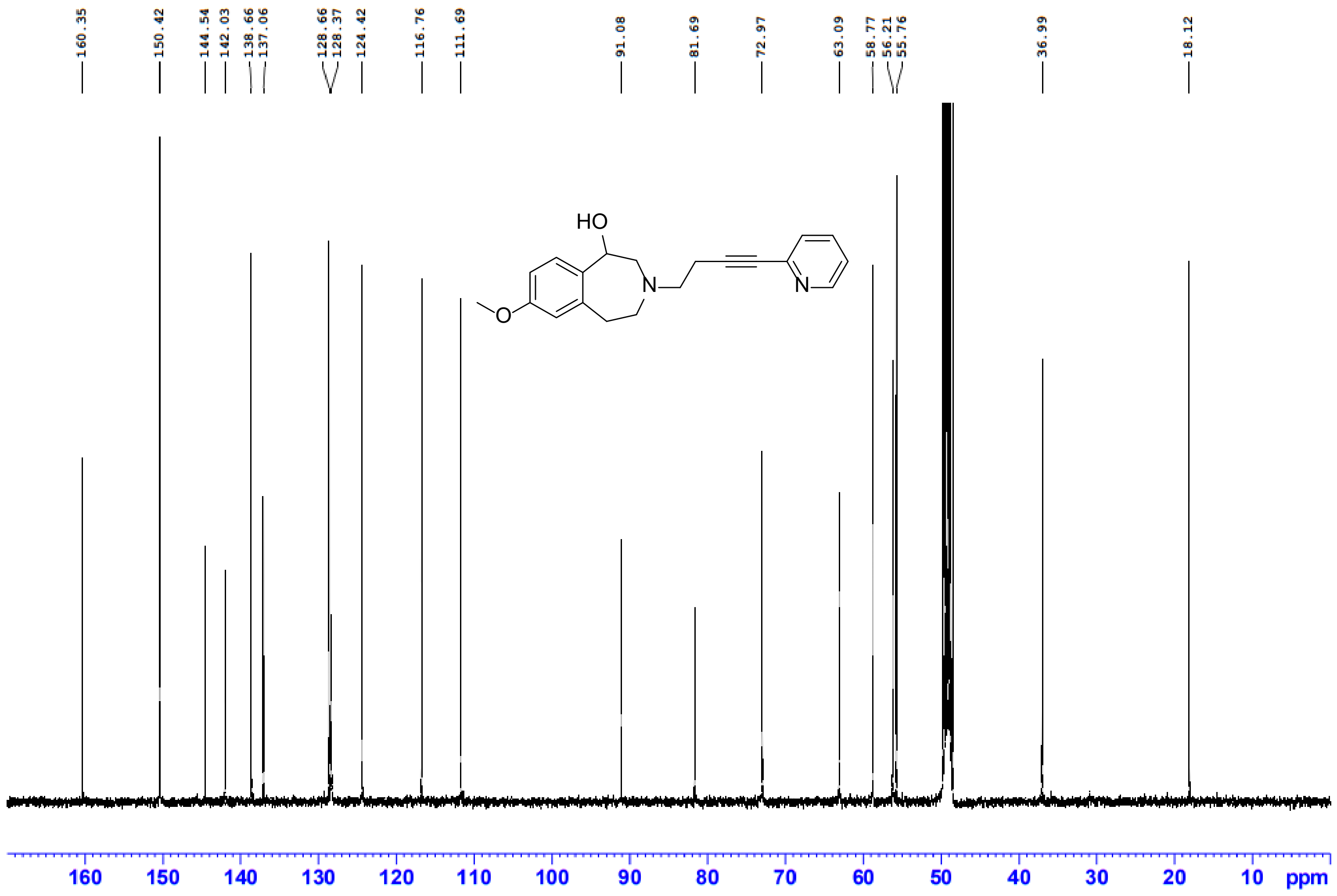


**Supplementary Figure 75.** $^{13}$C NMR Spectrum of **L11** (101 MHz, $CD_3OD$)

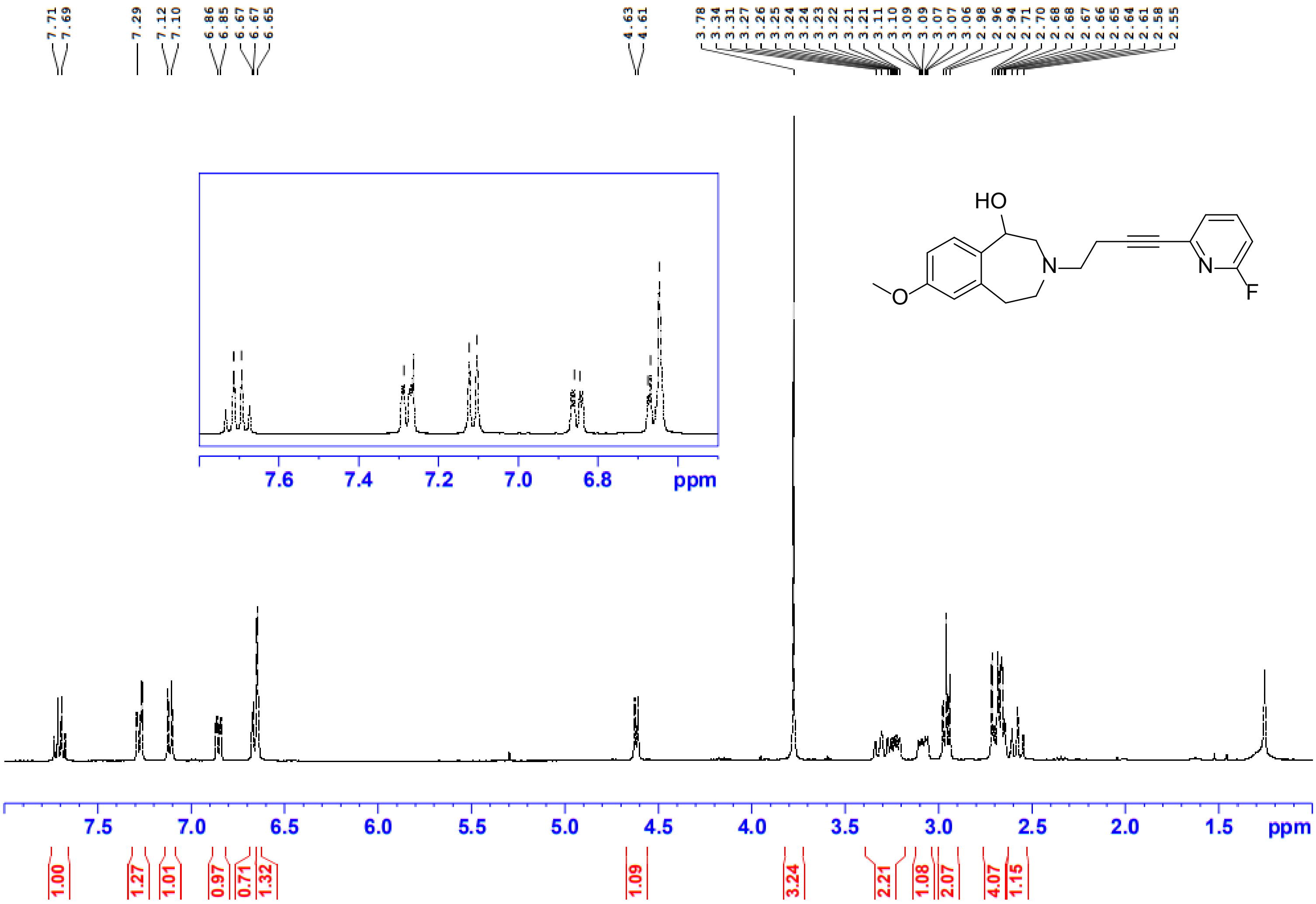

**Supplementary Figure 76.** $^{1}$H NMR Spectrum of **L12** (400 MHz, $CDCl_3$)

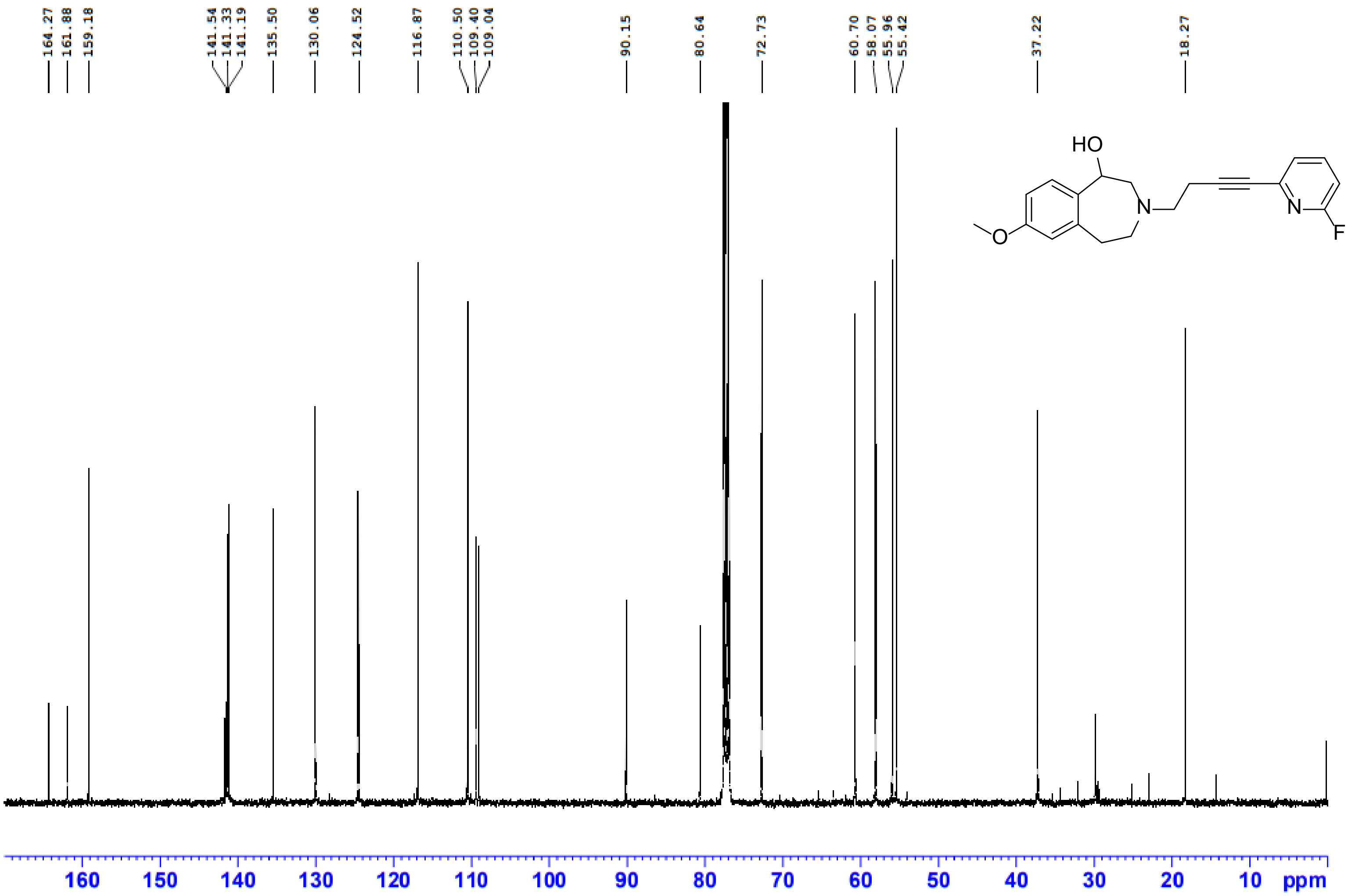


**Supplementary Figure 77.** $^{13}$C NMR Spectrum of **L12** (101 MHz, $CDCl_3$)

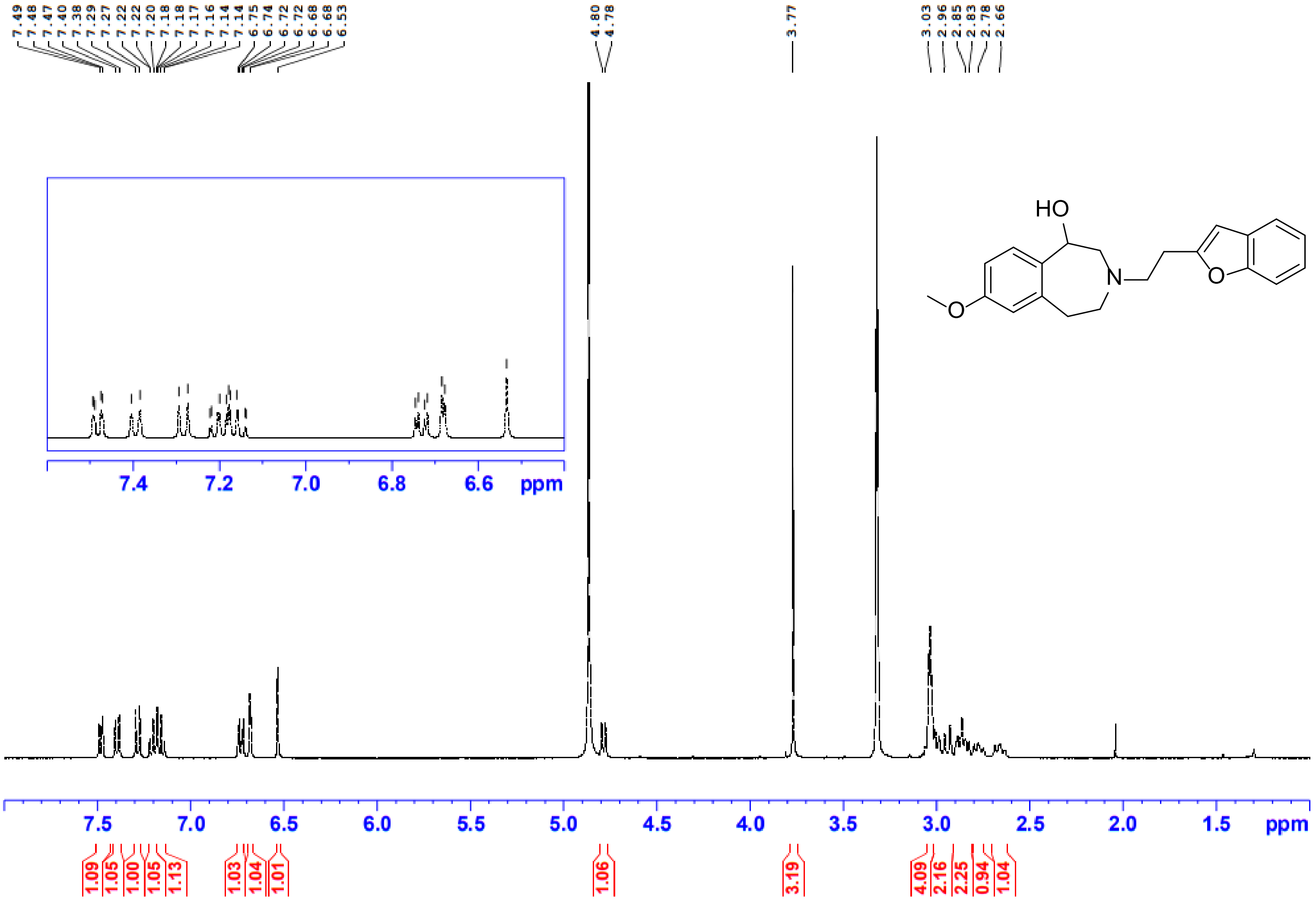


**Supplementary Figure 78.** $^{1}$H NMR Spectrum of **L13** (400 MHz, $CD_3OD$)

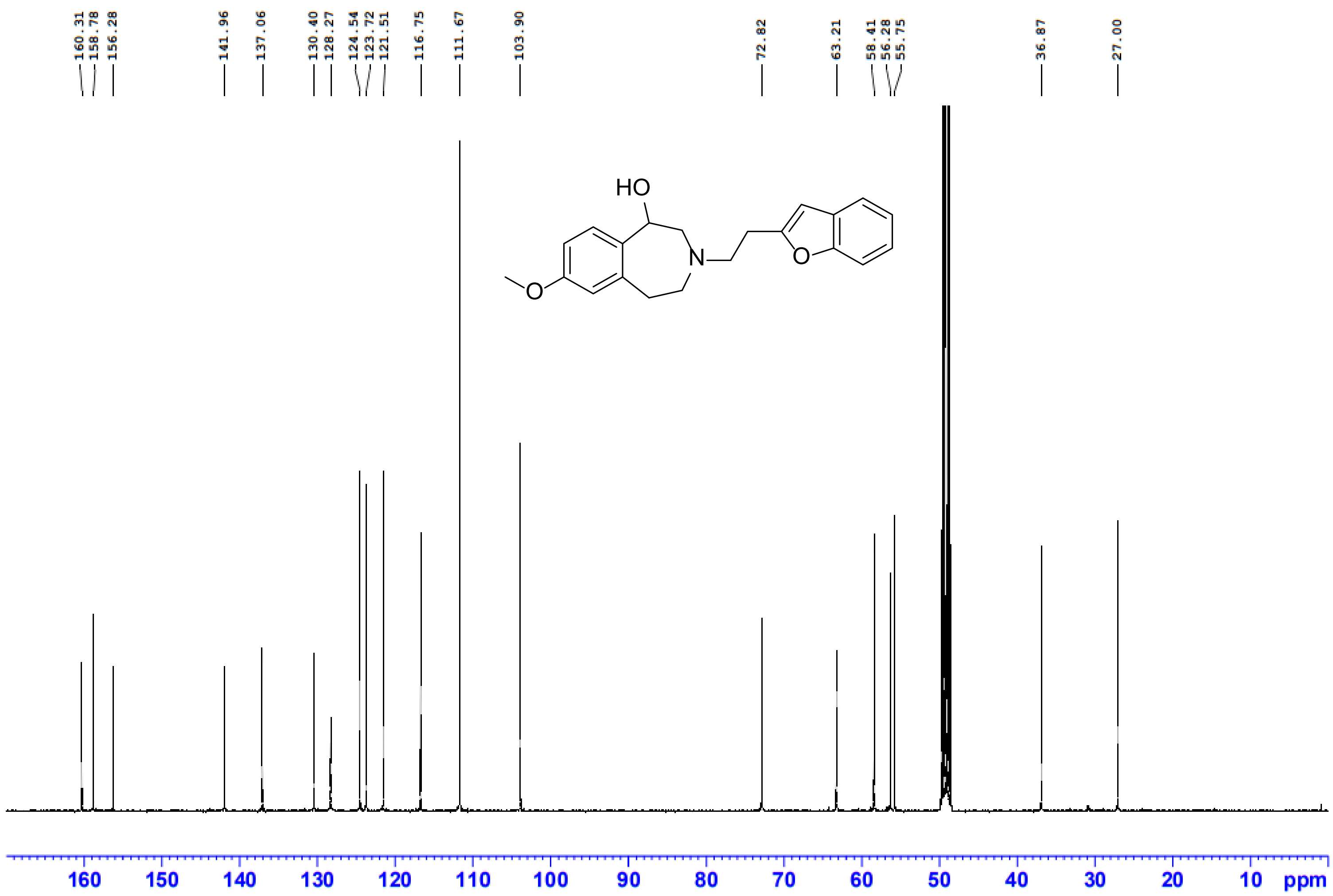


**Supplementary Figure79.** $^{13}C$ NMR Spectrum of **L13** (101 MHz, $CD_3OD$)

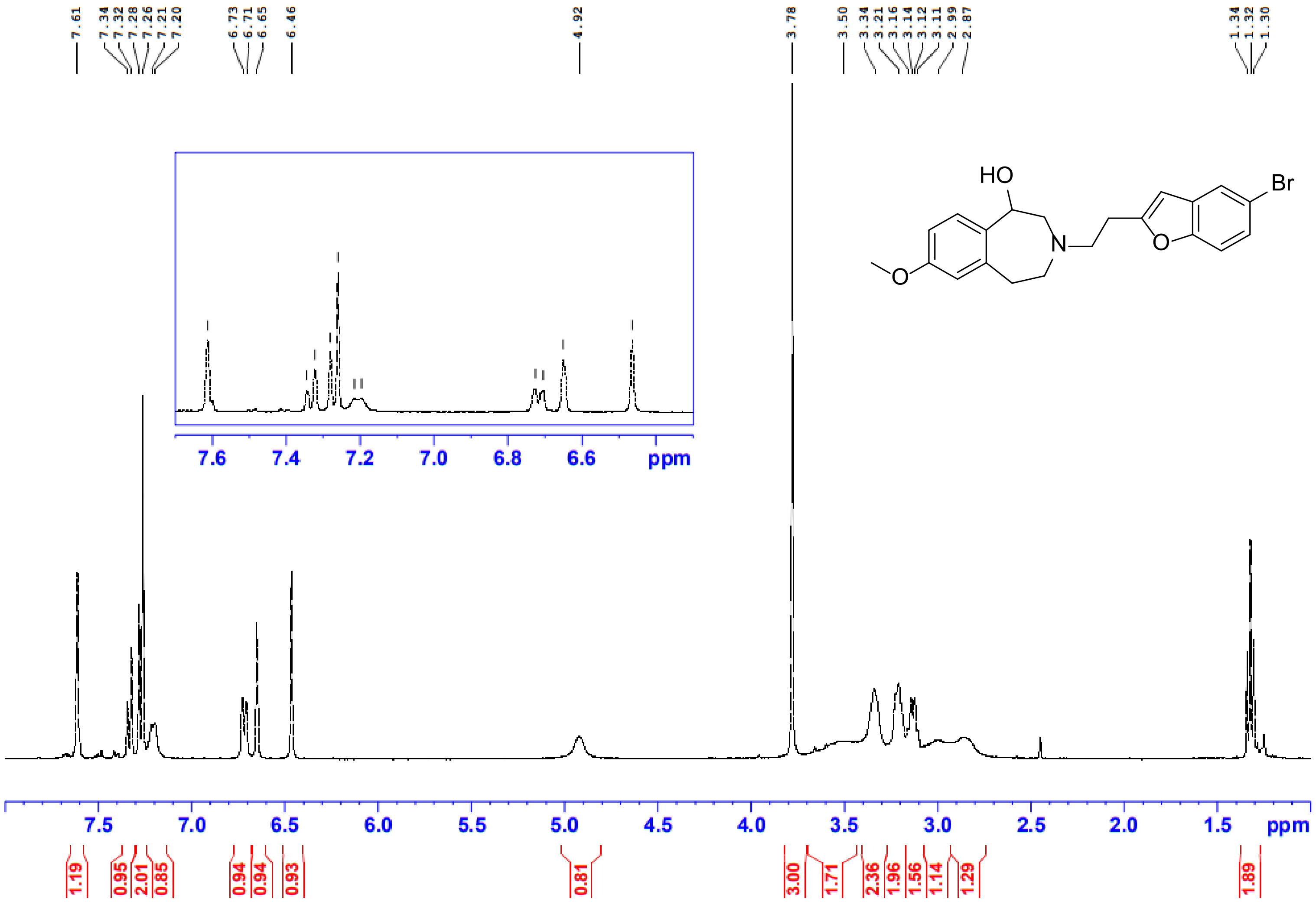


**Supplementary Figure 80.** $^{1}$H NMR Spectrum of **L14** (400 MHz, $CDCl_3$)

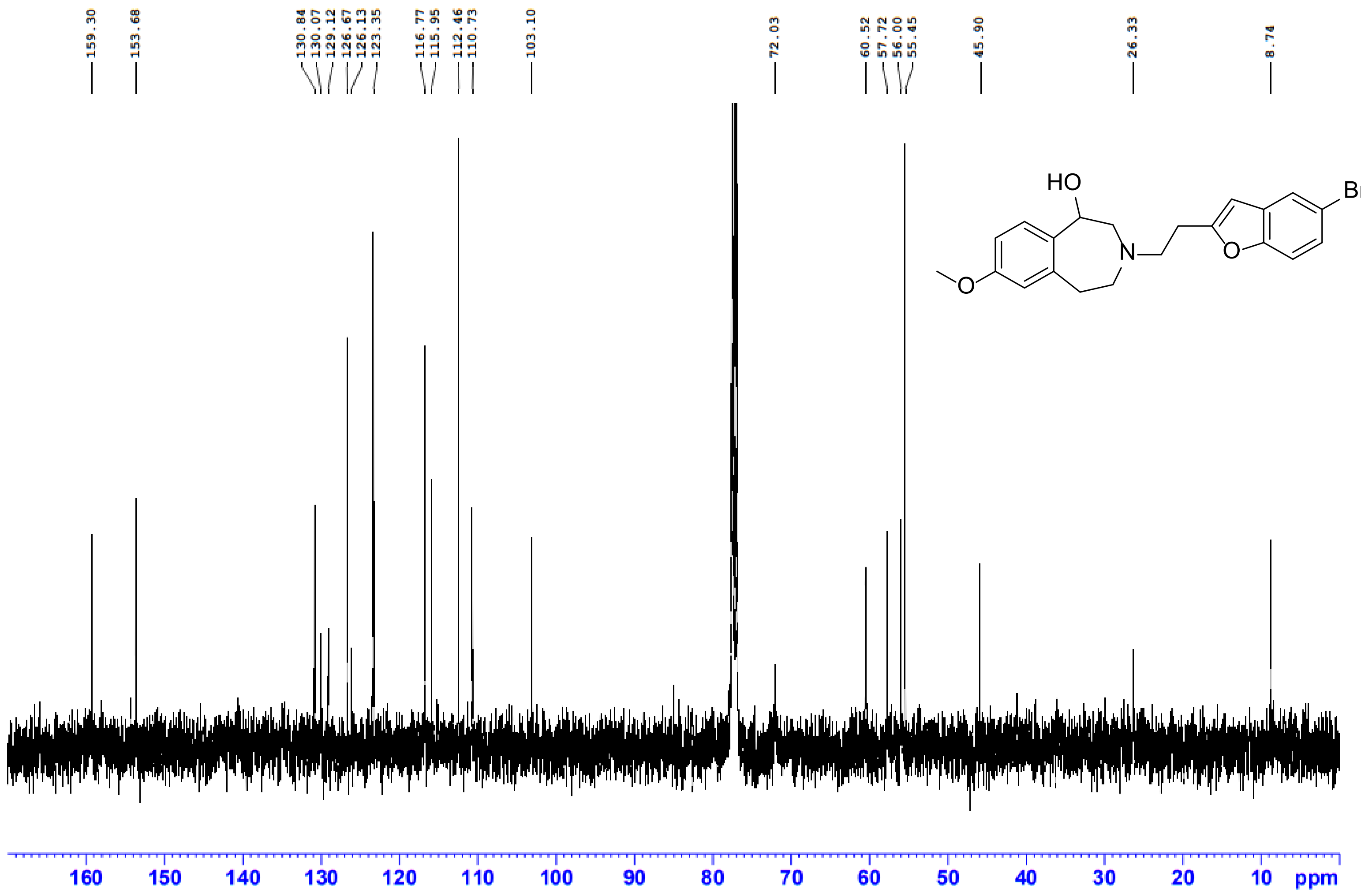


**Supplementary Figure 81.** $^{13}$C NMR Spectrum of **L14** (101 MHz, $CDCl_3$)

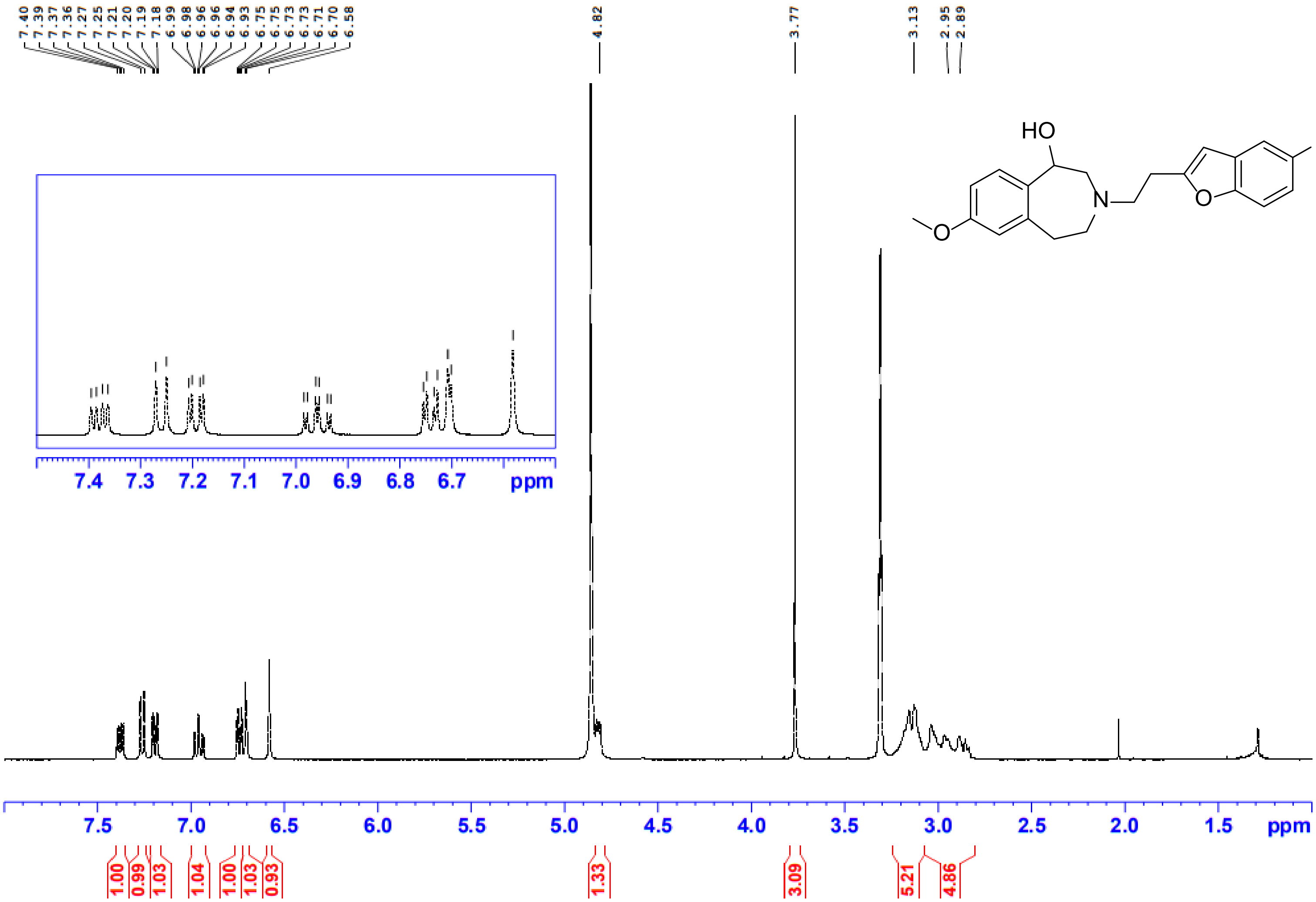

**Supplementary Figure 82.** $^{1}$H NMR Spectrum of **L15** (400 MHz, $CD_3OD$)

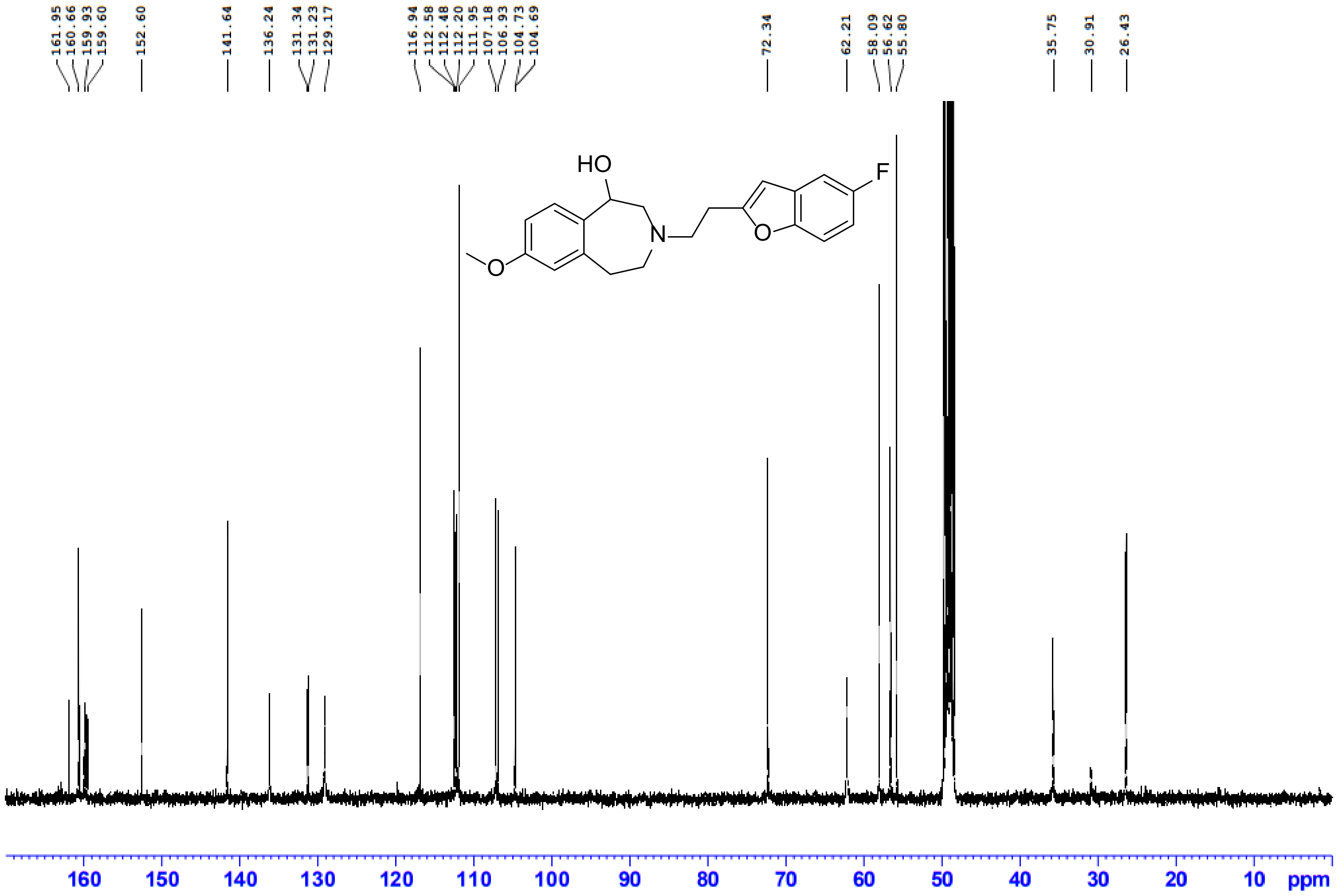


**Supplementary Figure 83.** $^{13}C$ NMR Spectrum of **L15** (101 MHz, $CD_3OD$)

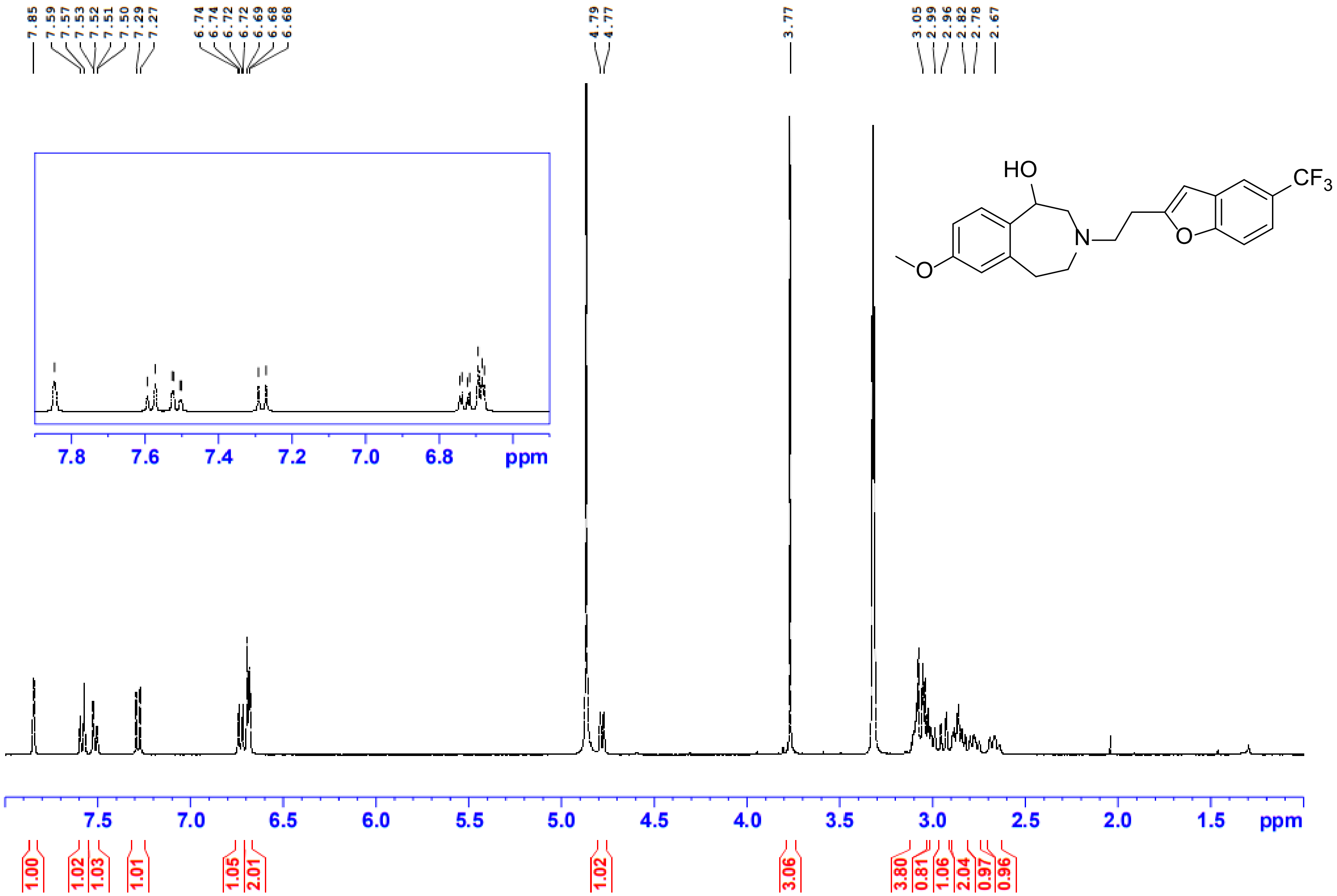

**Supplementary Figure 84.** $^{1}$H NMR Spectrum of **L16** (400 MHz, $CD_3OD$)

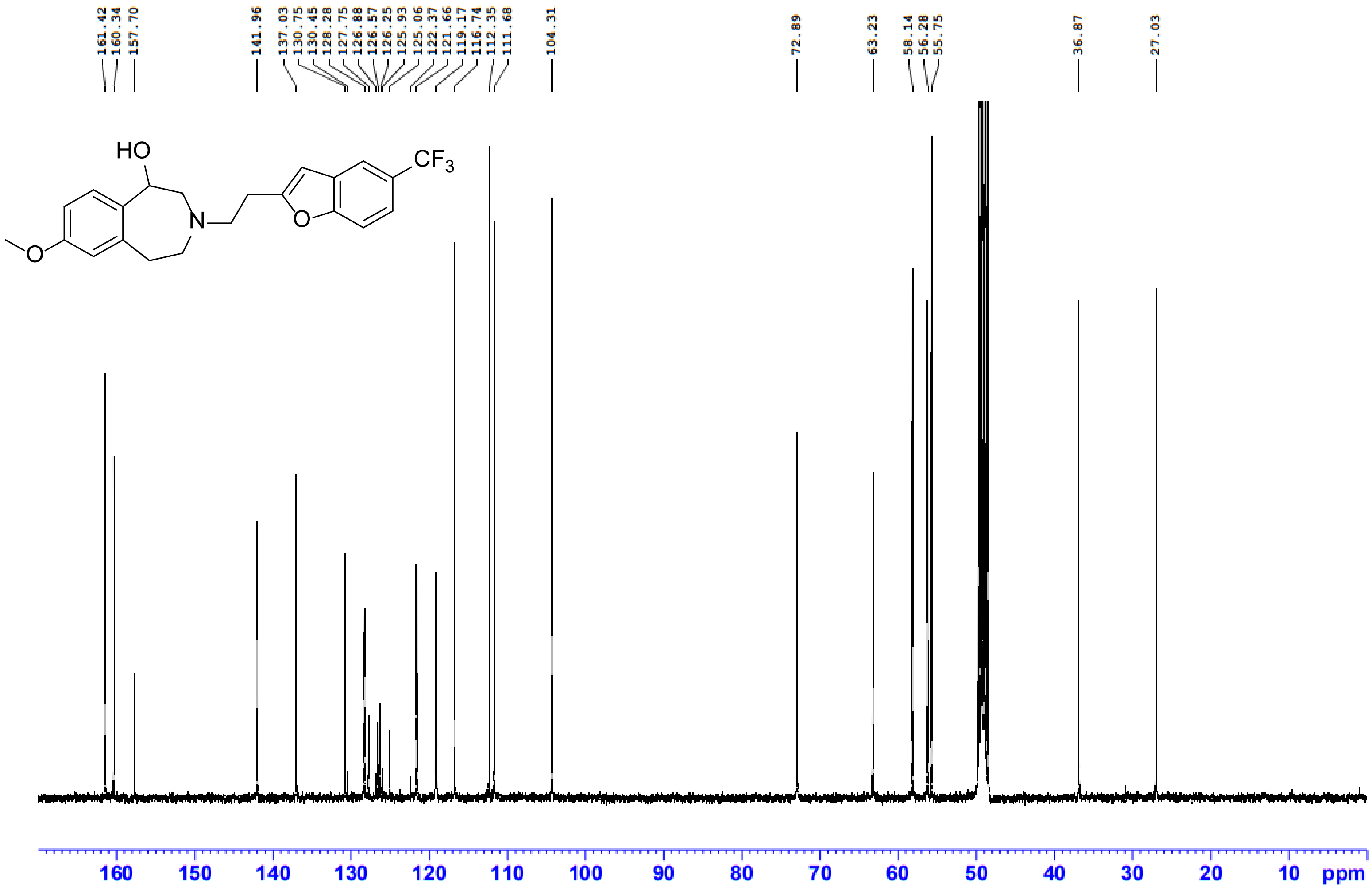


**Supplementary Figure 85.** $^{13}$C NMR Spectrum of **L16** (101 MHz, $CD_3OD$)

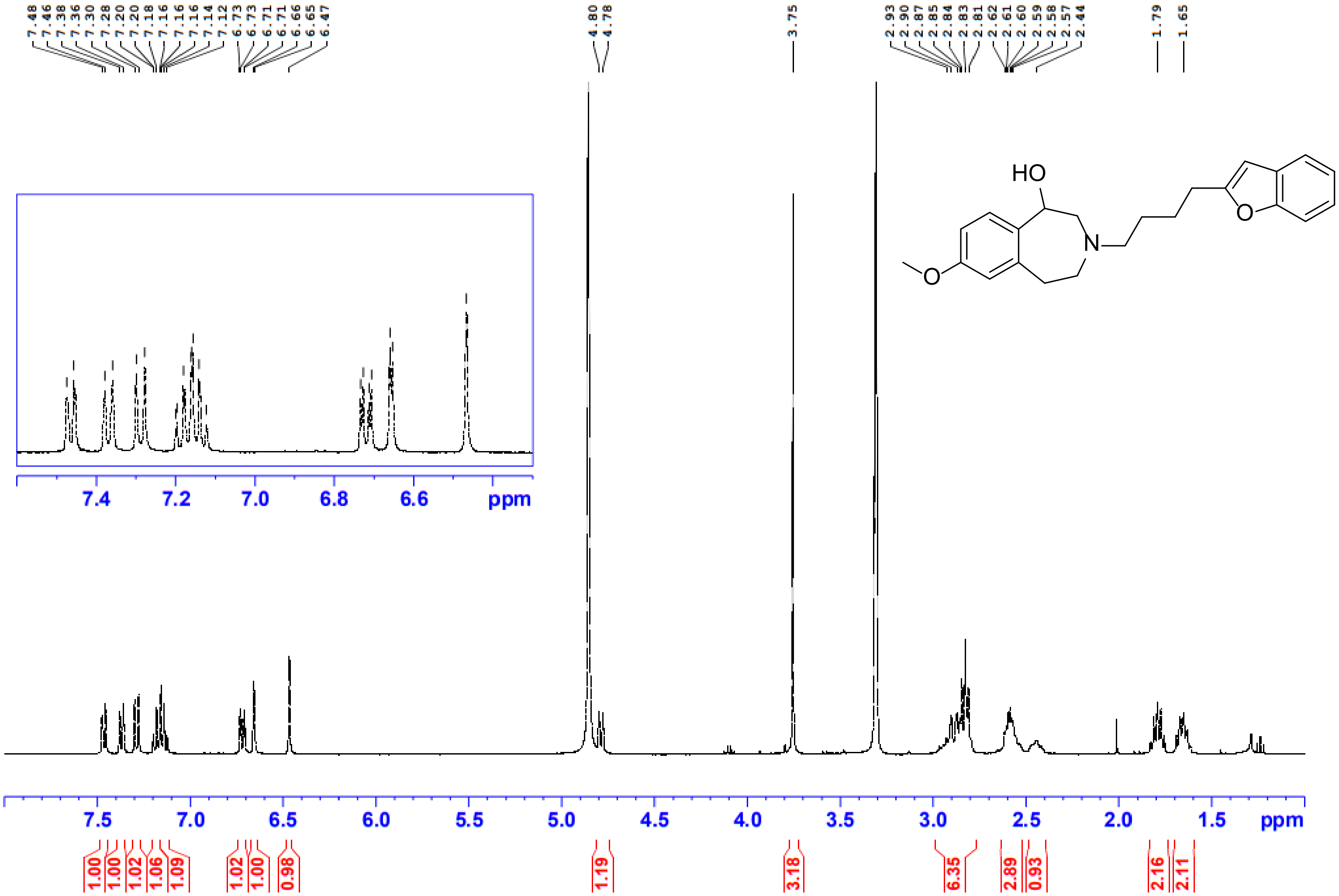


**Supplementary Figure 86.** $^{1}$H NMR Spectrum of **L17** (400 MHz, $CD_3OD$)

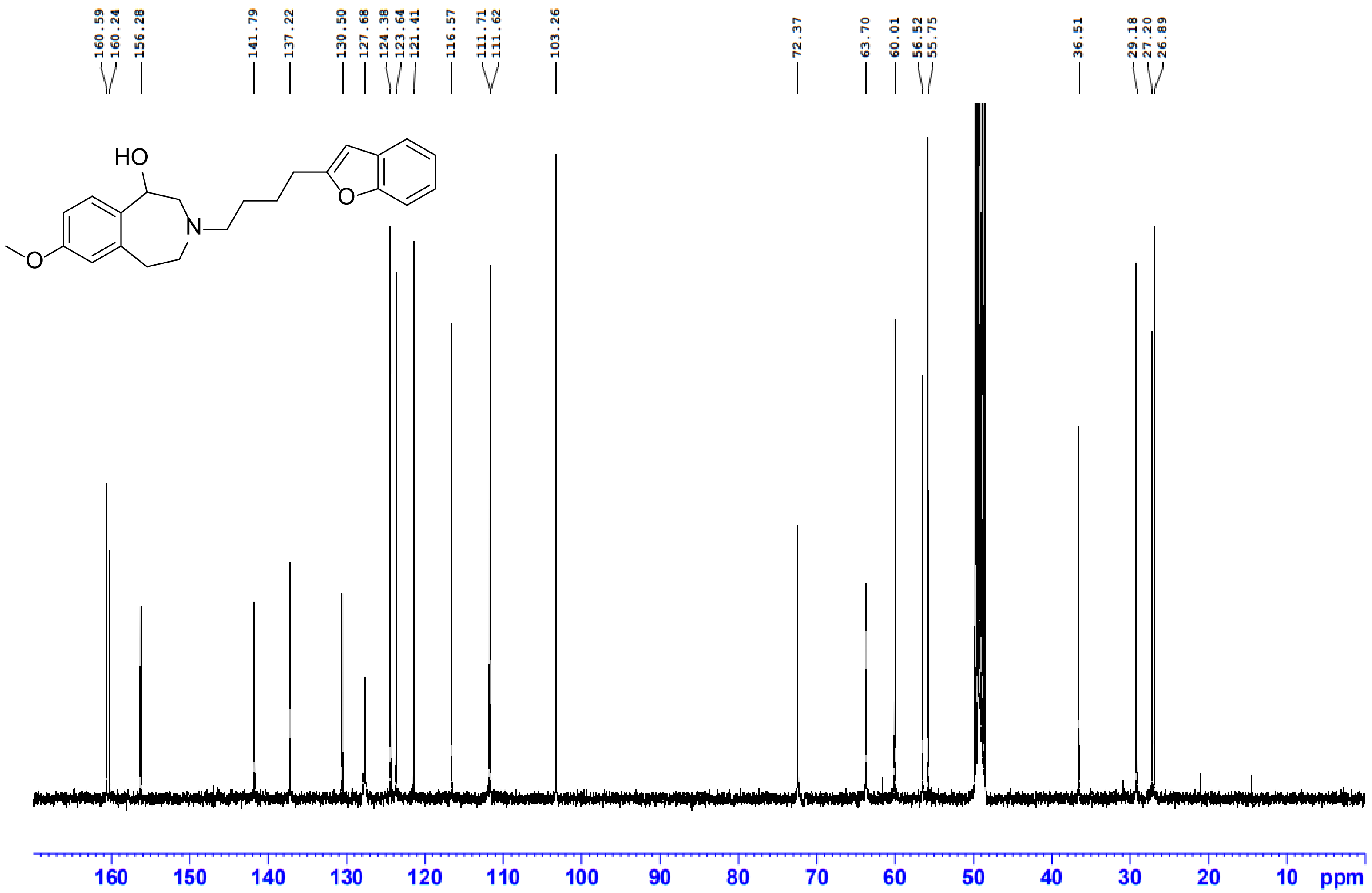


**Supplementary Figure 87.** $^{13}$C NMR Spectrum of **L17** (101 MHz, $CD_3OD$)

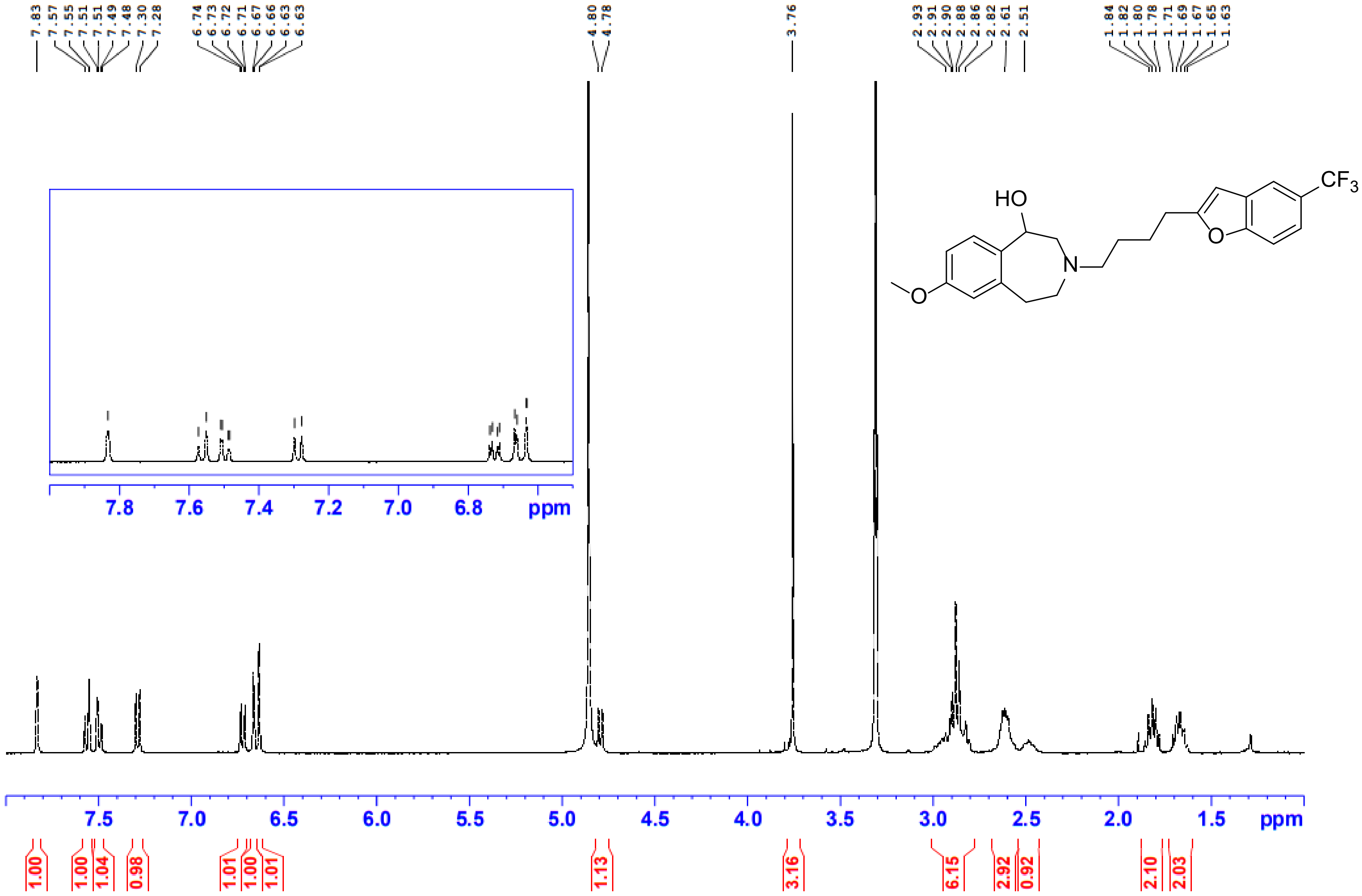

**Supplementary Figure 88.** $^{1}$H NMR Spectrum of **L18** (400 MHz, $CD_3OD$)

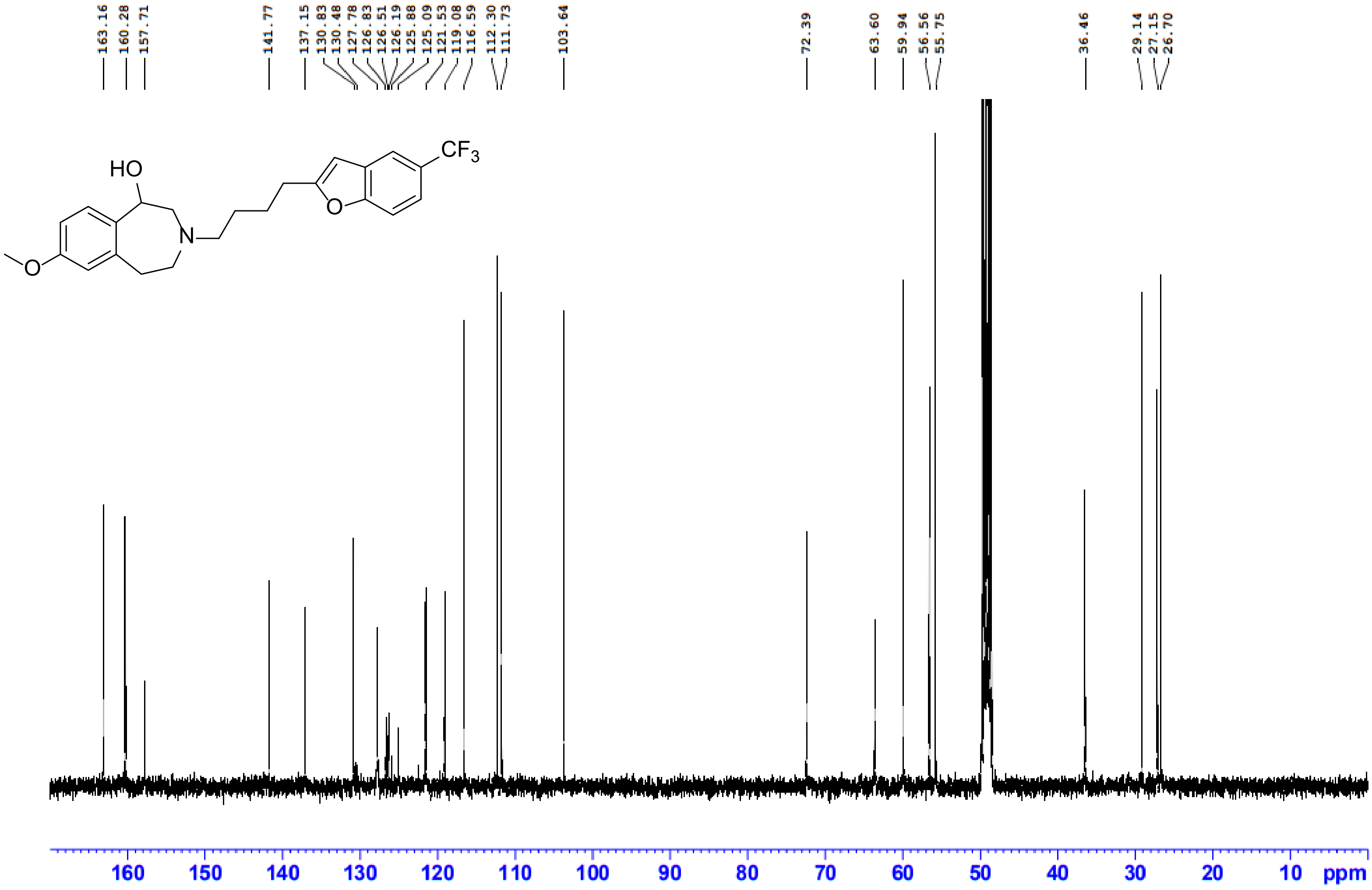


**Supplementary Figure 89.** $^{13}C$ NMR Spectrum of **L18** (101 MHz, $CD_3OD$)

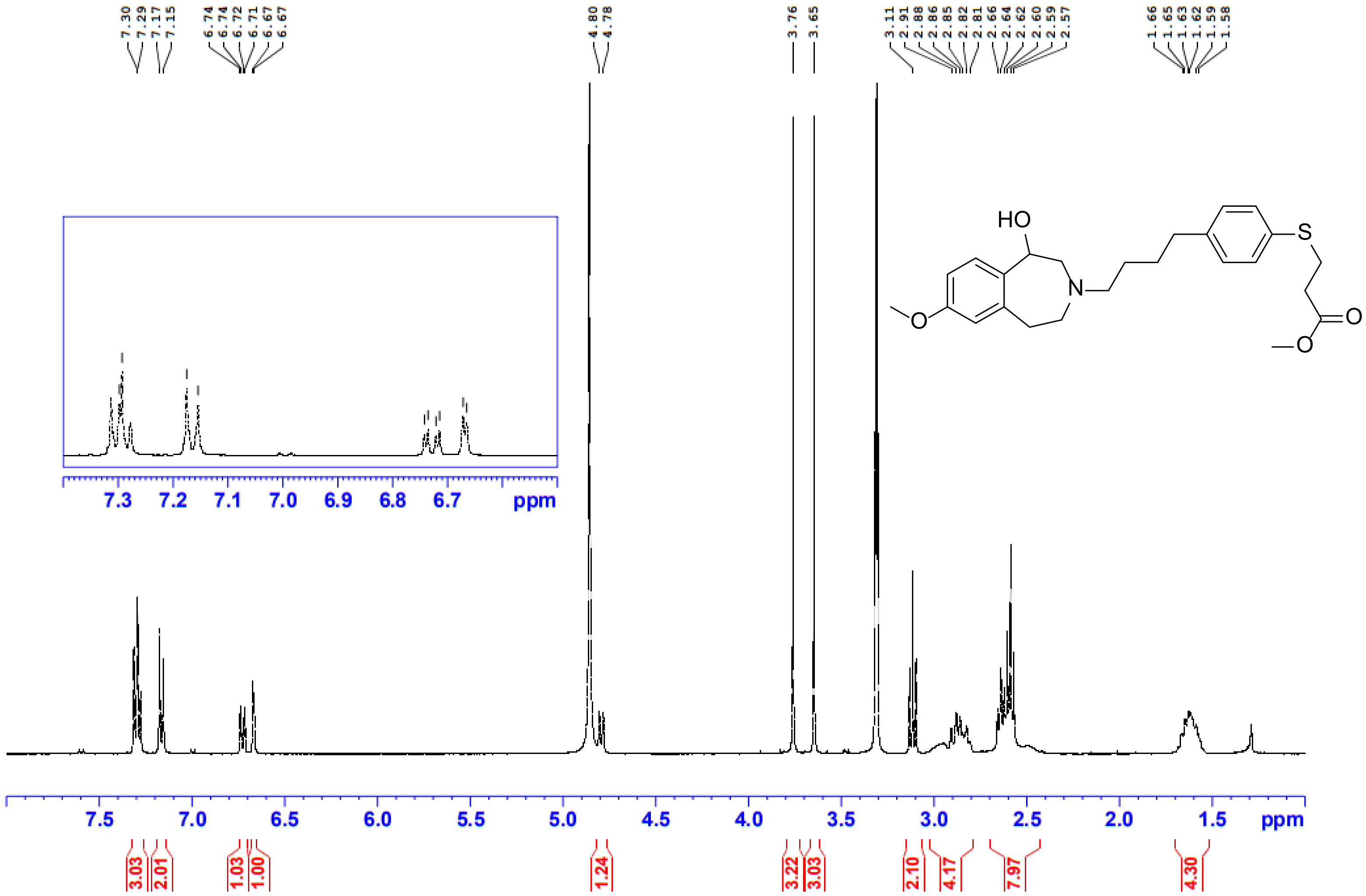


**Supplementary Figure 90.** $^1$H NMR Spectrum of **L19** (400 MHz, $CD_3OD$)

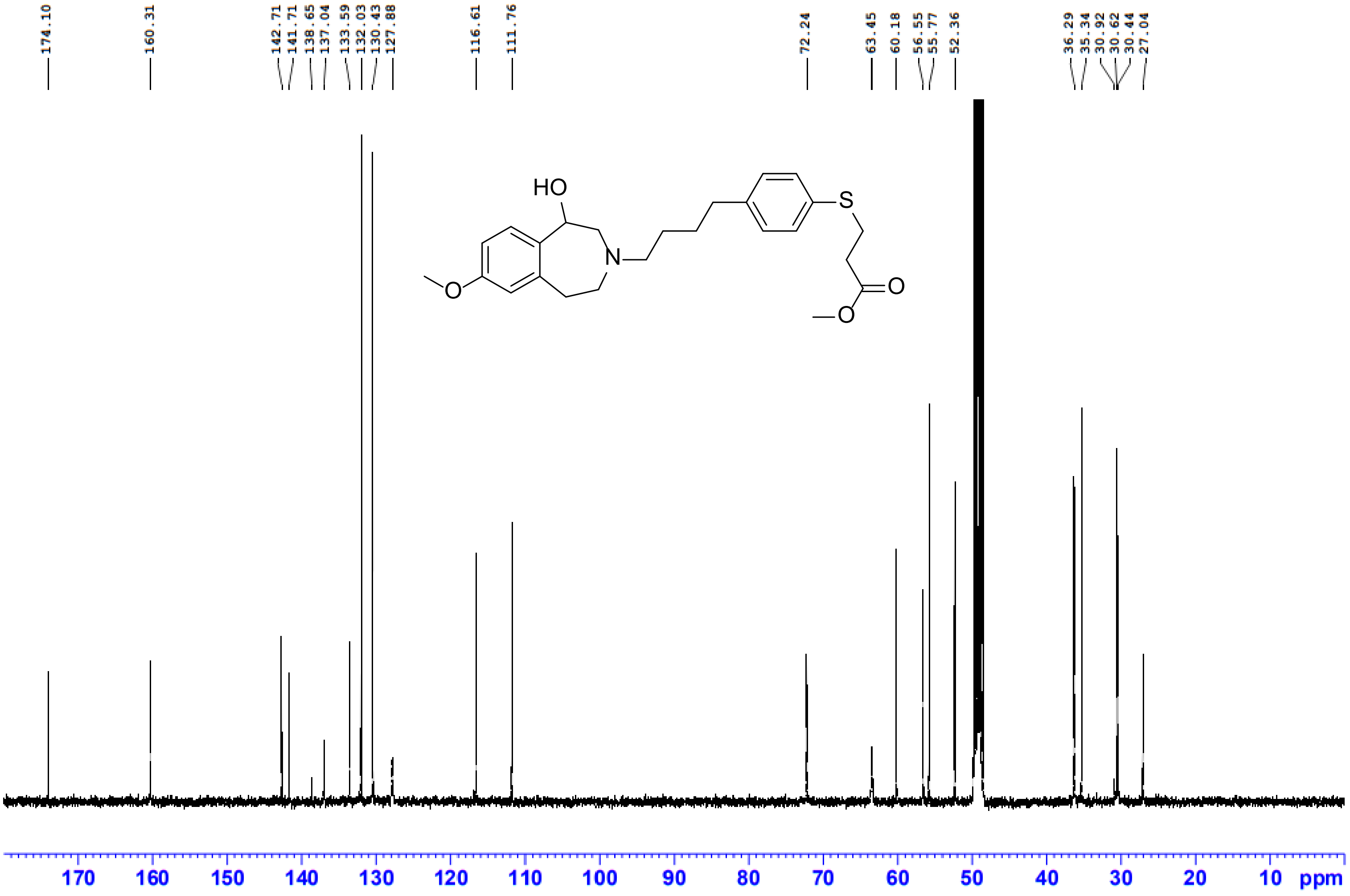


**Supplementary Figure 91.** $^{13}$C NMR Spectrum of **L19** (101 MHz, $CD_3OD$)

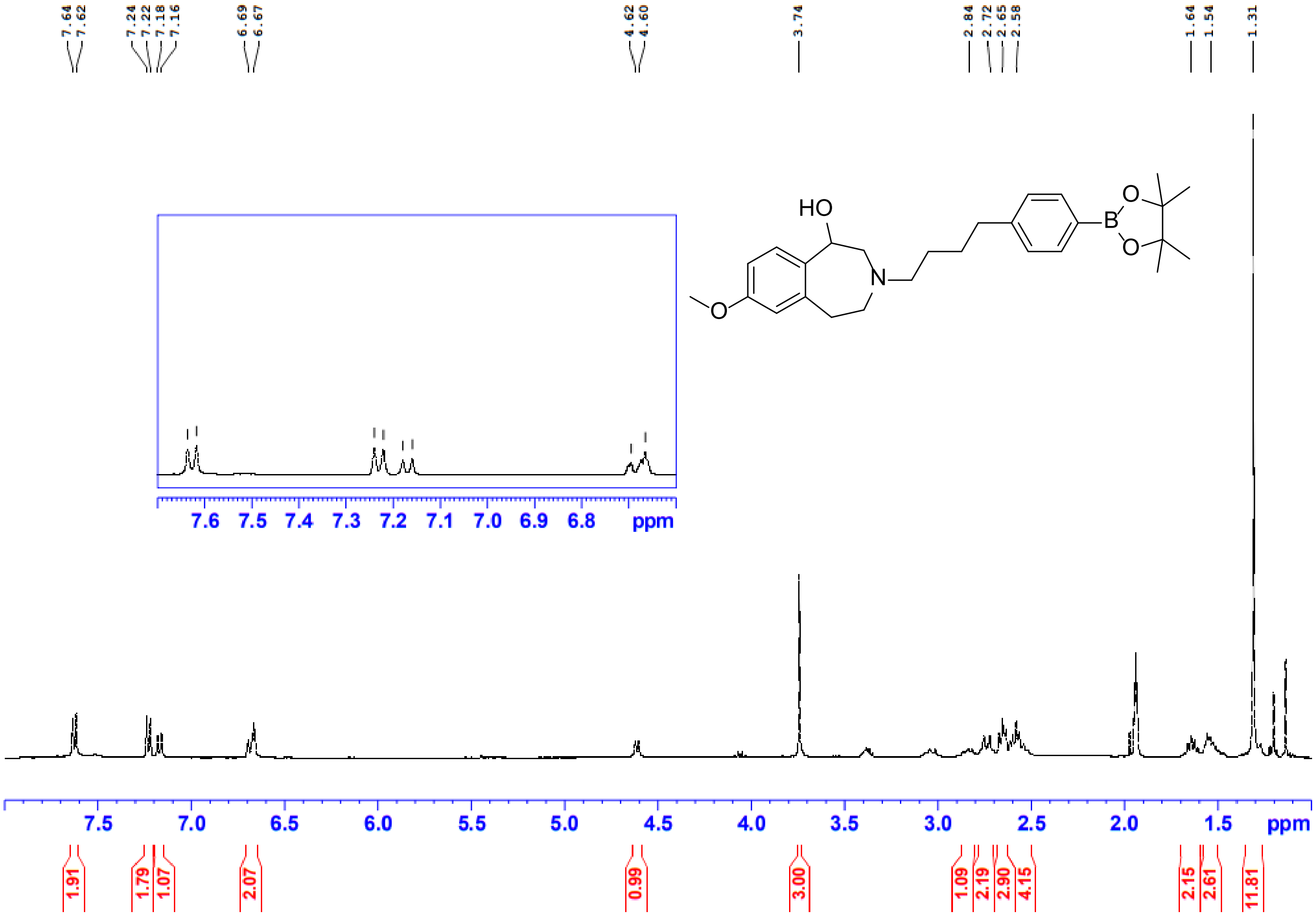


**Supplementary Figure 92.** $^{1}$H NMR Spectrum of **L20** (400 MHz, $CD_3CN$)

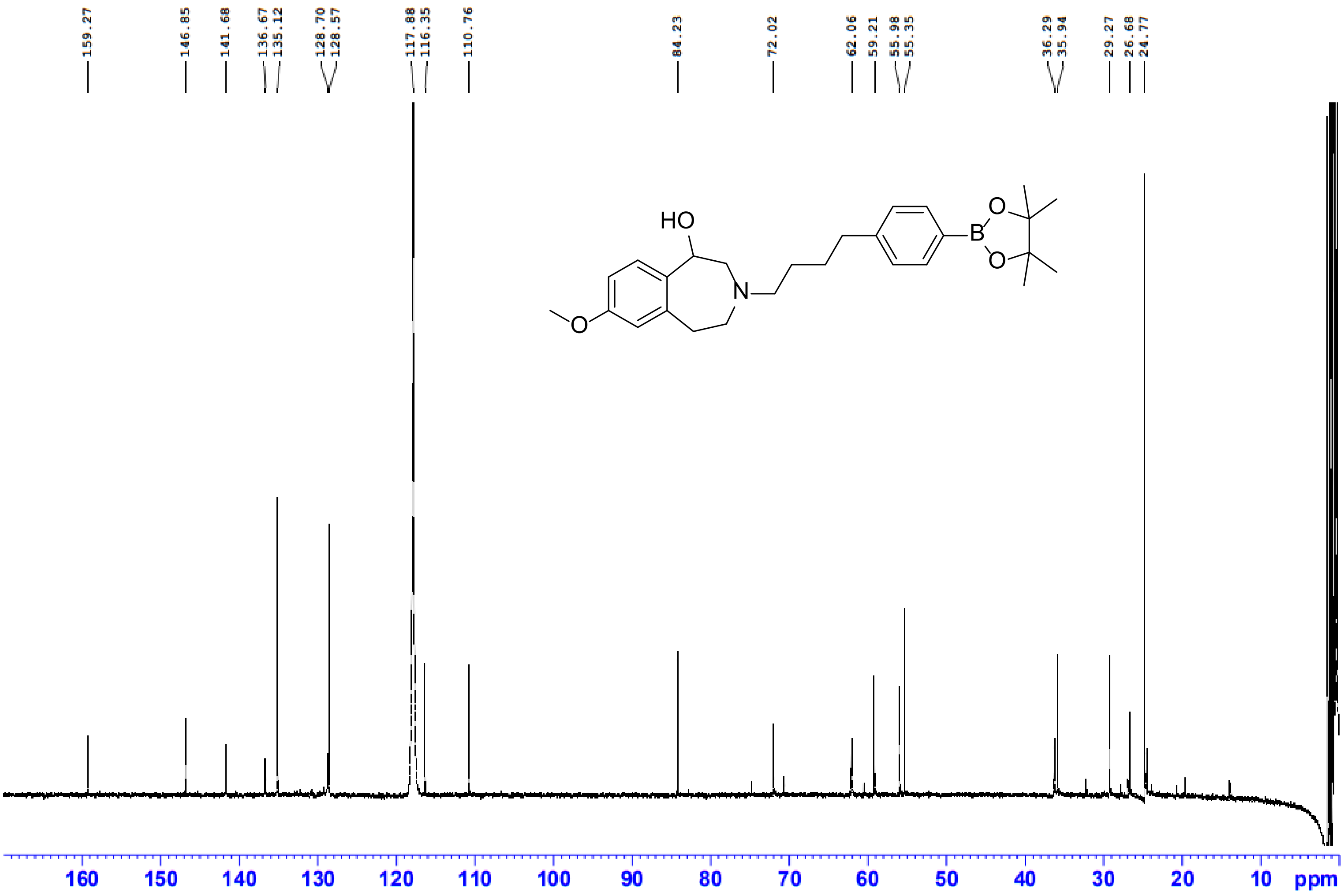


**Supplementary Figure 93.** ${}^{13}$C NMR Spectrum of **L20** (101 MHz, $CD_3CN$)

## 6. Chiral Chromatograms for L2, L3, L6, L19, and L20, and Enantiomers

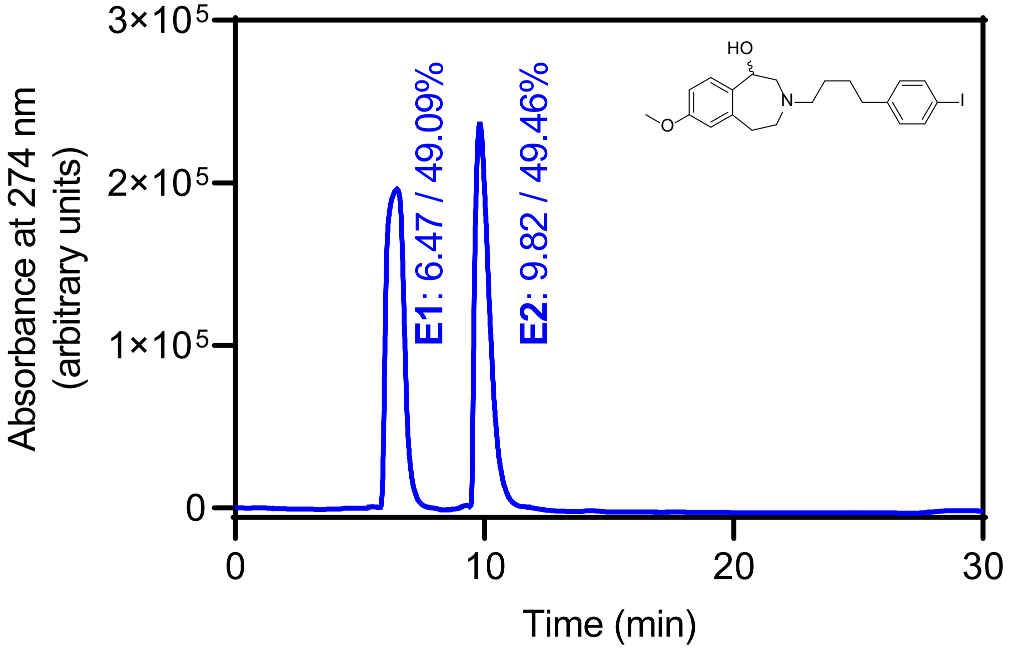


**Supplementary Figure 94.** Chiral HPLC chromatogram for racemic **L2** (general method F).

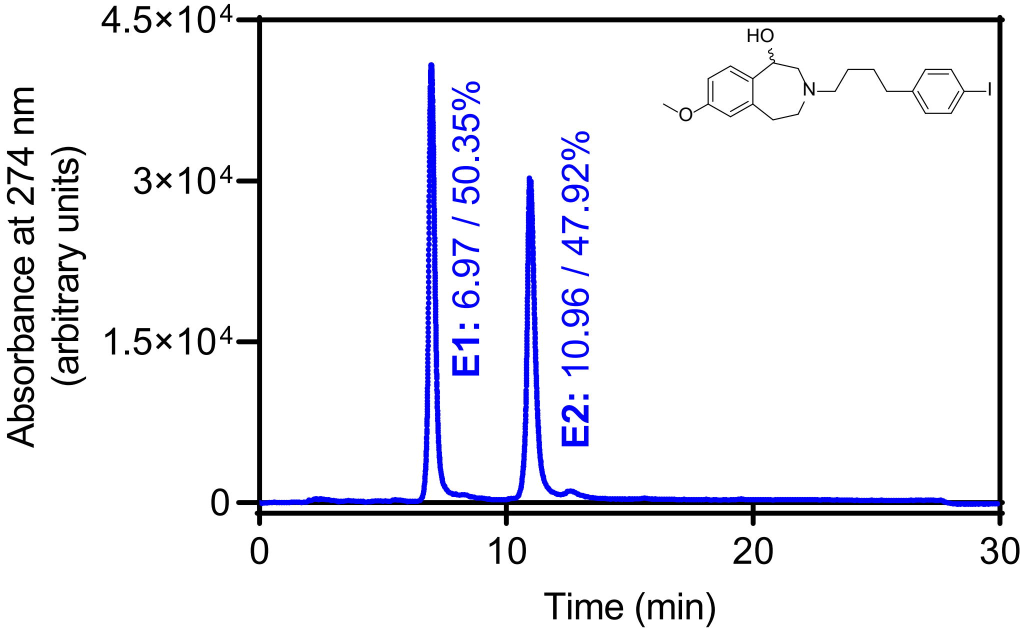


**Supplementary Figure 95.** Chiral HPLC chromatogram for racemic **L2** (general method E).

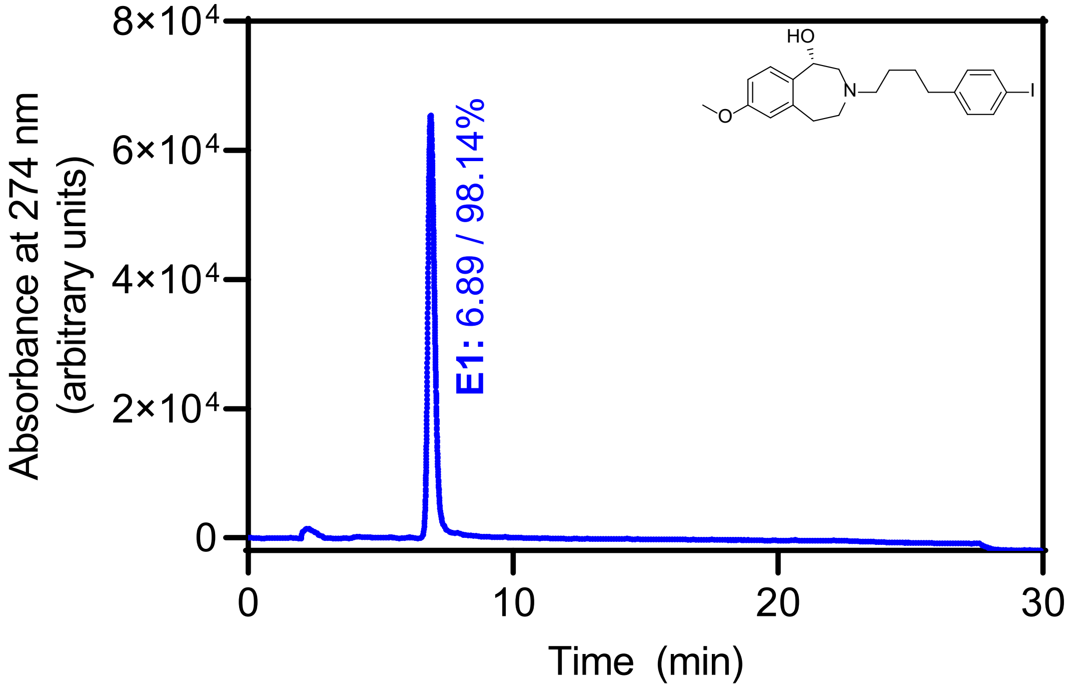


**Supplementary Figure 96.** Chiral HPLC chromatogram for ***(S)*-L2** (general method E).

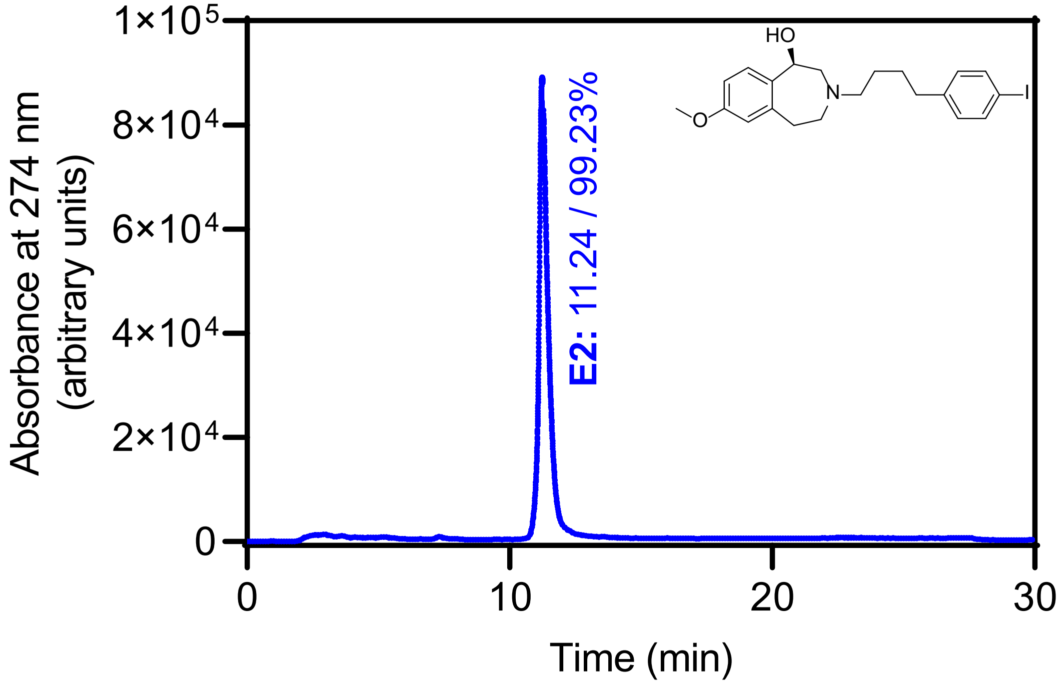


**Supplementary Figure 97.** Chiral HPLC chromatogram for ***(R)*-L2** (general method E).

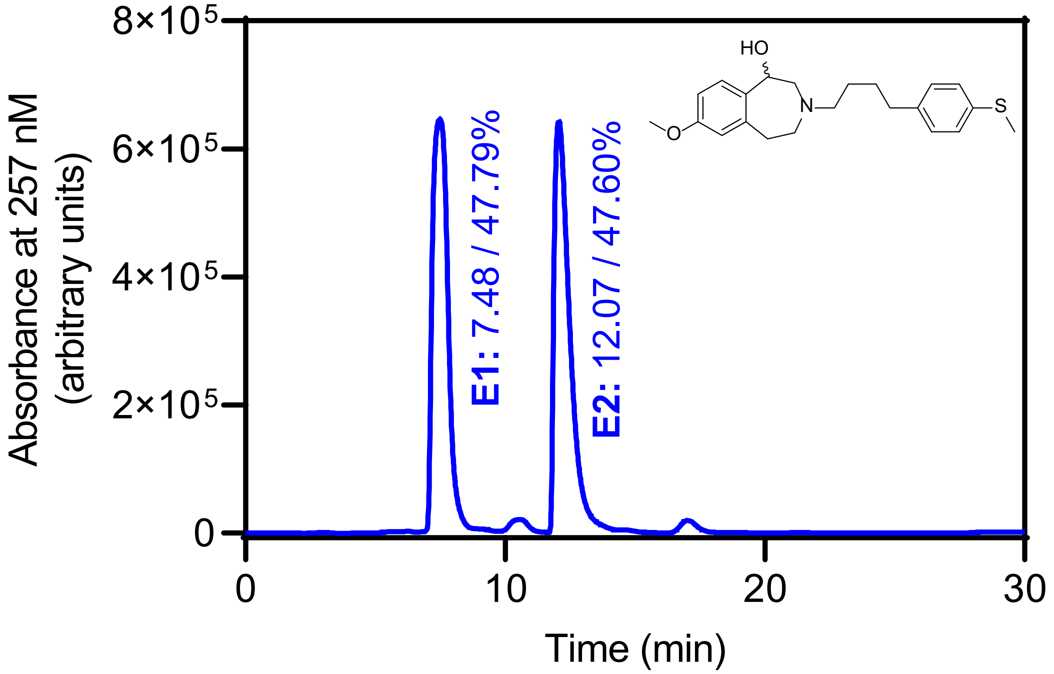


**Supplementary Figure 98.** Preparative chiral HPLC chromatogram for racemic **L3** (general method F).

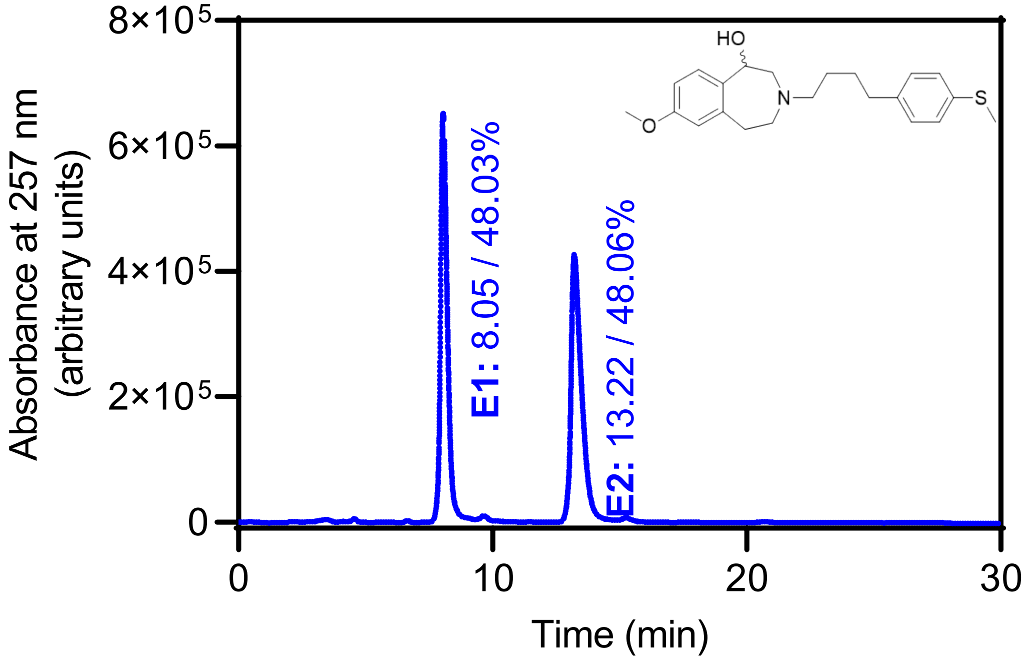


**Supplementary Figure 99.** Chiral HPLC chromatogram for racemic **L3** (general method E).

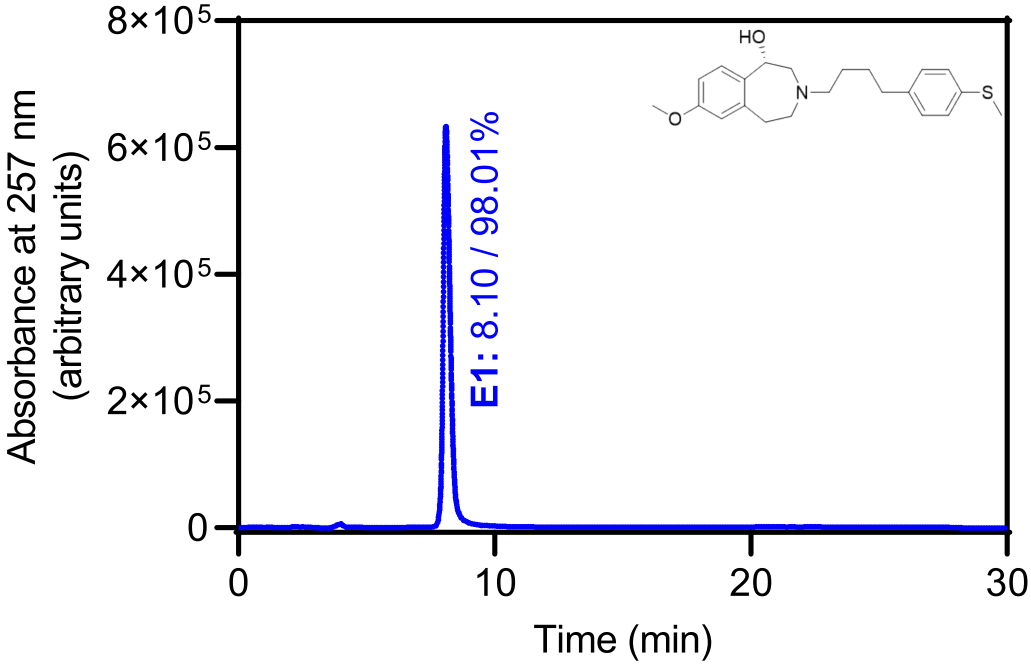


**Supplementary Figure 100.** Chiral HPLC chromatogram for **(*S*)-L3** (general method E).

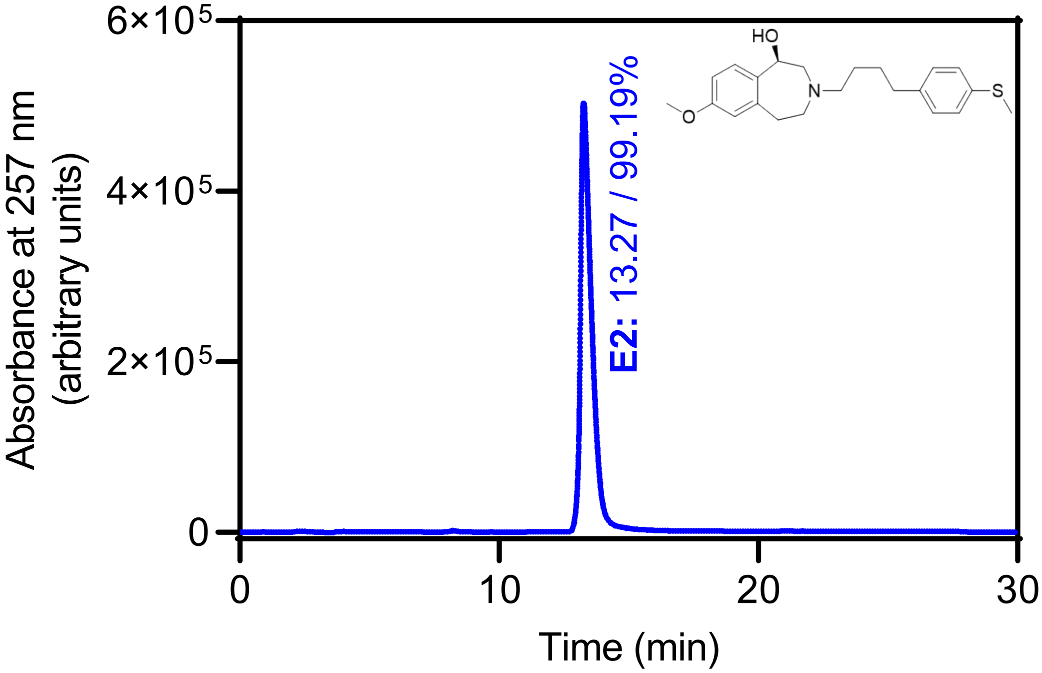


**Supplementary Figure 101.** Chiral HPLC chromatogram **(*R*)-L3** (general method E).

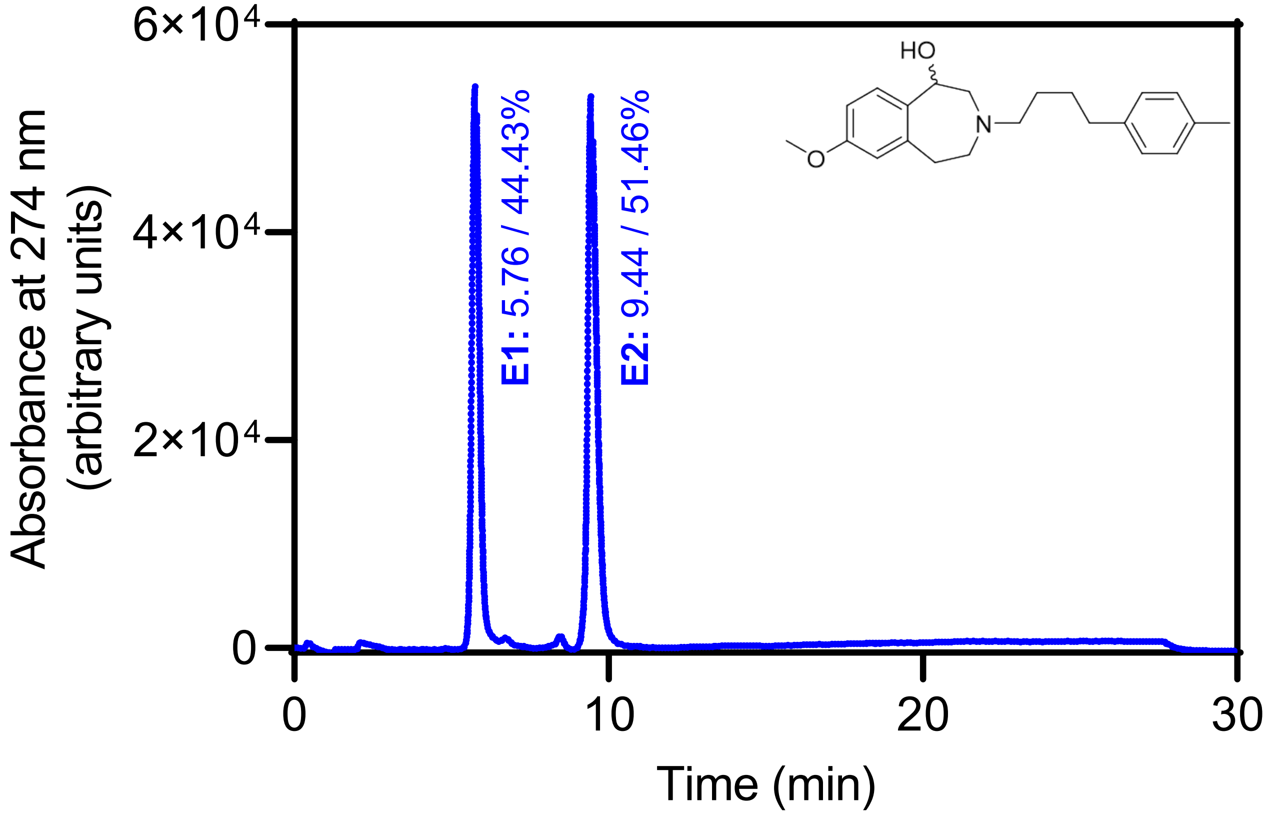


**Supplementary Figure 102.** Chiral HPLC chromatogram for **L6** (general method E).

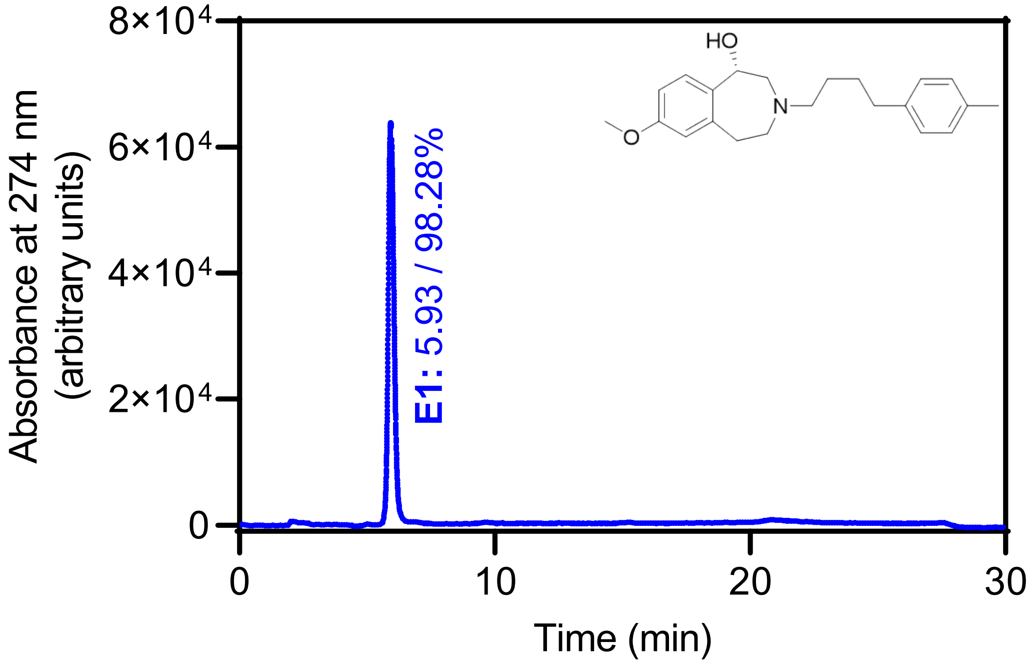


**Supplementary Figure 103.** Chiral HPLC chromatogram for (***S***)-**L6** (general method E).

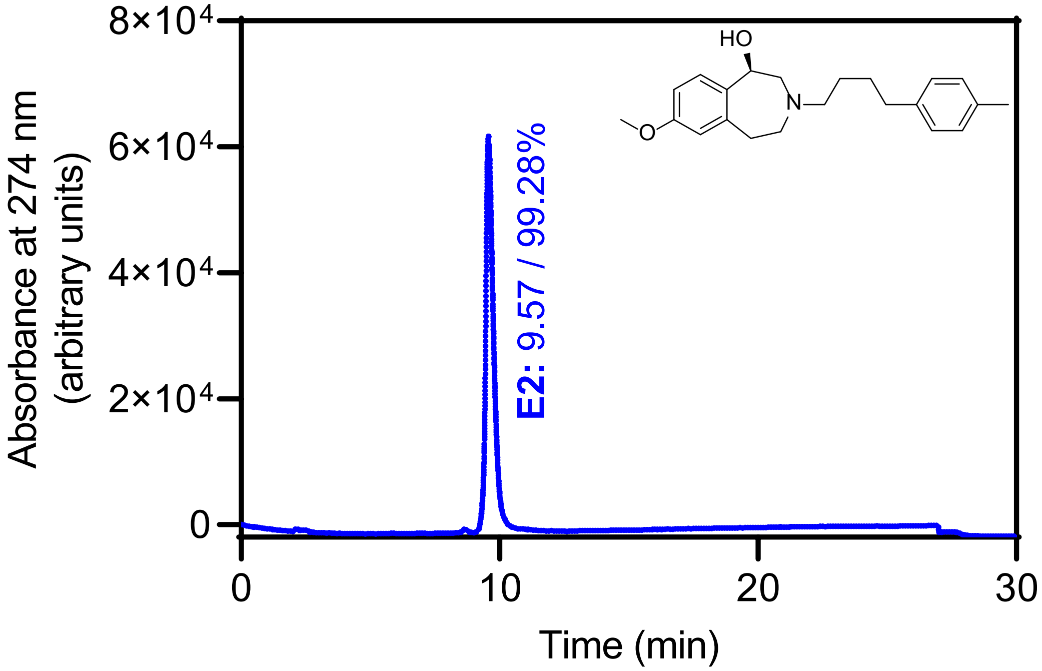


**Supplementary Figure 104.** Chiral HPLC chromatogram for **(*R*)-L6** (general method E).

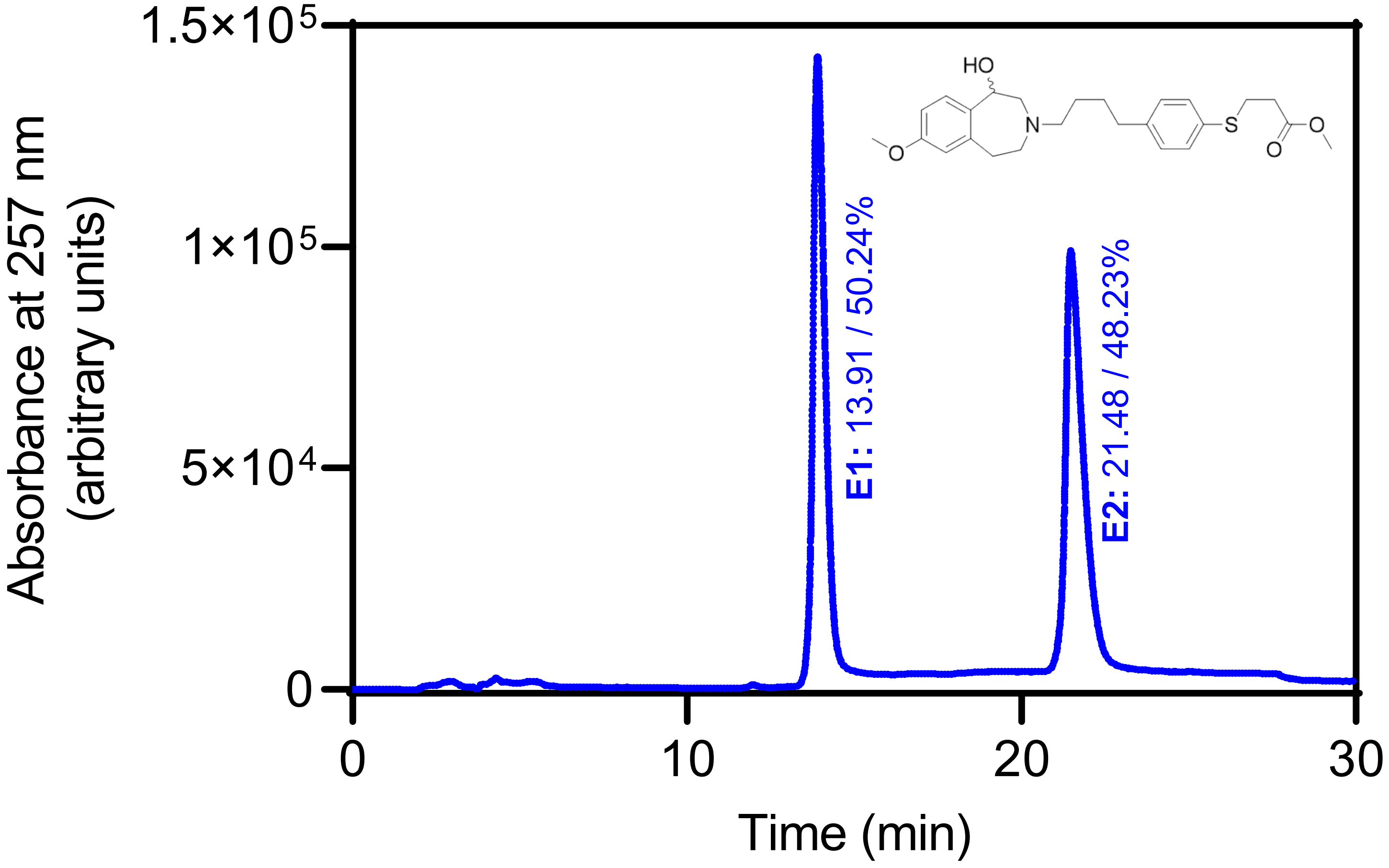


**Supplementary Figure 105.** Chiral HPLC chromatogram for **L19** (general method E).

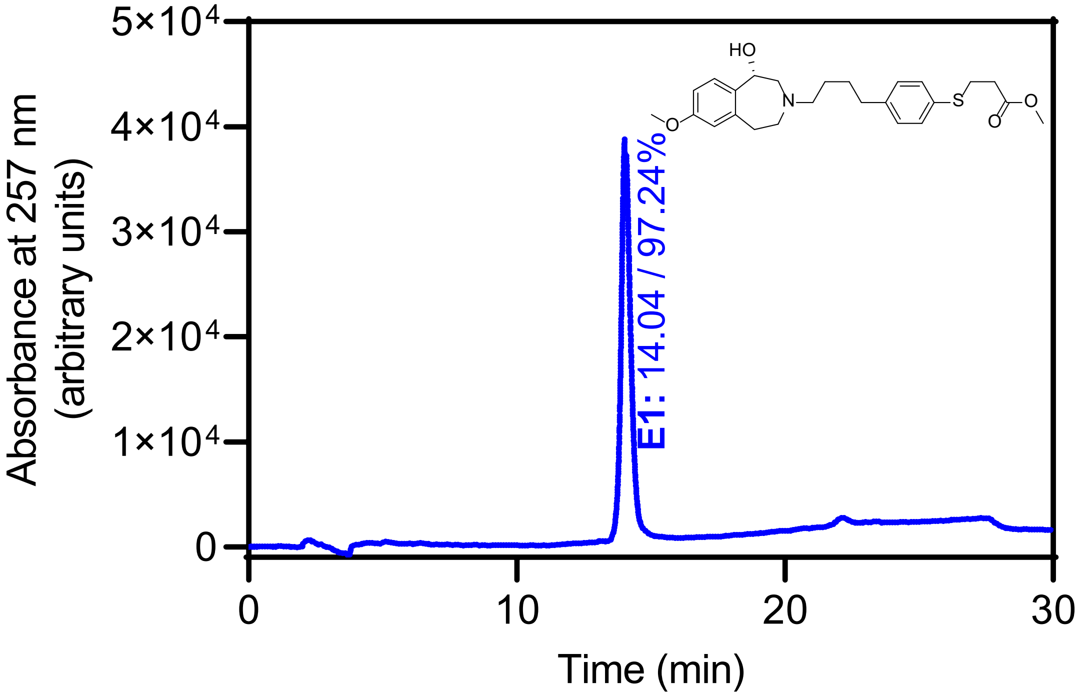


**Supplementary Figure 106.** Chiral HPLC chromatogram for **(*S*)-L19** (general method E)

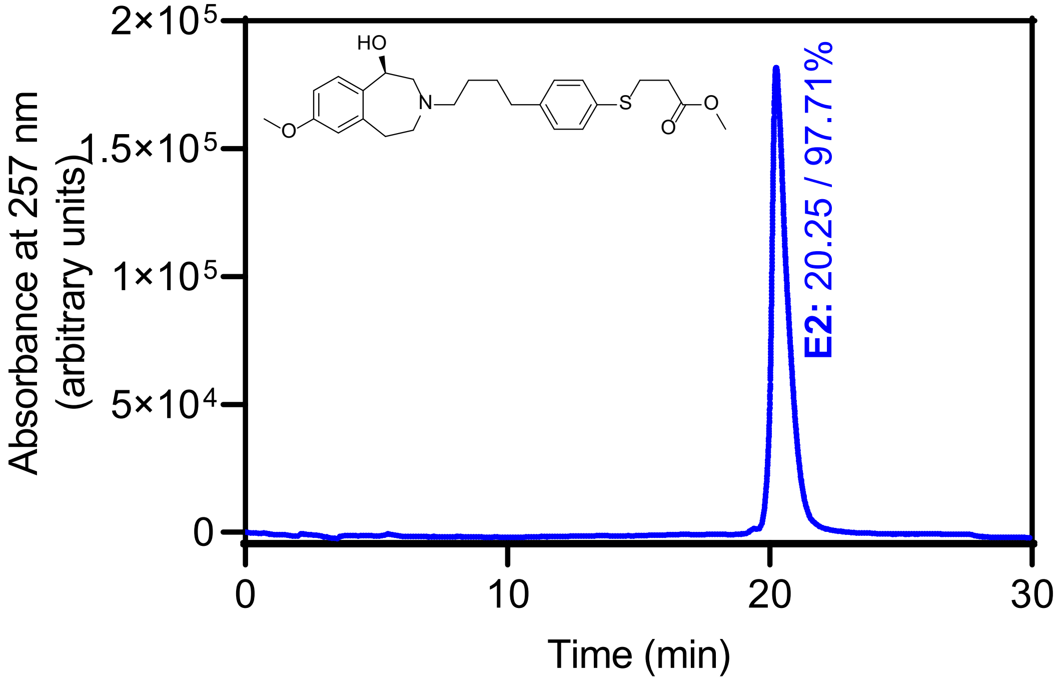


**Supplementary Figure 107.** Chiral HPLC chromatogram for **(*R*)-L19** (general method E)

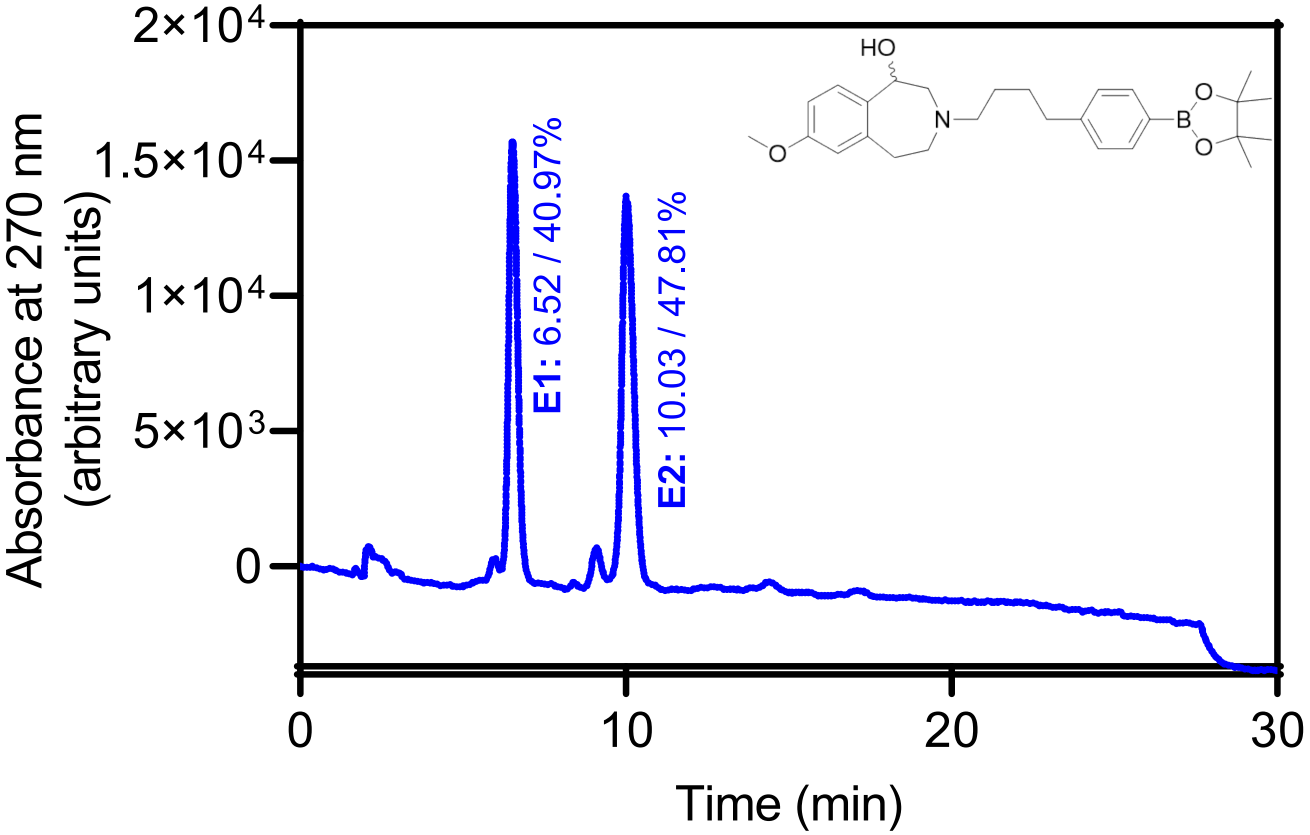


**Supplementary Figure 108.** Chiral HPLC chromatogram for **L20** (general method E).

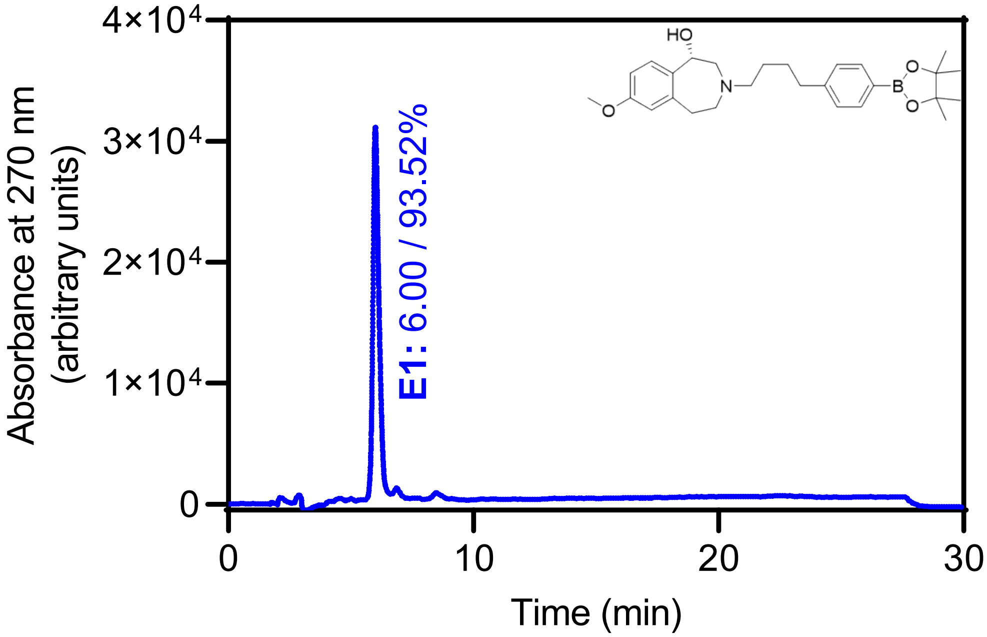


**Supplementary Figure 109.** Chiral HPLC chromatogram for **(*S*)-L20** (general method E).

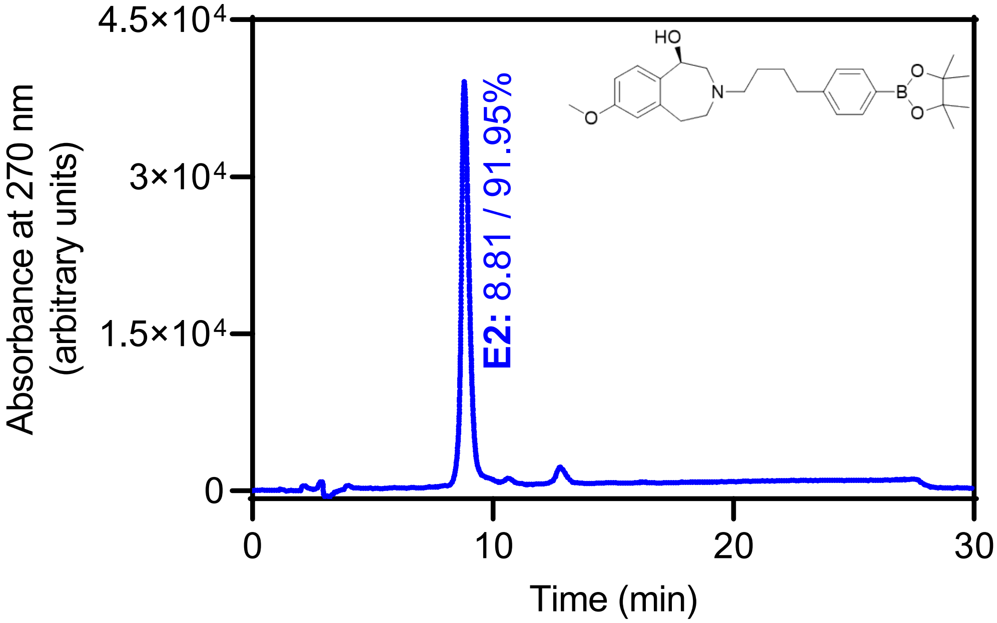


**Supplementary Figure 110.** Chiral HPLC chromatogram for **(*R*)-L20** (general method E).

## 7. Determination of Absolute Configuration for L3 (NR2B-SMe) Enantiomers

We purified each enantiomer of **L3** by HPLC on a preparative-size chiral (*S,S*) Whelk-O1 HPLC column (general method F). Each enantiomer was then analyzed by chiral chromatography (general method E). The first eluting enantiomer (E1) had $t_R$ = 8.5 min, and the second eluting enantiomer (E2) had $t_R$ = 13.2 min, the same order of elution as in the chiral HPLC purification by general method F.

Each enantiomer (E1 and E2) was subjected to vibrational circular dichroism (VCD) to obtain the spectrum (BioTools, Inc, Jupiter, FL) and subjected to quantum mechanics calculation using Gaussian 16 software suite (Genion Quantum Technologies, Inc., Rockville, MD) to obtain VCD spectra as a pair of mirror images (Supplementary Figure S109). The level of applied density functional theory (DFT) was B#LYP/6-311G(d,p) to locate the minimum energy structure of the molecule and eliminate non-physical imaginary vibrational modes. In addition, frequency and spectral analysis at a theory level of PBE0/6-311G(d,p) for infrared (IR)/VCD was used to generate the vibrational modes, IR intensities, and VCD rotational strengths for comparison with the experimental spectra.

The measured and calculated VCD spectra were matched to obtain absolute configuration and to assess similarity score (Supplementary Figures S110 and Figure S111). We found that the first eluting enantiomer in the chiral chromatography (E1) has *S*-configuration and the second eluting enantiomer (E2) has *R*-configuration.

Absolute optical rotation data for these enantiomers in chloroform and ethanol are reported in the manuscript Results and Discussion section as follows: (*S*)-**L3** chiral HPLC (general method E): $t_R$ = 8.10 min, 98.01%. $[\alpha]D^{20}$ = –39.02° (*c* 1.0, $CHCl_3$), $[\alpha]D^{20}$ = +8.89° (*c* 1.0, EtOH). (*R*)-**L3**: chiral HPLC (general method E): $t_R$ = 13.27 min, 99.19%. $[\alpha]D^{20}$ = +34.03° (*c* 1.0, $CHCl_3$), $[\alpha]D^{20}$ = –3.67° (*c* 1.0, EtOH).

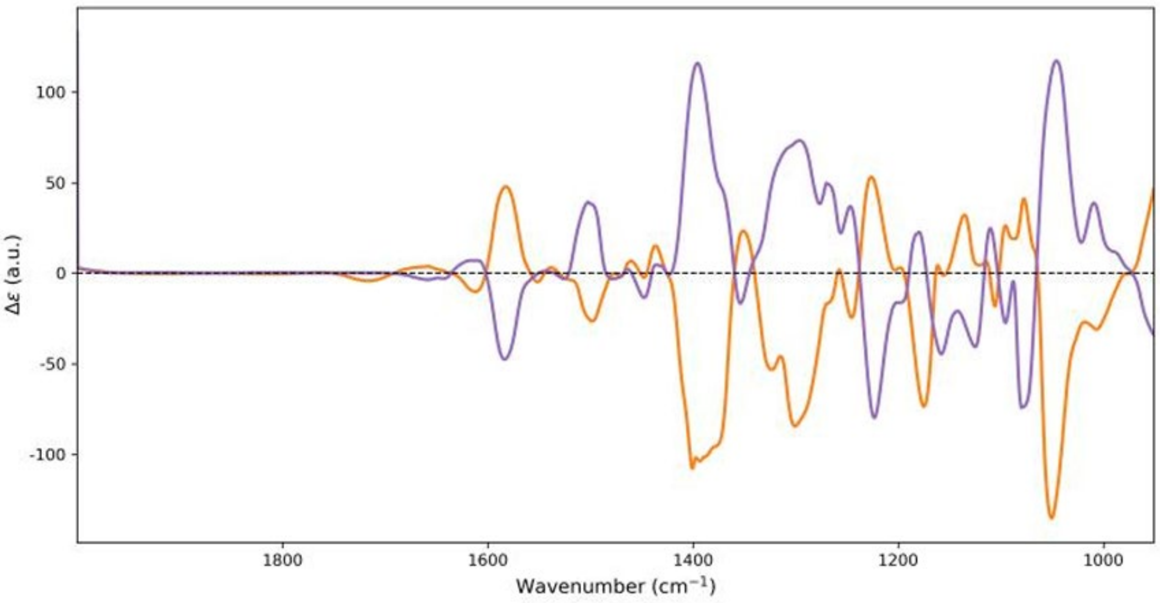


**Supplementary Figure S111**. The DFT-calculated VCD spectra for the enantiomers, **(*S*)-L3** [(*S*)-NR2B-SMe] (orange line) and **(*R*)-L3** [(*R*)-NR2B-SMe] (purple line).

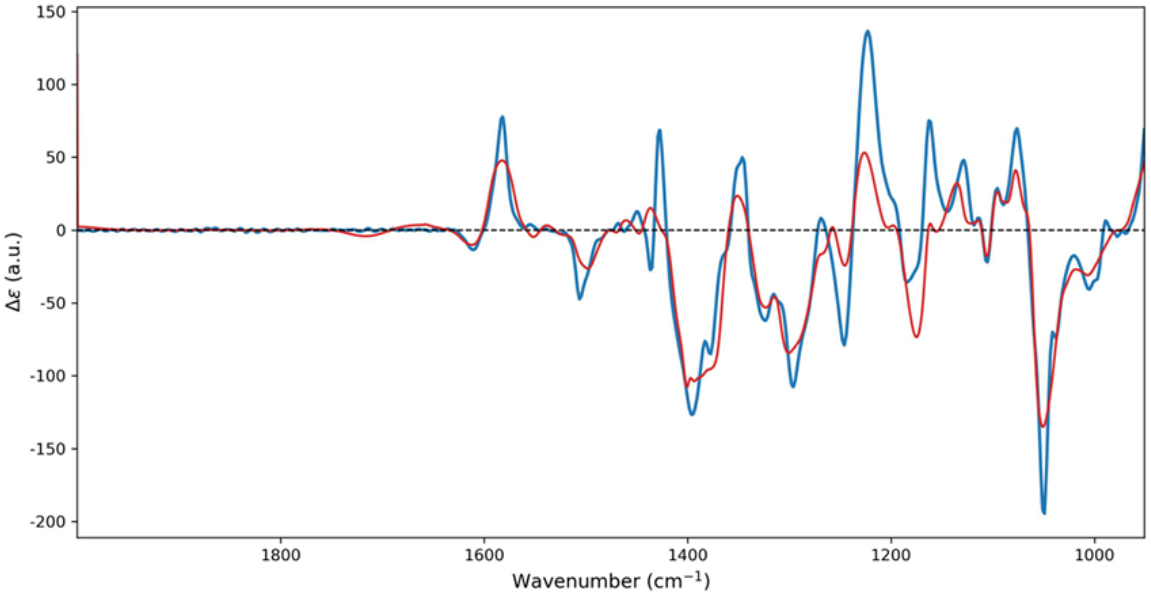


**Supplementary Figure S112**. The DFT-calculated VCD spectra for **(*S*)-L3** [(*S*)-NR2B-SMe] (red curve) versus the experimental VCD spectra (blue line) for the **L3** [NR2B-SMe] enantiomer eluting first in chiral chromatography on a (*S*,*S*) Whelk column ($t_R$ = 8.5 min) (the calculated similarity is 88.9%)

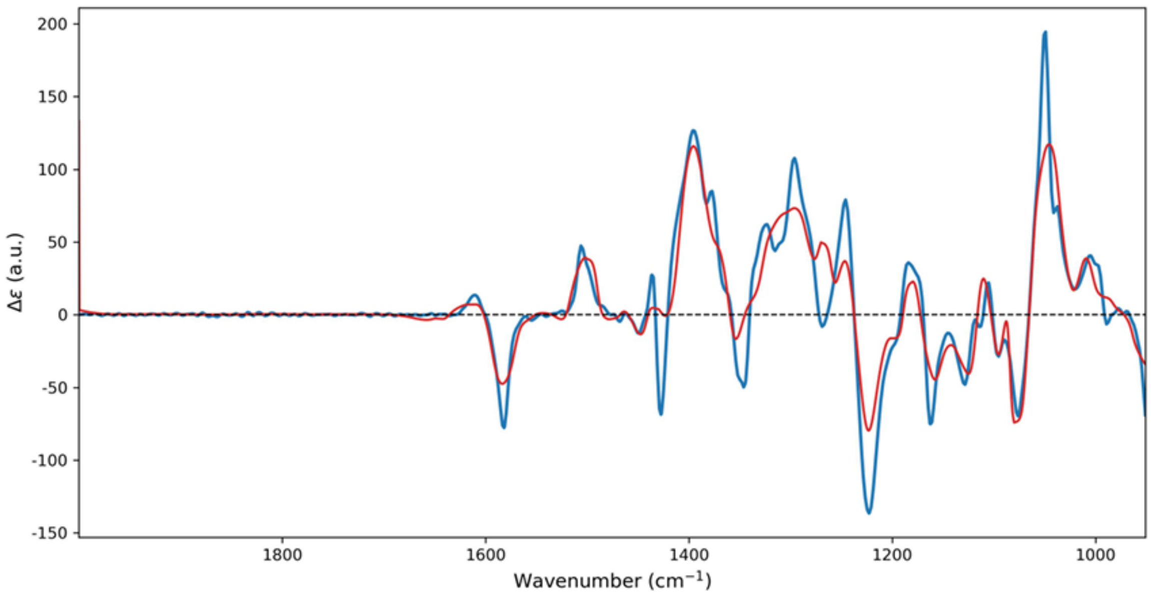


**Supplementary Figure S113**. The DFT-calculated VCD spectra for **(*R*)-L3** [(*R*)-NR2B-SMe] (red curve) versus the experimental VCD spectra for **L3** [NR2B-SMe] enantiomer (blue line), eluting second in chiral chromatography on an (*S*,*S*) Whelk column ($t_R$ = 13.8 min). (The calculated similarity is 92.0%).

A comparison of the experimental and calculated IR spectrum for **(*R*)-L3** is shown in supplementary Figure S112.

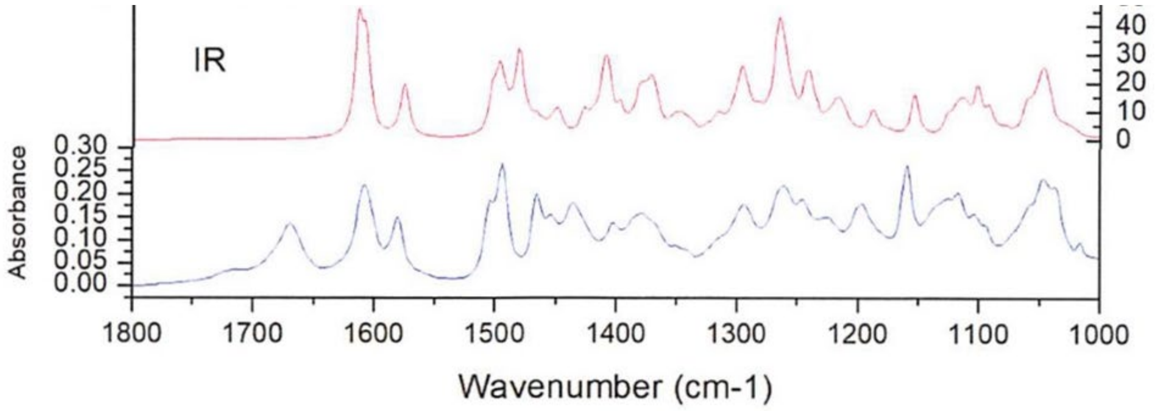


**Supplementary Figure S114.** Calculated (blue curve) and experimentally determined Boltzmann averaged (blue curve) infrared spectra for **(*R*)-L3.** Right axis labels are in units of molar absorptivity for calculated spectrum. The calculated similarity is 92.5%.

## 8. Radiochromatograms for Radiotracer Purifications and Analyses

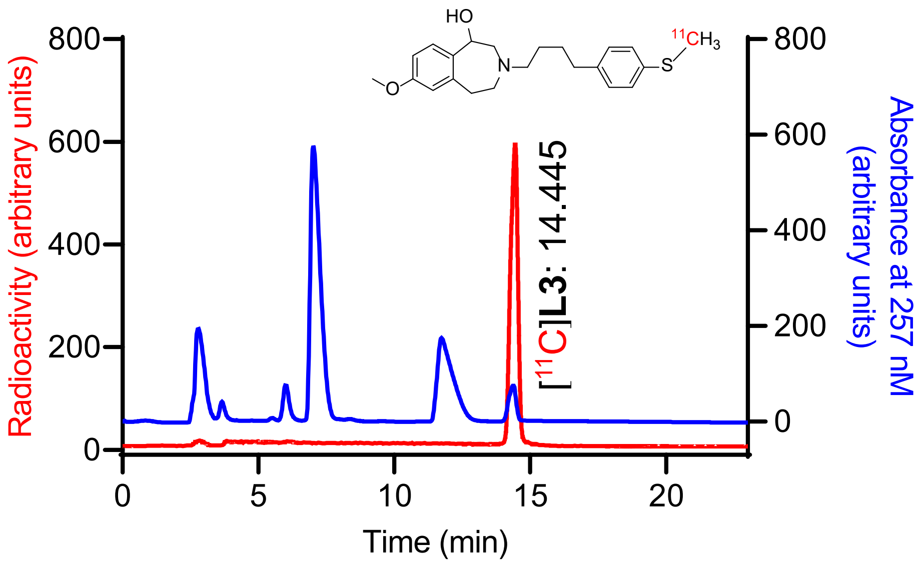


**Supplementary Figure 115.** HPLC chromatogram for purification of [$^{11}$C]***(S)*-L3** (general method H)

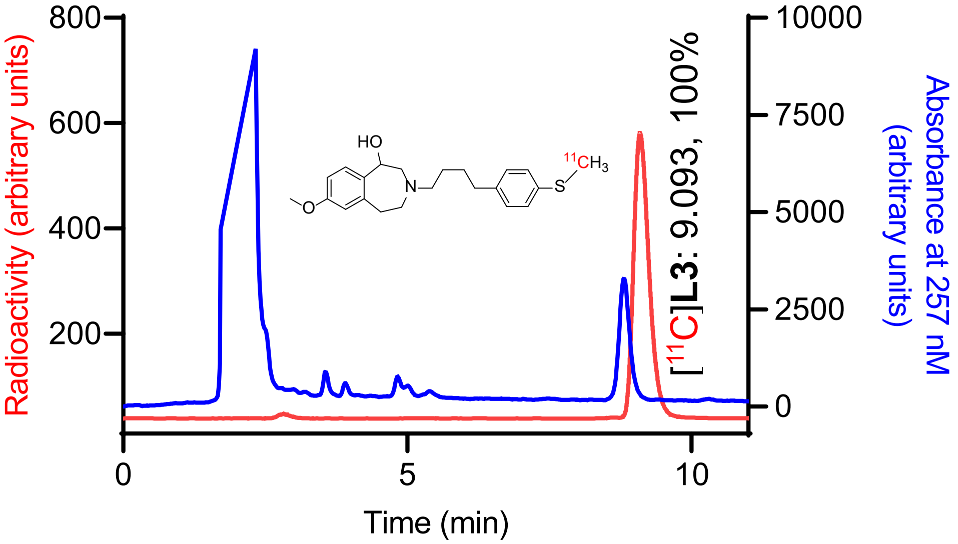


**Supplementary Figure 116.** Analytical HPLC chromatogram for [$^{11}$C]***(S)*-L3** (general method I)

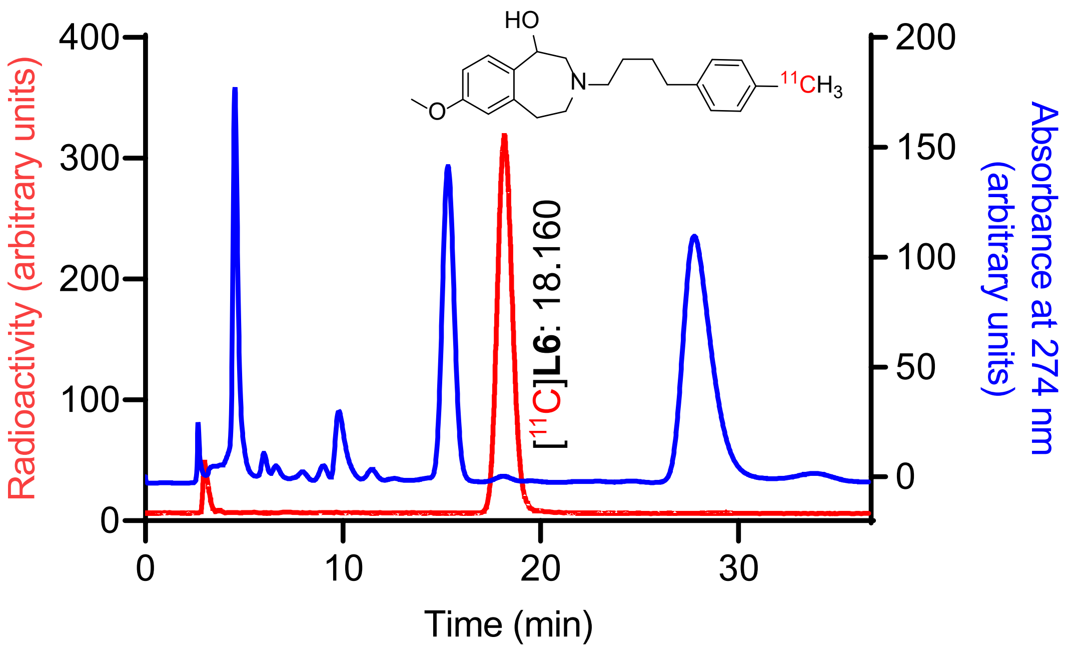


**Supplementary Figure 117.** HPLC chromatogram for purification of [11C]**(*S*)-L6** (general method H)

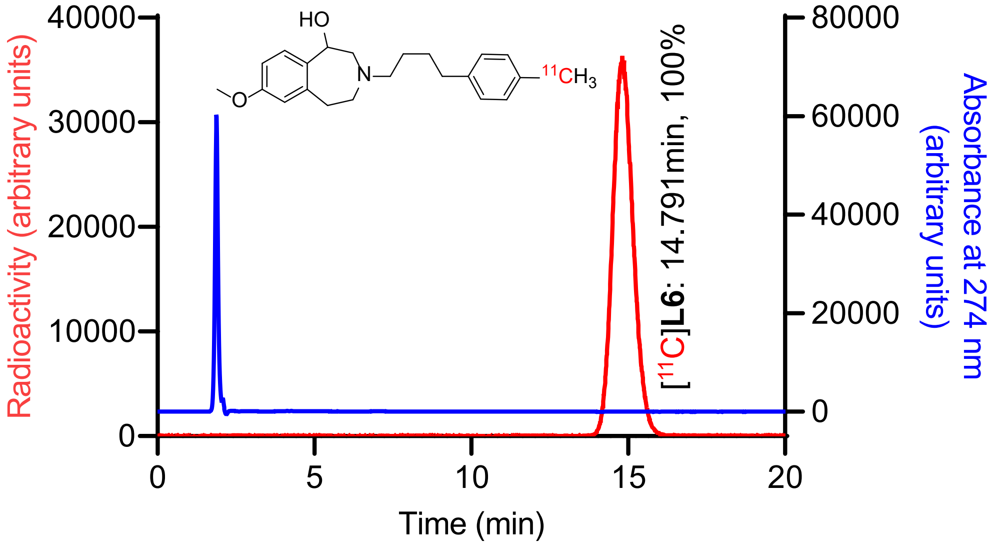


**Supplementary Figure 118.** Analytical HPLC chromatogram for [11C]**(*S*)-L6** (general method I).

# 9. Physicochemical Parameters and CNS PET MPO scores for Ligands L1–L20

**Supplementary Table 1.** Physicochemical parameters and CNS MPO and CNS PET MPO scores for ligands **L1**–**L20**

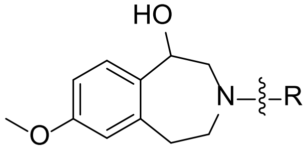


| Comp'd | Structure | cLogP | cLogD | tPSA (Å$^2$) | MW (Da) | pKa | CNS PET MPO |
|---|---|---|---|---|---|---|---|
| **L1** | 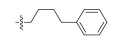 | 3.71 | 3.13 | 32.7 | 325.5 | 7.86 | 2.5 |
| **L2** | 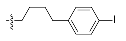 | 4.71 | 4.12 | 32.7 | 451.4 | 7.86 | 1.7 |
| **L3** | 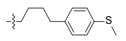 | 3.98 | 3.39 | 32.7 | 371.5 | 7.86 | 1.8 |
| **L4** | 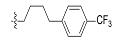 | 4.42 | 3.83 | 32.7 | 357.5 | 7.86 | 1.7 |
| **L5** | 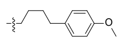 | 3.48 | 2.90 | 41.9 | 393.5 | 7.86 | 2.9 |
| **L6** | 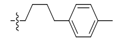 | 3.93 | 3.34 | 32.7 | 339.5 | 7.86 | 2.0 |
| **L7** | 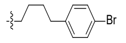 | 4.49 | 3.90 | 32.7 | 404.4 | 7.86 | 1.7 |
| **L8** | 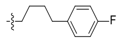 | 3.75 | 3.17 | 32.7 | 343.4 | 7.86 | 2.0 |
| **L9** | 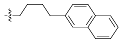 | 5.11 | 4.53 | 32.7 | 375.5 | 7.86 | 1.7 |
| **L10** | 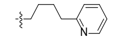 | 2.51 | 1.87 | 45.1 | 326.4 | 7.91 | 5.0 |
| **L11** | 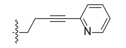 | 3.60 | 3.20 | 45.1 | 322.4 | 7.58 | 3.7 |
| **L12** | 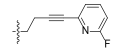 | 4.26 | 3.86 | 45.1 | 340.4 | 7.58 | 3.0 |
| **L13** | 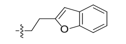 | 3.13 | 2.59 | 41.9 | 337.4 | 7.79 | 3.7 |
| **L14** | 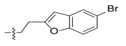 | 4.95 | 4.41 | 41.9 | 416.3 | 7.79 | 2.5 |
| **L15** | 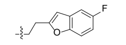 | 4.22 | 3.68 | 41.9 | 355.4 | 7.79 | 2.5 |
| **L16** | 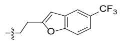 | 4.93 | 4.39 | 41.9 | 405.4 | 7.79 | 2.5 |
| **L17** | 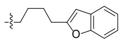 | 3.76 | 3.14 | 41.9 | 365.5 | 7.91 | 2.7 |
| **L18** | 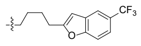 | 4.29 | 3.67 | 41.9 | 433.5 | 7.91 | 2.5 |
| **L19** | 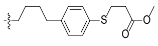 | 3.82 | 3.23 | 59.0 | 443.6 | 7.86 | 2.9 |
| **L20** | 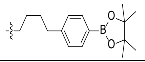 | 5.66 | 4.42 | 51.2 | 451.4 | 8.7 | |

## 10. GluN2B Ligand Binding Affinity Versus Computed Lipophilicity (cLog*D*)

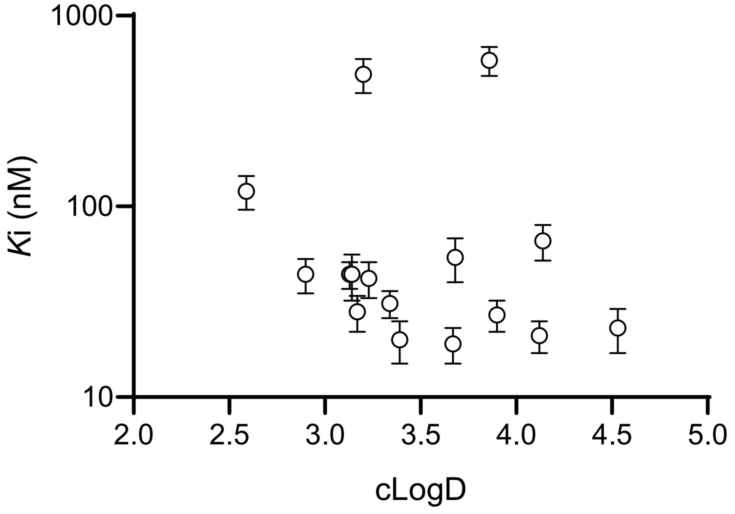


**Supplementary Figure 119.** GluN2B Ligand Binding Affinity Versus Computed Lipophilicity (cLog*D*). Data are taken from manuscript Table 1 ($K_i$ values are from automated PDSP assay). No discernible correlation is found.

## 11. Binding of GluN2B Ligands to Sigma Receptors

**Supplementary Table 2.** Inhibition of binding at $\sigma_1$ and $\sigma_2$ receptors by various GluN2B ligands at 10 $\mu$M concentration.

| Ligand | Inhibition at cellular $\sigma_1$ receptor[a] (%) | $\sigma_1$ receptor $K_i$ (nM) | Inhibition at cellular $\sigma_2$ receptor[b] (%) |
|---|---|---|---|
| **L1** (WMS 1405) | 89 ± 11 | | 88 ± 9 |
| **L3** | 84[c] | | 90[c] |
| (*R*)-**L3** | | 88[d] | |
| (*S*)-**L3** | | 24[d] | |
| **L5** | 94 ± 12 | | 91 ± 10 |
| **L6** | 90 ± 8 | | 91 ± 12[c] |
| (−)-**L6** | | 42[e] | |
| (+)-**L6** | | 130[e] | |
| **L7** | 84 ± 9 | | 90 ± 11 |
| **L8** | 58 ± 7 | | 77 ± 8 |
| **L9** | 84 ± 9 | | 94 ± 12 |
| **L11** | 66 ± 8 | | 74 ± 9 |
| **L12** | 41 ± 6 | | 56 ± 4 |

[a] Reference radioligand [$^3$H]Ifenprodil. [b] Reference radioligand [$^3$H]DTG. The assays were performed by the PDSP. [c] Data first reported in manuscript reference *24*. [d] Data first reported in manuscript reference 24. [e] Data first reported in reference 25.

# 12. Docking of (1*R*,2*S*)-Ifenprodil with the GluN1a/GluN2B Complex

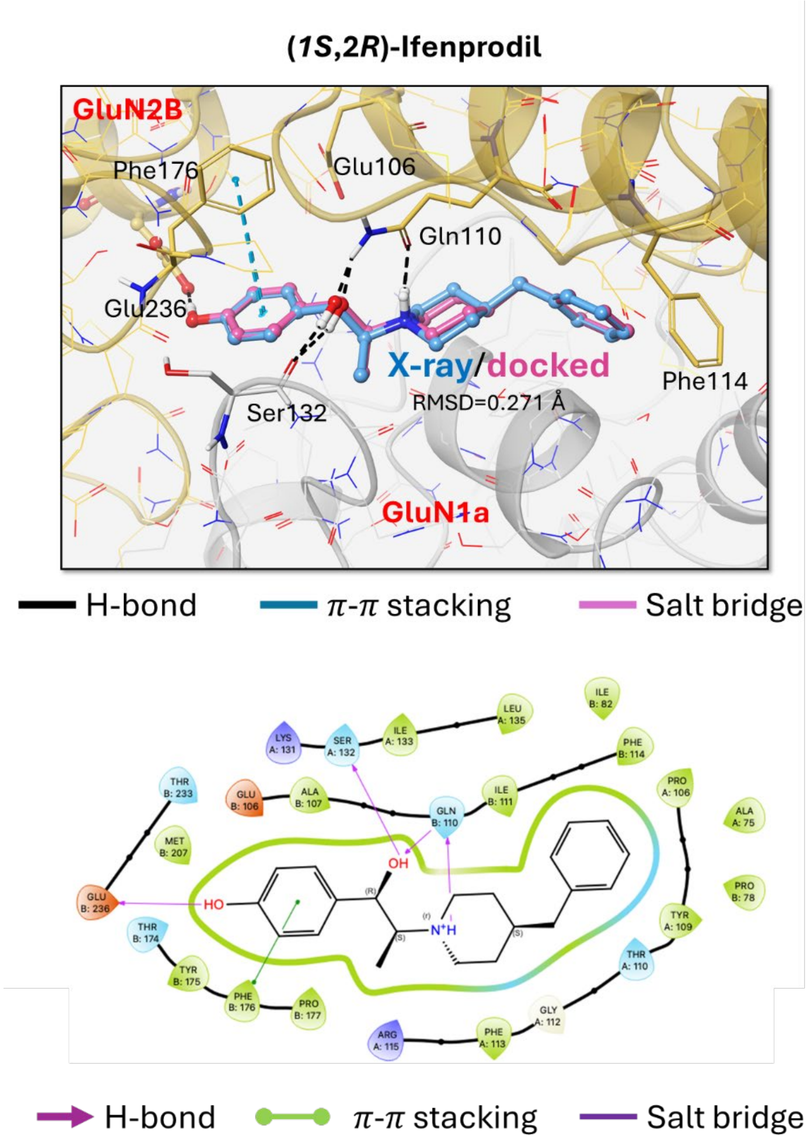


**Supplementary Figure 120**. Binding pose and interactions of (1*R*,2*S*)-ifenprodil with the GluN1a/GluN2B complex. The docked pose (magenta) closely matches the experimental structure (light blue), showing a similar binding mode and interactions within the pocket, with an RMSD of 0.271 Å. In the 3D interaction representations, GluN1a and GluN2B are shown in light gray and yellow, respectively, and residues involved in key interactions are highlighted as sticks. In the 2D interaction diagrams, positively charged, negatively charged, hydrophobic, and hydrophilic residues are colored dark orange, blue, green, and cyan, respectively.

## 13. Docking of (*R*)-L-19 and (*S*)-L19 with the GluN1a/GluN2B Complex

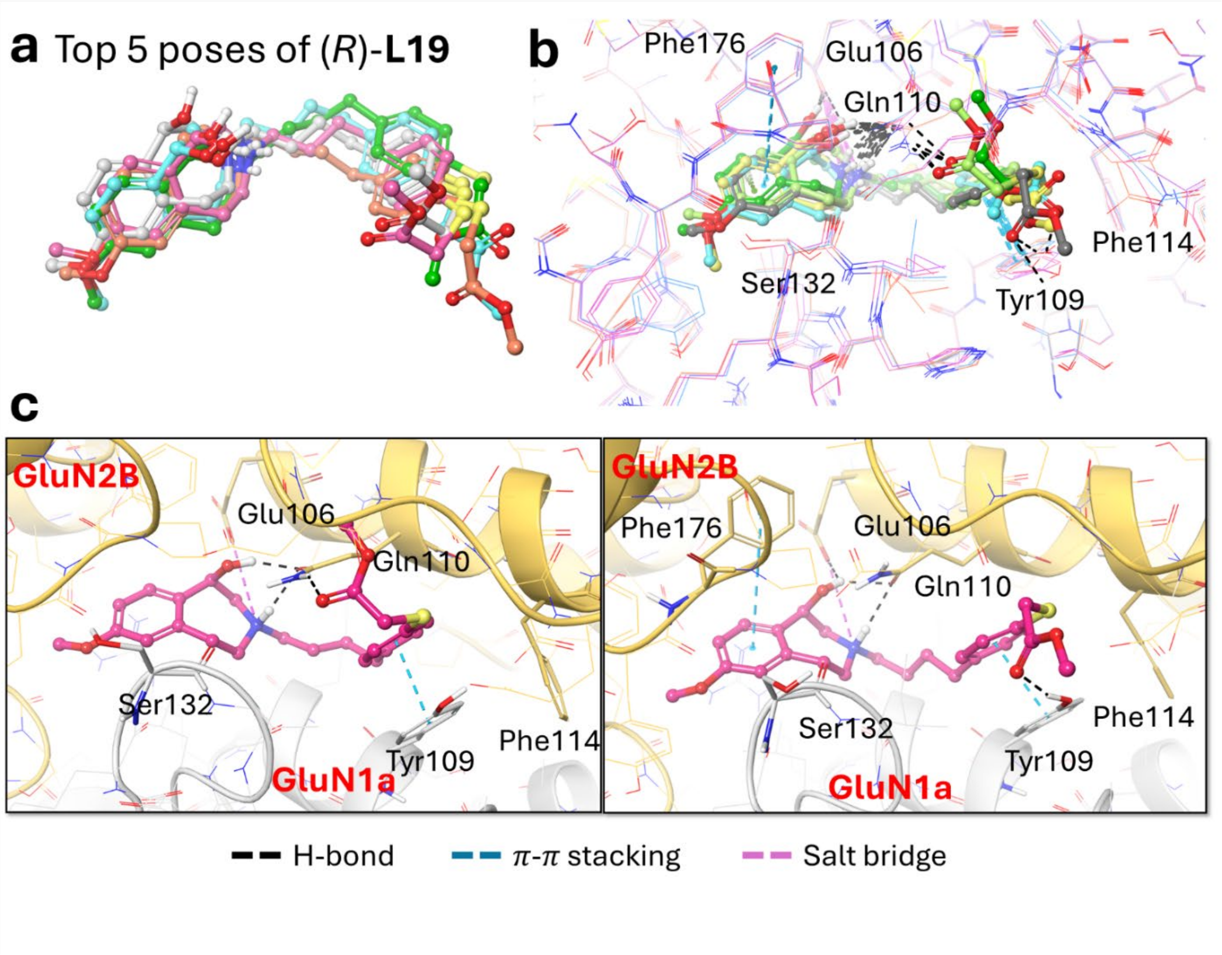


**Supplementary Figure 121.** Binding mode analysis of (*R*)-**L19**. (**a**) Overlay of the top five docking poses, illustrating the conformational variability of (*R*)-**L19** within the binding pocket. (**b**) Superposition of clustered poses highlighting key interactions with surrounding residues. (**c**) Representative binding poses showing detailed interactions of (*R*)-**L19** within the GluN1a/GluN2B binding pocket. The top five docking poses were evaluated using MM/GBSA (MM/GBSA, Molecular Mechanics Generalized Born Surface Area; multi parameter optimization)

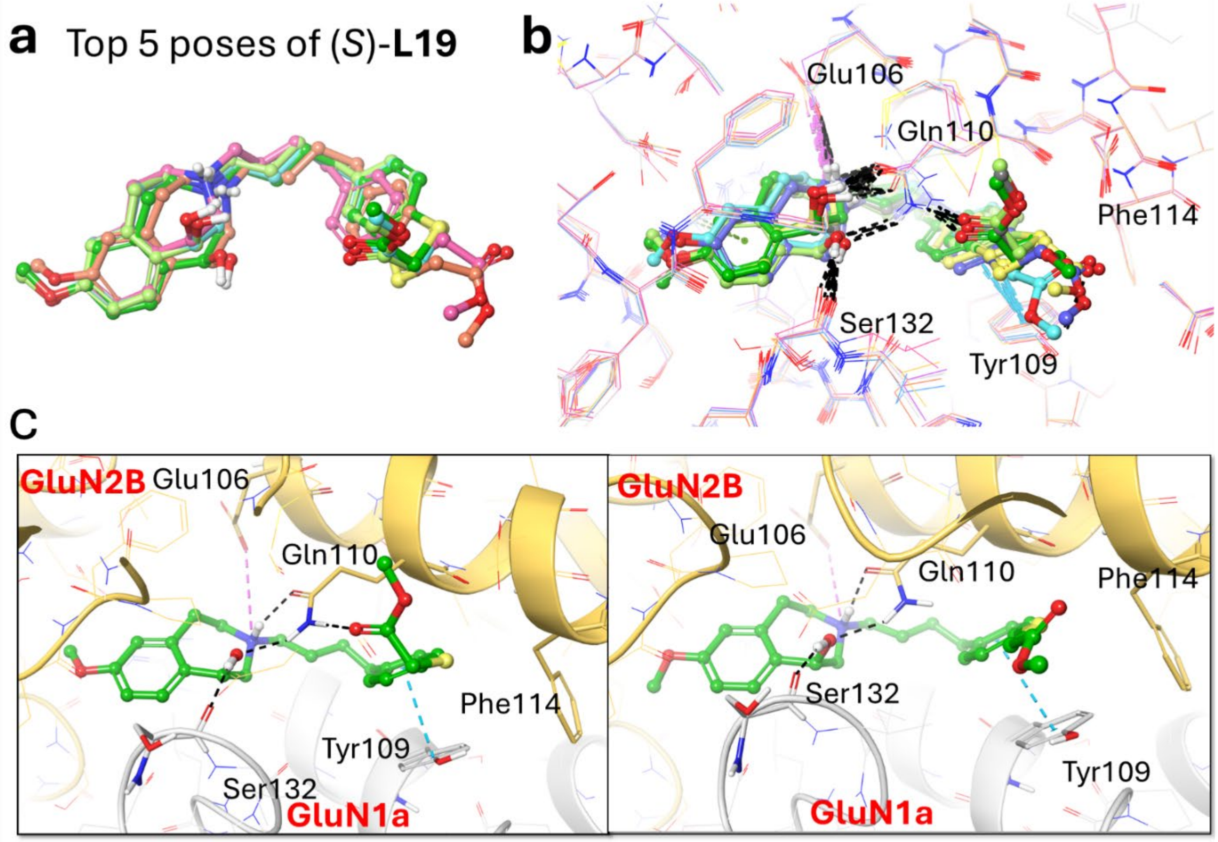


**Supplementary Figure 122.** Binding mode analysis of (*S*)-**L19**. (**a**) Overlay of the top five docking poses, illustrating the conformational variability of (*S*)-**L19** within the binding pocket. (**b**) Superposition of clustered poses highlighting key interactions with surrounding residues. (**c**) Representative binding poses showing detailed interactions of (*S*)-**L19** within the GluN1a/GluN2B binding pocket. The top five docking poses were evaluated using MM/GBSA.

## 14. References


1. Wang, L.; Jacobson, O.; Avdic, D.; Rotstein, B.H.; Weiss, I.D.; Collier, L.; Chen, X.; Vasdev, N.; Liang, S.H. *Ortho*-stabilized $^{18}$F-azido click agents and their application in PET imaging with single-stranded DNA aptamers. *Angew. Chem. Int. Ed*. **2015**, *54*, 12777-12781. https://doi.org/10.1002/anie.201505927.
2. Pingali, H.; Jain, M.; Shah, S.; Basu, S.; Makadia, P.; Goswami, A.; Zaware, P.; Patil, P.; Godha, A.; Giri, S.; Goel, A.; Patel, M.; Patel, H.; Patel, P. Discovery of a highly orally bioavailable *c*-5-[6-(4-methanesulfonyloxyphenyl)hexyl]-2-methyl-1,3-dioxane-*r*-2-carboxylic acid as a potent hypoglycemic and hypolipidemic agent. *Bioorg. Med. Chem. Lett.* **2008**, *18*, 5586-5590. https://doi.org/10.1016/j.bmcl.2008.08.112.
3. Sooriyaarachchi, S.; Chofor, R.; Risseeuw, M.D.P.; Bergfors, T.; Pouvez, J.; Dowd, C.S.; Maes, L.; Wouters, J.; Jones, A.; Van Calenbergh, S.; Mowbray, S.L. Targeting an aromatic hotspot in *Plasmodium falciparum* 1-deoxy-D-xylulose-5-phosphate reductoisomerase with β-arylpropyl analogues of fosmidomycin. *ChemMedChem*. **2016**, *11,* 2024-2036. https://doi.org/10.1002/cmdc.201600249.
4. Gu, Z.-S.; Zhou, A.; Xiao, Y.; Zhang, Q.W.; Li. J.-Q. Synthesis and antidepressant-like activity of novel aralkyl piperazine de-rivatives targeting SSRI/5-$HT_{1A}$/5-$HT_7$. *Eur. J. Med. Chem*. **2018**, *144*, 701-715. https://doi.org/10.1016/j.ejmech.2017.12.063.
5. Banerjee, T.S.; Paul, S.; Sinha, S.; Das, S. Synthesis of iboga-like isoquinuclidines: dual opioid receptor agonists having antino-ciceptive properties. *Bioorg. Med. Chem*. **2014**, *22*, 6062-6070. https://doi.org/10.1016/j.bmc.2014.09.001.
6. Cowart, M.; Faghih, R.; Curtis, M.P.; Gfesser, G.A.; Bennani, Y.L.; Black, L.A.; Pan, L.; Marsh, K.C.; Sullivan, J.P.; Esbenshade, T.A.; Fox, G.B.; Hancock, A.A. 4-(2-[2-(2(*R*)-Methylpyrrolidin-1-yl)ethyl]benzofuran-5-yl)benzonitrile and related 2-aminoethylbenzofuran $H_3$ receptor antagonists potently enhance cognition and attention. *J. Med. Chem*. **2005**, *48*, 38-55. https://doi.org/10.1021/jm040118g.
7. Huang, H.-M.; McDouall, J.J.W.; Procter, D. $SmI_2$-catalysed cyclization cascades by radical relay. *Nat. Catal.* **2019,** *2*, 211-218. https://doi.org/10.1038/s41929-018-0219-x.
